# Formal C−H Carboxylation of Unactivated Arenes

**DOI:** 10.1002/chem.202000515

**Published:** 2020-05-04

**Authors:** Ashot Gevorgyan, Kathrin H. Hopmann, Annette Bayer

**Affiliations:** ^1^ Department of Chemistry UiT The Arctic University of Norway 9037 Tromsø Norway; ^2^ Hylleraas Centre for Quantum Molecular Sciences Department of Chemistry UiT The Arctic University of Norway 9037 Tromsø Norway

**Keywords:** carbon dioxide, carboxylation, C−H activation, green solvent, late-stage functionalization

## Abstract

A formal C−H carboxylation of unactivated arenes using CO_2_ in green solvents is described. The present strategy combines a sterically controlled Ir‐catalyzed C−H borylation followed by a Cu‐catalyzed carboxylation of the in situ generated organoboronates. The reaction is highly regioselective for the C−H carboxylation of 1,3‐disubstituted and 1,2,3‐trisubstituted benzenes, 1,2‐ or 1,4‐symmetrically substituted benzenes, fluorinated benzenes and different heterocycles. The developed methodology was applied to the late‐stage C−H carboxylation of commercial drugs and ligands.

## Graphical abstract

**Late‐stage carboxylation**: Regioselective C−H carboxylation of unactivated arenes is now possible. C−H carboxylation can be realized in green solvents and can be used for the late‐stage carboxylation of practically valuable molecules.

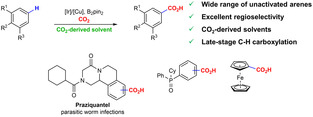

## Introduction

The last two decades have witnessed an exponential growth in the field of direct carbon–hydrogen (C−H) bond functionalization. A number of challenging carbon–carbon (C−C) and carbon–heteroatom (C−X) bond forming reactions can now be realized by direct transition metal‐catalyzed C−H activation.^1^ Well‐established C−H activations, which operate on unactivated systems with good functional group tolerance, can be applied to the late‐stage substitution of valuable and rather complex systems, such as commercial drugs and natural products.^2^ A holy grail in this field is the development of protocols that allow the direct carboxylation of C−H bonds with CO_2_; a sustainable and fossil‐free carbon source.^3^ The resulting products, carboxylic acids and their derivatives, are widespread structural motifs in commercial drugs and natural products.^4^ The use of CO_2_ as a carboxylating agent in C−H functionalizations is also attractive for the late‐stage isotopic labeling of pharmaceuticals and other biologically active molecules.^5^

**Scheme 1:**
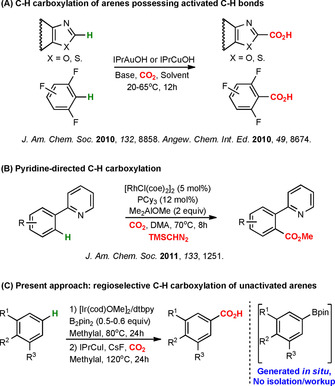
Previous works (A,^6^ B^7^) and present study (C).

Despite considerable progress in the field, known protocols for C−H functionalization with CO_2_ still have pronounced limitations. Most of them are working selectively only on activated molecules.3a Nolan,6a, 6b Hou6c and co‐workers have reported good regioselectivities for carboxylation of oxazoles and perfluorinated arenes (Scheme 1 A); however, this protocol is limited to activated aromatic systems with acidic C−H bonds. For unactivated systems, Iwasawa et al. found that Rh‐catalyzed C−H carboxylations can provide good regioselectivities, but only in the presence of nitrogen‐based directing groups (Scheme 1 B).^7^ Practical applications of directed C−H functionalizations are limited by the fact that directing groups may not be removable or modifiable.^8^

The aim of the work described here was to establish a method for regioselective C−H carboxylation of unactivated arenes with focus on substrates that are not reactive in currently known protocols.3a, 6, 7 We envisioned that the known reactivity and selectivity issues in the C−H carboxylation of unactivated arenes may be overcome by applying a sequential Ir/Cu‐catalyzed C−H transformation strategy: Regioselective C−H activation may be achieved through a sterically controlled Ir‐mediated C−H borylation,^9^, ^10^ which we hypothesized could be followed by a Cu‐catalyzed carboxylation of the in situ generated organoboronates (Scheme 1 C). If successful, our strategy would provide new opportunities for formal C−H carboxylation of real‐life systems, such as pharmaceuticals. Besides using CO_2_ as a sustainable carboxylating agent, we further decided to ensure that our procedure would be applicable in green solvents, making our protocol relevant in the context of green chemistry.

## Results and Discussion

In our proposed strategy (Scheme 1 C), a key intermediate is the in situ generated organoboronate, which subsequently is carboxylated. To evaluate the feasibility of this protocol, we started our investigation with the analysis of the carboxylation of a model organoboronate, phenylboronic acid pinacol ester **b1**. During optimization of this reaction step (Supporting Information, Tables S1–S4, Scheme S1), particular attention was paid to the use of renewable/sustainable solvents, such as polyethylene glycol (PEG400), CO_2_‐derived diethyl carbonate (DEC), dimethyl carbonate (DMC), methylal^11^ and biomass‐derived γ‐valerolactone (GVL) and 2‐methyltetrahydrofuran (2MeTHF).

An exhaustive screening of various parameters showed that the carboxylation of the proposed organoboronate intermediate is possible in green solvents. The best yields of benzoic acid **p1** were obtained in CO_2_‐derived DEC (86 %), DMC (89 %) and methylal (81 %) using a Cu‐catalyst generated from CuI and the carbene ligand 1,3‐bis(2,6‐diisopropylphenyl)imidazolium chloride (IPrHCl), in combination with CsF as base (Supporting Information, Table S1–S4).^12^, ^13^ The use of common organic solvents like THF (76 %), dioxane (76 %), toluene (18 %) and DMF (64 %) did not improve the yields compared to renewable CO_2_‐derived solvents (Supporting Information, Table S4).

On basis of the successful carboxylation of the organoboronate intermediate, we then tested our proposed one‐pot C−H carboxylation strategy (Scheme 1 C), setting out from the unactivated arene substrate 1,3‐dimethoxybenzene **r2** (Table 1). For in situ generation of the corresponding organoboronate **b2**, we first evaluated different iridium complexes (entries 3, 11–14, Supporting Information, Table S5). We found that the proposed strategy shows exceptional regioselectivity for the substrate **r2** when using (1,5‐cyclooctadiene)(methoxy)iridium(I) dimer ([Ir(cod)OMe]_2_) as catalyst precursor and 4,4′‐di‐*tert*‐butyl‐2,2′‐bipyridyl (dtbpy) as ligand in only 0.25 and 0.5 mol % loading, respectively (entries 2–10).^9^ For our formal C−H carboxylation, a screening of the best solvents identified above revealed that the highest yield of the carboxylation product **p2** is obtained in ethers like methylal (73 %), THF (70 %) or 2MeTHF (61 %) (entries 3, 8, 9).

**Table 1:** Optimization of formal C−H carboxylation.^[a]^

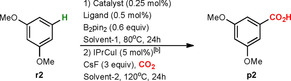			
Entry	Catalyst/ Ligand	Solvent‐1 [mL]/ Solvent‐2 [mL]	Yield [%]^[c]^
1	[Ir(cod)OMe]_2_/ dtbpy	DEC (4)/ DEC (4)	0^[d]^
2	[Ir(cod)OMe]_2_/ dtbpy	Methylal (4)/ Methylal (4)	69^[d]^
3	[Ir(cod)OMe]_2_/ dtbpy	Methylal (4)/ Methylal (4)	73
4	[Ir(cod)OMe]_2_/ dtbpy	Methylal (4)/ DEC (4)	70
5	[Ir(cod)OMe]_2_/ dtbpy	Methylal (4)/ DEC (4)	72^[e]^
6	[Ir(cod)OMe]_2_/ dtbpy	Methylal (3)/ DEC (6)	68
7	[Ir(cod)OMe]_2_/ dtbpy	Methylal (4)/ DMC (4)	49
8	[Ir(cod)OMe]_2_/ dtbpy	THF (4)/ THF (4)	70
9	[Ir(cod)OMe]_2_/ dtbpy	2MeTHF (4)/ 2MeTHF (4)	61
10	[Ir(cod)OMe]_2_/ dtbpy	GVL (3)/ GVL (3)	31
11	[Ir(cod)Cl]_2_/ dtbpy	Methylal (4)/ Methylal (4)	0
12	[Cp*IrCl_2_]_2_/ dtbpy	Methylal (4)/ Methylal (4)	0
13	[Ir(cod)OMe]_2_/ 1,10‐phen	Methylal (4)/ Methylal (4)	0
14	[Ir(cod)OMe]_2_/ Me_4_phen	Methylal (4)/ Methylal (4)	47

[a] Reaction conditions: 1) **r2** (2.170 mmol), solvent‐1 (3–4 mL), catalyst (0.25 mol %), ligand (0.5 mol %), B_2_pin_2_ (0.6 equiv), 80 °C, 24 h. 2) CuI (5 mol %), IPrHCl (6 mol %), NaO*t*Bu (6 mol %), solvent‐2 (3–6 mL), CsF (3 equiv), CO_2_ (120 mL), 120 °C, 24 h. [b] The catalyst was generated in situ. [c] Isolated yields. [d] Both steps were performed for 18 h. [e] C−H borylation step was performed for 36 h. [Ir(cod)OMe]_2_=(1,5‐cyclooctadiene)(methoxy)iridium(I) dimer; [Ir(cod)Cl]_2_=bis(1,5‐cyclooctadiene)diiridium(I) dichloride; [Cp*IrCl_2_]_2_=pentamethylcyclopentadienyliridium(III) chloride dimer; dtbpy=4,4’‐di‐*tert*‐butyl‐2,2’‐dipyridyl; 1,10‐phen=1,10‐phenanthroline; Me_4_phen=3,4,7,8‐tetramethyl‐1,10‐phenanthroline.

**Scheme 2:**
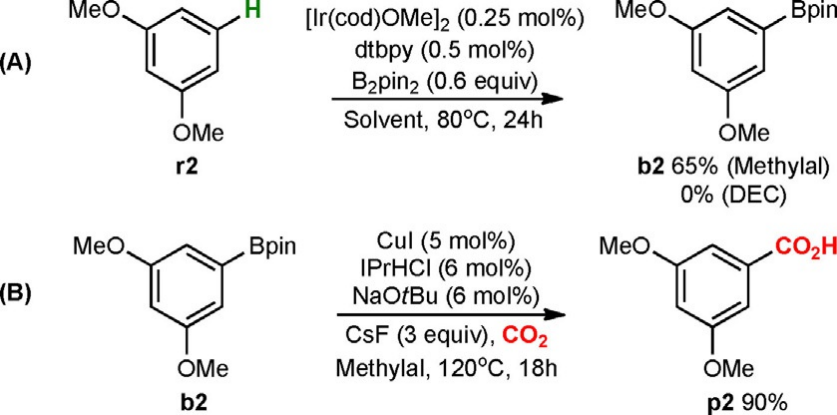
Stepwise analysis of formal C−H carboxylation (**r**=reactant (arene), **b**=boronate (intermediate), **p**=product (carboxylic acid)).

Carbonates were not suitable as solvents for the formal C−H carboxylation (Table 1, entry 1), even though they were the best solvents for the carboxylation of the intermediate organoboronate (Supporting Information, Table S1–S4). Stepwise analysis of the reaction showed that the Ir‐catalyzed C−H borylation of **r2** is not working in carbonates (Scheme 2 A). A possible explanation may be that carbonates are being reduced by B_2_pin_2_/HBpin (bis(pinacolato)diboron/pinacolborane), thus consuming the reagent of C−H borylation.^14^

In contrast to Ir‐catalyzed C−H borylation in carbonates as solvents, borylation of **r2** in methylal leads to the corresponding organoboronate **b2** in 65 % isolated yield (Scheme 2 A). The following Cu‐catalyzed carboxylation of **b2** in methylal provided **p2** in 90 % isolated yield (Scheme 2 B). Application of a two‐solvent system, applying methylal in the C−H borylation step and exchanging the solvent to carbonates (DEC or DMC) for the carboxylation step, gave lower yields than the reaction performed using only methylal as solvent (Table 1, entry 3 versus 4 and 7). The yields for the two‐solvent system can be slightly improved (72 %) by extending the initial C−H borylation in methylal to 36 h (Table 1, entry 5).

**Scheme 3:**
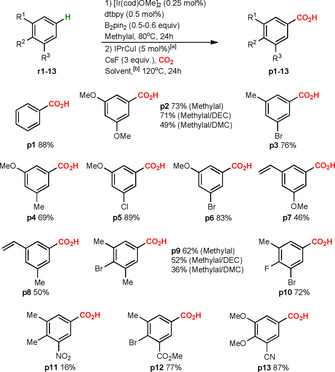
Formal C−H carboxylation of benzene, 1,3‐ and 1,2,3‐substituted arenes. [a] The catalyst was generated in situ. [b] If not otherwise mentioned, the reaction was performed in methylal.

**Scheme 4:**
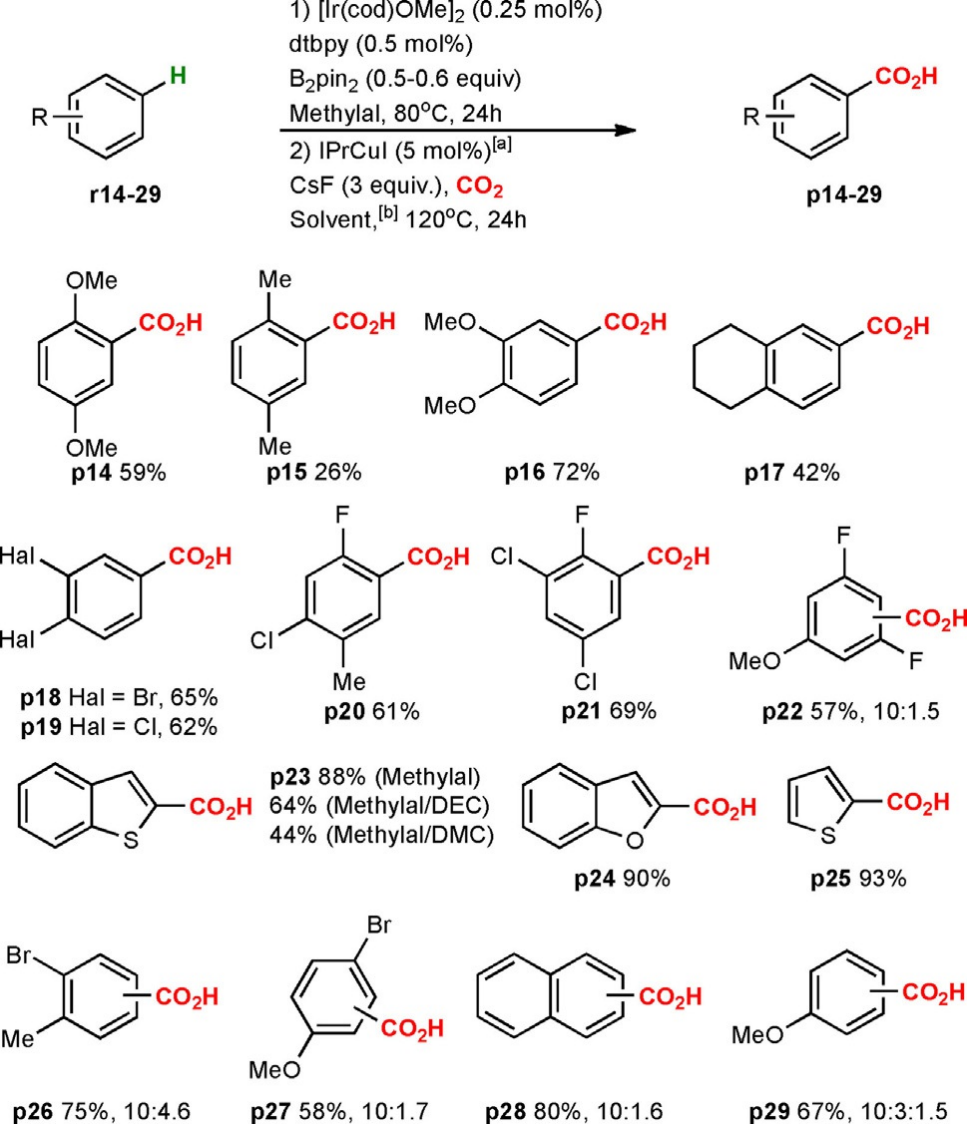
Formal C−H carboxylation of various arenes. [a] The catalyst was generated in situ. [b] If not otherwise mentioned, the reaction was performed in methylal.

With these promising results in hand, we turned to the analysis of the scope and limitations of the new formal C−H carboxylation method (Schemes 3 and 4). The sequential Ir/Cu‐catalyzed reaction was examined on a wide range of unactivated arenes, including benzene, 1,2‐, 1,4‐, 1,3‐ and 1,2,3‐substituted arenes, and heterocycles (for a full overview of used starting materials see Supporting Information, Scheme S2). We found that the formal C−H carboxylation of benzene **r1** provide benzoic acid **p1** in 88 % yield (Scheme 3). Further, both 1,3‐disubstituted arenes **r2**–**8** as well as 1,2,3‐trisubstituted arenes **r9**–**13** could successfully be carboxylated at position 5. The corresponding carboxylic acids **p2**–**13** were observed as single regioisomers in 16–89 % yields.

Excellent regioselectivities were observed for carboxylations of 1,2‐ and 1,4‐symmetrically substituted benzenes **r14**–**19** (**p14**–**19**, 26–72 %), and heterocycles like benzothiophene **r23** (**p23**, 88 %), benzofuran **r24** (**p24**, 90 %) and thiophene **r25** (**p25**, 93 %) (Scheme 4). The observed regioselectivities indicate that the Ir‐catalyzed C−H borylation occurs at the least sterically hindered position, in agreement with previous reports.^9^ Likewise, monofluorinated arenes **r20**–**22** were carboxylated at the expected *ortho* position to fluorine (**p20**–**22**, 57–69 %).10b The regioselectivity of the reaction was slightly reduced for 1,2‐ and 1,4‐unsymmetrically substituted benzenes **r26** (**p26**, 75 %; 10:4.6) and **r27** (**p27**, 58 %; 10:1.7) and for naphthalene **r28** (**p28**, 80 %; 10:1.6). The C−H carboxylation of anisole **r29** (monosubstituted benzene) lead to a mixture of *meta*‐, *para*‐ and *ortho*‐carboxylated products **p29** in 67 % overall yield and 10:3:1.5 ratio. In spite of the moderate regioselectivities observed for products **p26**–**29**, our protocol provides improved results compared to other methods available for C−H carboxylation of this type of unactivated arenes.3a, 7b In general, it may be noted that for the majority of carboxylic acids described in the Schemes 3 and 4, no other obvious synthesis approach is currently available, and many of the obtained products have not been previously described in the literature (e.g. **p7**, **p8**, **p10**, **p12**, **p13**, **p18**). For practical late‐stage applications, it is relevant to note that our formal C−H carboxylation strategy shows excellent functional group tolerance, with successful carboxylation of halogenated arenes, styrenes, aromatic nitriles and esters. Difficulties were observed only for nitro‐substituted systems (**r11**), in which the nitro group can be reduced by B_2_pin_2_ or in situ generated HBpin.^9^, ^10^

**Scheme 5:**
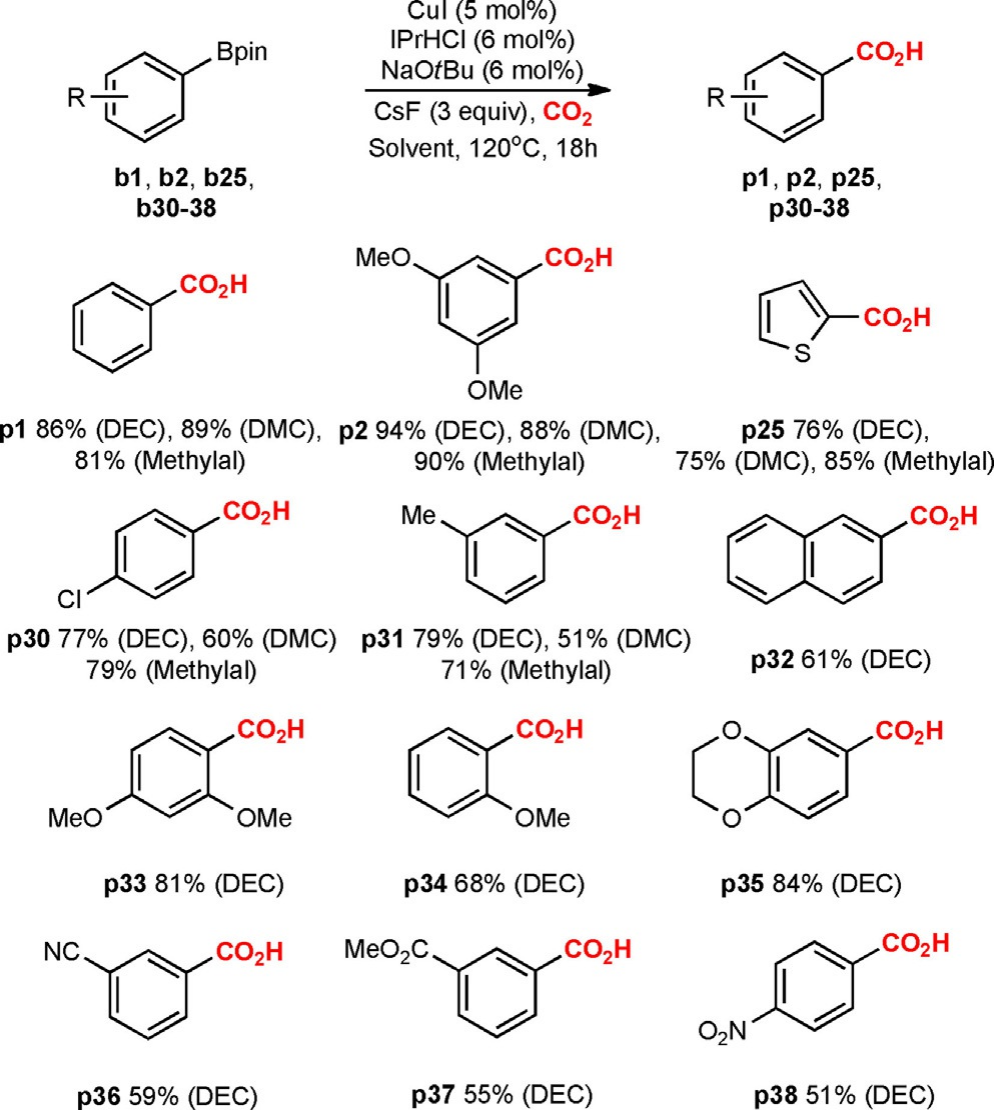
Analysis of the substrate dependency of the Cu‐catalyzed carboxylation step.

Previous reports indicate that the Ir‐catalyzed C−H borylation shows no preference for electron‐rich or ‐deficient systems and is mainly controlled by steric effects.^9^, ^10^ To further understand any limitations of the developed sequential Ir/Cu‐catalyzed method, we examined the substrate dependency of the Cu‐catalyzed carboxylation step on several arylboronic acid pinacol esters **b1**, **b2**, **b25**, **b30**–**38** (Scheme 5). Electron‐deficient arylboronates turned out to provide less yields (**p30** 77 %, **p36** 59 %, **p37** 55 %, **p38** 51 %) compared to electron‐rich systems (**p2** 94 %, **p25** 76 %, **p33** 81 %, **p34** 68 %, **p35** 84 %). However, the carboxylation does not appear to be strongly affected by steric hindrance; thus, *ortho*‐substituted organoboronates can be carboxylated in moderate to good yields (**p33** 81 %, **p34** 68 %).

The influence of solvent on the outcome of the carboxylation of arylboronic acid pinacol esters showed that the best yields are achieved in DEC, although in some cases the yield differences between the solvents were negligible (**p1**, **p2**, Scheme 5). Thiophene‐2‐boronic acid pinacol ester **b25** and **b30** were the single exception, providing best results in methylal (Scheme 5, Supporting Information, Scheme S2). However, note that for the sequential Ir/Cu‐catalyzed formal C−H carboxylation, the CO_2_‐derived methylal proved to be the best, as evaluated for several reactions (**p2**, **p9**, **p23**, Scheme 3 and 4, Supporting Information, Table S4).

**Scheme 6:**
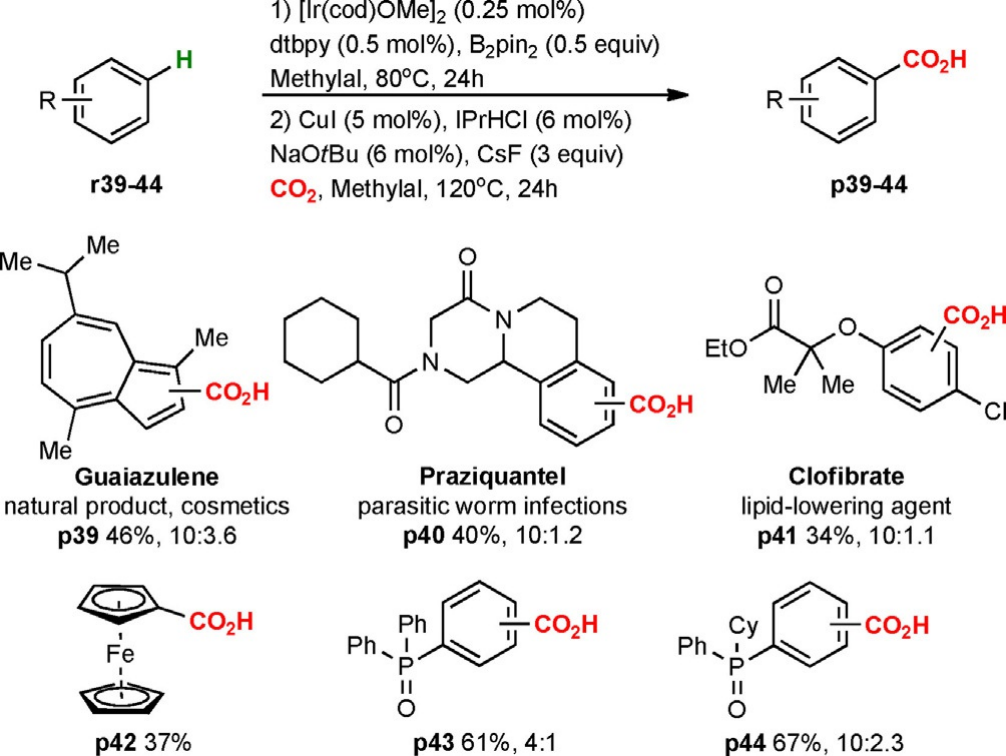
Late‐stage C−H carboxylation.

The exceptional substrate scope mainly based on unactivated arenes and the excellent functional group tolerance allowed us to use the formal C−H carboxylation for the late‐stage functionalization of complex and practically valuable systems, such as commercial drugs and natural products (Scheme 6).^2^, ^10^, ^15^ For example, we could carboxylate the natural product guaiazulene **r39** (cosmetic ingredient) with 46 % yield of **p39** as a mixture of regioisomers (10:∼4). The commercial drugs praziquantel **r40** (worm treatment) and clofibrate **r41** (lipid‐lowering agent) were carboxylated to provide, respectively, **p40** (40 %) and **p41** (34 %) in decent yields and with better regioselectivity (10:∼1). Observed regioselectivities are similar to previously reported late‐stage functionalizations of guaiazulene, praziquantel and clofibrate, which in all cases provided mixtures of regioisomers.10e, 15e

The developed methodology was further evaluated for the late‐stage carboxylation of organometallics and phosphine ligands (Scheme 6, **p42** to **p44**), as the generation of carboxylated organometallics and phosphines could be highly relevant to the production of water‐soluble homogeneous catalysts.^16^ The formal C−H carboxylation of ferrocene **r42** provided **p42** in 37 % yield, as a single product. All attempts to introduce a carboxyl group directly into unprotected phosphines failed, however, phosphine oxides were successfully carboxylated by our method. For triphenylphosphine oxide **r43** and diphenyl(cyclohexyl)phosphine oxide **r44**, the reaction gave a regioisomeric mixture of carboxylated phosphine oxides **p43**, **p44** in 61 % and 67 % yields, respectively. To the best of our knowledge, the late‐stage C−H functionalization of phosphine ligands has not been described before.

## Conclusions

We have developed a robust and versatile strategy for a formal C−H carboxylation of unactivated arenes. The present strategy consists of Ir‐catalyzed C−H borylation and subsequent Cu‐catalyzed carboxylation of in situ generated organoboronates. The protocol does not require any workup or purification during the two steps. The formal C−H carboxylation reaction proceeds with remarkable regioselectivity for 1,3‐disubstituted and 1,2,3‐trisubstituted benzenes, 1,2‐ and 1,4‐symmetrically substituted benzenes, fluorinated benzenes and several heterocycles. The developed methodology shows excellent functional group tolerance and can be applied for the late‐stage C−H functionalization of commercial drugs and ligands. Thus, the present protocol has capacity for creating structurally diverse molecular libraries for modern medicinal chemistry and drug discovery, avoiding parallel de novo synthesis.

Evaluation of a range of green solvents showed that the formal C−H carboxylation can be conducted in CO_2_‐derived solvents, which perform better than common organic solvents for these reactions. We believe that the present methodology will open a new chapter for the application of CO_2_ as a sustainable carboxylating agent in medicinal chemistry, material sciences and catalysis.

## Experimental Section

*General experimental procedure for formal C−H carboxylation of arenes* (Schemes 3, 4, and 6). Inside a glove box, a 45 mL pressure tube was charged with appropriate arene (2 mmol), dry methylal (4 mL), [Ir(cod)OMe]_2_ (0.25 mol %), dtbpy (0.5 mol %) and B_2_pin_2_ (0.5 equiv for benzene **r1** and arenes **r14**–**22**, **r25**–**29**, **r39**–**41**, **r43**, **r44**; 0.6 equiv for arenes **r2**–**13**, heterocycles **r23**, **r24** and ferrocene **r42**). The pressure tube was closed with a suitable cap, removed from the glove box and stirred at 80 °C for 24 h. Next, the pressure tube was transferred into the glove box in which CsF (3 equiv) and a previously prepared solution of Cu‐catalyst [the mixture of CuI (5 mol %), IPrHCl (6 mol %) and NaO*t*Bu (6 mol %) in appropriate dry solvent (4 mL) was stirred at 20 °C for 30 min] were added to the reaction mixture at 20 °C. The pressure tube was closed with the cap and removed from the glove box. Afterwards, CO_2_ (120 mL) was added through a syringe, followed by stirring of the reaction mixture at 120 °C for 24 h. Next, the reaction mixture was diluted with 30 mL Et_2_O and transferred into a 500 mL separating funnel. The resulting mixture was extracted with 30 mL saturated NaHCO_3_ solution (3 times). The resulting basic aqueous extracts were combined, washed with 15 mL Et_2_O (3 times), acidified (50–55 mL 6 m HCl) and extracted with 30 mL Et_2_O (3 times). The resulting solution of Et_2_O was distilled to dryness to give corresponding acid.

Other renewable solvents like 2MeTHF, diethoxymethane or methylal can replace Et_2_O without any noticeable difference (the difference was in the range ±3 %). Similarly, saturated solution of NaHCO_3_ can be replaced by 2 m solution of KOH.

## Conflict of interest

The authors declare no conflict of interest.

## Supporting information

As a service to our authors and readers, this journal provides supporting information supplied by the authors. Such materials are peer reviewed and may be re‐organized for online delivery, but are not copy‐edited or typeset. Technical support issues arising from supporting information (other than missing files) should be addressed to the authors.

Supplementary
