# Supplementary material for: Formal C−H Carboxylation of Unactivated Arenes
Source: Chemistry. 2020 May 4;26(27):6064–9. doi: 10.1002/chem.202000515 (PMC7317471; doi:10.1002/chem.202000515)

Chemistry–A European Journal

Supporting Information

Formal C–H Carboxylation of Unactivated Arenes

Ashot Gevorgyan,*[a] Kathrin H. Hopmann,[b] and Annette Bayer*[a]

Supplementary Information
Formal C-H Carboxylation of Unactivated Arenes

Ashot Gevorgyan,*[,a] Kathrin H. Hopmann,[b] Annette Bayer,*[,a]

[a] Department of Chemistry, UiT The Arctic University of Norway, 9037 Tromsø, Norway.
[b] Hylleraas Centre for Quantum Molecular Sciences, Department of Chemistry, UiT The Arctic University of Norway, 9037 Tromsø, Norway.
E-mail: ashot.gevorgyan@uit.no; annette.bayer@uit.no

Table of content:

General considerations 2
Optimization of reaction. 2
Table S1. Screening of catalysts 4
Table S2. Screening of duration and temperature 5
Table S3. Screening of bases 6
Table S4. Screening of solvents 7
Table S5. Optimization of formal C-H carboxylation 8
Starting materials used in the study 9
Control experiments on stepwise carboxylation of 1,3-dimethoxybenzene 10
Setup of the reaction 11
General procedures 15
Characterization of products 17
Copies of spectra 29

General considerations

Commercially available starting materials, reagents, catalysts and anhydrous and degassed solvents were used without further purification. Flash column chromatography was performed with Merck silica gel 60 (230-400 mesh). The solvents for column chromatography were distilled before use (in case of technical solvents). Thin layer chromatography was carried out using Merck TLC Silica gel 60 F_{254} and visualized by short-wavelength ultraviolet light or by treatment with potassium permanganate ($KMnO_4$) stain. ^{1}H, ^{13}C, ^{31}P and ^{19}F NMR spectra were recorded on a Bruker Avance 400 MHz at 20°C. All ^{1}H NMR spectra are reported in parts per million (ppm) downfield of TMS and were measured relative to the signals for $CHCl_3$ (7.26 ppm), methanol (4.87 ppm, 3.31 ppm) and DMSO (2.50 ppm). All ^{13}C NMR spectra were reported in ppm relative to residual $CDCl_3$ (77.20 ppm), methanol (49.1 ppm) or DMSO (39.70 ppm) and were obtained with ^{1}H decoupling. Coupling constants, *J*, are reported in Hertz (Hz). High-resolution mass spectra (HRMS) were recorded from methanol solutions on an LTQ Orbitrap XL (Thermo Scientific) either in negative or in positive electrospray ionization (ESI) mode. Melting points were measured using Stuart SMP50 automatic melting point detector.

Diethyl carbonate, ethylene carbonate, propylene carbonate, ethyl formate, 2MeTHF, ethanol and methanol were bought as anhydrous solvents equipped with a septa. Other solvents were reagent grade; they were degassed and kept over activated molecular sieves (4 Å) at least for a week before use.

Warning! Most of the reactions were performed in specialized glassware under pressure. The glassware should always be examined for damages before any manipulation. All laboratory safety procedures must be followed strictly and the work with pressure tubes must be conducted behind a shield.

Optimization of reaction.

General experimental procedure for Cu-catalyzed carboxylation of phenylboronic acid pinacol ester. For general setup, see Figure S1-S4.

Inside of glove box 45 mL pressure tube was charged with phenylboronic acid pinacol ester (0.735 mmol, 150 mg), base (0-3 equiv.) and corresponding dry solvent (2 mL). This was followed by addition of previously prepared solution of the catalyst (the mixture of transition metal (0-5 mol%), ligand (0-6 mol%) and NaO*t*Bu (0-6 mol%) in appropriate dry solvent (2 mL) was stirred at 20°C for 30 min). The pressure tube was closed with the cap and removed from the glove box. Afterwards CO_2 (120 mL) was added *via* a syringe, which was followed by stirring of reaction mixture at 60-120°C for 6-28h. Next, the reaction mixture was diluted with 15 mL Et_2O and transferred into 250 mL separating funnel. The resulting mixture was extracted with 15 mL saturated $NaHCO_3$ solution (3 times). The resulting basic solution was washed with 10 mL Et_2O (once), acidified (25-30 mL 6M HCl) and extracted with 15 mL Et_2O (3 times). The resulting solution of Et_2O was distilled to dryness to give corresponding acid.

In cases of PC, 1,2-BC, EC, PEG400 and GVL the basic solution was washed with either DCM or Et_2O (15 mL, 3 times), and the final Et_2O solution was washed with 10 mL distilled water (3 times) before evaporation.

In case of water, the reaction was performed outside of glove box using Ar flow for the inert atmosphere.

Other renewable solvents like 2MeTHF, diethoxymethane or dimethoxymethane can replace Et_2O without any noticeable difference (the difference was in the range ±3%). Similarly, saturated solution of $NaHCO_3$ can be replaced by 2M solution of KOH.

General experimental procedure for formal C-H carboxylation of 1,3-dimethoxybenzene. For general setup, see Figure S1-S4.

Inside of glove box 45 mL pressure tube was charged with 1,3-dimethoxybenzene (2.170 mmol), corresponding dry solvent-1 (3-4 mL), Ir-catalyst (0.25-1 mol%), ligand (0.5-2 mol%) and B_2pin_2 (0.6 equiv.). The pressure tube was closed with suitable cap, removed from the glove box and stirred at 80°C for 24h. Next, the pressure tube was transferred into the glove box where at 20°C to the reaction mixture was added CsF (3 equiv.) and previously prepared solution of Cu-catalyst (the mixture of CuI (5 mol%), IPrHCl (6 mol%) and NaO*t*Bu (6 mol%) in appropriate dry solvent-2 (3-6 mL) was stirred at 20°C for 30 min). The pressure tube was closed with the cap and removed from the glove box. Afterwards CO_2 (120 mL) was added *via* a syringe, which was followed by stirring of reaction mixture at 120°C for 24h. Next, the reaction mixture was diluted with 30 mL Et_2O and transferred into 500 mL separating funnel. The resulting mixture was extracted with 30 mL saturated $NaHCO_3$ solution (3 times). The resulting basic solution was washed with 15 mL Et_2O (once), acidified (50-55 mL 6M HCl) and extracted with 30 mL Et_2O (3 times). The resulting solution of Et_2O was distilled to dryness to give corresponding acid.

In case of GVL the basic solution was washed with either DCM or Et_2O (15 mL, 3 times), and the final Et_2O solution was washed with 10 mL distilled water (3 times) before evaporation.

Other renewable solvents like 2MeTHF, diethoxymethane or dimethoxymethane can replace Et_2O without any noticeable difference (the difference was in the range ±3%). Similarly, saturated solution of $NaHCO_3$ can be replaced by 2M solution of KOH.

Table S1. Screening of catalysts

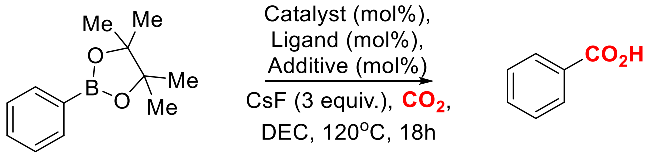

b1 p1

Entry	Catalyst (mol%)	Ligand (mol%)	Additive (mol%)	Yield of p1 %[a,b]
**1**	**CuI (5)**	**IPrHCl (6)**[c]	**NaO*t*Bu (6)**	**86**
2	CuBr (5)	IPrHCl (6)	NaO*t*Bu (6)	77
3	CuCl (5)	IPrHCl (6)	NaO*t*Bu (6)	70
4	$Cu(OAc)_2$ (5)	IPrHCl (6)	NaO*t*Bu (6)	62
**5**	**Ag_2CO_3 (5)**	**IPrHCl (6)**	**NaO*t*Bu (6)**	**71**
6	CuI (5)	I*t*BuHBF_4 (6)[d]	NaO*t*Bu (6)	70
7	CuI (5)	IAdHCl (6)[e]	NaO*t*Bu (6)	52
8	CuI (5)	IMesHCl (6)[f]	NaO*t*Bu (6)	47
9	CuI (5)	1,10-phen (6)[g]	NaO*t*Bu (6)	38
10	CuI (3)	IPrHCl (4)	NaO*t*Bu (4)	78%
11	-	-	-	0

[a] Reaction conditions: **b1** (0.735 mmol), DEC (2 mL), CsF (3 equiv.), transition metal (0-5 mol%), ligand (0-6 mol%), NaO*t*Bu (0-6 mol%), DEC (2 mL), CO_2 (120 mL), 120°C, 18h. [b] Isolated yields. [c] IPrHCl = 1,3-bis(2,6-diisopropylphenyl)imidazolium chloride. [d] I*t*BuHBF_4 = 1,3-di-*tert*-butylimidazolium tetrafluoroborate. [e] IAdHCl = 1,3-bis(1-adamantyl)imidazolium chloride. [f] IMesHCl = 1,3-bis(2,4,6-trimethylphenyl)imidazolium chloride. [g] 1,10-phen = 1,10-phenanthroline.

Table S2. Screening of duration and temperature

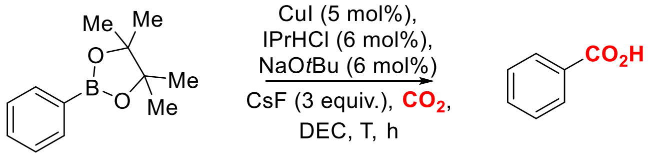

Entry	T, ºC	h	Yield of p1 %[a,b]
**1**	**120**	**18**	**86**
2	80	18	32
3	120	6	37
4	120	24	86
5	120	28	85

[a] Reaction conditions: **b1** (0.735 mmol), DEC (2 mL), CsF (3 equiv.), CuI (5 mol%), IPrHCl (6 mol%), NaO*t*Bu (6 mol%), DEC (2 mL), CO_2 (120 mL), 80-120ºC, 6-28h. [b] Isolated yields.

Table S3. Screening of bases

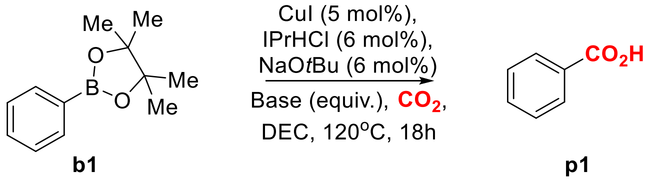

Entry	Base (equiv.)	Yield of p1 %[a,b]
**1**	**CsF (3)**	**86**
2	KF (3)	0
**3**	**Cs_2CO_3 (3)**	**84**
4	K_2CO_3 (3)	16
5	Rb_2CO_3 (3)	25
6	CsOAc (3)	0
7	KO*t*Bu (3)[c]	64
8	K_3PO_4 (3)	17
9	NaF (3)	0
10	CsF (2)	71
11	-	0

[a] Reaction conditions: **b1** (0.735 mmol), DEC (2 mL), base (0-3 equiv.), CuI (5 mol%), IPrHCl (6 mol%), NaO*t*Bu (6 mol%), DEC (2 mL), CO_2 (120 mL), 120°C, 18h. [b] Isolated yields. [c] Before addition of CO_2 the reaction mixture, containing KO*t*Bu, was mixed at 20°C for 30 min.

Table S4. Screening of solvents

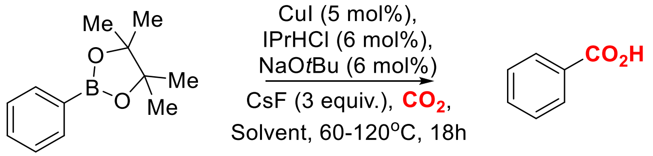

b1 **p1**

Entry	Solvent	T, °C	Yield of p1 %[a,b]
**1**	**DEC[c]**	**120**	**86**
**2**	**DMC[c]**	**120**	**89**
3	PC[c]	120	57
4	1,2-BC[c]	120	69
5	EC[c]	120	17
6	MeOH[c]	120	6
7	HCO_2Et[c]	60	0
8	HCO_2*n*Bu[c]	100	25
9	HCO_2Bn[c]	120	20
**10**	**Methylal[c]**	**120**	**81**
11	EtOH	120	0
12	Glycerol	120	0
13	H_2O	120	0
14	PEG400[d]	120	16
15	2MeTHF[e]	120	71
16	GVL[f]	120	65
17	Limonene	120	33
18	EtOAc	120	70
19	THF	120	76
20	Dioxane	120	76
21	Toluene	120	18
22	DMF	120	64

[a] Reaction conditions: **b1** (0.735 mmol), Solvent (2 mL), CsF (3 equiv.), CuI (5 mol%), IPrHCl (6 mol%), NaO*t*Bu (6 mol%), Solvent (2 mL), CO_2 (120 mL), 60-120°C, 18h. [b] Isolated yields. [c] For full names and structure see Scheme S1. [d] PEG400 = polyethylene glycol 400. [e] 2MeTHF = 2-methyltetrahydrofuran. [f] GVL = *gamma*-valerolactone.

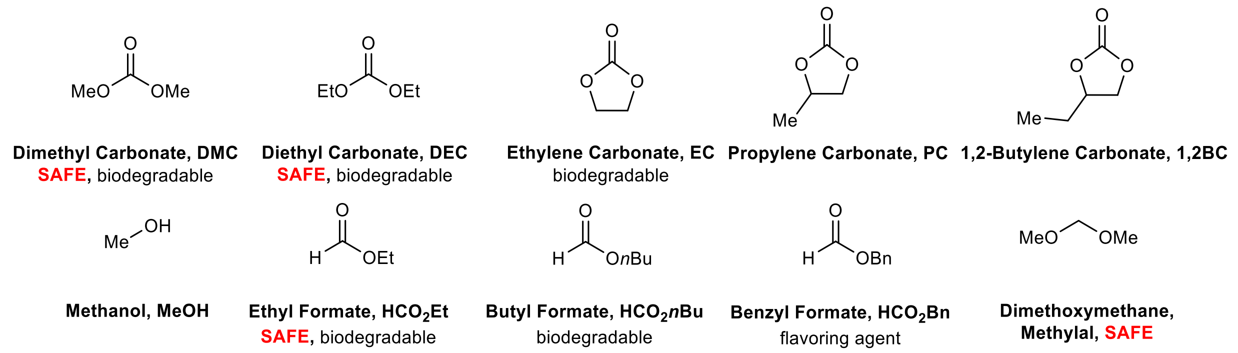

Scheme S1. Structure of CO_2-derived solvents with suitable abbreviations.

Table S5. Optimization of formal C-H carboxylation

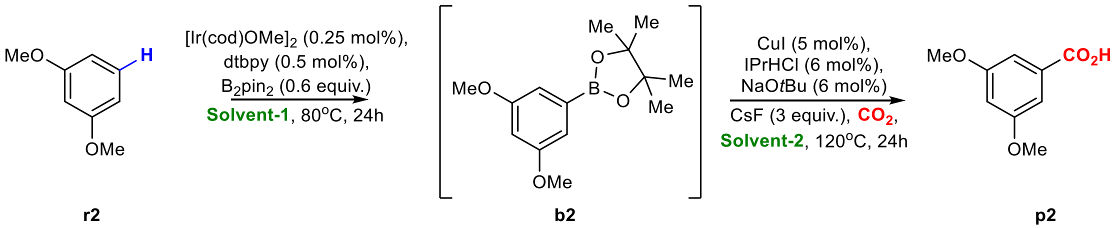

Entry	Solvent-1 (mL)	Solvent-2 (mL)	Yield of p2 %[a,b]
1	DEC (4)[c]	DEC (4)	0[d]
2	Methylal (4)	Methylal (4)	69[d]
**3**	**Methylal (4)**	**Methylal (4)**	**73**
4	Methylal (4)	DEC (4)	70
5	Methylal (4)	DEC (4)	72[e]
6	Methylal (3)	DEC (6)	68
7	Methylal (4)	DMC (4)	49
8	THF (4)	THF (4)	70
9	Hexane (4)	Hexane (4)	0
10	2MeTHF (4)[f]	2MeTHF (4)	61
11	GVL (3)[g]	GVL (3)	31
12	EtOAc (4)	EtOAc (4)	23
13	Methylal (4)	Methylal (4)	0[h]
14	Methylal (4)	Methylal (4)	0[i]
15	Methylal (4)	Methylal (4)	0[j]
16	Methylal (4)	Methylal (4)	47[k]
17	Methylal (4)	Methylal (4)	74[l]
18	Methylal (4)	Methylal (4)	67[m]

[a] Reaction conditions: 1) **r2** (2.170 mmol), Solvent-1 (3-4 mL), $[Ir(cod)OMe]_2$ (0.25 mol%), dtbpy (0.5 mol%), B_2pin_2 (0.6 equiv.), 80°C, 24h. 2) CuI (5 mol%), IPrHCl (6 mol%), NaO*t*Bu (6 mol%), Solvent-2 (3-6 mL), CsF (3 equiv.), CO_2 (120 mL), 120°C, 24h. [b] Isolated yields. [c] For full names and structure see Scheme S1. [d] The reaction was performed for 18h. [e] C-H borylation step was performed for 36h. [f] 2MeTHF = 2-methyltetrahydrofuran. [g] GVL = *gamma*-valerolactone. [h] The reaction was performed using $[Ir(cod)Cl]_2$. [i] The reaction was performed using pentamethylcyclopentadienyliridium(III) chloride dimer. [j] The reaction was performed using 1,10-phenanthroline as ligand. [k] The reaction was performed using 3,4,7,8-tetramethyl-1,10-phenanthroline as ligand. [l] The catalyst loading was $[Ir(cod)OMe]_2$ (1 mol%), dtbpy (2 mol%). [m] C-H borylation step was performed at 100°C.

Starting materials used in the study

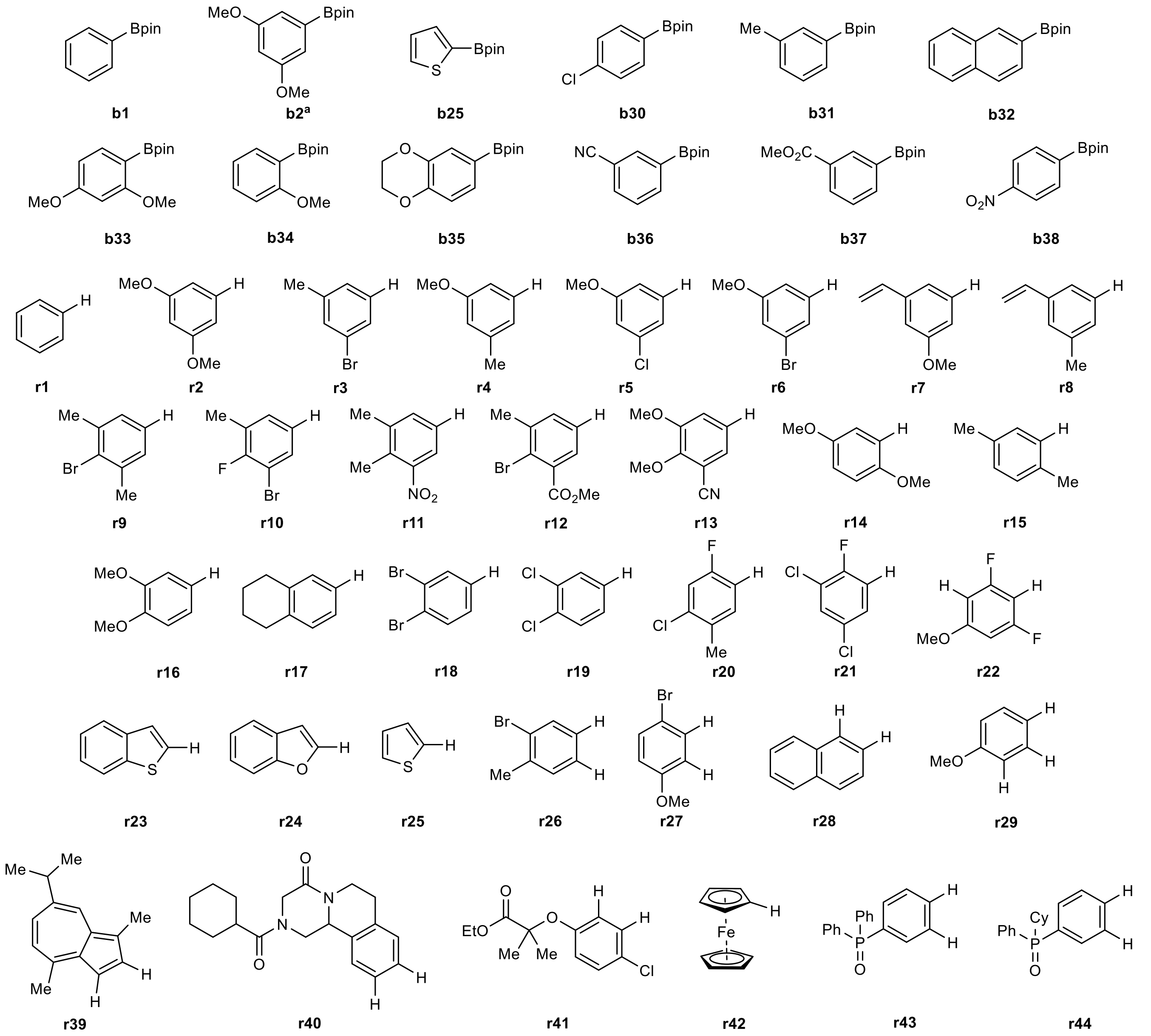

Scheme S2. List of starting materials used in the work. [a] This compound was synthesized, while others are commercially available.

Control experiments on stepwise carboxylation of 1,3-dimethoxybenzene

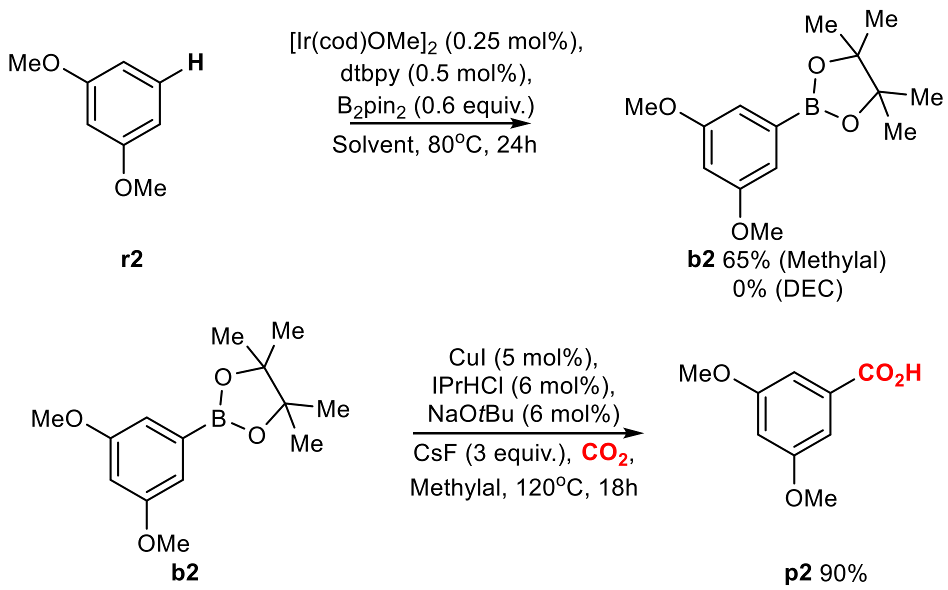

Scheme S3. Control experiments on stepwise carboxylation of 1,3-dimethoxybenzene.

Setup of the reaction

Figure S1. 45mL pressure tube with suitable stabilizer and septa.

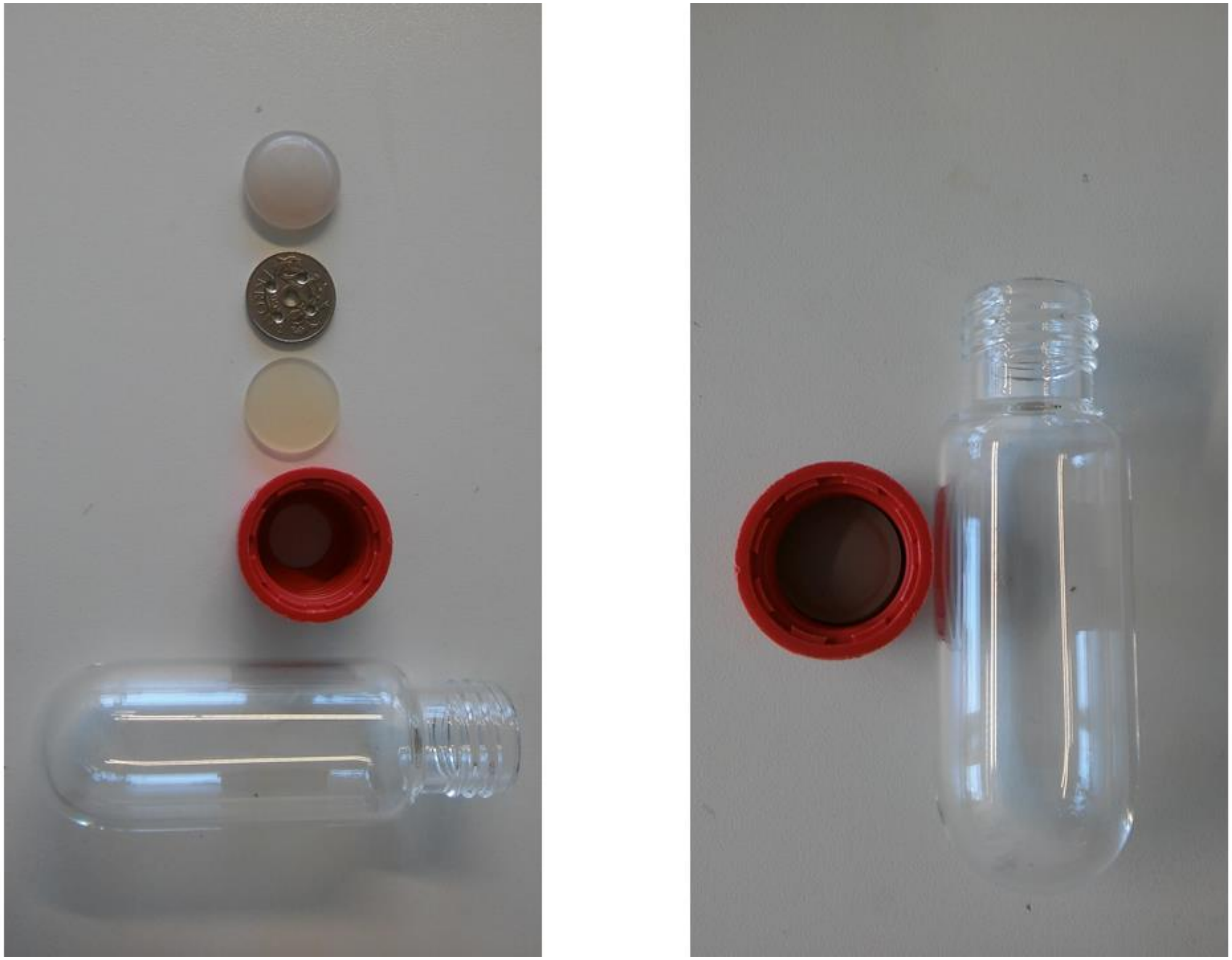

Figure S2. 45mL pressure tube with suitable stabilizer and septa.

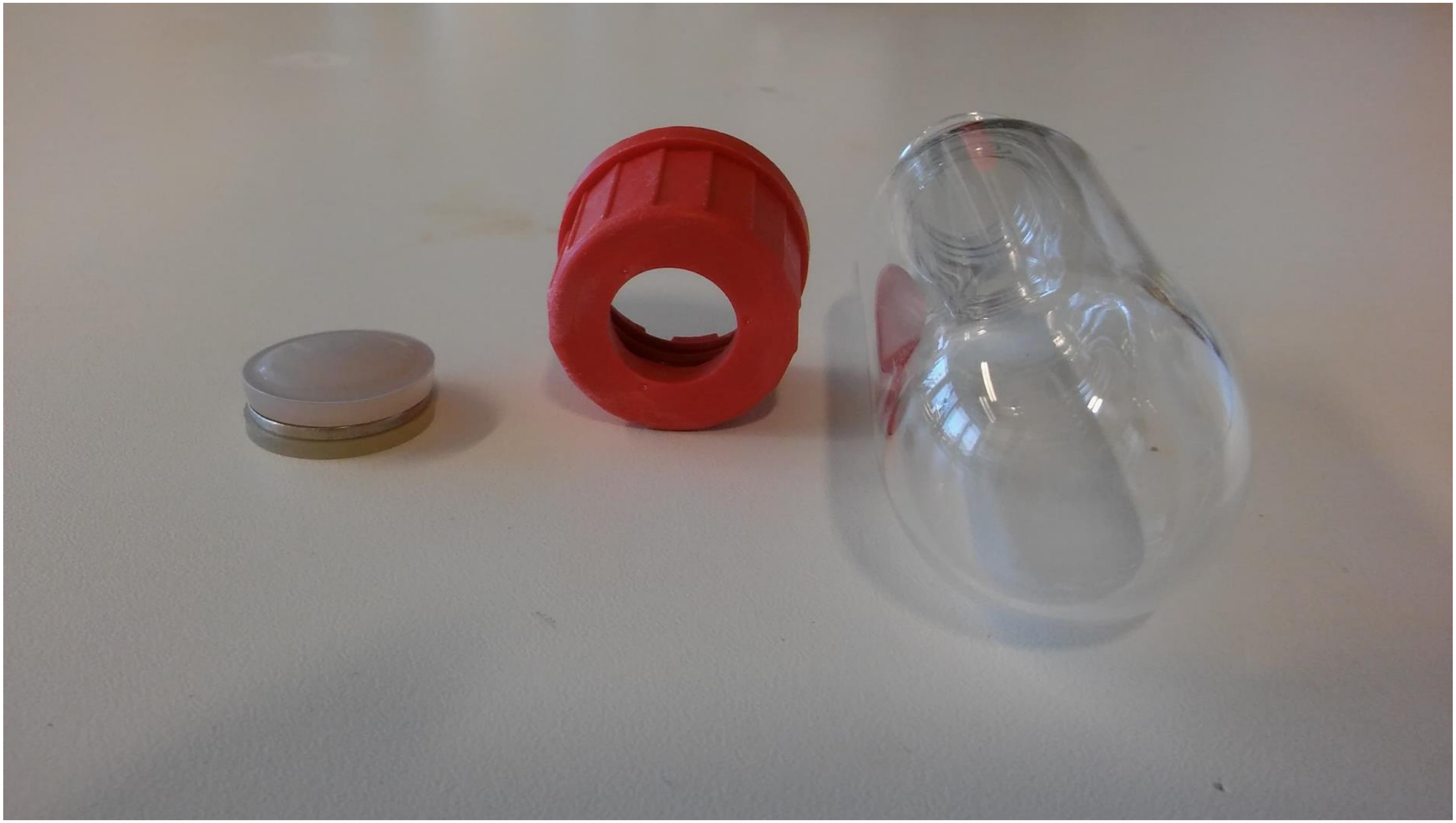

Figure S3. Syringe with CO_2 ready for injection.

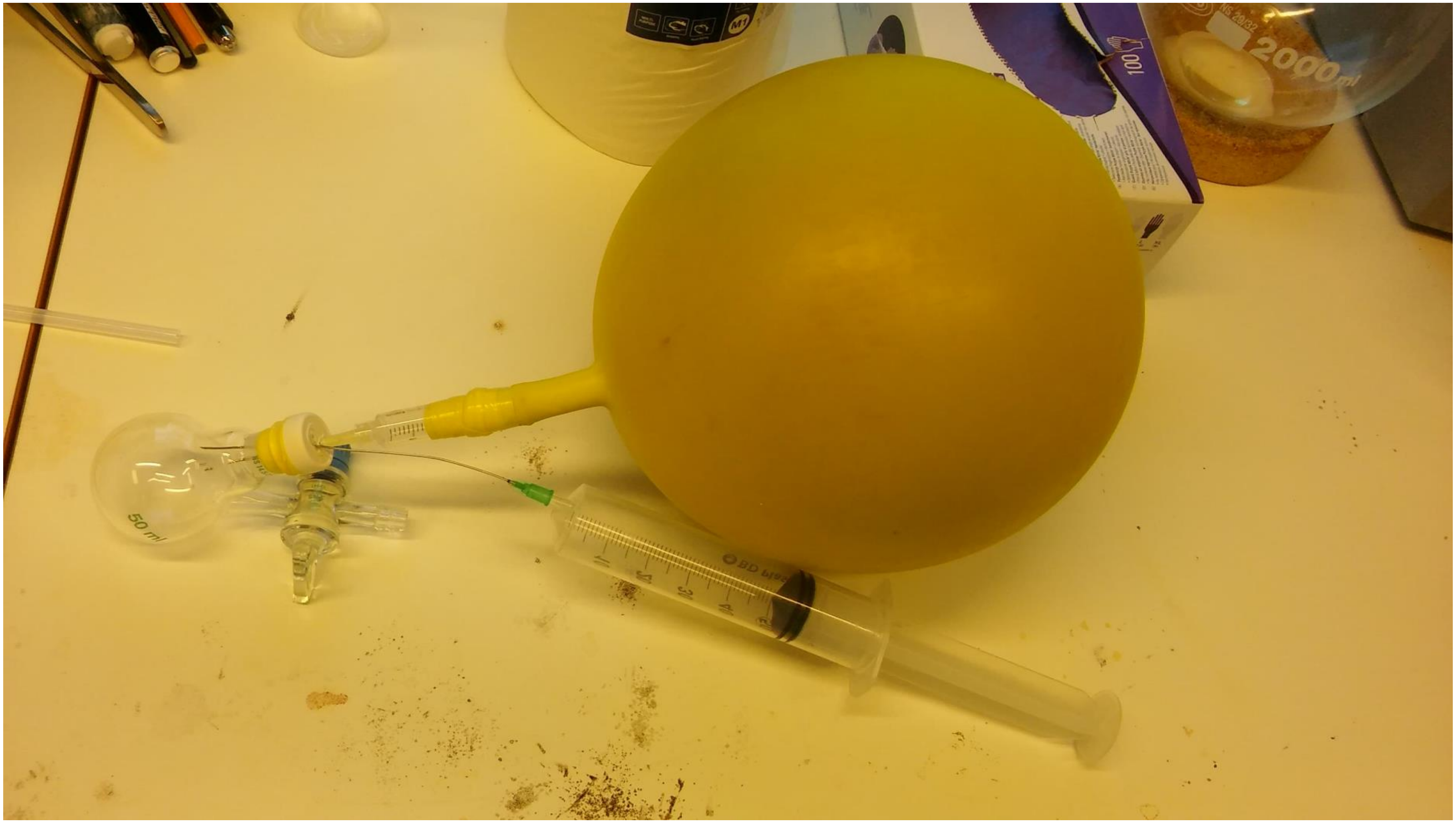

Figure S4. First injection of CO_2.

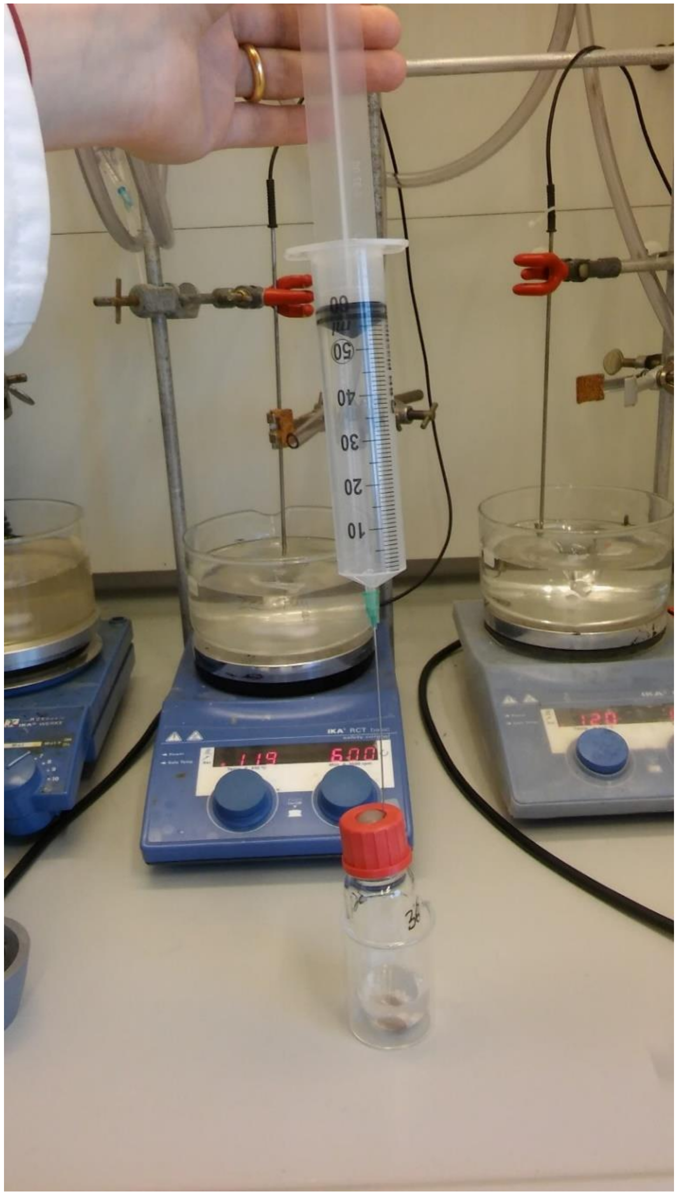

General procedures

General experimental procedure for formal C-H carboxylation of arenes. For general setup, see Figure S1-S4.

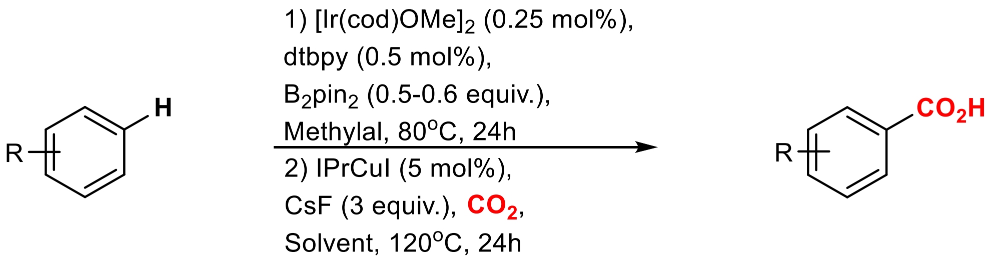

Inside of glove box 45 mL pressure tube was charged with appropriate arene (2 mmol), dry methylal (4 mL), $[Ir(cod)OMe]_2$ (0.25 mol%), dtbpy (0.5 mol%) and B_2pin_2 (0.5 equiv. for benzene **r1** and arenes **r14-22**, **r25-29**, **r39-41**, **r43, r44**; 0.6 equiv. for arenes **r2-13**, heterocycles **r23**, **r24** and ferrocene **r42**). The pressure tube was closed with suitable cap, removed from the glove box and stirred at 80°C for 24h. Next, the pressure tube was transferred into the glove box where at 20°C to the reaction mixture was added CsF (3 equiv.) and previously prepared solution of Cu-catalyst (the mixture of CuI (5 mol%), IPrHCl (6 mol%) and NaO*t*Bu (6 mol%) in appropriate dry solvent (4 mL) was stirred at 20°C for 30 min). The pressure tube was closed with the cap and removed from the glove box. Afterwards CO_2 (120 mL) was added *via* a syringe, which was followed by stirring of reaction mixture at 120°C for 24h. Next, the reaction mixture was diluted with 30 mL Et_2O and transferred into 500 mL separating funnel. The resulting mixture was extracted with 30 mL saturated $NaHCO_3$ solution (3 times). The resulting basic solution was washed with 15 mL Et_2O (3 times), acidified (50-55 mL 6M HCl) and extracted with 30 mL Et_2O (3 times). The resulting solution of Et_2O was distilled to dryness to give corresponding acid.
Other renewable solvents like 2MeTHF, diethoxymethane or dimethoxymethane can replace Et_2O without any noticeable difference (the difference was in the range ±3%). Similarly, saturated solution of $NaHCO_3$ can be replaced by 2M solution of KOH.

General experimental procedure for Cu-catalyzed carboxylation of organoboronates. For general setup, see Figure S1-S4.

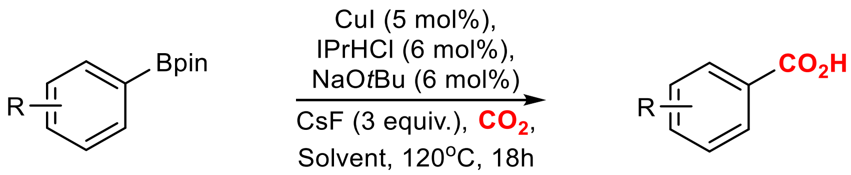

Inside of glove box 45 mL pressure tube was charged with appropriate organoboronate (0.8 mmol), CsF (3 equiv.) and corresponding dry solvent (3 mL). This was followed by addition of previously prepared solution of Cu-catalyst (the mixture of CuI (5 mol%), IPrHCl (6 mol%) and NaO*t*Bu (6 mol%) in appropriate dry solvent (3 mL) was stirred at 20°C for 30 min). The pressure tube was closed with the cap and

removed from the glove box. Afterwards CO_2 (120 mL) was added *via* a syringe, which was followed by stirring of reaction mixture at 120^oC for 18h. Next, the reaction mixture was diluted with 30 mL Et_2O and transferred into 500 mL separating funnel. The resulting mixture was extracted with 30 mL saturated $NaHCO_3$ solution (3 times). The resulting basic solution was washed with 15 mL Et_2O (once), acidified (50-55 mL 6M HCl) and extracted with 30 mL Et_2O (3 times). The resulting solution of Et_2O was distilled to dryness to give corresponding acid. Other renewable solvents like 2MeTHF, diethoxymethane or dimethoxymethane can replace Et_2O without any noticeable difference (the difference was in the range ±3%). Similarly, saturated solution of $NaHCO_3$ can be replaced by 2M solution of KOH.

General experimental procedure for Ir-catalyzed C-H borylation of 1,3-dimethoxybenzene in methylal.

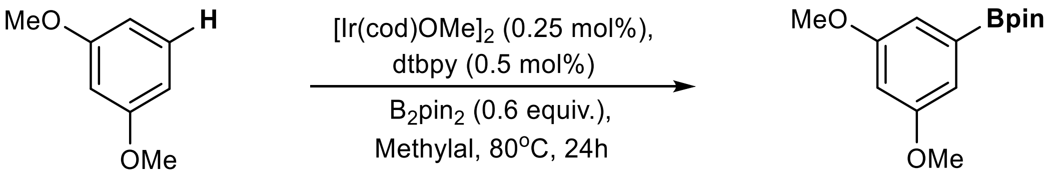

Inside of glove box 45 mL pressure tube was charged with 1,3-dimethoxybenzene (2.170 mmol), dry methylal (4 mL), $[Ir(cod)OMe]_2$ (0.25 mol%), dtbpy (0.5 mol%) and B_2pin_2 (0.6 equiv.). The pressure tube was closed with suitable cap, removed from the glove box and stirred at 80^oC for 24h. The resulting mixture was evaporated to dryness and purified using column chromatography.

Characterization of products

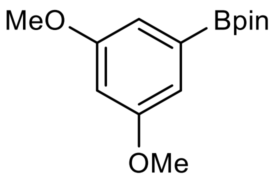

***2-(3,5-Dimethoxyphenyl)-4,4,5,5-tetramethyl-1,3,2-dioxaborolane*, b2.**[1] Starting from 2.170 mmol of corresponding arene the product was obtained as a white solid, m.p. = 92-94ºC, yield 65% (0.373 g, Methylal). **^{1}H NMR** (400 MHz, $CDCl_3$): δ = 1.35 (s, 12H, Bpin), 3.82 (s, 6H, 2 x OMe), 6.58 (t, *J* = 2.4 Hz, 1H, Ar), 6.96 (d, *J* = 2.5 Hz, 2H). **^{13}C NMR** (101 MHz, $CDCl_3$): δ = 25.0, 55.5, 84.0, 104.6, 111.8, 160.6.

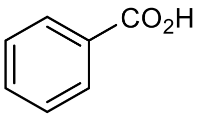

***Benzoic acid*, p1.**[2] Starting from 2.560 mmol of corresponding arene the product was obtained as a white solid, m.p. = 121-125ºC, yield 88% (0.274 g, Methylal). Starting from 0.980 mmol of corresponding boronic acid pinacol ester the product was obtained as a white solid, m.p. = 121-125ºC, yield 86% (0.103 g, DEC), 89% (0.106 g, DMC), 81% (0.097 g, Methylal). **^{1}H NMR** (400 MHz, $CDCl_3$): δ = 7.47-7.51 (m, 2H, Ar), 7.61-7.65 (m, 1H, Ar), 8.13-8.16 (m, 2H, Ar), 12.41 (br s, 1H, CO_2H). **^{13}C NMR** (101 MHz, $CDCl_3$): δ = 128.7, 129.5, 130.4, 134.0, 172.6. **HRMS-EI** (m/z) $[M-H]^-$ calcd. for $C_7H_5O_2$ 121.0295 found 121.0301.

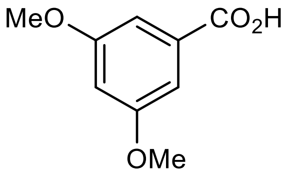

***3,5-Dimethoxybenzoic acid*, p2.**[3] Starting from 2.170 mmol of corresponding arene the product was obtained as a white solid, m.p. = 178-180ºC, yield 73% (0.287 g, Methylal), 71% (0.282 g, Methylal/DEC), 49% (0.194 g, Methylal/DMC). Starting from 0.757 mmol of corresponding boronic acid pinacol ester the product was obtained as a white solid, m.p. = 178-180ºC, yield 94% (0.129 g, DEC), 88% (0.122 g, DMC), 90% (0.124 g, Methylal). **^{1}H NMR** (400 MHz, MeOH-d4): δ = 3.81 (s, 6H, 2 x OMe), 6.69 (t, *J* = 2.4 Hz, 1H, Ar), 7.15 (d, *J* = 2.4 Hz, 2H, Ar). **^{13}C NMR** (101 MHz, MeOH-d4): δ = 56.1, 106.2, 108.4, 162.4, 169.6. **HRMS-EI** (m/z) $[M-H]^-$ calcd. for $C_9H_9O_4$ 181.0506 found 181.0510.

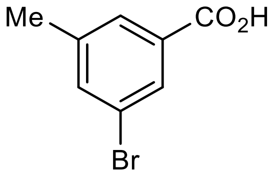

***3-Bromo-5-methylbenzoic acid*, p3.**[4] Starting from 1.750 mmol of corresponding arene the product was obtained as a white solid, m.p. = 151-153ºC, yield 76% (0.285 g, Methylal). **^{1}H NMR** (400 MHz, $CDCl_3$): δ = 2.40 (s, 3H, Me), 7.55-7.56 (m, 1H, Ar), 7.83-7.84 (m, 1H, Ar), 8.03-8.04 (m, 1H, Ar). **^{13}C NMR** (101 MHz, $CDCl_3$): δ = 21.2, 122.5, 129.6, 130.4, 131.2, 137.5, 140.6, 170.9. **HRMS-EI** (m/z) $[M-H]^-$ calcd. for $C_8H_6BrO_2$ 212.9557 found 212.9559.

---

[1] G. Wang, L. Xu and P. Li, *J. Am. Chem. Soc.,* 2015, **137**, 8058-8061.
[2] J. Takaya, S. Tadami, K. Ukai and N. Iwasawa, *Org. Lett.,* 2008, **10**, 2697-2700.
[3] H. Yu, S. Ru, G. Dai, Y. Zhai, H. Lin, S. Han and Y. Wei, *Angew. Chem. Int. Ed.,* 2017, **56**, 3867-3871.
[4] T. H. Fisher, S. M. Dershem and M. L. Prewitt, *J. Org. Chem.,* 1990, **55**, 1040-1043.

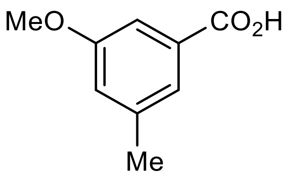

***3-Methoxy-5-methylbenzoic acid,* p4.**[5] Starting from 1.640 mmol of corresponding arene the product was obtained as a white solid, m.p. = 128-131°C, yield 69% (0.188 g, Methylal). **^{1}H NMR** (400 MHz, $CDCl_3$): δ = 2.38 (s, 3H, Me), 3.84 (s, 3H, OMe), 6.96-6.97 (m, 1H, Ar), 7.42-7.43 (m, 1H, Ar), 7.53-7.54 (m, 1H, Ar), 9.14 (br s, 1H, CO_2H). **^{13}C NMR** (101 MHz, $CDCl_3$): δ = 21.5, 55.6, 111.7, 121.3, 123.6, 130.6, 139.9, 159.7, 172.2. **HRMS-EI** (m/z) $[M-H]^-$ calcd. for $C_9H_9O_3$ 165.0557 found 165.0562.

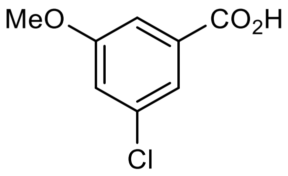

***3-Chloro-5-methoxybenzoic acid,* p5.**[6] Starting from 2.100 mmol of corresponding arene the product was obtained as a white solid, m.p. = 172-176°C, yield 89% (0.348 g, Methylal). **^{1}H NMR** (400 MHz, MeOH-d4): δ = 3.84 (s, 3H, OMe), 7.15 (t, *J* = 2.2 Hz, 1H, Ar), 7.45 (dd, *J* = 2.5, 1.3 Hz, 1H, Ar), 7.54 (t, *J* = 1.6 Hz, 1H, Ar). **^{13}C NMR** (101 MHz, MeOH-d4): δ = 56.4, 114.6, 119.8, 122.9, 136.2, 162.0, 168.2. **HRMS-EI** (m/z) $[M-H]^-$ calcd. for $C_8H_6ClO_3$ 185.0011 found 185.0016.

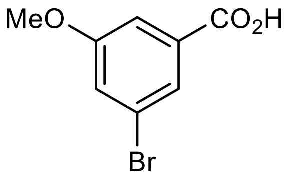

***3-Bromo-5-methoxybenzoic acid,* p6.**[7] Starting from 1.600 mmol of corresponding arene the product was obtained as a white solid, m.p. = 184-186°C, yield 83% (0.306 g, Methylal). **^{1}H NMR** (400 MHz, MeOH-d4): δ = 3.84 (s, 3H, OMe), 7.30-7.31 (m, 1H, Ar), 7.49-7.50 (m, 1H, Ar), 7.69-7.70 (m, 1H, Ar). **^{13}C NMR** (101 MHz, MeOH-d4): δ = 56.4, 115.0, 122.8, 123.7, 125.9, 162.1, 168.1. **HRMS-EI** (m/z) $[M-H]^-$ calcd. for $C_8H_6BrO_3$ 228.9506 found 228.9508.

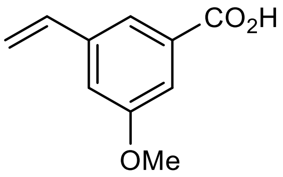

***3-Methoxy-5-vinylbenzoic acid,* p7.** Starting from 2.240 mmol of corresponding arene the product was obtained as a colourless viscous oil, yield 46% (0.182 g, Methylal). **^{1}H NMR** (400 MHz, MeOH-d4): δ = 3.81 (s, 3H, OMe), 6.46 (d, *J* = 16.0 Hz, 1H, olefin), 6.96 (ddd, *J* = 8.3, 2.6, 1.0 Hz, 1H, olefin), 7.11-7.16 (m, 2H, Ar), 7.30 (t, *J* = 7.9 Hz, 1H, Ar), 7.63 (d, *J* = 16.0 Hz, 1H, olefin). **^{13}C NMR** (101 MHz, MeOH-d4): δ = 55.9, 114.1, 117.4, 119.7, 121.8, 131.1, 137.3, 146.4, 161.6, 170.4. **HRMS-EI** (m/z) $[M-H]^-$ calcd. for $C_{10}H_9O_3$ 177.0557 found 177.0562.

---

[5] O. de Frutos, C. Atienza and A. M. Echavarren, *Eur. J. Org. Chem.,* 2001, 163-171.
[6] H. Tarazia, M. I. El-Gamala and C.-H. Oh, *Bioorg. Med. Chem.,* 2019, **27**, 655-663.
[7] R. Miyaji, Y. Wada, A. Matsumoto, K. Asano and S. Matsubara, *Beilstein J. Org. Chem.,* 2017, **13**, 1518-1523.

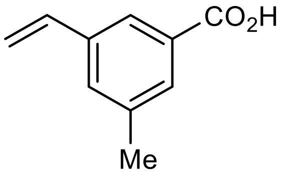

***3-Methyl-5-vinylbenzoic acid*, p8.** Starting from 2.120 mmol of corresponding arene the product was obtained as a white solid, m.p. = 101-104ºC, yield 50% (0.170 g, Methylal). **^{1}H NMR** (400 MHz, MeOH-d4): δ = 2.35 (s, 3H, Me), 6.45 (d, *J* = 16.0 Hz, 1H, olefin), 7.21 (d, *J* = 7.6 Hz, 1H, olefin), 7.27 (t, *J* = 7.5 Hz, 1H, Ar), 7.35-7.39 (m, 2H, Ar), 7.63 (d, *J* = 16.0 Hz, 1H, olefin). **^{13}C NMR** (101 MHz, MeOH-d4): δ = 25.1, 119.2, 126.4, 129.9, 130.0, 132.3, 135.9, 140.0, 146.6, 170.5. **HRMS-EI** (m/z) $[M-H]^-$ calcd. for $C_{10}H_9O_2$ 161.0608 found 161.0613.

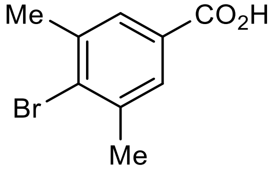

***4-Bromo-3,5-dimethylbenzoic acid*, p9.**[8] Starting from 1.620 mmol of corresponding arene the product was obtained as a white solid, m.p. = above 200ºC, yield 62% (0.230 g, Methylal), 52% (0.192 g, Methylal/DEC), 36% (0.134 g, Methylal/DMC). **^{1}H NMR** (400 MHz, MeOH-d4): δ = 2.42 (s, 6H, 2 x Me), 7.71 (s, 2H, Ar). **^{13}C NMR** (101 MHz, MeOH-d4): δ = 24.1, 128.5, 130.0, 130.3, 130.6, 133.6, 139.9, 169.4. **HRMS-EI** (m/z) $[M-H]^-$ calcd. for $C_9H_8BrO_2$ 226.9713 found 226.9721.

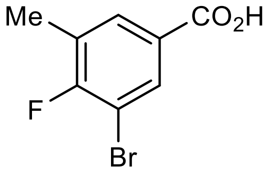

***3-Bromo-4-fluoro-5-methylbenzoic acid*, p10.** Starting from 1.590 mmol of corresponding arene the product was obtained as a white solid, m.p. = 173-176ºC, yield 72% (0.265 g, Methylal). **^{1}H NMR** (400 MHz, MeOH-d4): δ = 2.33 (d, *J* = 2.4 Hz, 3H, Me), 7.84 (ddd, *J* = 6.8, 2.1, 1.0 Hz, 1H, Ar), 8.01 (dd, *J* = 6.4, 2.1 Hz, 1H, Ar). **^{13}C NMR** (101 MHz, MeOH-d4): δ = 14.9 (d, *J* = 3.3 Hz), 109.7 (d, *J* = 22.5 Hz), 128.1 (d, *J* = 19.0 Hz), 129.2, 133.6 (d, *J* = 5.7 Hz), 133.8 (d, *J* = 1.4 Hz), 161.7 (d, *J* = 250.9 Hz), 167.6. **HRMS-EI** (m/z) $[M-H]^-$ calcd. for $C_8H_5BrFO_2$ 230.9462 found 230.9465.

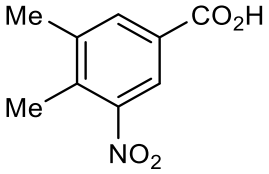

***3,4-Dimethyl-5-nitrobenzoic acid*, p11.**[9] Starting from 1.980 mmol of corresponding arene the product was obtained as a yellowish viscous oil, yield 16% (0.060 g, Methylal). **^{1}H NMR** (400 MHz, MeOH-d4): δ = 2.39 (s, 3H, Me), 2.42 (s, 3H, Me), 8.02 (d, *J* = 1.8 Hz, 1H, Ar), 8.13 (d, *J* = 1.7 Hz, 1H, Ar). **^{13}C NMR** (101 MHz, MeOH-d4): δ = Due to bad solubility it was not possible to measure. **HRMS-EI** (m/z) $[M-H]^-$ calcd. for $C_9H_8NO_4$ 194.0459 found 194.0462.

[8] Z. Chai, S. Wan, C. Zhong, T. Xu, M. Fang, J. Wang, Y. Xie, Y. Zhang, A. Mei, H. Han, Q. Peng, Q. Li and Z. Li, *ACS Appl. Mater. Interfaces,* 2016, **8**, 28652-28662.
[9] S. Yang and W. A. Denny, *Tetrahedron Lett.,* 2009, **50**, 3945-3947.

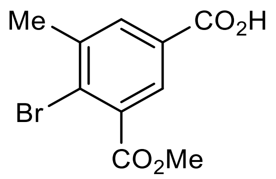

***4-Bromo-3-(methoxycarbonyl)-5-methylbenzoic acid*, p12.** Starting from 1.750 mmol of corresponding arene the product was obtained as a white solid, m.p. = 160-163°C, yield 77% (0.367 g, Methylal). **^{1}H NMR** (400 MHz, MeOH-d4): δ = 2.49 (s, 3H, Me), 3.93 (s, 3H, CO_2Me), 8.01-8.03 (m, 2H, Ar). **^{13}C NMR** (101 MHz, MeOH-d4): δ = 23.9, 53.3, 128.7, 129.9, 131.2, 134.7, 136.0, 141.7, 168.1, 168.5. **HRMS-EI** (m/z) $[M-H]^-$ calcd. for $C_{10}H_8BrO_4$ 270.9611 found 270.9612.

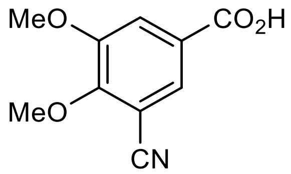

***3-Cyano-4,5-dimethoxybenzoic acid*, p13.** Starting from 1.840 mmol of corresponding arene the product was obtained as a white solid, m.p. = 185-188°C, yield 87% (0.331 g, Methylal). **^{1}H NMR** (400 MHz, MeOH-d4): δ = 3.95 (s, 3H, OMe), 4.07 (s, 3H, OMe), 7.78 (d, *J* = 1.9 Hz, 1H, Ar), 7.83 (d, *J* = 2.0 Hz, 1H, Ar). **^{13}C NMR** (101 MHz, MeOH-d4): δ = 56.9, 62.3, 107.5, 116.5, 119.2, 127.3, 128.4, 153.7, 156.3, 167.5. **HRMS-EI** (m/z) $[M-H]^-$ calcd. for $C_{10}H_8NO_4$ 206.0459 found 206.0461.

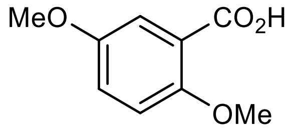

***2,5-Dimethoxybenzoic acid*, p14.**[10a] Starting from 2.170 mmol of corresponding arene the product was obtained as a white solid, m.p. = 76-78°C, yield 59% (0.233 g, Methylal). **^{1}H NMR** (400 MHz, MeOH-d4): δ = 3.76 (s, 3H, OMe), 3.86 (s, 3H, OMe), 7.01-7.10 (m, 2H, Ar), 7.36 (d, *J* = 3.1 Hz, 1H, Ar). **^{13}C NMR** (101 MHz, MeOH-d4): δ = 56.3, 57.2, 115.0, 117.4, 120.9, 154.7, 154.8, 169.1. **HRMS-EI** (m/z) $[M-H]^-$ calcd. for $C_9H_9O_4$ 181.0506 found 181.0508.

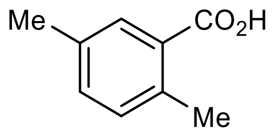

***2,5-Dimethylbenzoic acid*, p15.**[10b] Starting from 1.880 mmol of corresponding arene the product was obtained as a white solid, m.p. = 132-134°C, yield 26% (0.074 g, Methylal). **^{1}H NMR** (400 MHz, MeOH-d4): δ = 2.31 (s, 3H, Me), 2.51 (s, 3H, Me), 7.12 (d, *J* = 7.9 Hz, 1H, Ar), 7.21 (dd, *J* = 7.8, 2.0 Hz, 1H, Ar), 7.70-7.71 (m, 1H, Ar). **^{13}C NMR** (101 MHz, MeOH-d4): δ = 20.9, 21.5, 131.2, 132.2, 132.7, 133.8, 136.5, 138.1, 171.4. **HRMS-EI** (m/z) $[M-H]^-$ calcd. for $C_9H_9O_2$ 149.0608 found 149.0612.

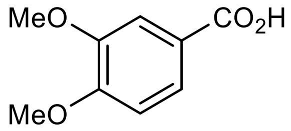

***3,4-Dimethoxybenzoic acid*, p16.**[11a] Starting from 2.170 mmol of corresponding arene the product was obtained as a white solid, m.p. = 179-182°C, yield 72% (0.286 g, Methylal). **^{1}H NMR** (400 MHz, MeOH-d4): δ = 3.87 (d, *J* = 9.6 Hz, 6H, 2 x OMe), 6.99 (d, *J* = 8.4 Hz, 1H, Ar), 7.55 (d, *J* = 2.0 Hz, 1H, Ar), 7.66 (dd, *J* = 8.4, 2.0 Hz, 1H, Ar).

---

[10] (a) A. Sarbajna, I. Dutta, P. Daw, S. Dinda, S. M. W. Rahaman, A. Sarkar and J. K. Bera, *ACS Catal.*, 2017, **7**, 2786-2790; (b) R. Shang, L. Ilies and E. Nakamura, *J. Am. Chem. Soc.*, 2016, **138**, 10132-10135.

^{13}**C NMR** (101 MHz, MeOH-d4): δ = 56.5, 56.5, 111.9, 113.7, 124.3, 125.2, 150.2, 154.8, 169.8. **HRMS-EI** (m/z) $[M-H]^-$ calcd. for $C_9H_9O_4$ 181.0506 found 181.0511.

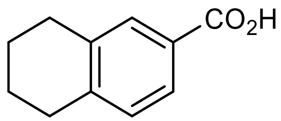

***5,6,7,8-Tetrahydronaphthalene-2-carboxylic acid*, p17.**[11b] Starting from 1.890 mmol of corresponding arene the product was obtained as a white solid, m.p. = 154-156°C, yield 42% (0.141 g, Methylal). ^{1}**H NMR** (400 MHz, MeOH-d4): δ = 1.77 (dq, *J* = 6.6, 3.0 Hz, 4H, 2xCH_2), 2.73-2.77 (m, 4H, 2xCH_2), 7.07 (d, *J* = 8.5 Hz, 1H, Ar), 7.65-7.68 (m, 2H, Ar). ^{13}**C NMR** (101 MHz, MeOH-d4): δ = 24.1, 24.2, 30.3, 30.6, 127.9, 129.0, 130.2, 131.6, 138.4, 144.0, 170.3. **HRMS-EI** (m/z) $[M-H]^-$ calcd. for $C_{11}H_{11}O_2$ 175.0765 found 175.0768.

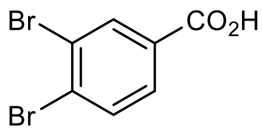

***3,4-Dibromobenzoic acid*, p18.** Starting from 1.700 mmol of corresponding arene the product was obtained as a white solid, m.p. = above 200°C, yield 65% (0.308 g, Methylal). ^{1}**H NMR** (400 MHz, MeOH-d4): δ = 7.80 (d, *J* = 8.3 Hz, 1H, Ar), 7.85 (dd, *J* = 8.3, 2.0 Hz, 1H, Ar), 8.25 (d, *J* = 2.0 Hz, 1H, Ar). ^{13}**C NMR** (101 MHz, MeOH-d4): δ = 125.8, 130.8, 130.9, 133.1, 135.2, 135.8, 167.6. **HRMS-EI** (m/z) $[M-H]^-$ calcd. for $C_7H_3Br_2O_2$ 278.8485 found 278.8486.

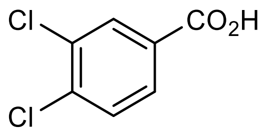

***3,4-Dichlorobenzoic acid*, p19.**[11c] Starting from 2.040 mmol of corresponding arene the product was obtained as a white solid, m.p. = above 200°C, yield 62% (0.240 g, Methylal). ^{1}**H NMR** (400 MHz, MeOH-d4): δ = 7.61 (d, *J* = 8.4 Hz, 1H, Ar), 7.89 (dd, *J* = 8.4, 2.0 Hz, 1H, Ar), 8.08 (d, *J* = 2.0 Hz, 1H, Ar). ^{13}**C NMR** (101 MHz, MeOH-d4): δ = 130.3, 131.9, 132.7, 133.8, 138.2, 167.5. **HRMS-EI** (m/z) $[M-H]^-$ calcd. for $C_7H_3Cl_2O_2$ 188.9516 found 188.9520.

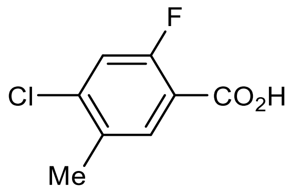

***4-Chloro-2-fluoro-5-methylbenzoic acid*, p20.**[12] Starting from 2.080 mmol of corresponding arene the product was obtained as a white solid, m.p. = 163-166°C, yield 61% (0.238 g, Methylal). ^{1}**H NMR** (400 MHz, MeOH-d4): δ = 2.33 (s, 3H, Me), 7.22 (d, *J* = 10.4 Hz, 1H, Ar), 7.81 (d, *J* = 7.8 Hz, 1H, Ar). ^{13}**C NMR** (101 MHz, MeOH-d4): δ = 19.2, 118.7 (d, *J* = 26.1 Hz), 133.4 (d, *J* = 4.1 Hz), 134.9 (d, *J* = 1.5 Hz), 140.5 (d, *J* = 10.3 Hz), 161.5 (d, *J* = 259.8 Hz), 166.5. **HRMS-EI** (m/z) $[M-H]^-$ calcd. for $C_8H_5ClFO_2$ 186.9968 found 186.9969.

---

[11] (a) Z.-z. Zhou, M. Liu, L. Lv and C.-J. Li, *Angew. Chem. Int. Ed.*, 2018, **57**, 2616-2620; (b) S. K. Bhunia, P. Das, S. Nandi and R. Jana, *Org. Lett.*, 2019, **21**, 4632-4637; (c) K.-J. Liu, Y.-L. Fu, L.-Y. Xie, C. Wu, W.-B. He, S. Peng, Z. Wang, W.-H. Bao, Z. Cao, X. Xu and W.-M. He, *ACS Sustainable Chem. Eng.*, 2018, **6**, 4916-4921.
[12] For C-H borylation of fluorinated benzenes, see: D. W. Robbins and J. F. Hartwig, *Org. Lett.*, 2012, **14**, 4266-4269.

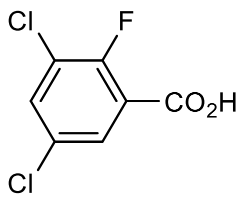

***3,5-Dichloro-2-fluorobenzoic acid*, p21.**[12] Starting from 1.820 mmol of corresponding arene the product was obtained as a white solid, m.p. = 162-164°C, yield 69% (0.261 g, Methylal). **^{1}H NMR** (400 MHz, MeOH-d4): δ = 7.75 (dd, *J* = 5.9, 2.7 Hz, 1H, Ar), 7.81 (dd, *J* = 5.6, 2.7 Hz, 1H, Ar). **^{13}C NMR** (101 MHz, MeOH-d4): δ = 124.7 (d, *J* = 20.0 Hz), 130.5 (d, *J* = 4.8 Hz), 131.5, 135.2, 157.6 (d, *J* = 261.2 Hz), 165.0. **HRMS-EI** (m/z) [M-H]$^-$ calcd. for $C_7H_2Cl_2FO_2$ 206.9421 found 206.9424.

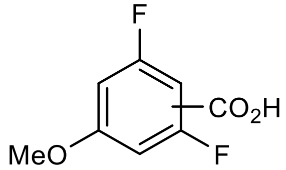

***Mixture of 2,6-difluoro-4-methoxybenzoic acid and 2,4-difluoro-6-methoxybenzoic acid*, 10:1.5, p22.**[12,13] Starting from 1.730 mmol of corresponding arene the product was obtained as a light brown viscous oil, yield 57% (0.185 g, Methylal). **^{1}H NMR** (400 MHz, MeOH-d4): δ = 3.84 (s, 2.92H, OMe, major isomere), 3.91 (s, 0.86H, OMe, minor isomere), 6.60-6.65 (m, 2H, Ar, major isomere), 6.84 (dd, *J* = 12.1, 1.8 Hz, 0.3H, Ar, minor isomere). **^{13}C NMR** (101 MHz, MeOH-d4): δ = 56.9, 57.6, 99.5 (d, *J* = 2.7 Hz), 99.7 (d, *J* = 2.8 Hz), 164.9, 165.1. **HRMS-EI** (m/z) [M-H]$^-$ calcd. for $C_8H_5F_2O_3$ 187.0212 found 187.0217.

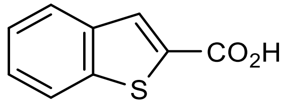

***Benzo[b]thiophene-2-carboxylic acid*, p23.**[2] Starting from 1.860 mmol of corresponding arene the product was obtained as a white solid, m.p. = above 200°C, yield 88% (0.293 g, Methylal), 64% (0.213 g, Methylal/DEC), 44% (0.146 g, Methylal/DMC). **^{1}H NMR** (400 MHz, MeOH-d4): δ = 7.43 (dddd, *J* = 20.2, 8.1, 7.1, 1.3 Hz, 2H, Ar), 7.89-7.93 (m, 2H, Ar), 8.04 (d, *J* = 0.8 Hz, 1H, thiophene). **^{13}C NMR** (101 MHz, MeOH-d4): δ = 123.8, 126.1, 126.7, 128.2, 131.7, 140.4, 143.7. **HRMS-EI** (m/z) [M-H]$^-$ calcd. for $C_9H_5O_2S$ 177.0016 found 177.0020.

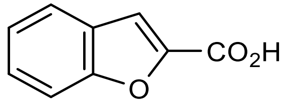

***Benzofuran-2-carboxylic acid*, p24.**[2] Starting from 1.690 mmol of corresponding arene the product was obtained as a white solid, m.p. = 193-195°C, yield 90% (0.247 g, Methylal). **^{1}H NMR** (400 MHz, MeOH-d4): δ = 7.28-7.33 (m, 1H, Ar), 7.44-7.48 (m, 1H, Ar), 7.55-7.58 (m, 2H, Ar), 7.72 (dt, *J* = 7.9, 1.0 Hz, 1H, furan). **^{13}C NMR** (101 MHz, MeOH-d4): δ = 113.0, 114.9, 124.1, 125.0, 128.6, 128.8, 147.5, 157.2, 162.5. **HRMS-EI** (m/z) [M-H]$^-$ calcd. for $C_9H_5O_3$ 161.0244 found 161.0248.

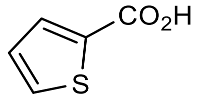

***Thiophene-2-carboxylic acid*, p25.**[3] Starting from 2.380 mmol of corresponding arene the product was obtained as a white solid, m.p. = 125-127°C, yield 93% (0.284 g, Methylal). Starting from 0.952 mmol of corresponding boronic acid pinacol ester the product was obtained as a white solid, m.p. = 125-127°C, yield 76% (0.093 g, DEC), 75% (0.092 g, DMC), 85% (0.104 g, Methylal). **^{1}H NMR** (400 MHz, $CDCl_3$): δ = 7.15 (dd, *J* = 4.9, 3.8 Hz, 1H, thiophene), 7.66 (dd, *J* = 5.0, 1.3 Hz, 1H, thiophene),

[13] C. Alonso-Alija, M. Michels, K. Peilstocker and H. Schirok, *Tetrahedron Lett.,* 2004, **45**, 95-98.

7.91 (dd, J = 3.8, 1.3 Hz, 1H, thiophene), 12.00 (br s, 1H, CO_2H). **^{13}C NMR** (101 MHz, $CDCl_3$): δ = 128.3, 133.1, 134.3, 135.3, 168.2. **HRMS-EI** (m/z) $[M-H]^-$ calcd. for $C_5H_3O_2S$ 126.9859 found 126.9863.

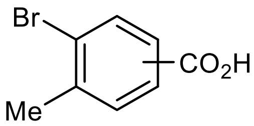

***Mixture of 4-bromo-3-methylbenzoic acid and 3-bromo-4-methylbenzoic acid*, 10:4.6, p26.**[14] Starting from 1.750 mmol of corresponding arene the product was obtained as a white solid, m.p. = above 200°C, yield 75% (0.281 g, Methylal). **^{1}H NMR** (400 MHz, MeOH-d4): δ = 2.44 (d, J = 3.4 Hz, 4.24H, Me, both isomers), 7.38 (d, J = 7.9 Hz, 1H, Ar, major isomere), 7.63 (d, J = 8.3 Hz, 0.46H, Ar, minor isomere), 7.70 (dd, J = 8.3, 2.1 Hz, 0.46H, Ar, minor isomere), 7.87 (dd, J = 7.9, 1.7 Hz, 1H, Ar, major isomere), 7.91 (d, J = 2.1 Hz, 0.49H, Ar, minor isomere), 8.13 (d, J = 1.7 Hz, 1H, Ar, major isomere). **^{13}C NMR** (101 MHz, MeOH-d4): δ = 23.0, 23.3, 125.6, 129.8, 129.8, 132.1, 133.0, 133.7, 134.6, 144.5, 168.4. **HRMS-EI** (m/z) $[M-H]^-$ calcd. for $C_8H_6BrO_2$ 212.9557 found 212.9560.

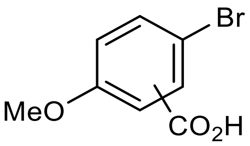

***Mixture of 5-bromo-2-methoxybenzoic acid and 2-bromo-5-methoxybenzoic acid*, 10:1.7, p27.**[15] Starting from 1.600 mmol of corresponding arene the product was obtained as a yellowish viscous oil, yield 58% (0.214 g, Methylal). **^{1}H NMR** (400 MHz, MeOH-d4): δ = 3.84 (d, J = 8.1 Hz, 3.62H, OMe, both isomers), 6.95-7.00 (m, 1.28H, Ar, both isomers), 7.53-7.57 (m, 1.20H, Ar, both isomers), 7.77 (d, J = 2.6 Hz, 0.17H, Ar, minor isomere), 7.86 (d, J = 2.7 Hz, 1H, Ar, major isomere). **^{13}C NMR** (101 MHz, MeOH-d4): δ = 56.7, 56.8, 108.0, 112.9, 113.2, 113.3, 115.5, 121.5, 122.7, 123.0, 133.0, 134.8, 135.2, 137.4, 137.5, 159.6, 166.9, 168.0. **HRMS-EI** (m/z) $[M-H]^-$ calcd. for $C_8H_6BrO_3$ 228.9506 found 228.9507.

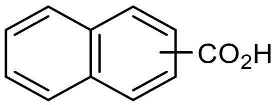

***Mixture of 2-naphthoic acid and 1-naphthoic acid*, 10:1.6, p28.**[18,16a,3] Starting from 2.340 mmol of corresponding arene the product was obtained as a white solid, m.p. = above 200°C, yield 80% (0.322 g, Methylal). **^{1}H NMR** (400 MHz, MeOH-d4): δ = 7.51-7.60 (m, 2H, Ar, major isomere), 7.82-8.08 (m, 5.57H, Ar, both isomers), 8.34-8.40 (m, 0.33H, Ar, minor isomere), 8.55-8.63 (m, 1.37H, Ar, both isomers). **^{13}C NMR** (101 MHz, MeOH-d4): δ = 126.4, 126.5, 127.9, 128.1, 128.9, 129.2, 129.3, 129.4, 130.0, 130.4, 132.0, 132.2, 132.9, 133.8, 134.0, 136.9, 137.1, 138.6, 170.0. **HRMS-EI** (m/z) $[M-H]^-$ calcd. for $C_{11}H_7O_2$ 171.0452 found 171.0456.

---

[14] (a) A. M. Fleifel, *J. Org. Chem.,* 1960, **25**, 1024-1025; (b) D. Nasipuri, A. K. Samaddar and I. Datta, *J. Chem. Soc. Perkin Trans. 1,* 1979, 3034-3036.
[15] (a) M. Zhao, J. Li, E. Mano, Z. Song, D. M. Tschaen, E. J. J. Grabowski and P. J. Reider, *J. Org. Chem.,* 1999, **64**, 2564-2566; (b) T. Kunkle, S. Abdeen, N. Salim, A.-M. Ray, M. Stevens, A. J. Ambrose, J. Victorino, Y. Park, Q. Q. Hoang, E. Chapman and S. M. Johnson, *J. Med. Chem.,* 2018, **61**, 10651-10664.
[16] (a) K. Paridala, S.-M. Lu, M.-M. Wang and C. Li, *Chem. Commun.,* 2018, **54**, 11574-11577; (b) H.-M. Liu, L. Jian, C. Li, C.-C. Zhang, H.-Y. Fu, X.-L. Zheng, H. Chen and R.-X. Li, *J. Org. Chem.,* 2019, **84**, 9151-9160.

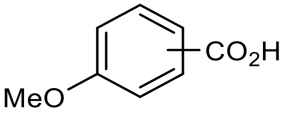

***Mixture of 3-methoxybenzoic acid (isomer 2), 4-methoxybenzoic acid (isomer 1) and 2-methoxybenzoic acid (isomer 3)*, 10:3:1.5, p29.**[16b] Starting from 1.850 mmol of corresponding arene the product was obtained as a white solid, m.p. = 91-94°C, yield 67% (0.188 g, Methylal). **^{1}H NMR** (400 MHz, MeOH-d4): δ = 3.80-3.83 (m, 4.13H, OMe, all isomers), 6.92-6.96 (m, 0.57H, Ar, isomer 1), 7.10-7.14 (m, 0.99H, Ar, isomer 2), 7.33-7.37 (m, 1H, Ar, isomer 2), 7.46-7.47 (m, 0.15H, Ar, isomer 3), 7.53-7.54 (m, 0.97H, Ar, isomer 2), 7.58-7.63 (m, 1.12H, Ar, isomer 2/3), 7.94-7.99 (m, 0.69H, Ar, isomer 1). **^{13}C NMR** (101 MHz, MeOH-d4): δ = 55.9, 55.9, 56.0, 85.6, 114.7, 115.6, 118.7, 120.1, 123.1, 124.1, 125.3, 129.3, 130.6, 132.9, 133.3, 160.7, 161.2, 165.1, 169.8. **HRMS-EI** (m/z) $[M-H]^-$ calcd. for $C_8H_7O_3$ 151.0401 found 151.0393.

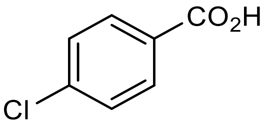

***4-Chlorobenzoic acid*, p30.**[3] Starting from 0.839 mmol of corresponding boronic acid pinacol ester the product was obtained as a white solid, m.p. = above 200°C, yield 77% (0.101 g, DEC), 60% (0.079 g, DMC), 79% (0.103 g, Methylal). **^{1}H NMR** (400 MHz, MeOH-d4): δ = 7.46-7.49 (m, 2H, Ar), 7.97-8.01 (m, 2H, Ar). **^{13}C NMR** (101 MHz, MeOH-d4): δ = 129.8, 130.8, 132.4, 140.4, 168.8. **HRMS-EI** (m/z) $[M-H]^-$ calcd. for $C_7H_4ClO_2$ 154.9905 found 154.9910.

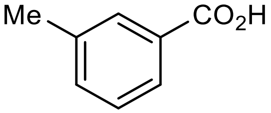

***3-Methylbenzoic acid*, p31.**[17] Starting from 0.917 mmol of corresponding boronic acid pinacol ester the product was obtained as a white solid, m.p. = 108-111°C, yield 79% (0.098 g, DEC), 51% (0.064 g, DMC), 71% (0.088 g, Methylal). **^{1}H NMR** (400 MHz, $CDCl_3$): δ = 2.43 (s, 3H, Me), 7.35-7.39 (m, 1H, Ar), 7.42-7.45 (m, 1H, Ar), 7.93-7.95 (m, 2H, Ar), 11.75 (br s, 1H, CO_2H). **^{13}C NMR** (101 MHz, $CDCl_3$): δ = 21.4, 127.6, 128.6, 129.5, 131.0, 134.8, 138.5, 172.8. **HRMS-EI** (m/z) $[M-H]^-$ calcd. for $C_8H_7O_2$ 135.0452 found 135.0457.

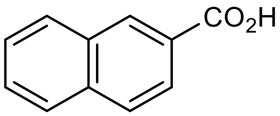

***2-Naphthoic acid*, p32.**[18,16a,3] Starting from 0.787 mmol of corresponding boronic acid pinacol ester the product was obtained as a white solid, m.p. = 185-187°C, yield 61% (0.082 g, DEC). **^{1}H NMR** (400 MHz, $CDCl_3$): δ = 7.61 (dddd, *J* = 22.1, 8.1, 6.9, 1.4 Hz, 2H, Ar), 7.90-7.94 (m, 2H, Ar), 7.99-8.01 (m, 1H, Ar), 8.14 (dd, *J* = 8.6, 1.7 Hz, 1H, Ar), 8.74 (d, *J* = 1.7 Hz, 1H, Ar). **^{13}C NMR** (101 MHz, $CDCl_3$): δ = 125.6, 126.7, 127.0, 128.0, 128.5, 128.9, 129.8, 132.4, 132.7, 136.2, 172.5. **HRMS-EI** (m/z) $[M-H]^-$ calcd. for $C_{11}H_7O_2$ 171.0452 found 171.0444.

---

[17] F.-P. Wu, J.-B. Peng, L.-S. Meng, X. Qi and X.-F. Wu, *ChemCatChem,* 2017, **16**, 3121-3124.

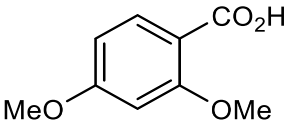

***2,4-Dimethoxybenzoic acid*, p33.**[18] Starting from 0.757 mmol of corresponding boronic acid pinacol ester the product was obtained as a white solid, m.p. = 107-109ºC, yield 81% (0.112 g, DEC). **^{1}H NMR** (400 MHz, $CDCl_3$): δ = 3.86 (s, 3H, OMe), 4.02 (s, 3H, OMe), 6.52 (d, *J* = 2.2 Hz, 1H, Ar), 6.61 (dd, *J* = 8.8, 2.3 Hz, 1H, Ar), 8.09 (d, *J* = 8.8 Hz, 1H, Ar), 9.82 (br s, 1H, CO_2H). **^{13}C NMR** (101 MHz, $CDCl_3$): δ = 55.9, 56.8, 98.8, 106.7, 110.4, 135.6, 159.8, 165.3, 165.8. **HRMS-EI** (m/z) $[M-H]^-$ calcd. for $C_9H_9O_4$ 181.0506 found 181.0496.

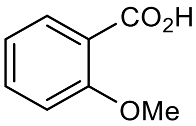

***2-Methoxybenzoic acid*, p34.**[18] Starting from 0.854 mmol of corresponding boronic acid pinacol ester the product was obtained as a white solid, m.p. = 98-100ºC, yield 68% (0.088 g, DEC). **^{1}H NMR** (400 MHz, $CDCl_3$): δ = 4.05 (s, 3H, OMe), 7.04-7.06 (m, 1H, Ar), 7.09-7.13 (m, 1H, Ar), 7.56 (ddd, *J* = 8.4, 7.4, 1.9 Hz, 1H, Ar), 8.13 (dd, *J* = 7.8, 1.9 Hz, 1H, Ar), 9.57 (br s, 1H, CO_2H). **^{13}C NMR** (101 MHz, $CDCl_3$): δ = 56.8, 111.9, 117.7, 122.2, 133.8, 135.2, 158.3, 165.9. **HRMS-EI** (m/z) $[M-H]^-$ calcd. for $C_8H_7O_3$ 151.0401 found 151.0396.

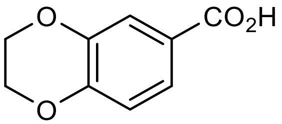

***2,3-Dihydrobenzo[b][1,4]dioxine-6-carboxylic acid*, p35.**[19] Starting from 0.763 mmol of corresponding boronic acid pinacol ester the product was obtained as a white solid, m.p. = 134-138ºC, yield 84% (0.116 g, DEC). **^{1}H NMR** (400 MHz, $CDCl_3$): δ = 4.27-4.34 (m, 4H, 2 x CH_2), 6.90-6.93 (m, 1H, Ar), 7.62-7.65 (m, 2H, Ar), 12.07 (br s, 1H, CO_2H). **^{13}C NMR** (101 MHz, $CDCl_3$): δ = 64.2, 64.9, 117.4, 119.8, 122.7, 124.4, 143.4, 148.7, 172.0. **HRMS-EI** (m/z) $[M-H]^-$ calcd. for $C_9H_7O_4$ 179.0350 found 179.0344.

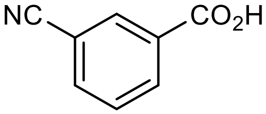

***3-Cyanobenzoic acid*, p36.**[20] Starting from 0.873 mmol of corresponding boronic acid pinacol ester the product was obtained as a white solid, m.p. = above 200ºC, yield 59% (0.076 g, DEC). **^{1}H NMR** (400 MHz, MeOH-d4): δ = 7.67 (t, *J* = 7.8 Hz, 1H, Ar), 7.94 (dt, *J* = 7.7, 1.5 Hz, 1H, Ar), 8.27-8.32 (m, 2H, Ar). **^{13}C NMR** (101 MHz, MeOH-d4): δ = 114.0, 119.0, 131.0, 134.4, 135.0, 137.2, 167.6. **HRMS-EI** (m/z) $[M-H]^-$ calcd. for $C_8H_4NO_2$ 146.0248 found 146.0244.

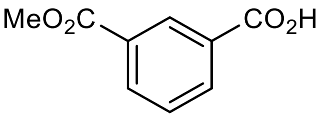

***3-(Methoxycarbonyl)benzoic acid*, p37.**[21] Starting from 0.763 mmol of corresponding boronic acid pinacol ester the product was obtained as a white solid, m.p. = 194-196ºC, yield 55% (0.075 g, DEC). **^{1}H NMR** (400 MHz, MeOH-d4): δ = 3.93 (s, 3H, CO_2Me), 7.56-7.60 (m, 1H, Ar), 8.18-8.24 (m, 2H, Ar), 8.61-8.62 (m, 1H, Ar). **^{13}C**

[18] A. Nakamura, H. Kanou, J. Tanaka, A. Imamiya, T. Maegawa and Y. Miki, *Org. Biomol. Chem.*, 2018, **16**, 541-544.
[19] S. Korsager, R. H. Taaning and T. Skrydstrup, *J. Am. Chem. Soc.*, 2013, **135**, 2891-2894.
[20] D. A. Watson, X. Fan and S. L. Buchwald, *J. Org. Chem.*, 2008, **73**, 7096-7101.
[21] J. T. Joseph, A. M. Sajith, R. C. Ningegowda and S. Shashikanth, *Adv. Synth. Catal.*, 2017, **359**, 419-425.

NMR (101 MHz, MeOH-d4): δ = 52.9, 130.0, 131.7, 131.9, 132.7, 134.7, 135.1, 167.7, 168.7. **HRMS-EI** (m/z) $[M-H]^-$ calcd. for $C_9H_7O_4$ 179.0350 found 179.0339.

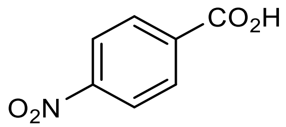

***4-Nitrobenzoic acid*, p38.**[18] Starting from 0.803 mmol of corresponding boronic acid pinacol ester the product was obtained as a white solid, m.p. = above 200ºC, yield 51% (0.068 g, DEC). **^{1}H NMR** (400 MHz, MeOH-d4): δ = 8.21-8.24 (m, 2H, Ar), 8.30-8.33 (m, 2H, Ar). **^{13}C NMR** (101 MHz, MeOH-d4): δ = 124.6, 132.0, 152.1, 167.7. **HRMS-EI** (m/z) $[M-H]^-$ calcd. for $C_7H_4NO_4$ 166.0146 found 166.0136.

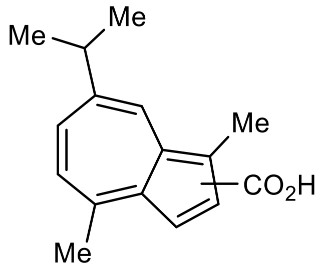

***Mixture of 7-isopropyl-1,4-dimethylazulene-2-carboxylic acid and 5-isopropyl-3,8-dimethylazulene-1-carboxylic acid*, 10:3.6, p39.**[22] Starting from 1.760 mmol of Guaiazulene the product was obtained as a dark blue viscous oil, yield 46% (0.196 g, Methylal). **^{1}H NMR** (400 MHz, DMSO-d6): δ = 1.28-1.35 (m, 9.61H, Me, both isomers), 2.76-2.80 (m, 5.34H, Me, both isomers), 3.07-3.18 (m, 1.67H, CH, both isomers), 7.08-7.15 (m, 1.50H, Ar, both isomers), 7.55-7.58 (m, 1.61H, Ar, both isomers), 7.61-7.67 (m, 1.19H, Ar, both isomers), 8.37 (d, *J* = 1.9 Hz, 0.36H, Ar, minor isomer), 8.40-8.41 (m, 1H, Ar, major isomer). **^{13}C NMR** (101 MHz, DMSO-d6): δ = 11.7, 24.0, 24.5, 24.6, 25.2, 26.2, 30.6, 34.6, 37.5, 37.6, 42.7, 43.3, 48.8, 73.7, 115.0, 115.1, 125.1, 126.2, 126.8, 127.0, 127.2, 127.5, 134.8, 135.3, 135.5, 135.6, 135.7, 137.2, 137.6, 137.7, 137.8, 137.9, 138.1, 138.6, 141.2, 141.9, 142.1, 143.9, 144.8, 148.6, 167.5, 167.6, 171.2, 172.2. **HRMS-EI** (m/z) $[M-H]^-$ calcd. for $C_{16}H_{17}O_2$ 241.1234 found 241.1235.

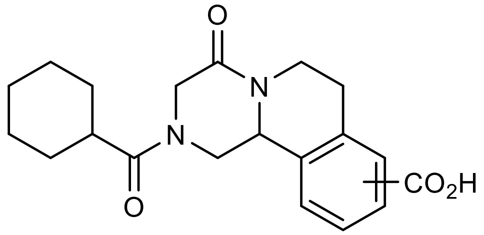

***Mixture of 2-(cyclohexanecarbonyl)-4-oxo-1,3,4,6,7,11b-hexahydro-2H-pyrazino[2,1-a]isoquinoline-10-carboxylic acid and 2-(cyclohexanecarbonyl)-4-oxo-1,3,4,6,7,11b-hexahydro-2H-pyrazino[2,1-a]isoquinoline-9-carboxylic acid*, 10:1.2, p40.** Starting from 1.600 mmol of Praziquantel the product was obtained as a yellowish viscous oil, yield 40% (0.226 g, Methylal). **^{1}H NMR** (400 MHz, DMSO-d6): δ = 1.14-1.22 (m, 1.81H, Cy, both isomers), 1.27-1.41 (m, 5.73H, Cy, both isomers), 1.62-1.74 (m, 6.94H, Cy, both isomers), 2.62-2.67 (m, 0.69H, CH_2, minor isomer), 2.89-2.95 (m, 2.10H, CH_2, major isomer), 3.34-3.40 (m, 0.29H, CH_2, minor isomer), 3.47 (t, *J* = 11.8 Hz, 0.58H, CH_2, minor isomer), 3.70-3.80 (m, 1.17H, CH_2, major isomer), 4.10 (d, *J* = 17.2 Hz, 1.02H, CH_2, major isomer), 4.38-4.57 (m, 3.60H, CH_2, both isomers), 4.83-4.86 (m, 1.34H, CH_2, both isomers), 5.01-5.05 (m, 0.65H, CH_2, minor isomer), 7.34 (d, *J* = 7.9 Hz, 1H, Ar, major isomer), 7.41 (d, *J* = 8.2 Hz, 0.12H, Ar, minor isomer), 7.63 (d, *J* = 8.2 Hz, 0.17H, Ar, minor isomer), 7.79-7.84 (m, 2.09H, Ar, major isomer), 7.99 (s, 0.44H, Ar, minor isomer).

---

[22] For C-H borylation of Guaiazulene, see: Z.-T. He, H. Li, A. M. Haydl, G. T. Whiteker and J. F. Hartwig, *J. Am. Chem. Soc.*, 2018, **140**, 17197-17202.

^{13}C NMR (101 MHz, DMSO-d6): δ = Due to poor solubility it was not possible to measure. **HRMS-EI** (m/z) $[M-H]^-$ calcd. for $C_{20}H_{23}N_2O_4$ 355.1663 found 355.1661.

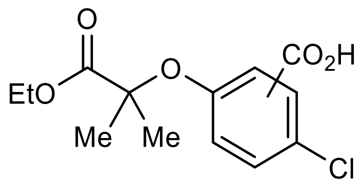

***Mixture of 5-chloro-2-((1-ethoxy-2-methyl-1-oxopropan-2-yl)oxy)benzoic acid and 2-chloro-5-((1-ethoxy-2-methyl-1-oxopropan-2-yl)oxy)benzoic acid*, 10:1.1, p41.** Starting from 1.650 mmol of Clofibrate the product was obtained as a yellowish viscous oil, yield 34% (0.159 g, Methylal). **^{1}H NMR** (400 MHz, MeOH-d4): δ = 1.23 (t, *J* = 7.2 Hz, 4.15H, Me, both isomers), 1.57-1.58 (m, 6.86H, Me, both isomers), 4.21 (q, *J* = 7.1 Hz, 2.22H, CH_2, both isomers), 6.97 (dd, *J* = 8.8, 3.1 Hz, 1H, Ar, major isomer), 7.30 (d, *J* = 3.1 Hz, 1H, Ar, major isomer), 7.35 (d, *J* = 8.8 Hz, 1.22H, Ar, both isomers), 7.71-7.72 (m, 0.33H, Ar, minor isomer). **^{13}C NMR** (101 MHz, MeOH-d4): δ = 14.4, 23.2, 25.7, 31.0, 62.9, 81.1, 115.9, 120.8, 122.5, 122.8, 123.9, 124.7, 125.1, 125.7, 127.3, 132.8, 155.5, 168.4, 175.0. **HRMS-EI** (m/z) $[M-H]^-$ calcd. for $C_{13}H_{14}ClO_5$ 285.0535 found 285.0531.

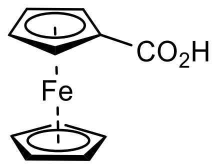

***Ferrocenecarboxylic acid*, p42.**[23] Starting from 1.610 mmol of ferrocene the product was obtained as a red solid, m.p. = above 200°C, yield 37% (0.137 g, Methylal). **^{1}H NMR** (400 MHz, MeOH-d4): δ = 4.22 (s, 5H, ferrocene), 4.45-4.46 (m, 2H, ferrocene), 4.77-4.78 (m, 2H, ferrocene). **^{13}C NMR** (101 MHz, MeOH-d4): δ = 71.1, 71.6, 72.9, 75.9. **HRMS-EI** (m/z) $[M-H]^-$ calcd. for $C_{11}H_9FeO_2$ 228.9957 found 228.9960.

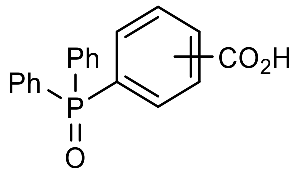

***Mixture of 3-(diphenylphosphoryl)benzoic acid and 4-(diphenylphosphoryl)benzoic acid*, 4:1, p43.** Starting from 1.800 mmol of corresponding phosphine oxide the product was obtained as a colourless viscous oil, yield 61% (0.355 g, Methylal). **^{1}H NMR** (400 MHz, MeOH-d4): δ = 7.48-7.54 (m, 4.56H, Ar, both isomers), 7.56-7.68 (m, 7.76H, Ar, both isomers), 7.76 (dd, *J* = 11.7, 8.3 Hz, 0.88H, Ar, minor isomer), 7.80-7.89 (m, 1.13H, Ar, major isomer), 8.13-8.18 (m, 0.92H, Ar, major isomer), 8.22-8.26 (m, 0.92H, Ar, major isomer), 8.32-8.39 (m, 1H, Ar, major isomer). **^{13}C NMR** (101 MHz, MeOH-d4): δ = 130.0, 130.2, 130.3, 130.4, 130.6, 130.8, 130.9, 131.0, 131.5, 132.5, 132.6, 132.7, 132.8, 133.0, 133.1, 133.2, 133.7, 133.9, 134.0, 134.1, 134.7, 134.9, 135.7, 137.0, 137.1, 137.9, 168.2, 168.3. **HRMS-EI** (m/z) $[M-H]^-$ calcd. for $C_{19}H_{14}O_3P$ 321.0686 found 321.0684.

[23] (a) A. F. Neto, J. Miller, V. F. d. Andrade, S. Y. Fujimoto, M. M. d. F. Afonso, F. C. Archanjo, V. A. Darin, M. L. A. Silva, A. D. L. Borges and G. D. Ponte, *Z. Anorg. Allg. Chem.*, 2002, **628**, 209-216; (b) For C-H borylation of ferrocene, see: A. Datta, A. Kollhofer and H. Plenio, *Chem. Commun.*, 2004, 1508-1509.

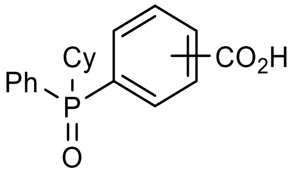

***Mixture of 4-(cyclohexyl(phenyl)phosphoryl)benzoic acid and 3-(cyclohexyl(phenyl)phosphoryl)benzoic acid,* 10:2.3, p44.** Starting from 1.760 mmol of corresponding phosphine oxide the product was obtained as a colourless viscous oil, yield 67% (0.387 g, Methylal). **^{1}H NMR** (400 MHz, MeOH-d4): δ = 1.26-1.36 (m, 3.27H, CH_2/Cy), 1.40-1.49 (m, 2.56H, CH_2/Cy), 1.59-1.74 (m, 6.35H, CH_2/Cy), 2.53-2.63 (m, 1.23H, CH/Cy), 7.48-7.55 (m, 3.11H, Ar), 7.63 (qd, J = 7.5, 2.8 Hz, 1H, Ar), 7.80-7.85 (m, 2H, Ar), 7.90-8.07 (m, 1.80H, Ar), 8.13-8.21 (m, 1.78H, Ar), 8.45-8.52 (m, 1H, Ar). **^{13}C NMR** (101 MHz, MeOH-d4): δ = 25.1, 25.7, 25.8, 26.8, 26.9, 27.1, 31.0, 36.8, 37.5, 50.0, 130.1, 130.2, 130.3, 130.4, 130.6, 130.8, 131.0, 131.2, 132.0, 132.1, 132.2, 132.7, 132.9, 133.0, 133.1, 133.2, 133.4, 133.7, 134.1, 134.2, 134.3, 135.3, 136.1, 136.2, 168.5, 168.6. **HRMS-EI** (m/z) $[M-H]^-$ calcd. for $C_{19}H_{20}O_3P$ 327.1156 found 327.1152.

Copies of spectra

Compound b2

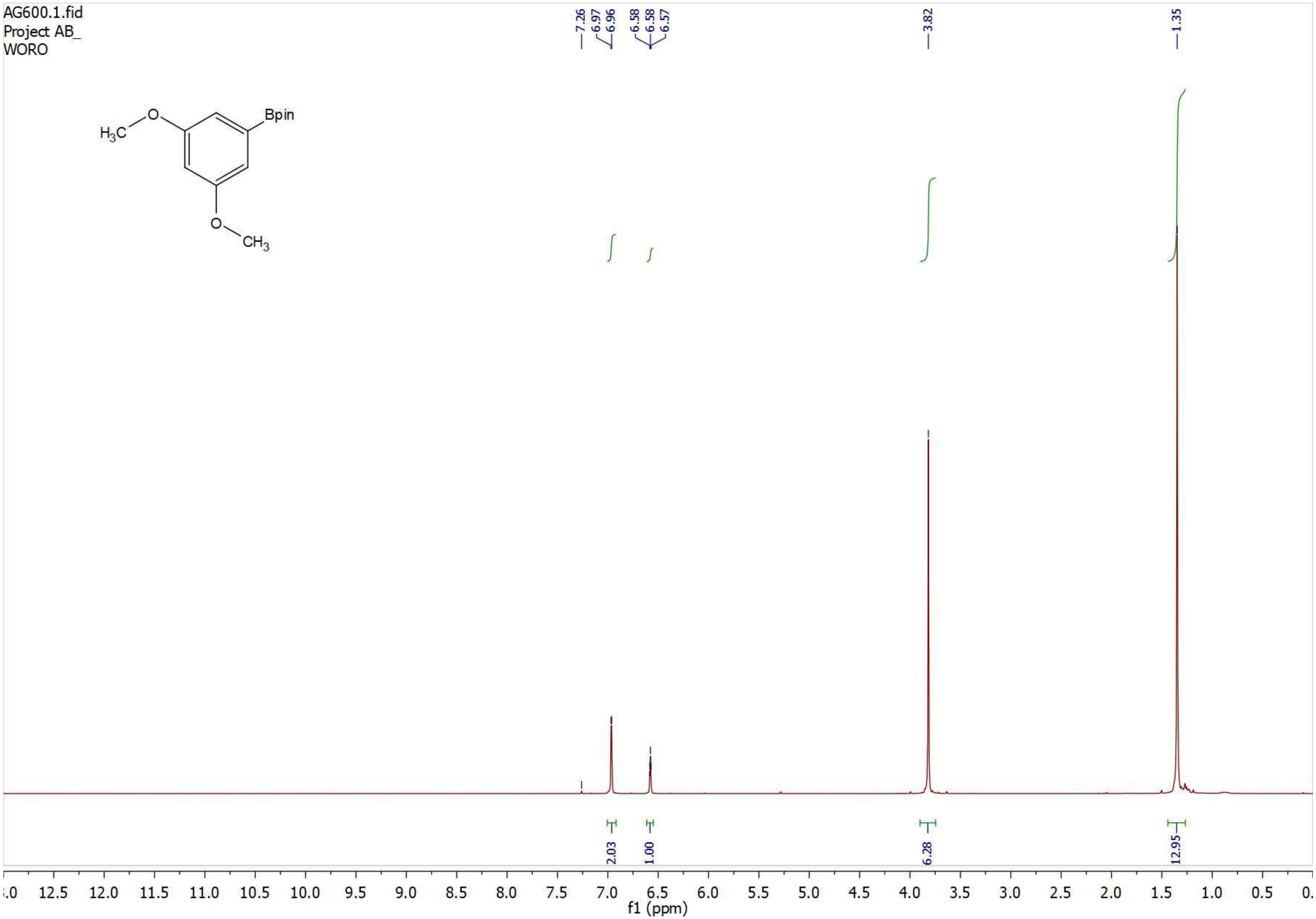

Compound b2

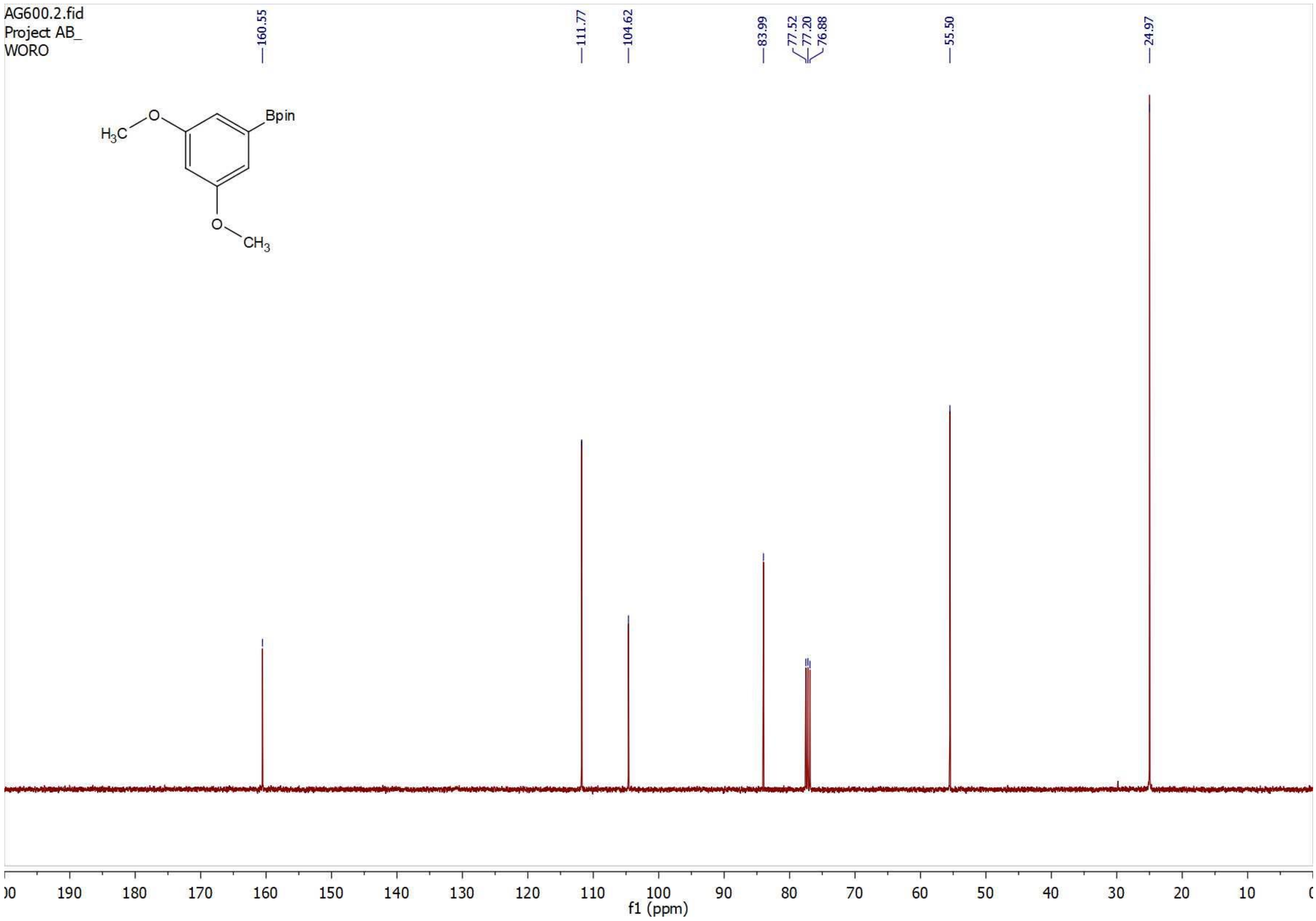
AG600.2.fid
Project AB_
WORO
160.55
111.77
104.62
83.99
77.52
77.20
76.88
55.50
24.97
Bpin
H_3C
O
O
CH_3
190
180
170
160
150
140
130
120
110
100
90
80
70
60
50
40
30
20
10
f1 (ppm)

Compound p1

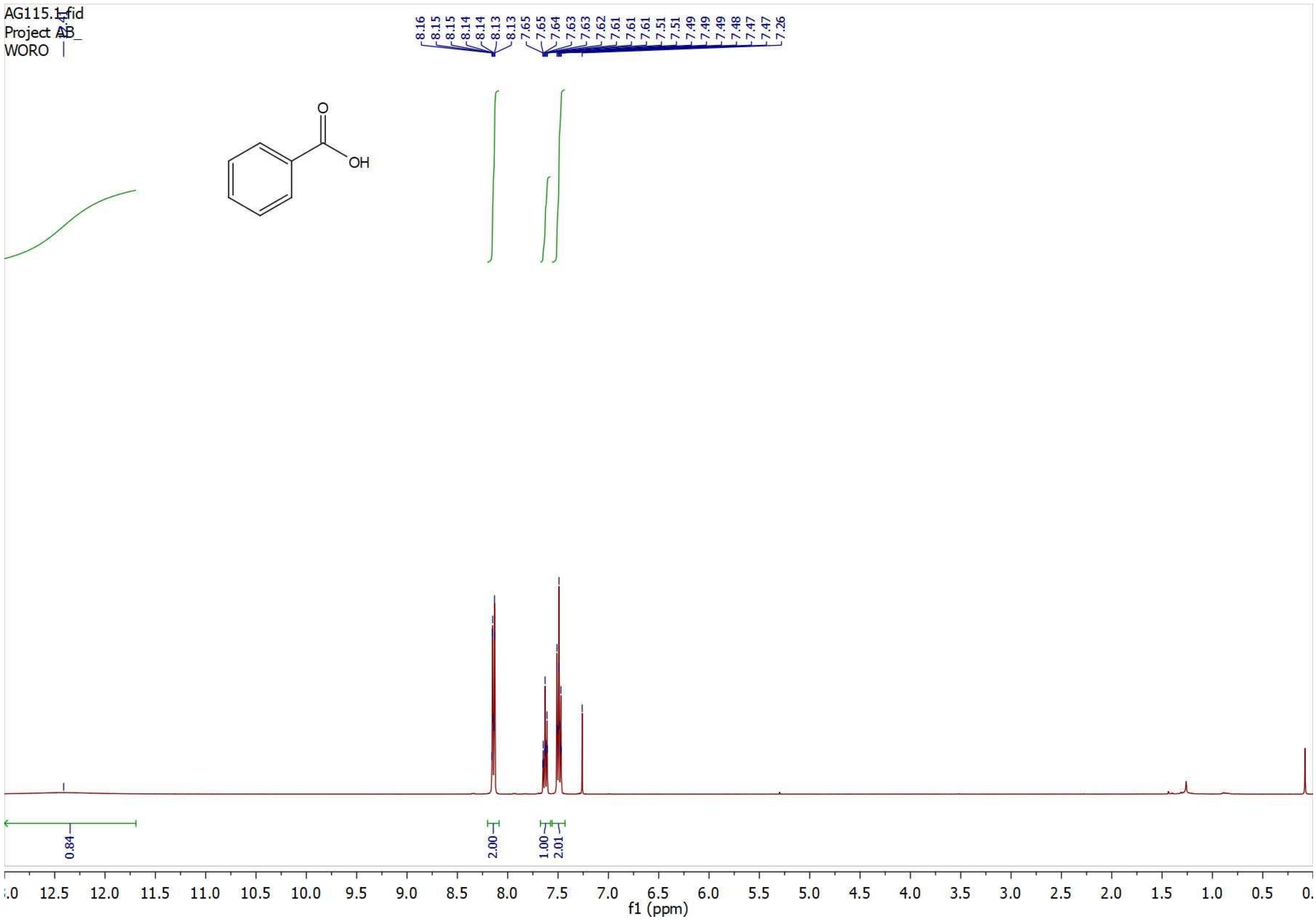
AG115.1.fid
Project AB_
WORO
8.16
8.15
8.15
8.14
8.14
8.13
8.13
7.65
7.65
7.64
7.63
7.63
7.62
7.61
7.61
7.61
7.51
7.51
7.49
7.49
7.49
7.48
7.47
7.47
7.26
OH
O
0.84
2.00
1.00
2.01
12.5
12.0
11.5
11.0
10.5
10.0
9.5
9.0
8.5
8.0
7.5
7.0
6.5
6.0
5.5
5.0
4.5
4.0
3.5
3.0
2.5
2.0
1.5
1.0
0.5
f1 (ppm)

Compound p1

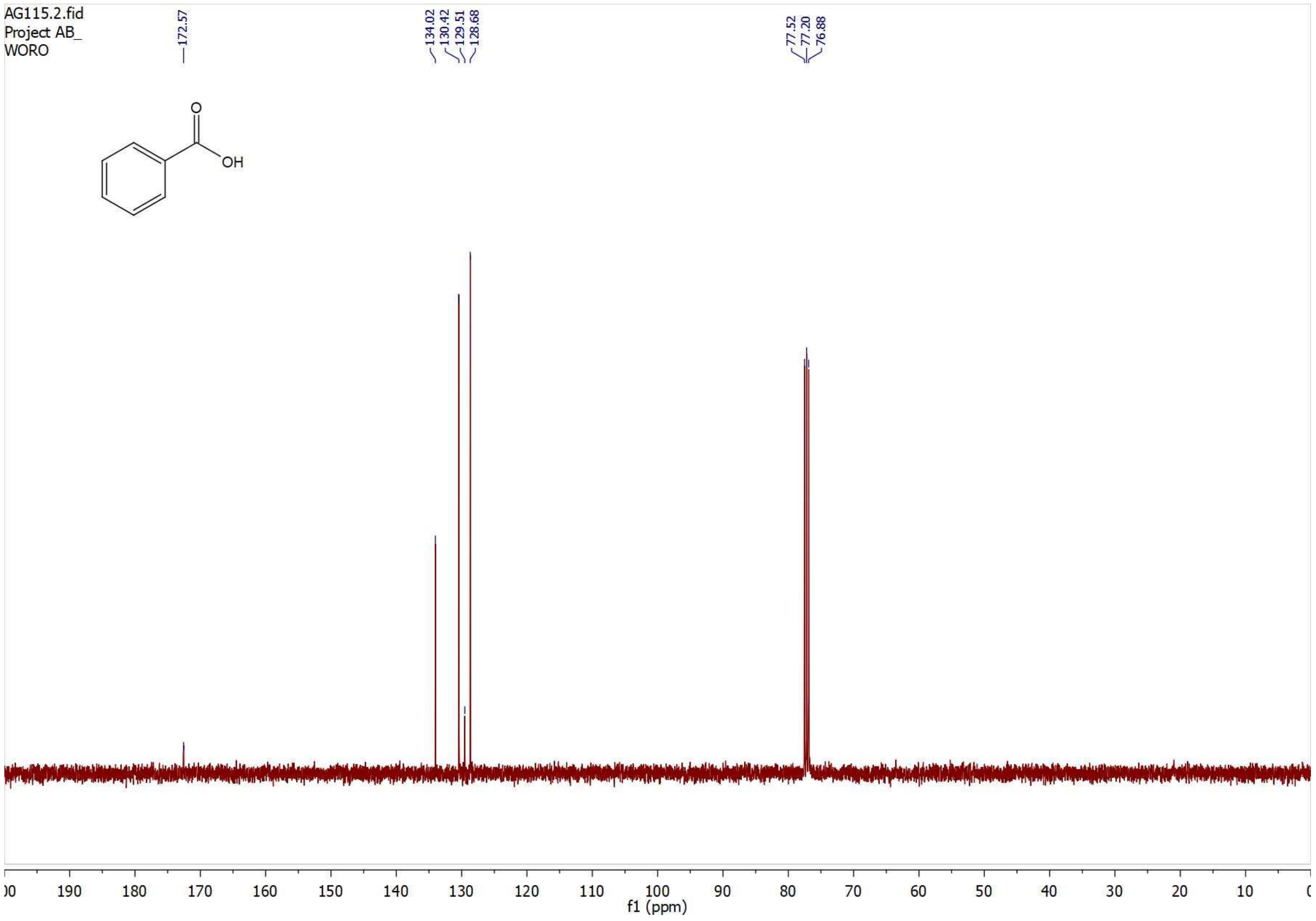
AG115.2.fid
Project AB_
WORO
172.57
134.02
130.42
129.51
128.68
77.52
77.20
76.88
O
OH
190
180
170
160
150
140
130
120
110
100
90
80
70
60
50
40
30
20
10
f1 (ppm)

Compound p2

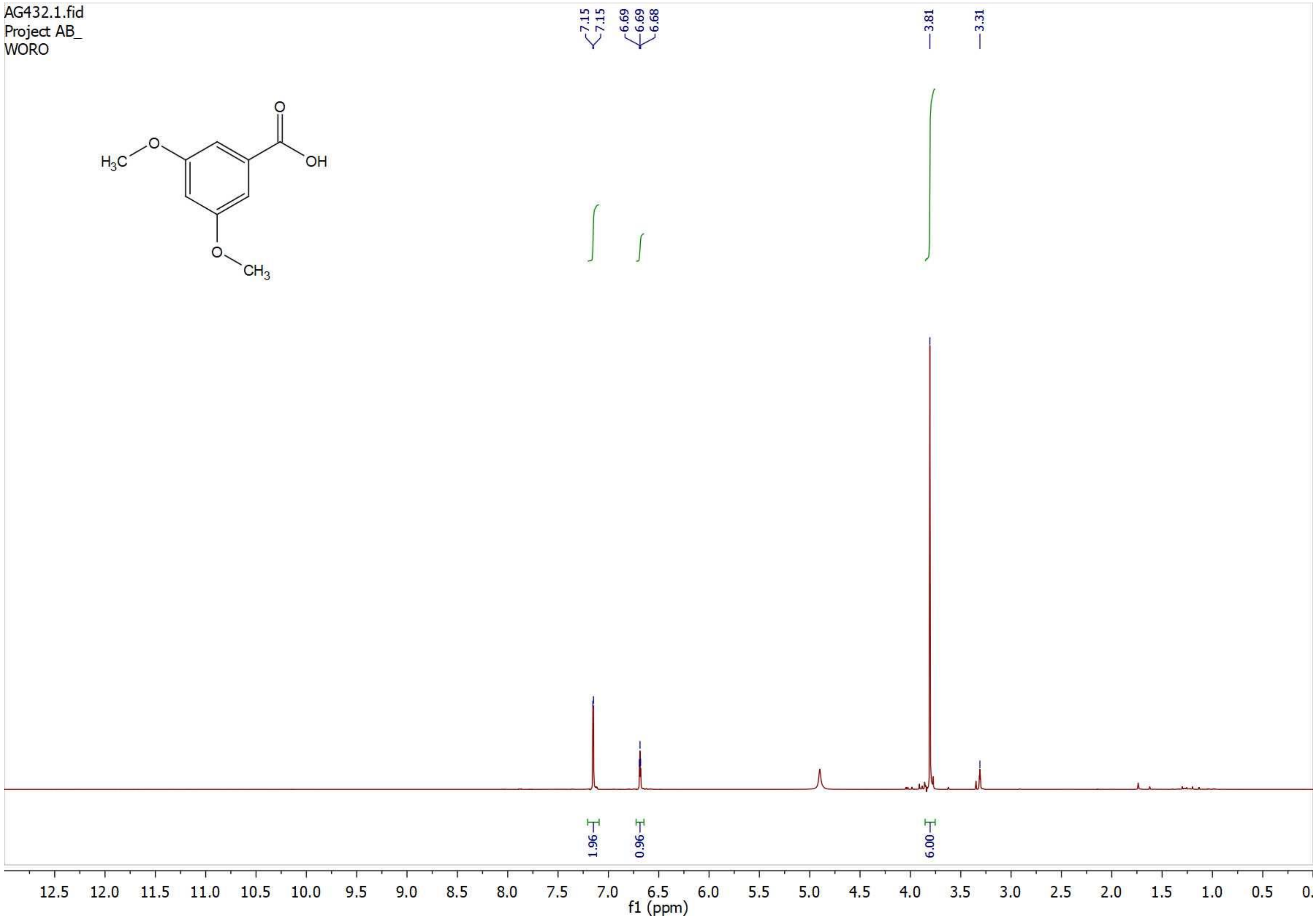
AG432.1.fid
Project AB_
WORO
7.15
7.15
6.69
6.69
6.68
3.81
3.31
O
OH
H3C
O
O
CH3
1.96
0.96
6.00
12.5 12.0 11.5 11.0 10.5 10.0 9.5 9.0 8.5 8.0 7.5 7.0 6.5 6.0 5.5 5.0 4.5 4.0 3.5 3.0 2.5 2.0 1.5 1.0 0.5 0.
f1 (ppm)

Compound p2

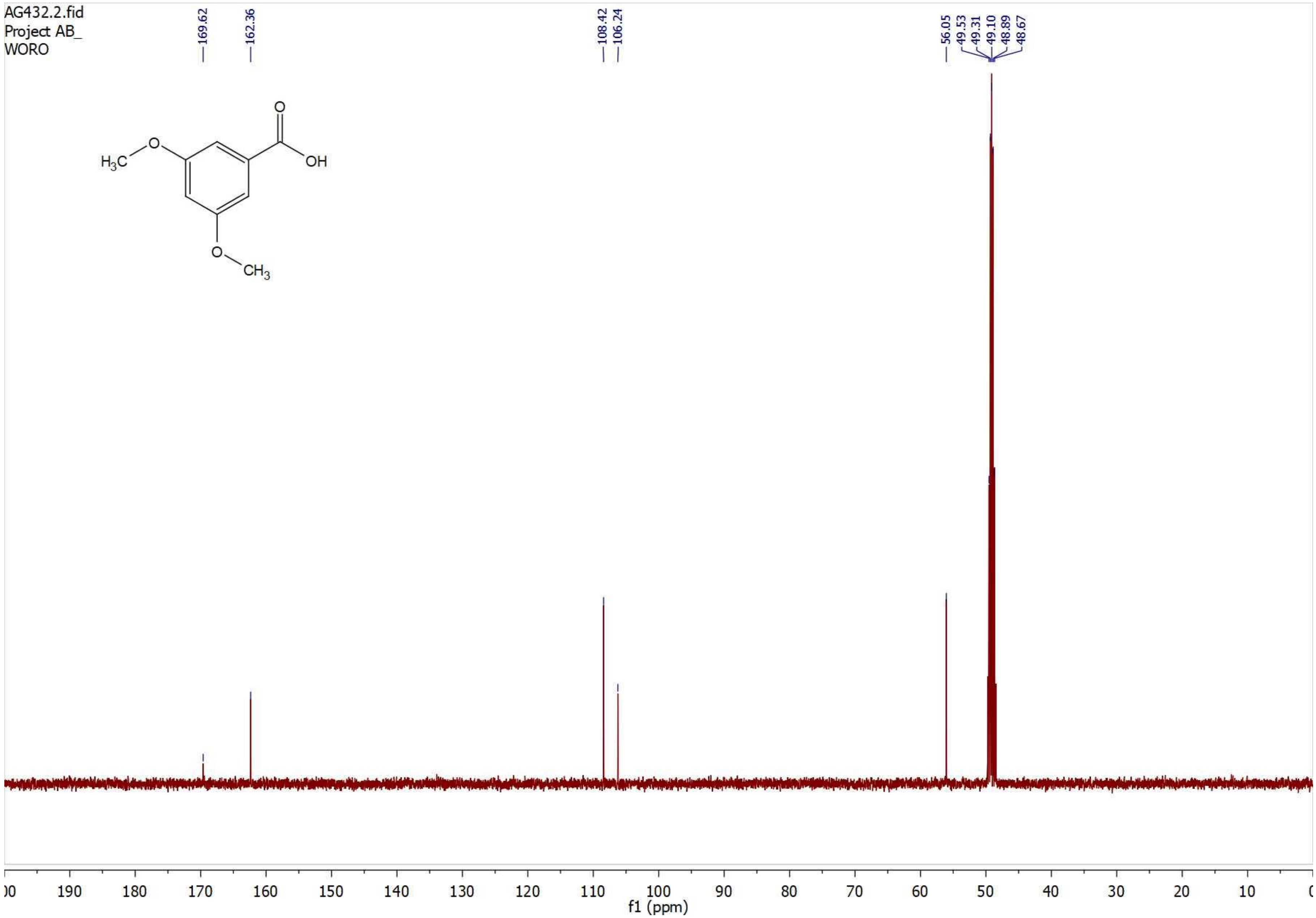

AG432.2.fid
Project AB_
WORO
169.62
162.36
108.42
106.24
56.05
49.53
49.31
49.10
48.89
48.67
O
OH
H3C
O
O
CH3
190
180
170
160
150
140
130
120
110
100
90
80
70
60
50
40
30
20
10
f1 (ppm)

Compound p3

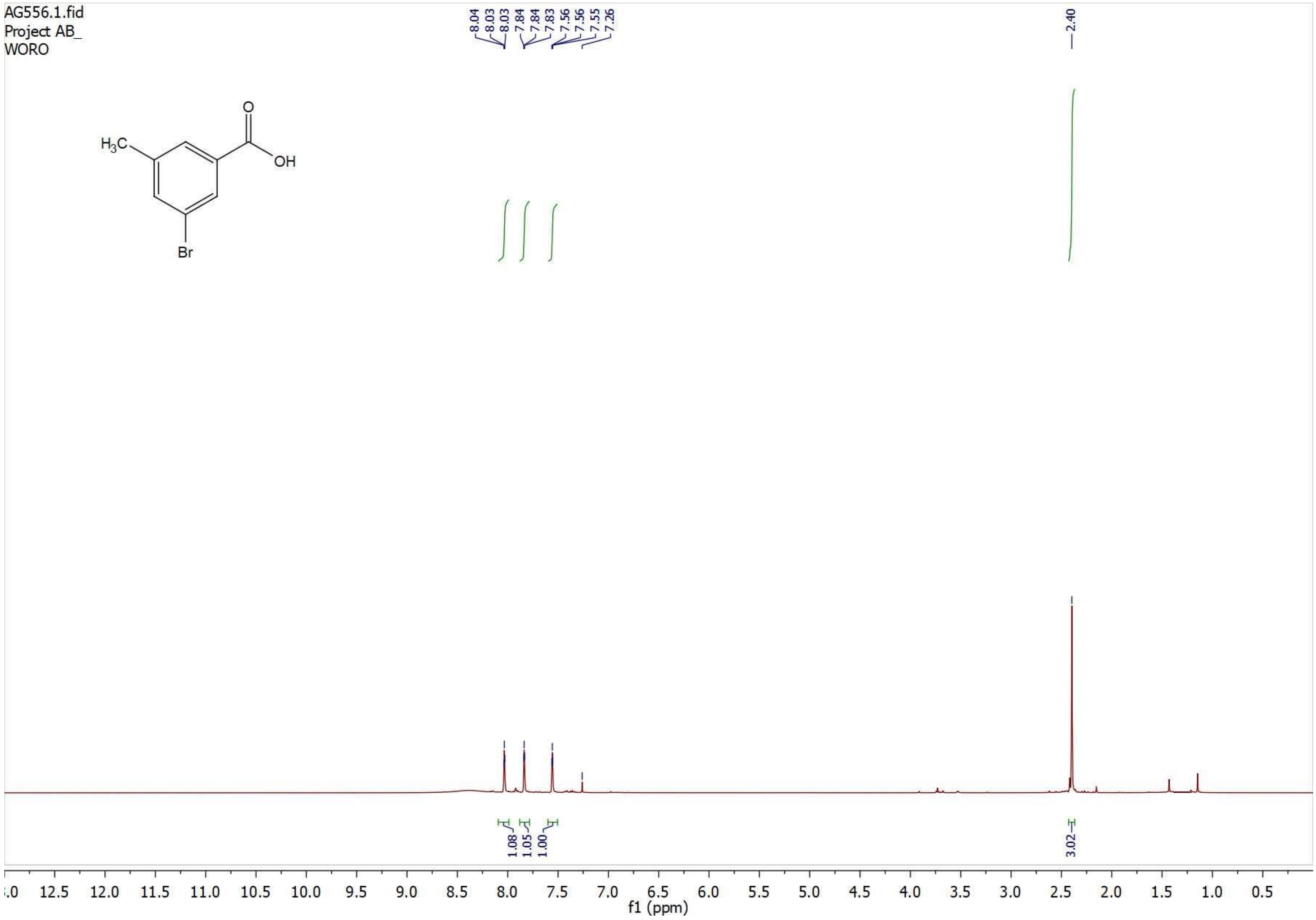

AG556.1.fid
Project AB_
WORO
8.04
8.03
8.03
7.84
7.84
7.83
7.56
7.56
7.55
7.26
2.40
H3C
O
OH
Br
1.08
1.05
1.00
3.02
12.5 12.0 11.5 11.0 10.5 10.0 9.5 9.0 8.5 8.0 7.5 7.0 6.5 6.0 5.5 5.0 4.5 4.0 3.5 3.0 2.5 2.0 1.5 1.0 0.5
f1 (ppm)

Compound p3

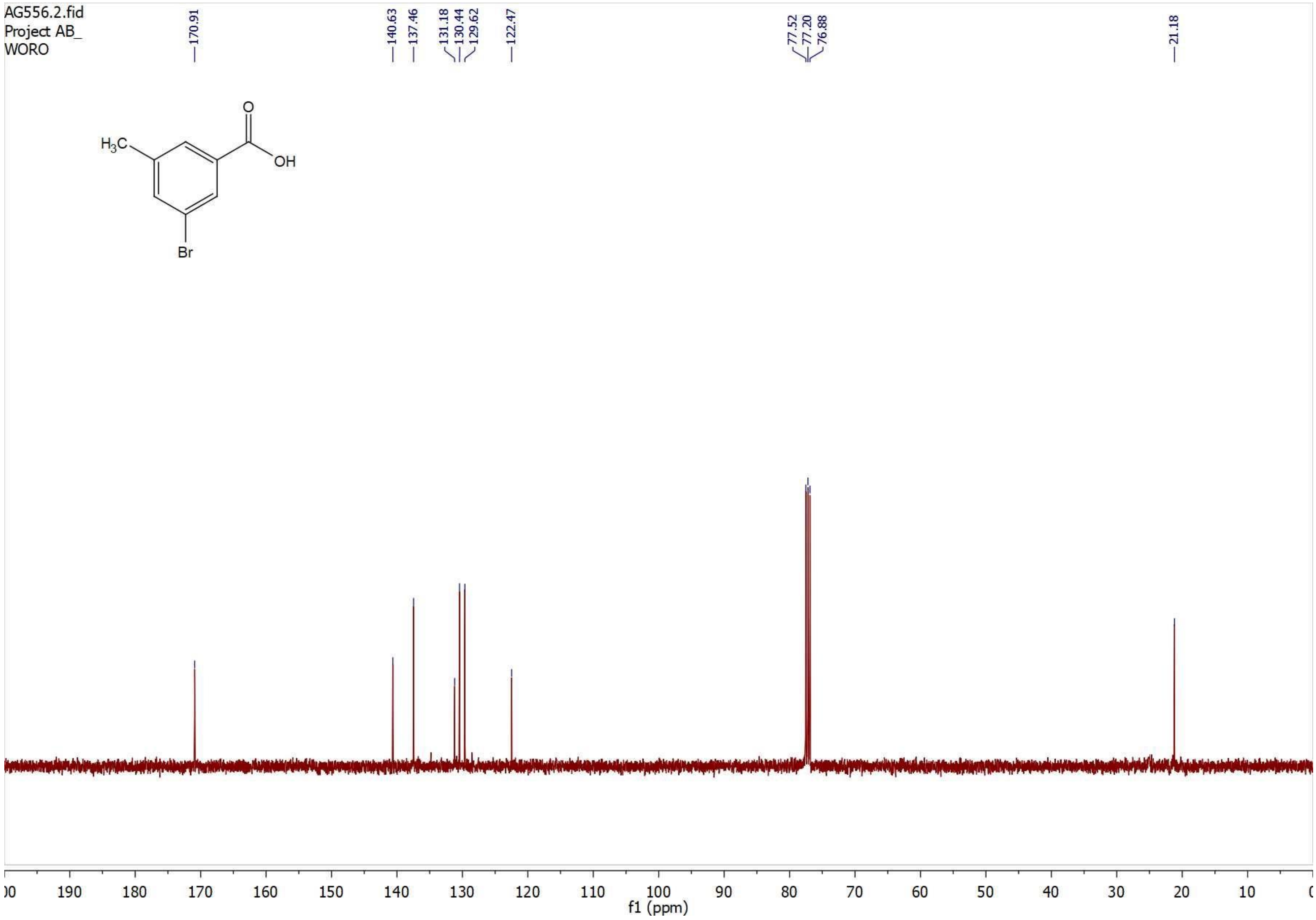

AG556.2.fid
Project AB_
WORO
170.91
140.63
137.46
131.18
130.44
129.62
122.47
77.52
77.20
76.88
21.18
H3C
O
OH
Br
f1 (ppm)

Compound p4

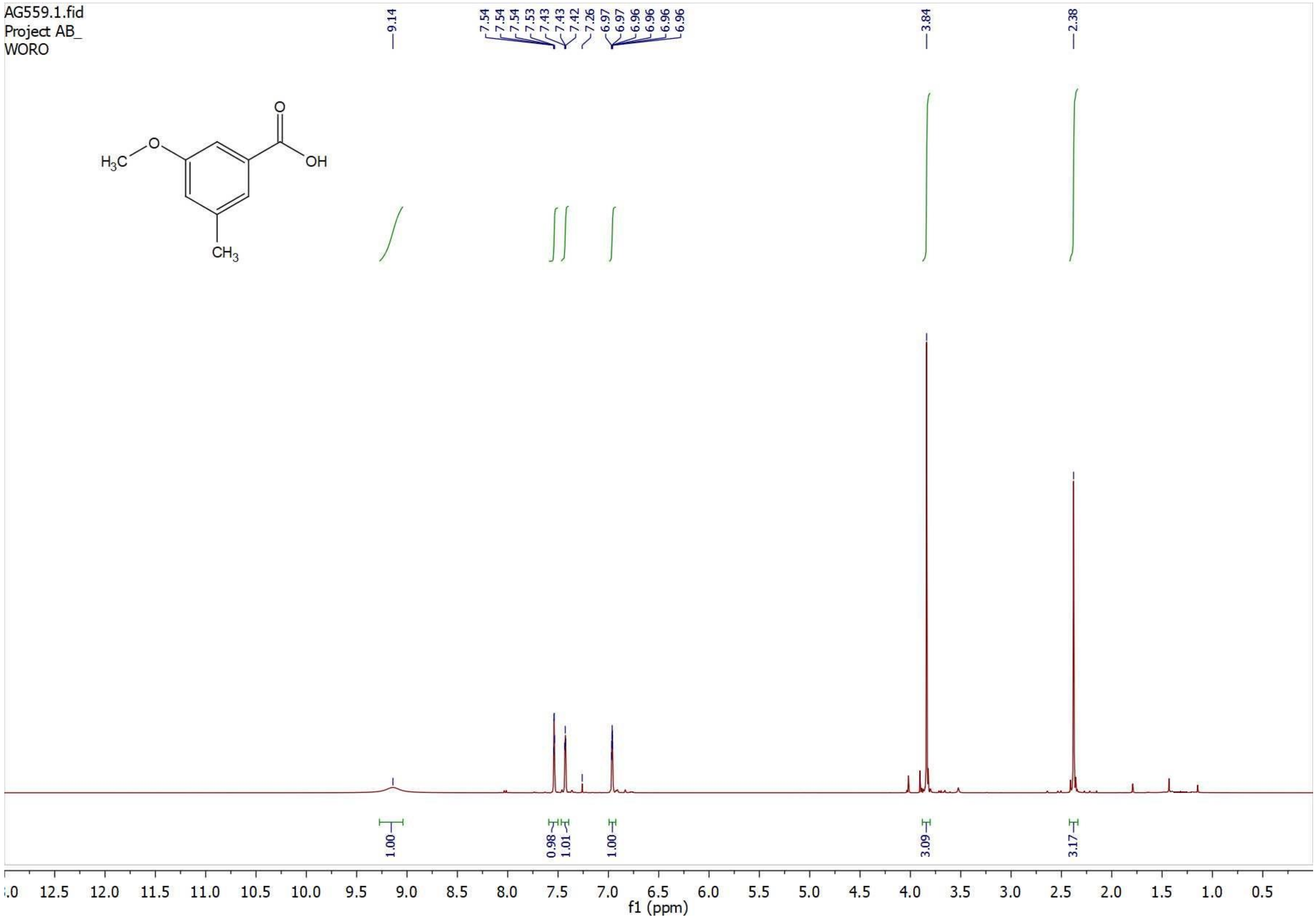
AG559.1.fid
Project AB_
WORO
9.14
7.54
7.54
7.54
7.53
7.43
7.43
7.42
7.26
6.97
6.97
6.96
6.96
6.96
6.96
3.84
2.38
H3C
O
OH
CH3
1.00
0.98
1.01
1.00
3.09
3.17
f1 (ppm)

Compound p4

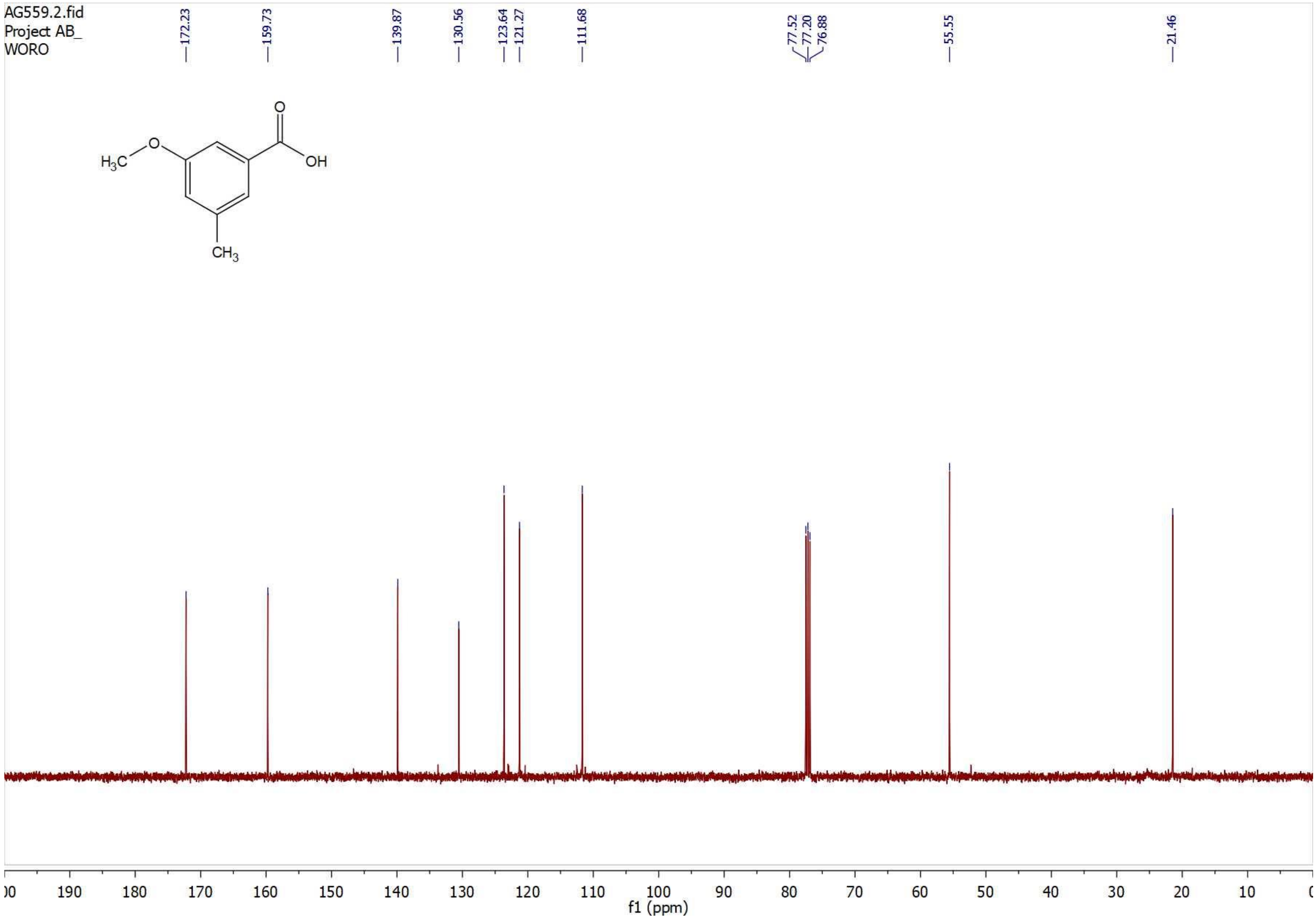

AG559.2.fid
Project AB_
WORO
172.23
159.73
139.87
130.56
123.64
121.27
111.68
77.52
77.20
76.88
55.55
21.46
O
OH
H_3C
O
CH_3
f1 (ppm)

Compound p5

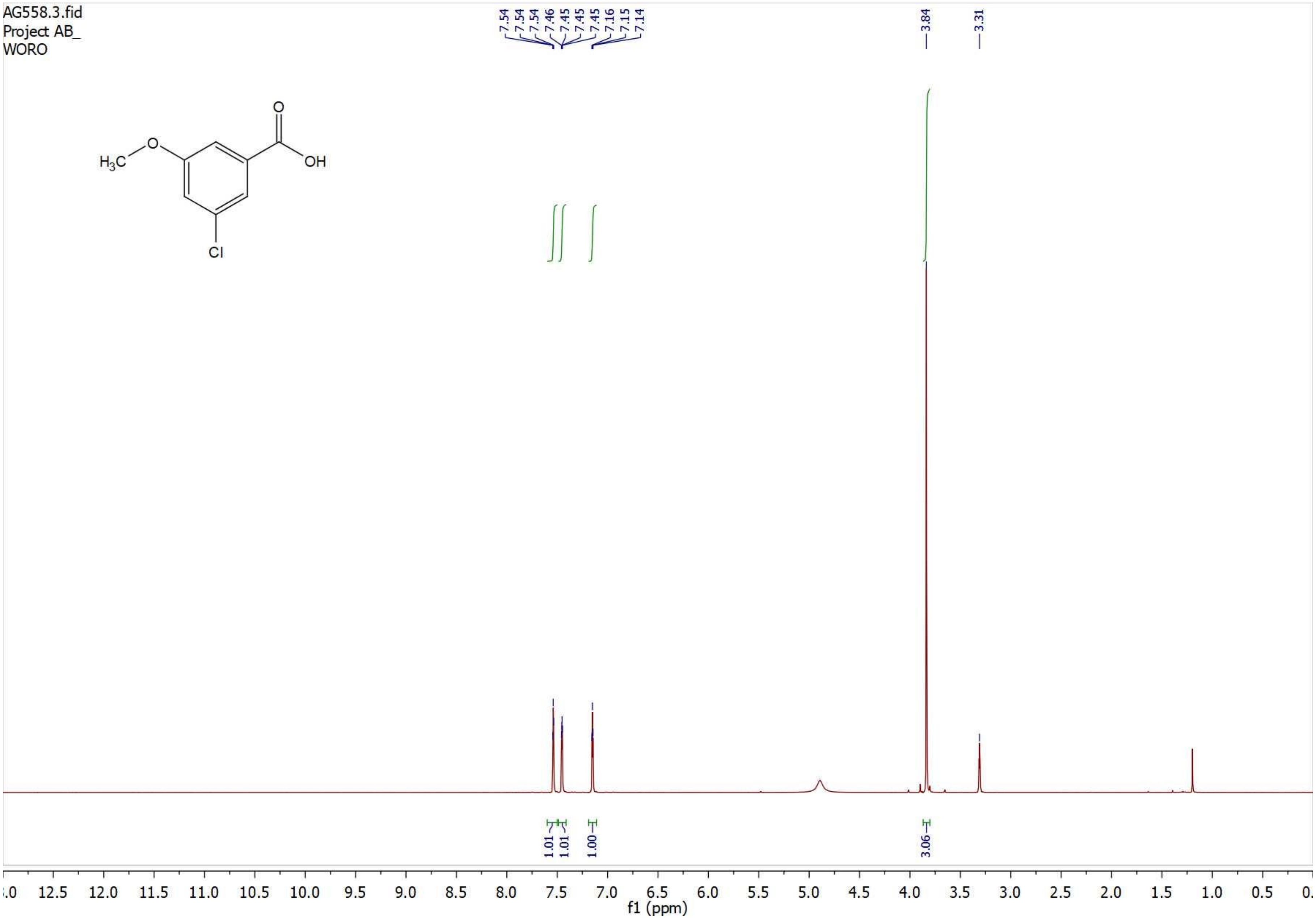
AG558.3.fid
Project AB_
WORO
7.54
7.54
7.54
7.46
7.45
7.45
7.45
7.16
7.15
7.14
3.84
3.31
O
OH
H3C
O
Cl
1.01
1.01
1.00
3.06
.0
12.5
12.0
11.5
11.0
10.5
10.0
9.5
9.0
8.5
8.0
7.5
7.0
6.5
6.0
5.5
5.0
4.5
4.0
3.5
3.0
2.5
2.0
1.5
1.0
0.5
0.
f1 (ppm)

Compound p5

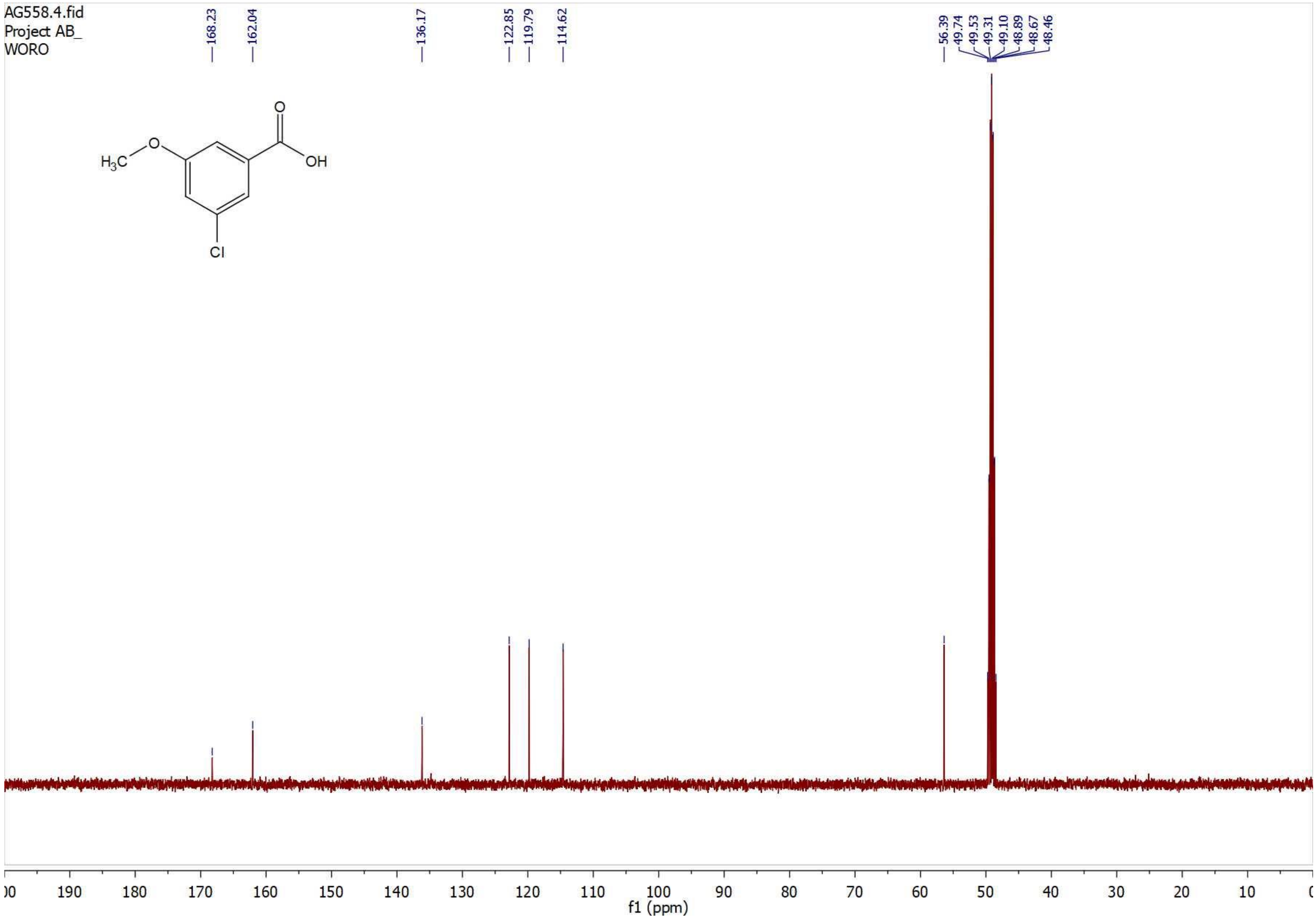
AG558.4.fid
Project AB_
WORO
168.23
162.04
136.17
122.85
119.79
114.62
56.39
49.74
49.53
49.31
49.10
48.89
48.67
48.46
O
OH
H3C
O
Cl
190
180
170
160
150
140
130
120
110
100
90
80
70
60
50
40
30
20
10
f1 (ppm)

Compound p6

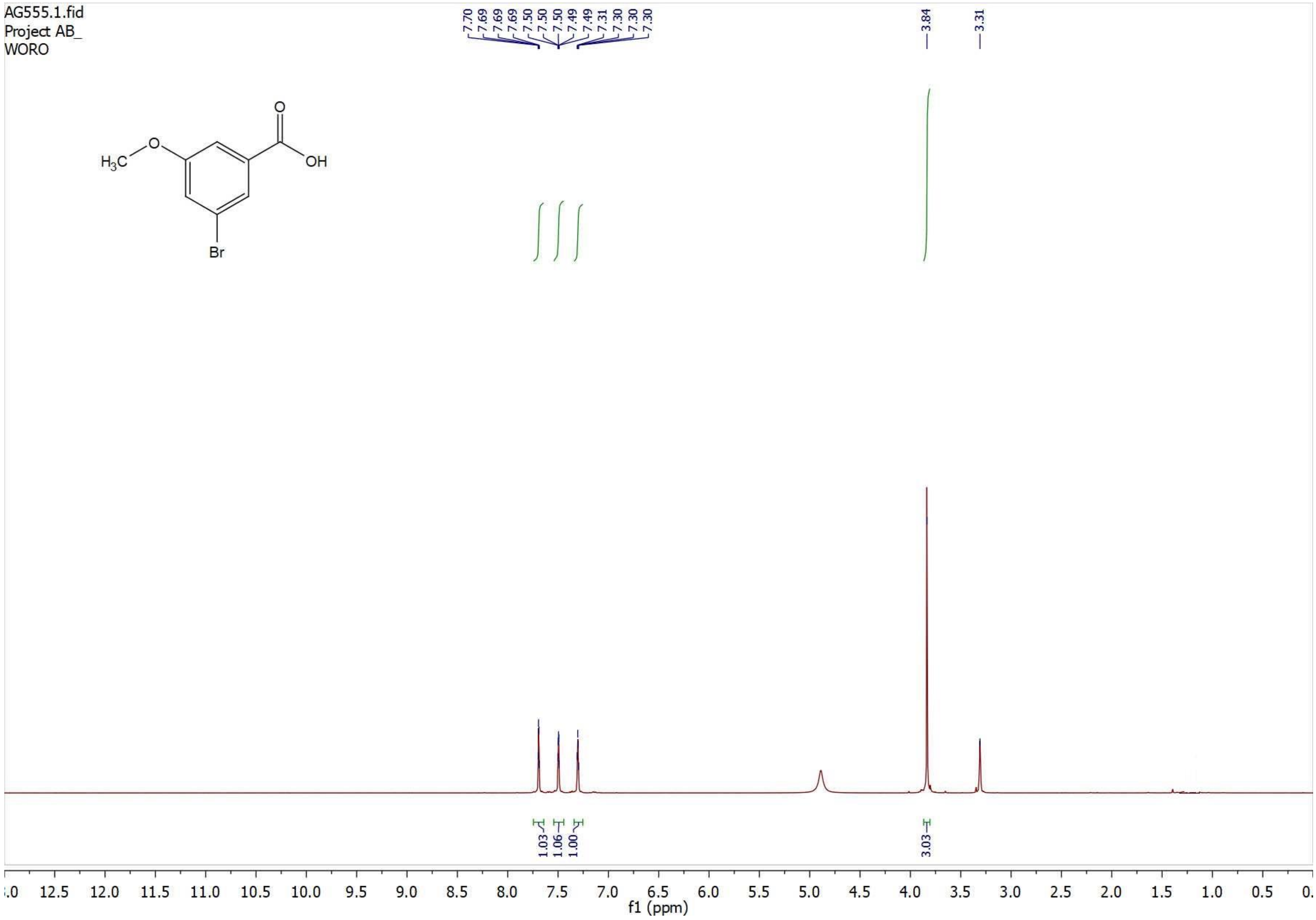
AG555.1.fid
Project AB_
WORO
7.70
7.69
7.69
7.69
7.50
7.50
7.50
7.49
7.49
7.31
7.30
7.30
7.30
3.84
3.31
H3C
O
O
OH
Br
1.03
1.06
1.00
3.03
12.5
12.0
11.5
11.0
10.5
10.0
9.5
9.0
8.5
8.0
7.5
7.0
6.5
6.0
5.5
5.0
4.5
4.0
3.5
3.0
2.5
2.0
1.5
1.0
0.5
f1 (ppm)

Compound p6

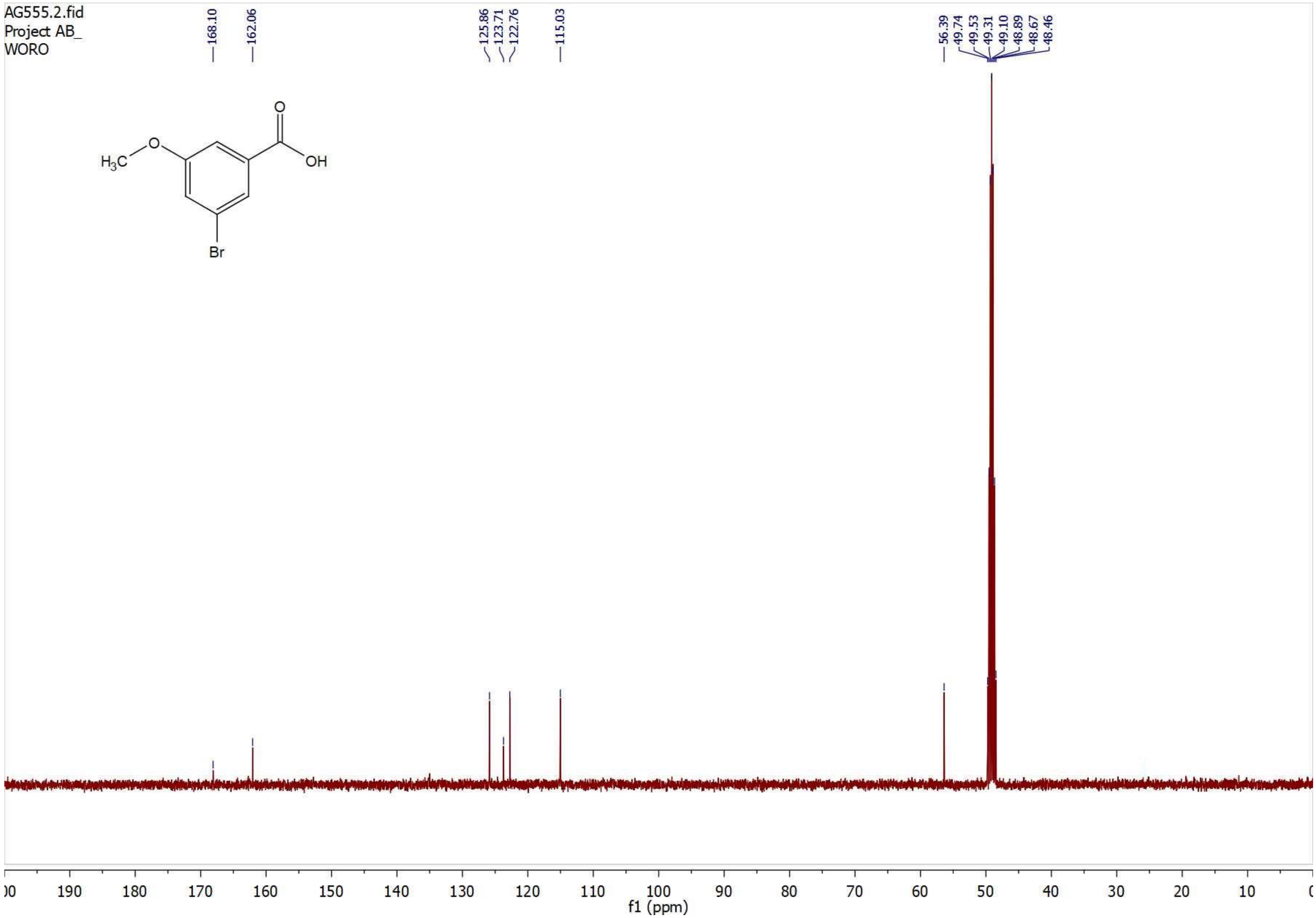
AG555.2.fid
Project AB_
WORO
168.10
162.06
125.86
123.71
122.76
115.03
56.39
49.74
49.53
49.31
49.10
48.89
48.67
48.46
H3C
O
OH
Br
00
190
180
170
160
150
140
130
120
110
100
90
80
70
60
50
40
30
20
10
f1 (ppm)

Compound p7

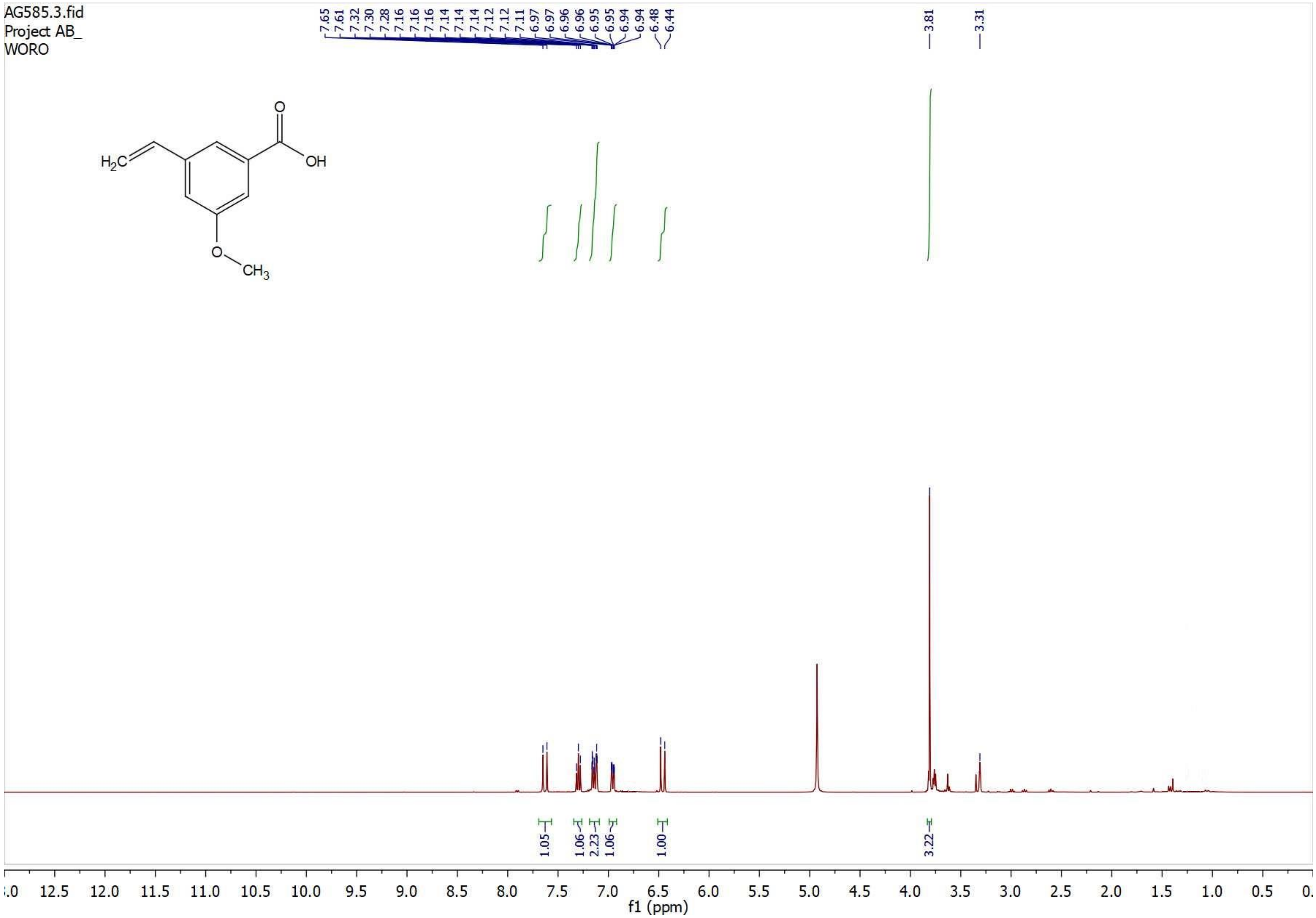
AG585.3.fid
Project AB_
WORO
7.65
7.61
7.32
7.30
7.28
7.16
7.16
7.16
7.14
7.14
7.14
7.12
7.12
7.11
6.97
6.97
6.96
6.96
6.95
6.95
6.94
6.94
6.48
6.44
3.81
3.31
O
OH
H_2C
O
CH_3
1.05
1.06
2.23
1.06
1.00
3.22
12.5
12.0
11.5
11.0
10.5
10.0
9.5
9.0
8.5
8.0
7.5
7.0
6.5
6.0
5.5
5.0
4.5
4.0
3.5
3.0
2.5
2.0
1.5
1.0
0.5
f1 (ppm)

Compound p7

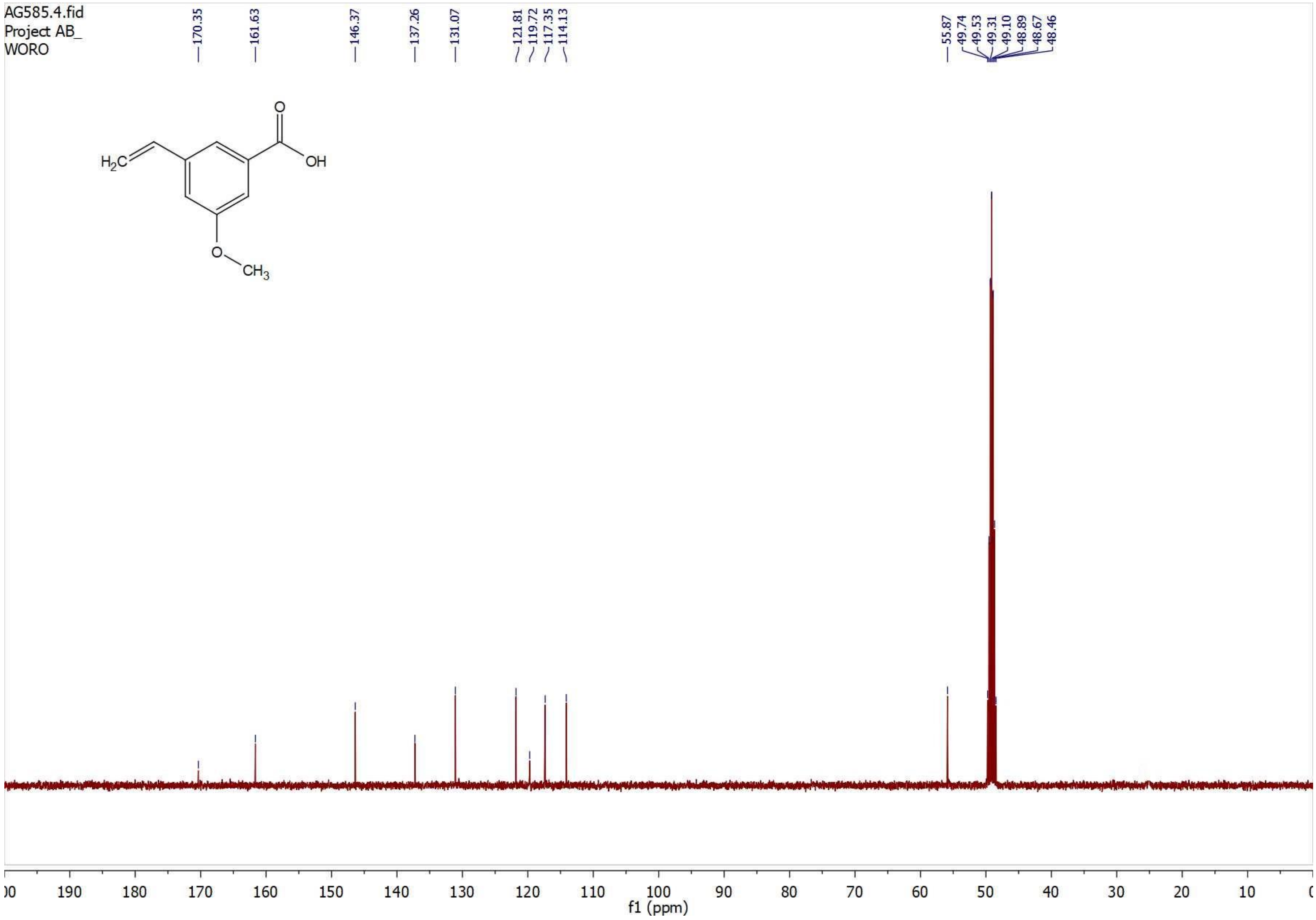

AG585.4.fid
Project AB_
WORO
170.35
161.63
146.37
137.26
131.07
121.81
119.72
117.35
114.13
55.87
49.74
49.53
49.31
49.10
48.89
48.67
48.46
H2C
O
OH
O
CH3
f1 (ppm)

Compound p8

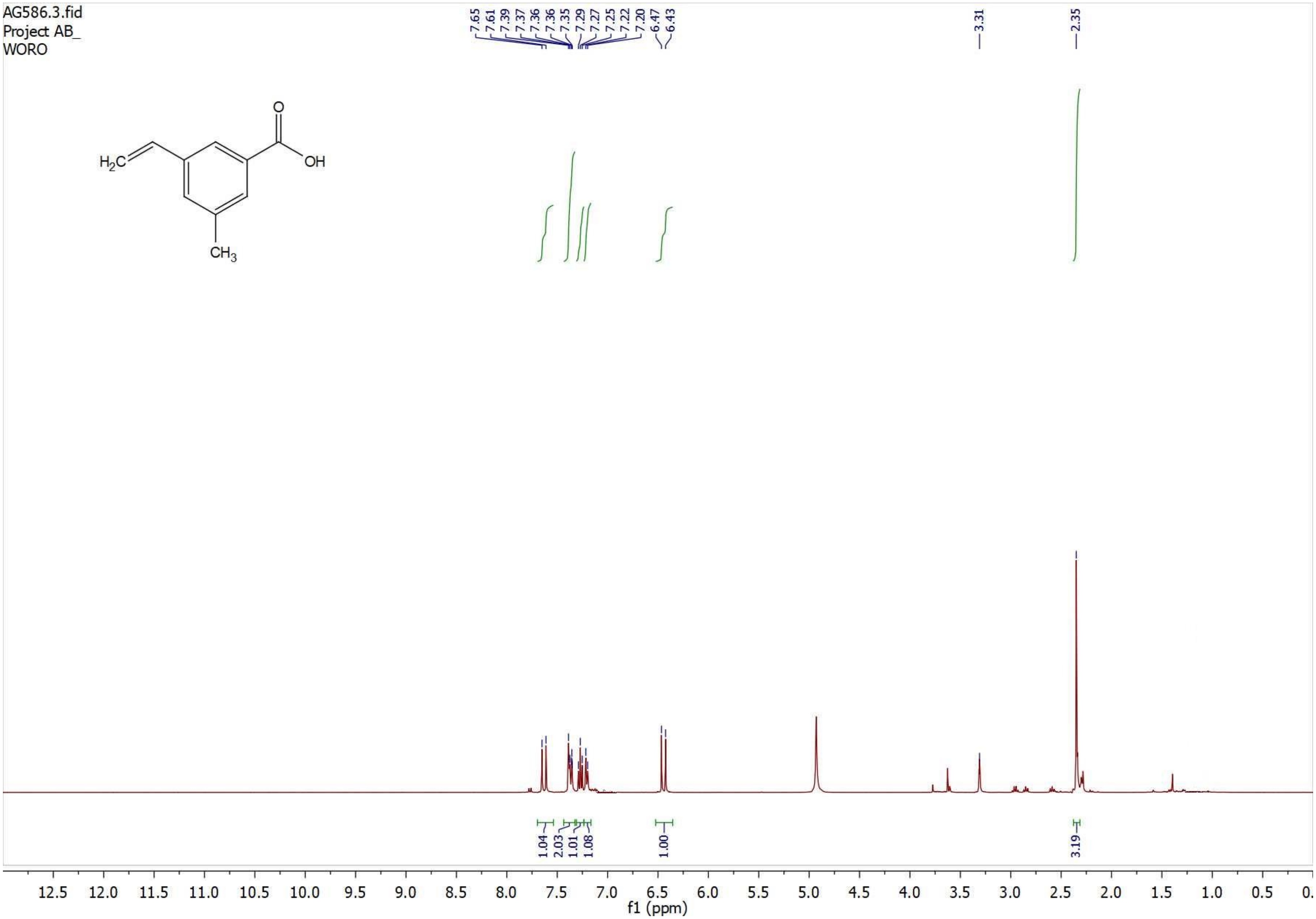
AG586.3.fid
Project AB_
WORO
7.65
7.61
7.39
7.37
7.36
7.36
7.35
7.29
7.27
7.25
7.22
7.20
6.47
6.43
3.31
2.35
H2C
O
OH
CH3
1.04
2.03
1.01
1.08
1.00
3.19
12.5
12.0
11.5
11.0
10.5
10.0
9.5
9.0
8.5
8.0
7.5
7.0
6.5
6.0
5.5
5.0
4.5
4.0
3.5
3.0
2.5
2.0
1.5
1.0
0.5
0.
f1 (ppm)

Compound p8

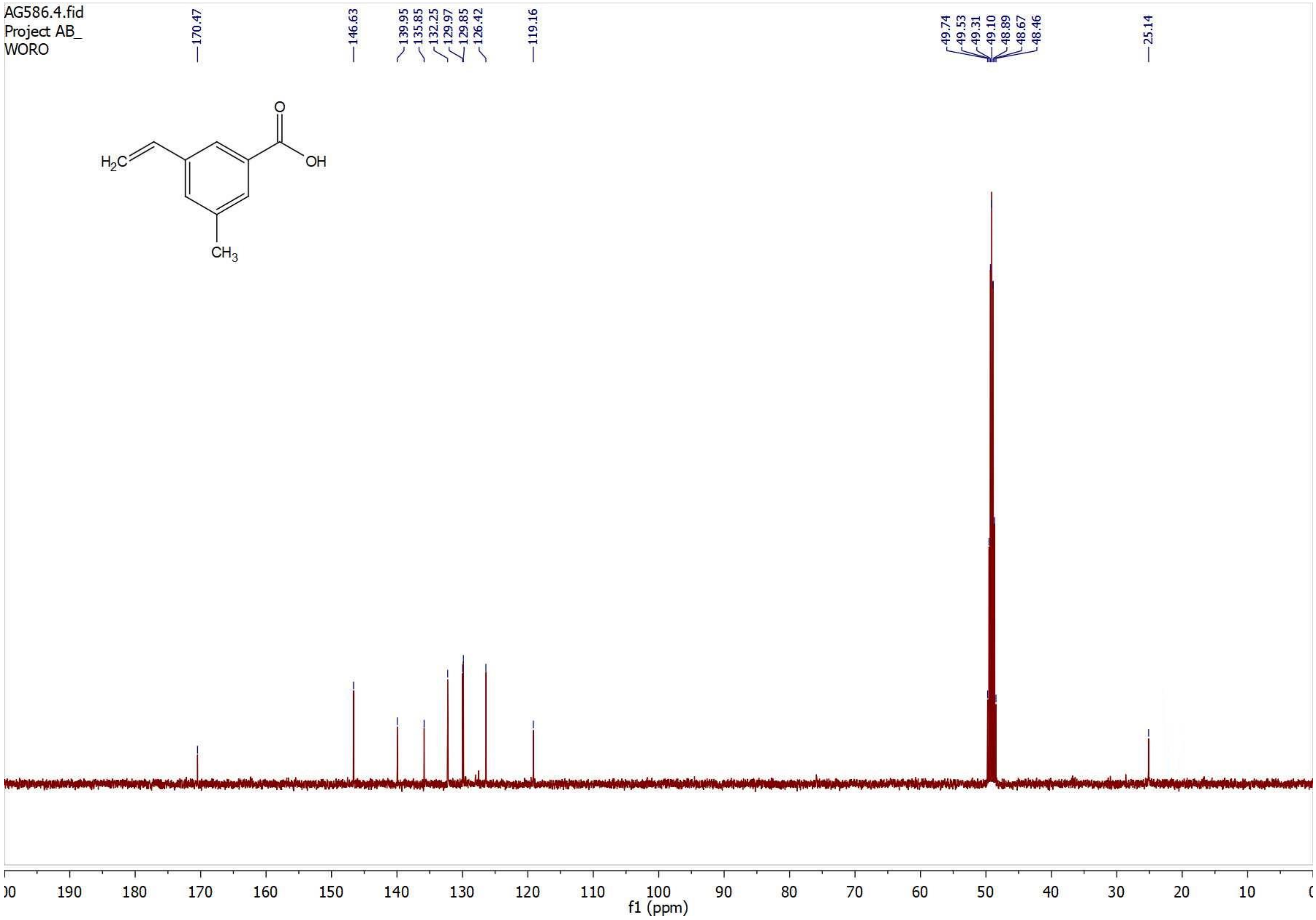
AG586.4.fid
Project AB_
WORO
170.47
146.63
139.95
135.85
132.25
129.97
129.85
126.42
119.16
49.74
49.53
49.31
49.10
48.89
48.67
48.46
25.14
O
OH
H_2C
CH_3
f1 (ppm)

Compound p9

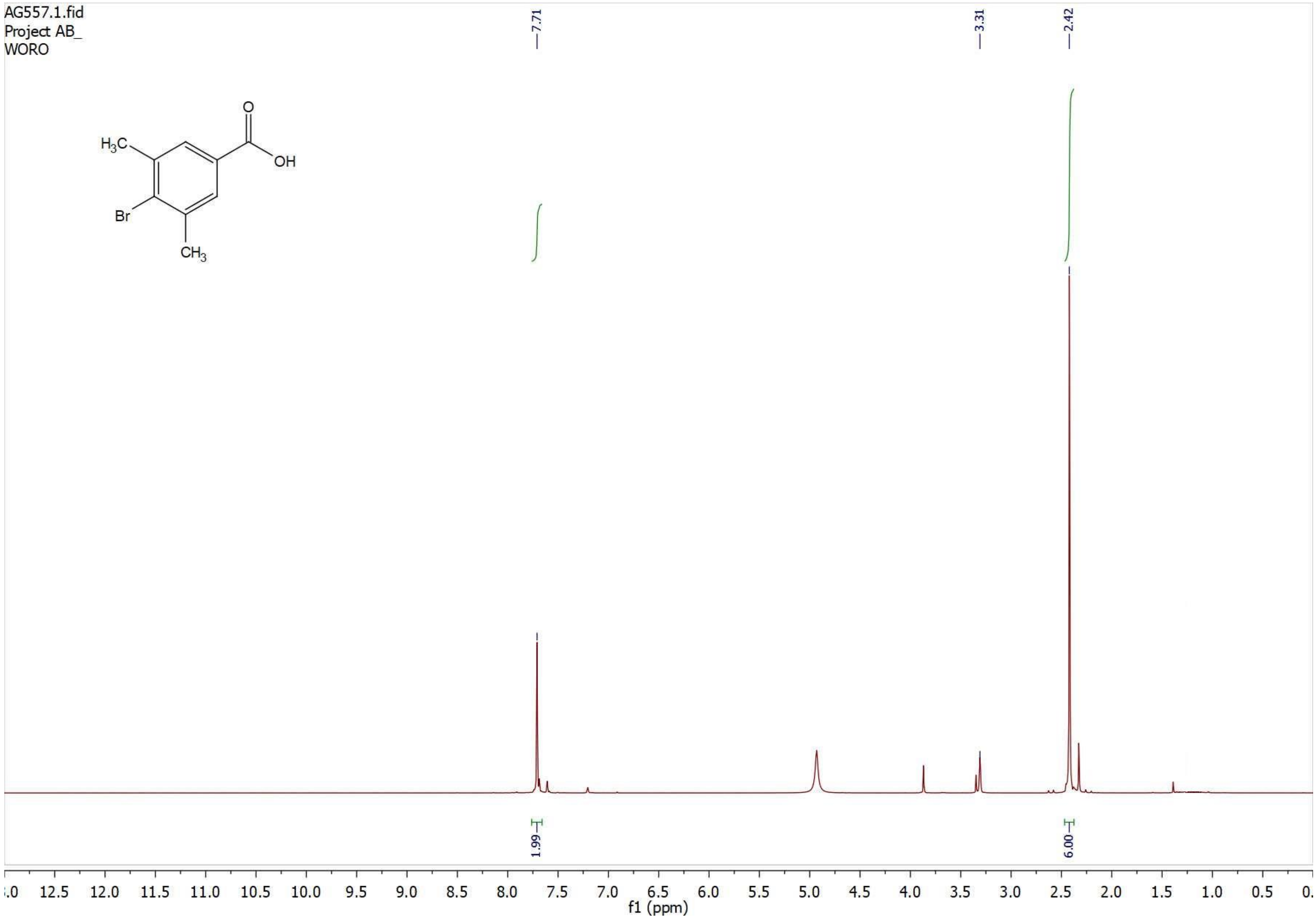

AG557.1.fid
Project AB_
WORO
7.71
3.31
2.42
H3C
OH
O
Br
CH3
1.99
6.00
f1 (ppm)

Compound p9

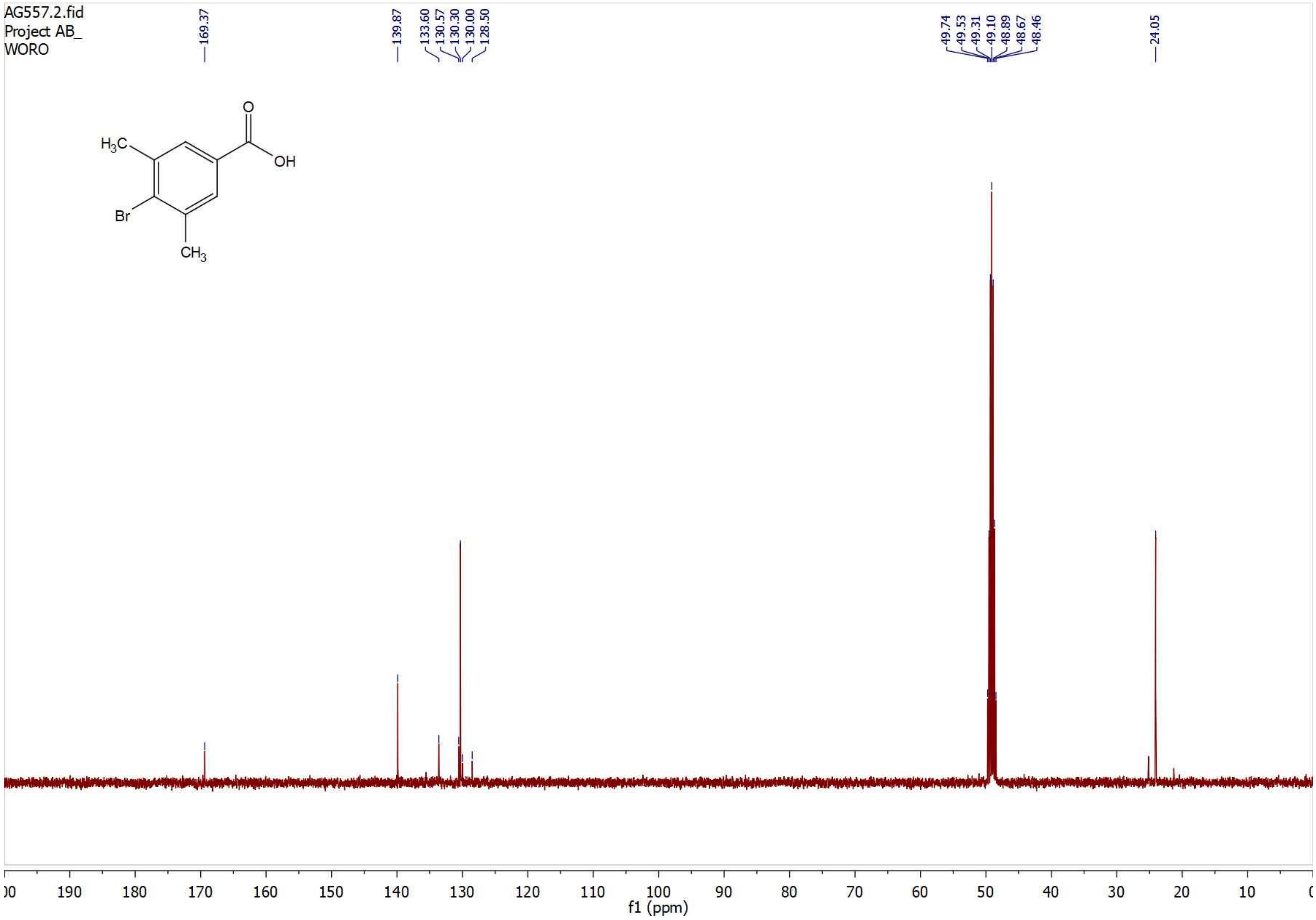

AG557.2.fid
Project AB_
WORO
169.37
139.87
133.60
130.57
130.30
130.00
128.50
49.74
49.53
49.31
49.10
48.89
48.67
48.46
24.05
O
OH
H3C
Br
CH3
00
190
180
170
160
150
140
130
120
110
100
90
80
70
60
50
40
30
20
10
f1 (ppm)

Compound p10

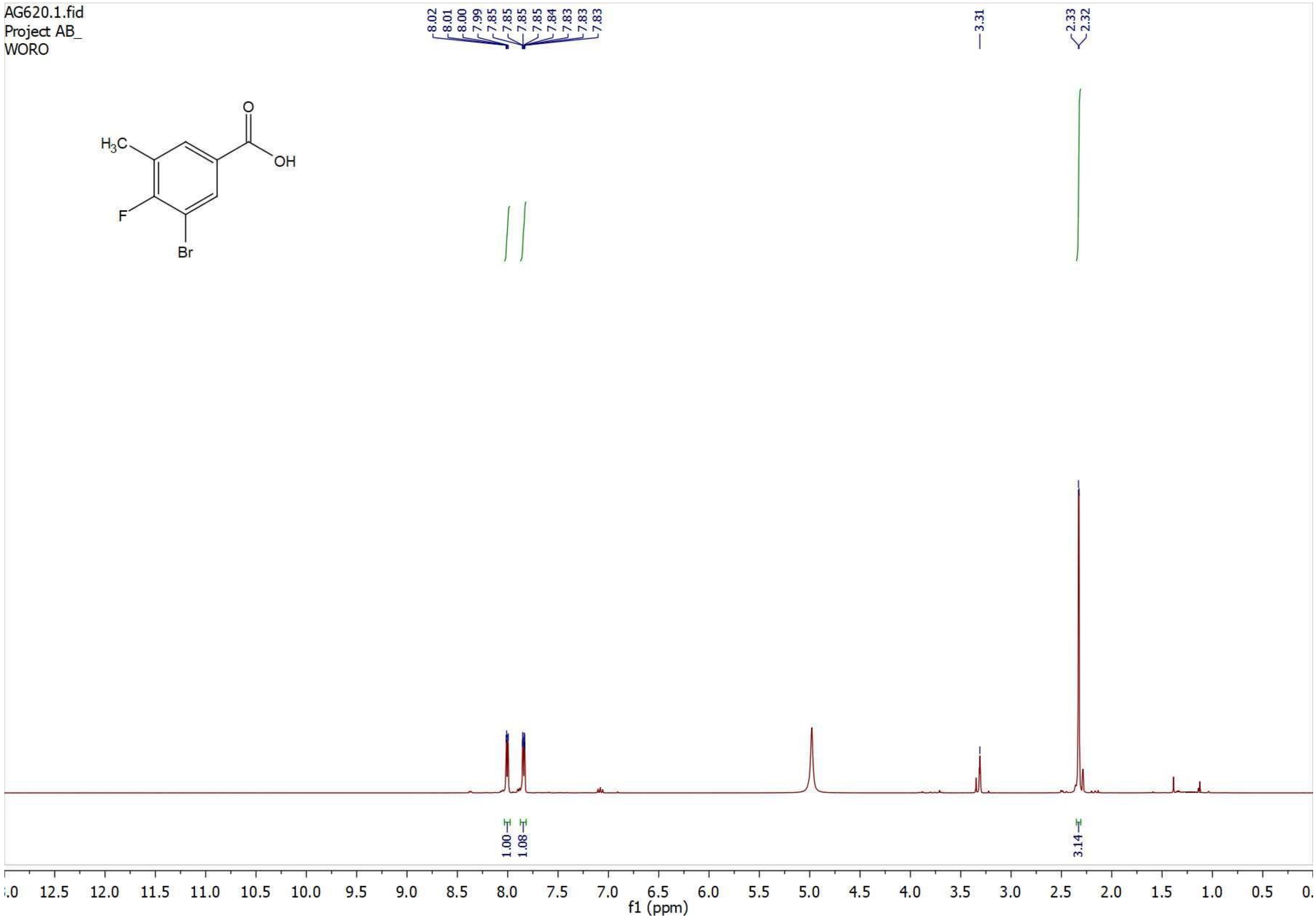

Compound p10

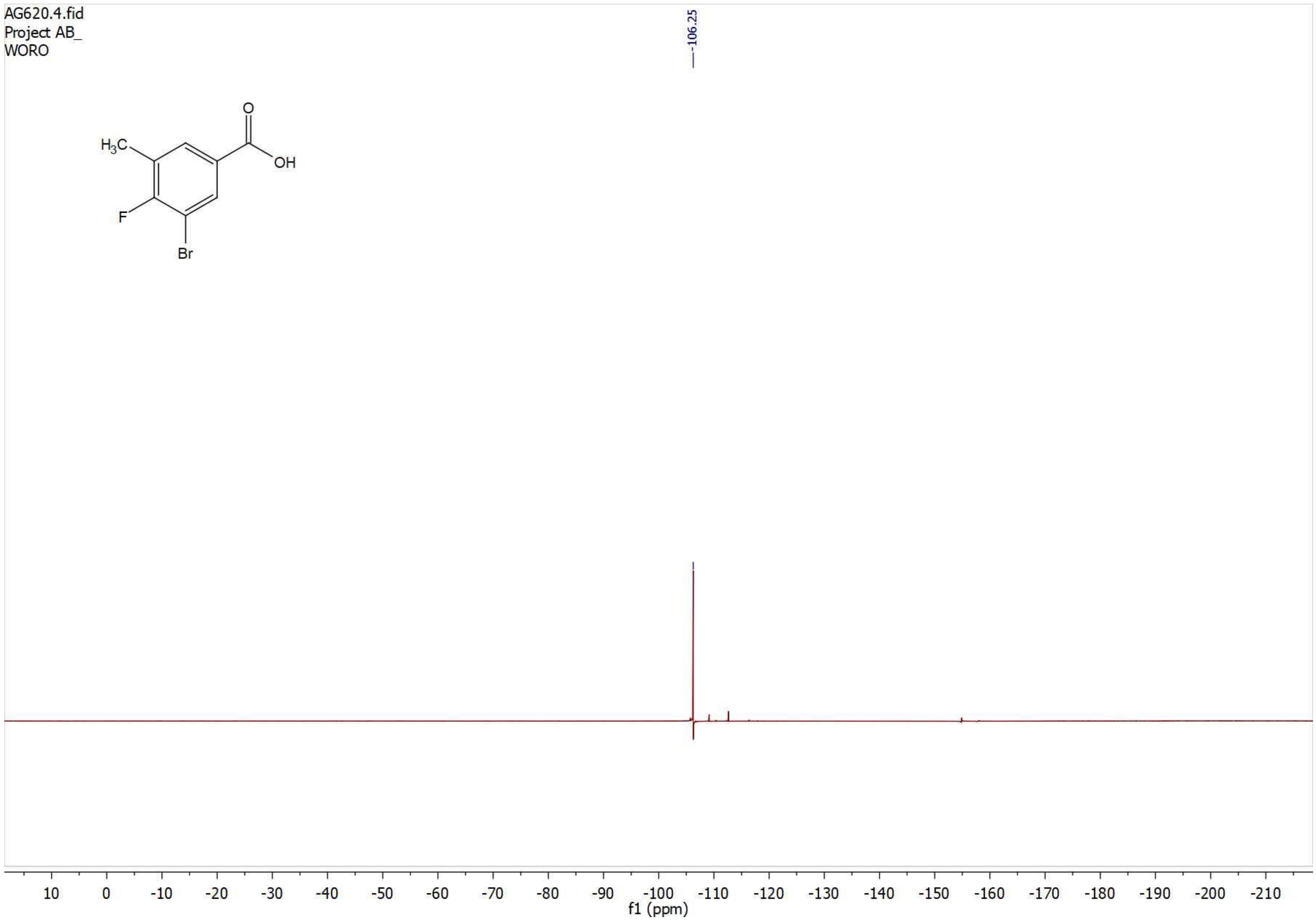

Compound p10

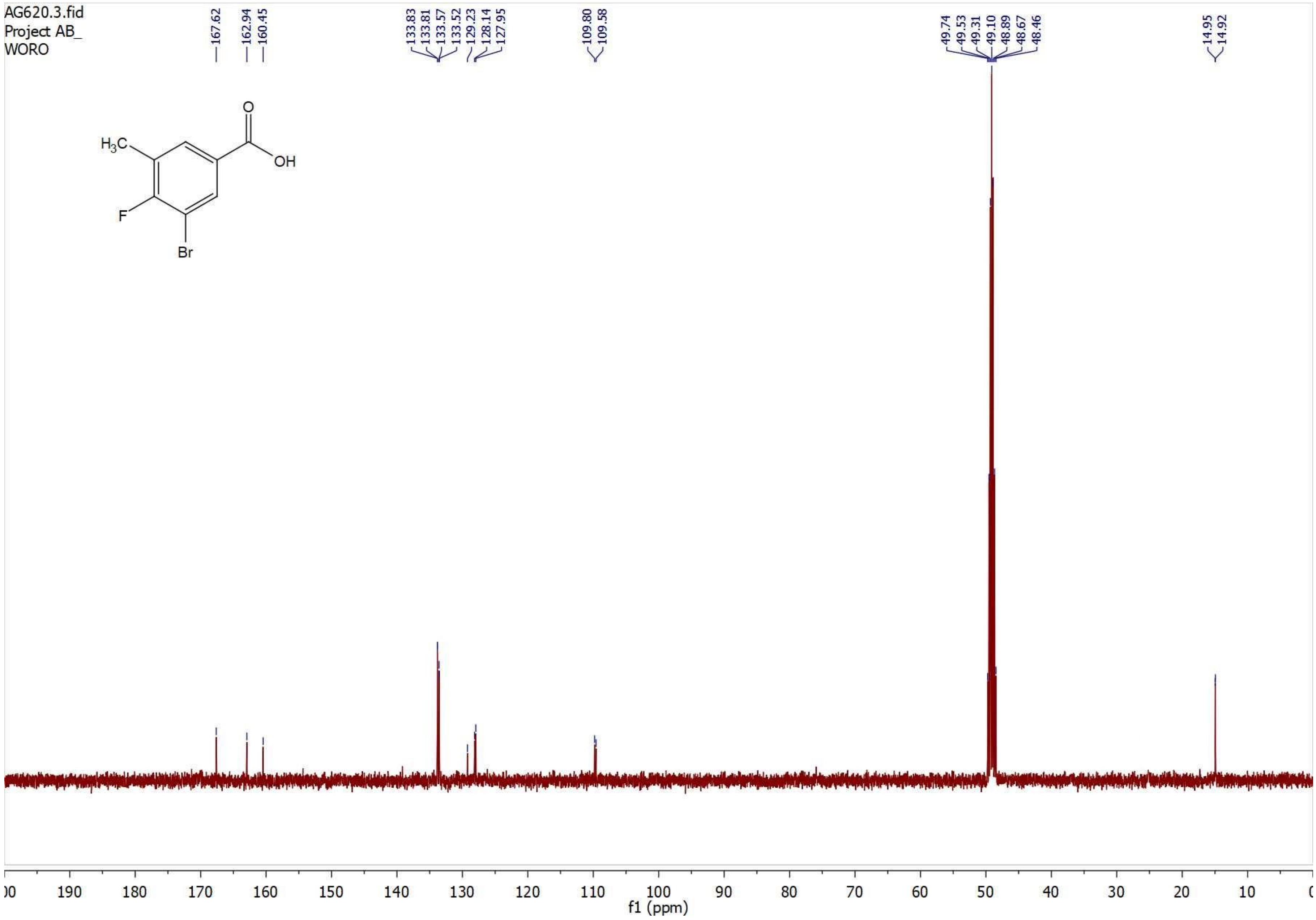

Compound p11

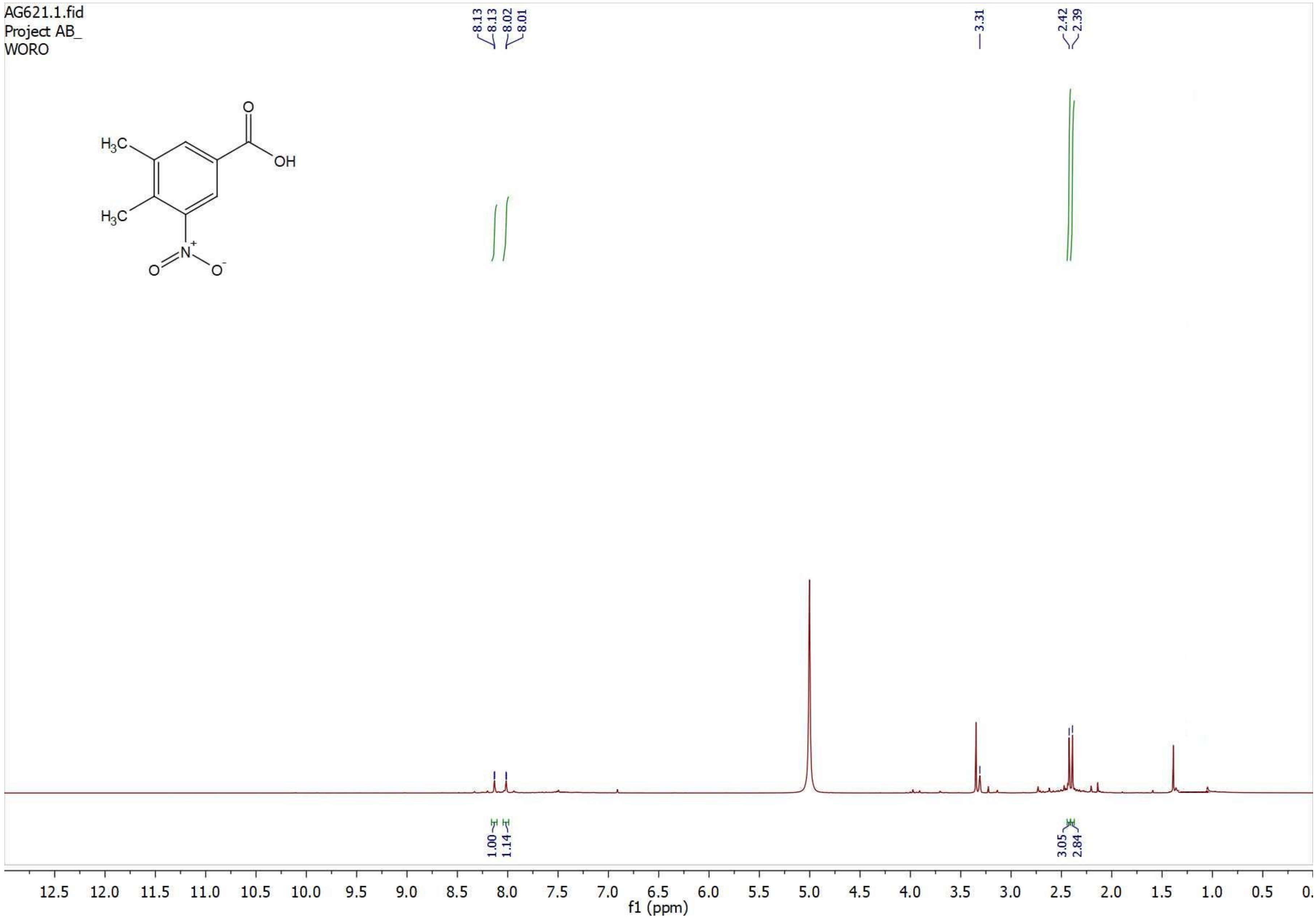

Compound p11

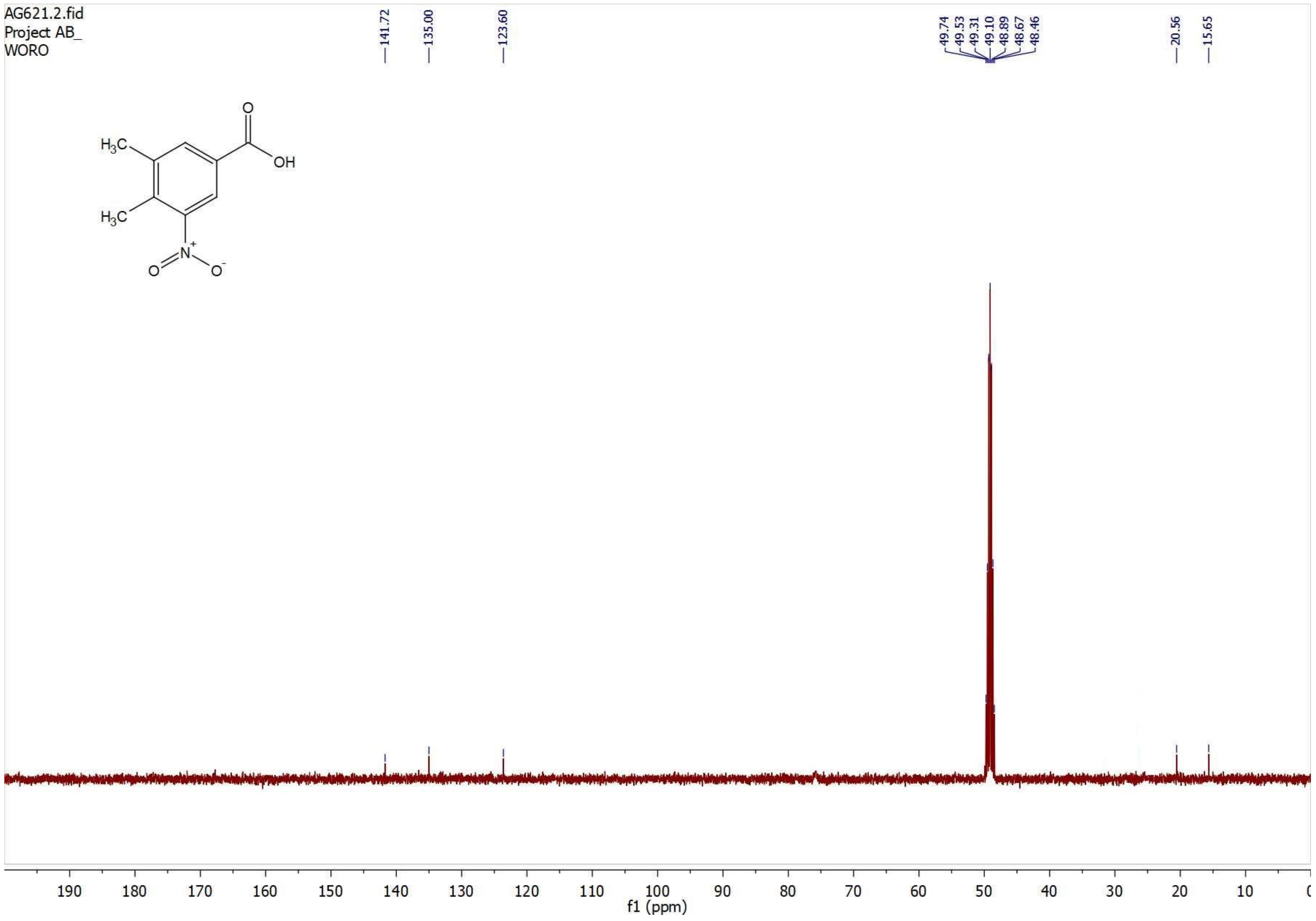

Compound p12

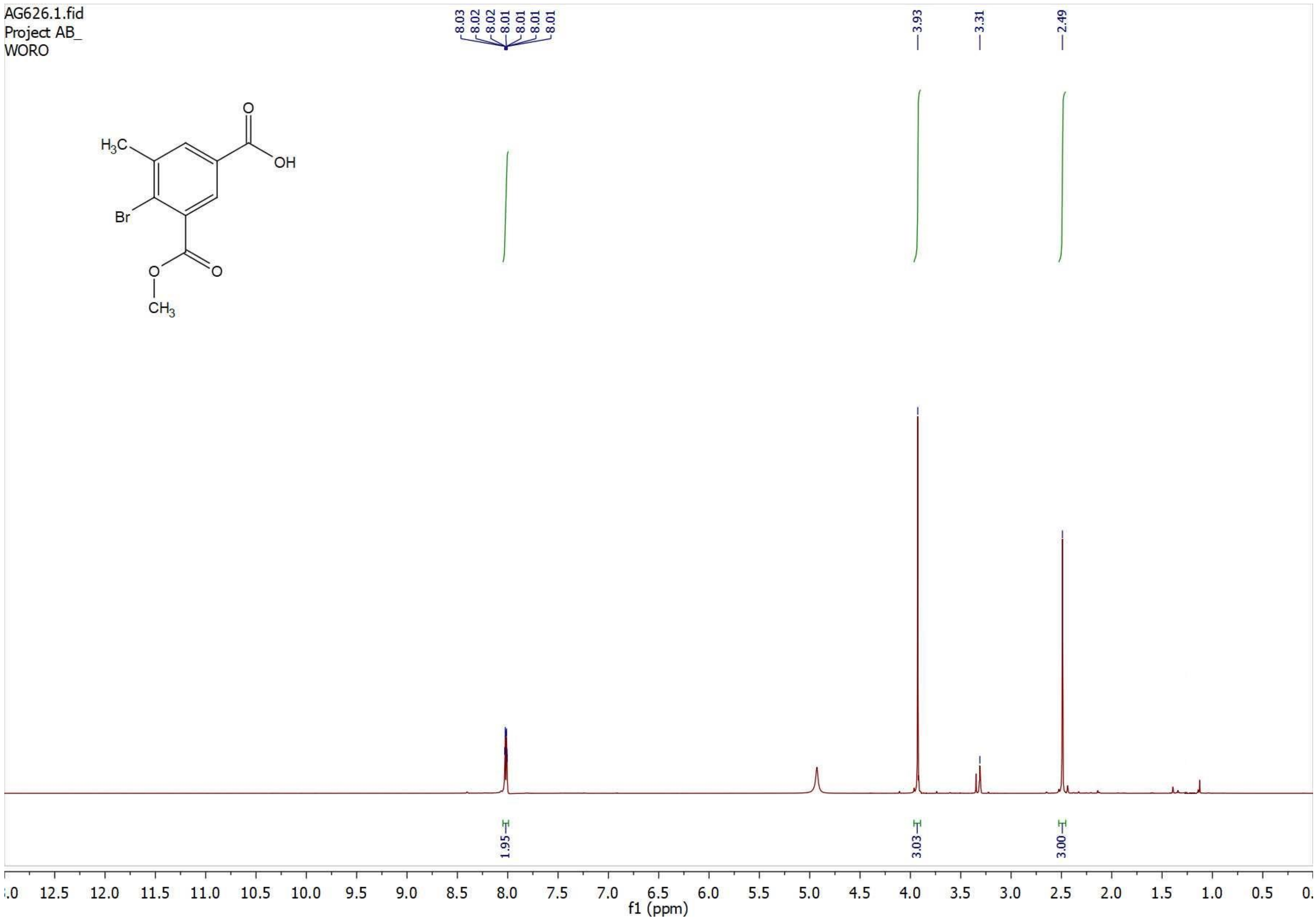

Compound p12

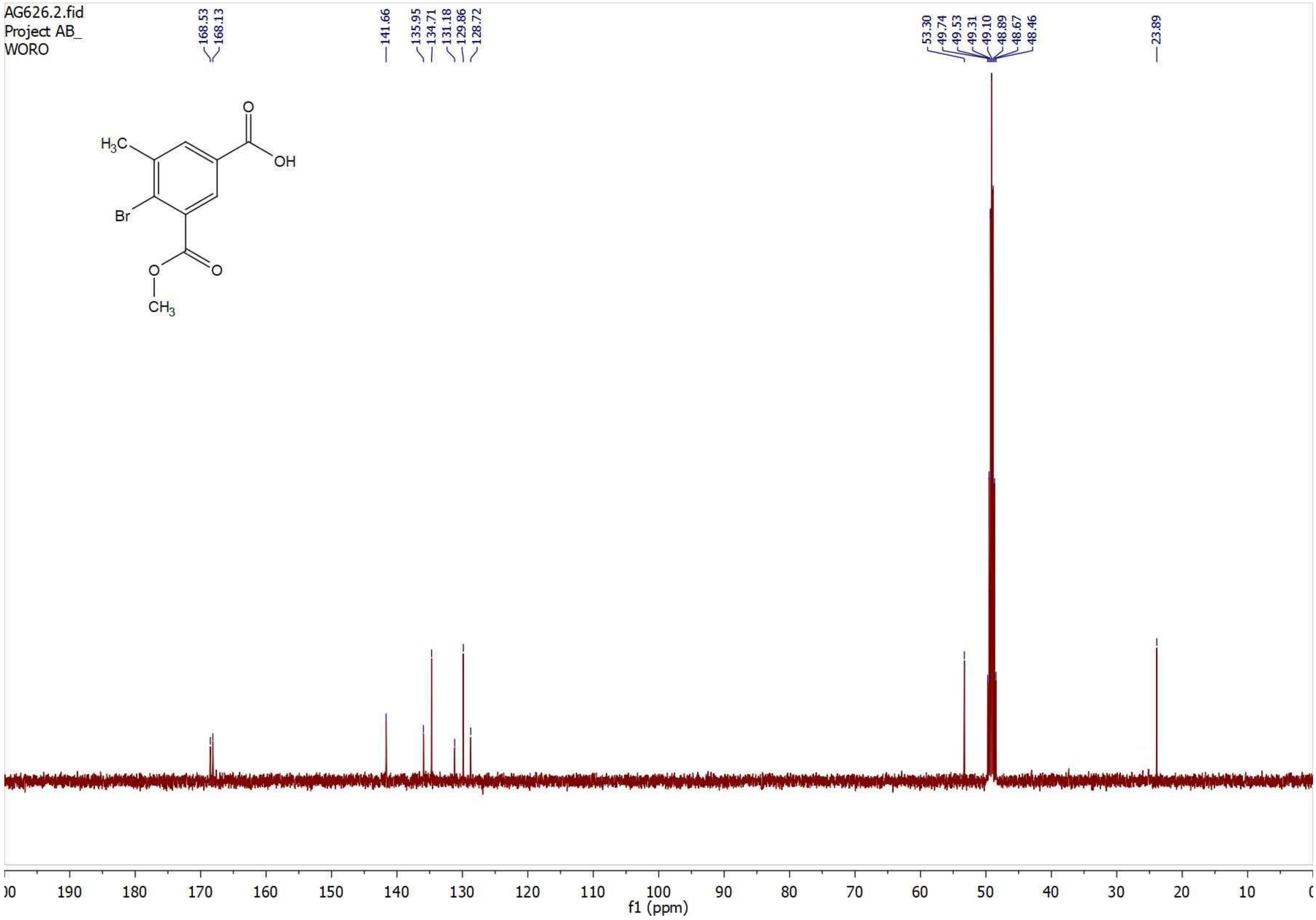

Compound p13

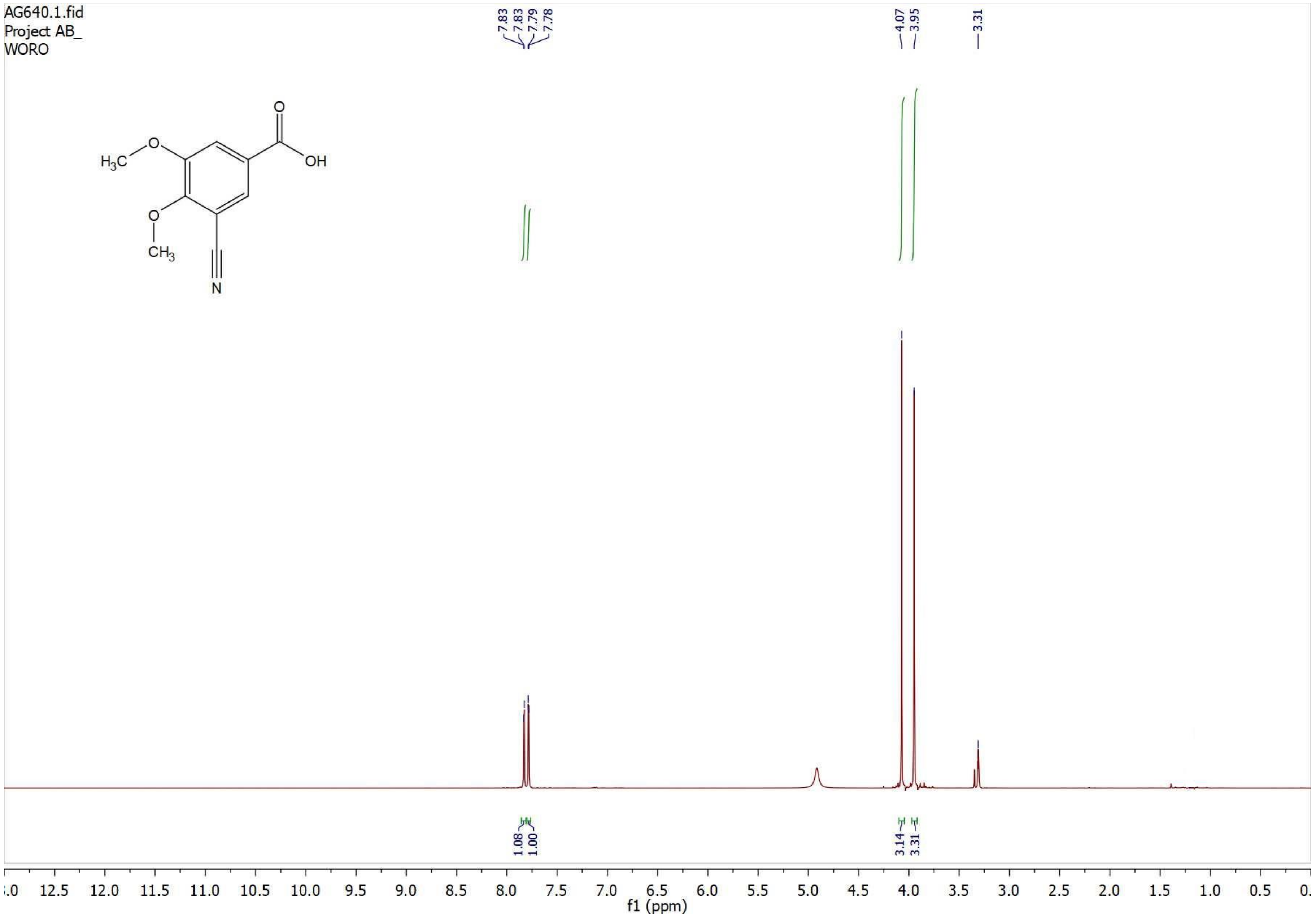

Compound p13

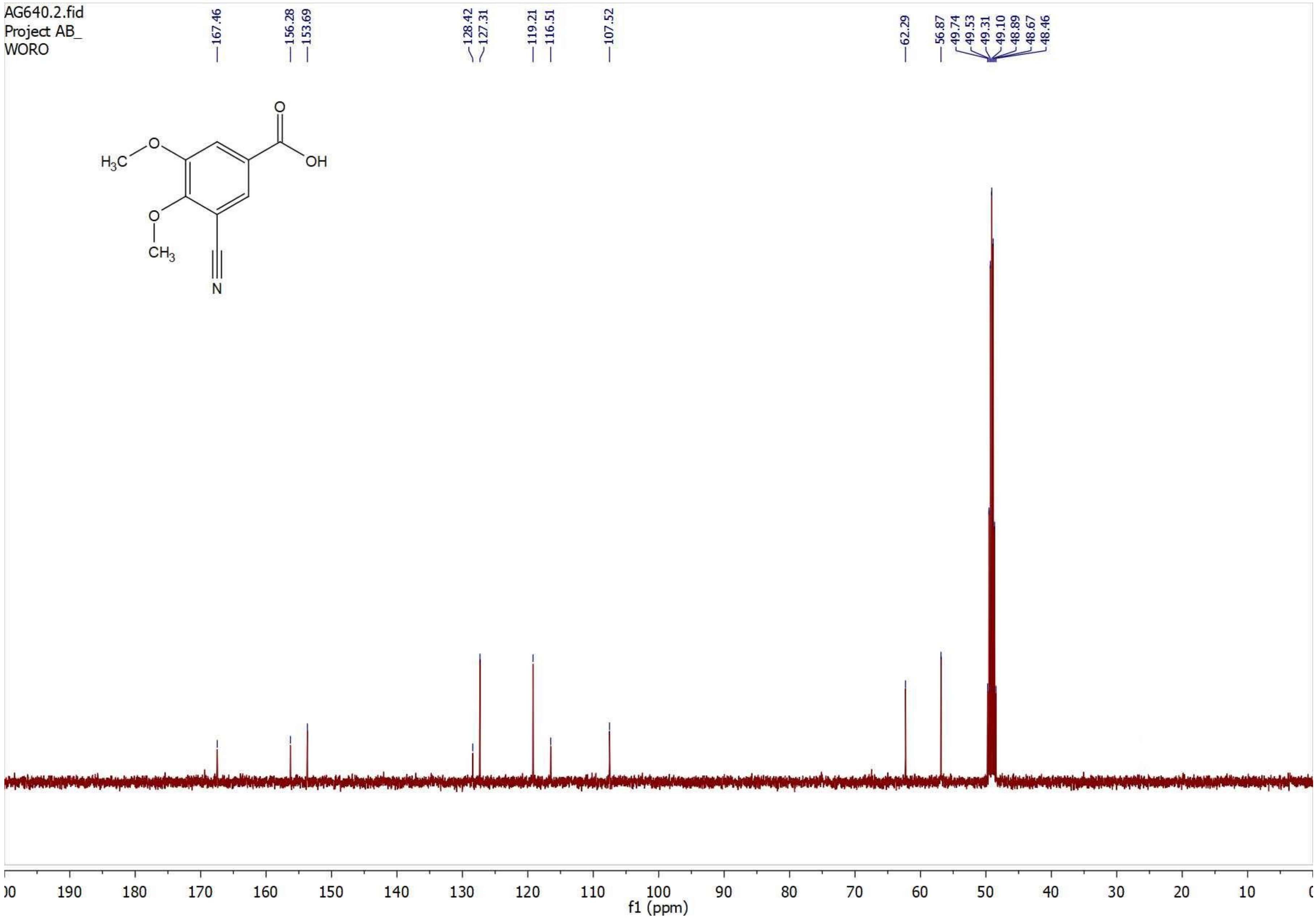

Compound p14

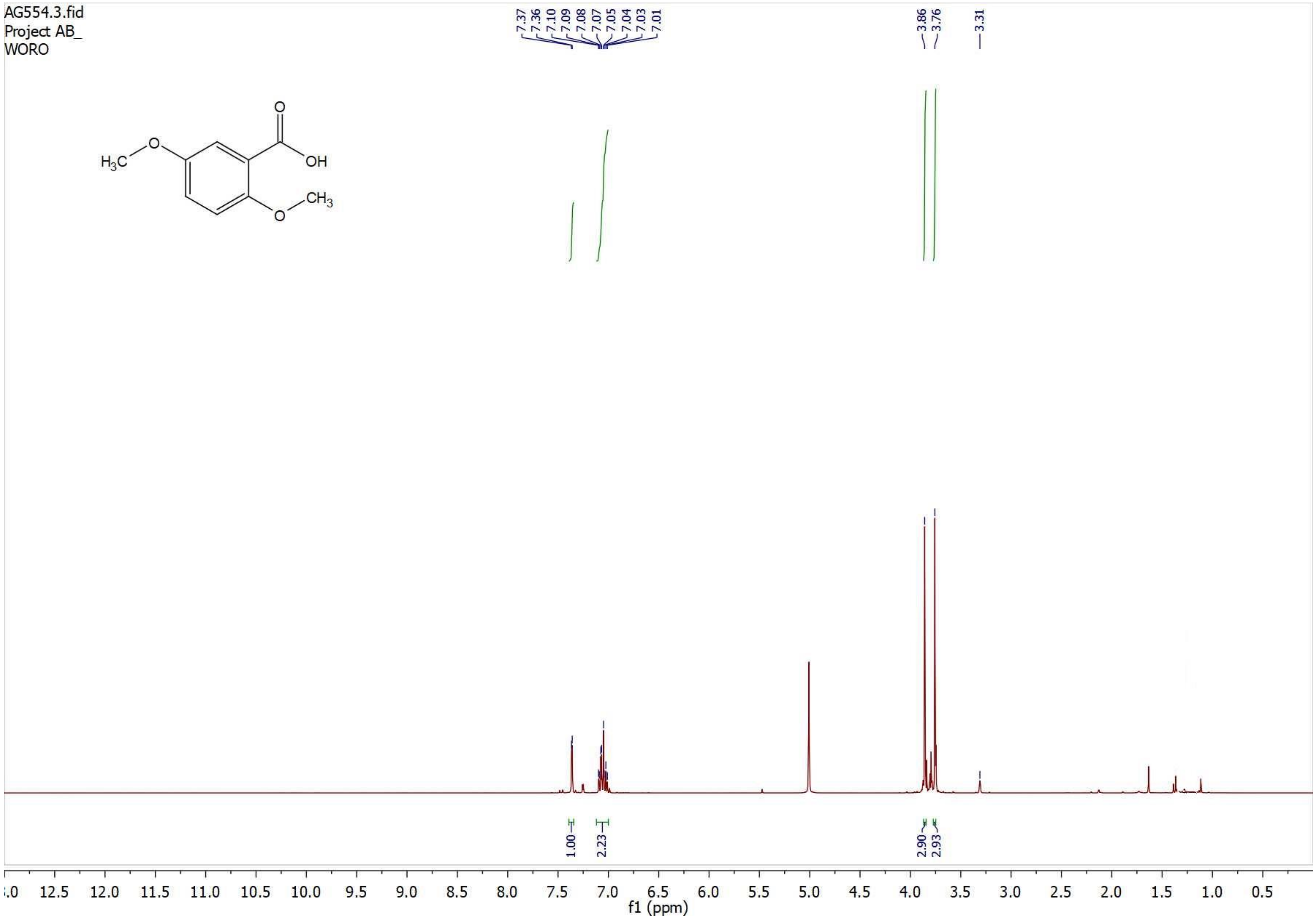

Compound p14

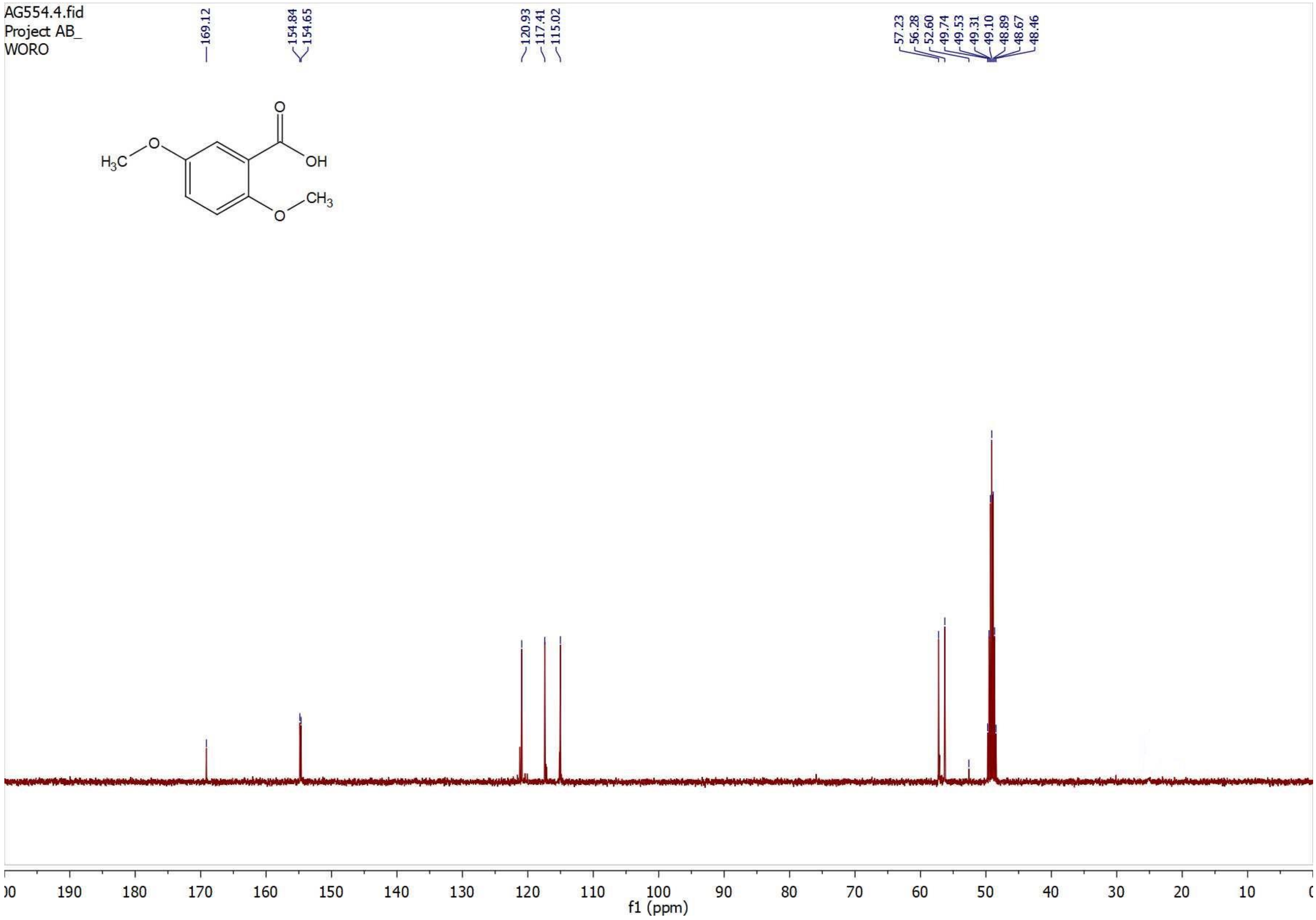

Compound p15

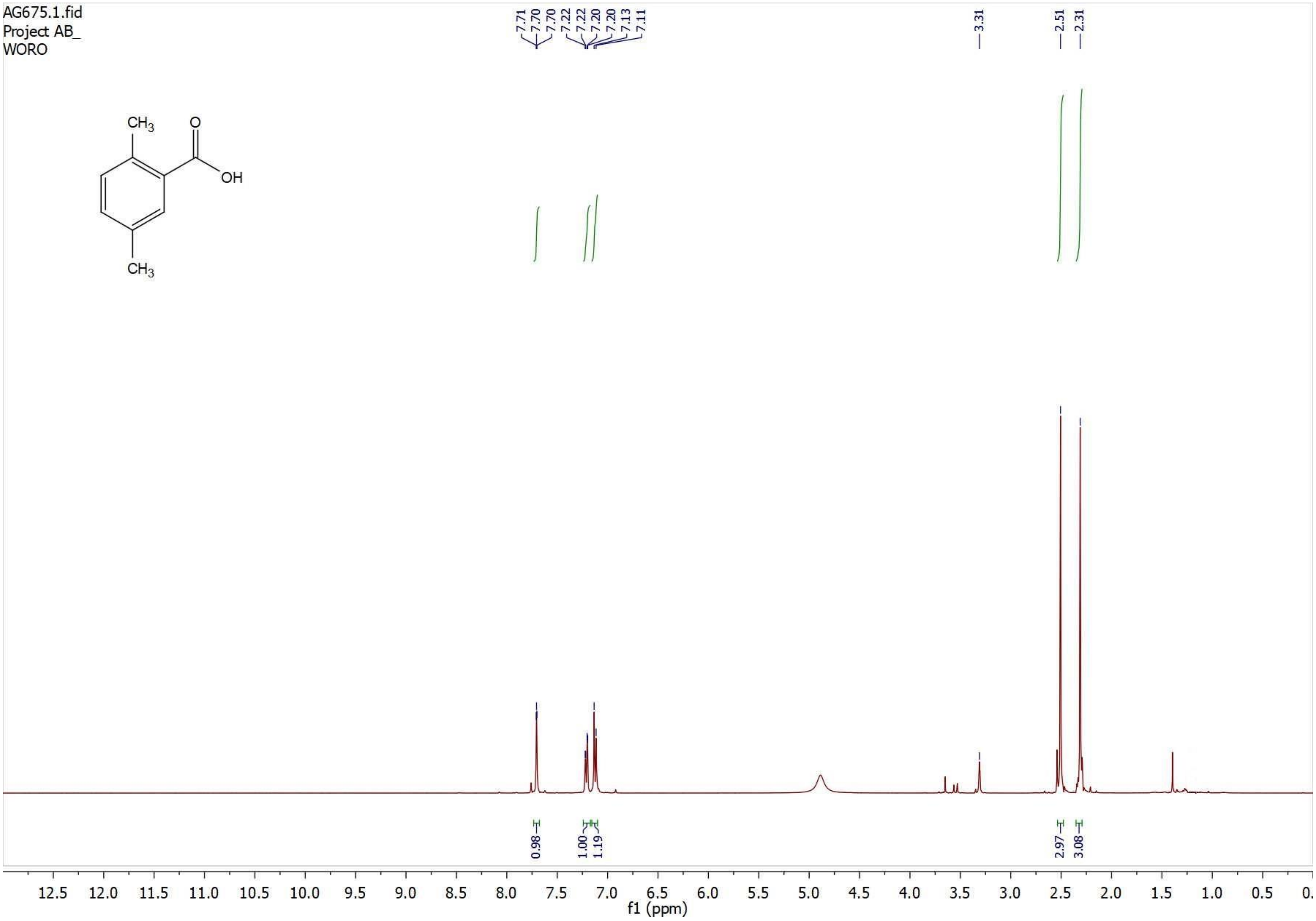

Compound p15

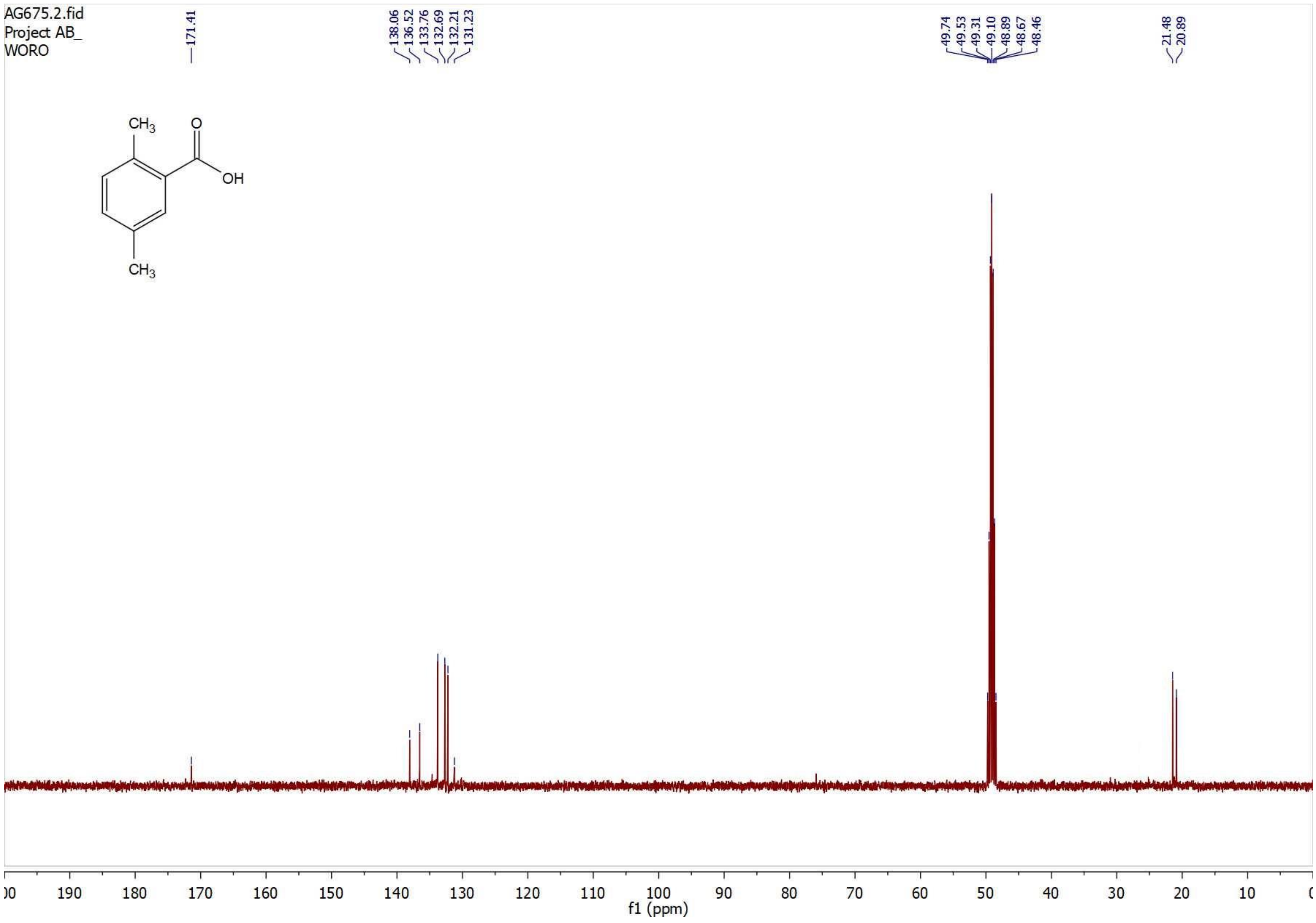

Compound p16

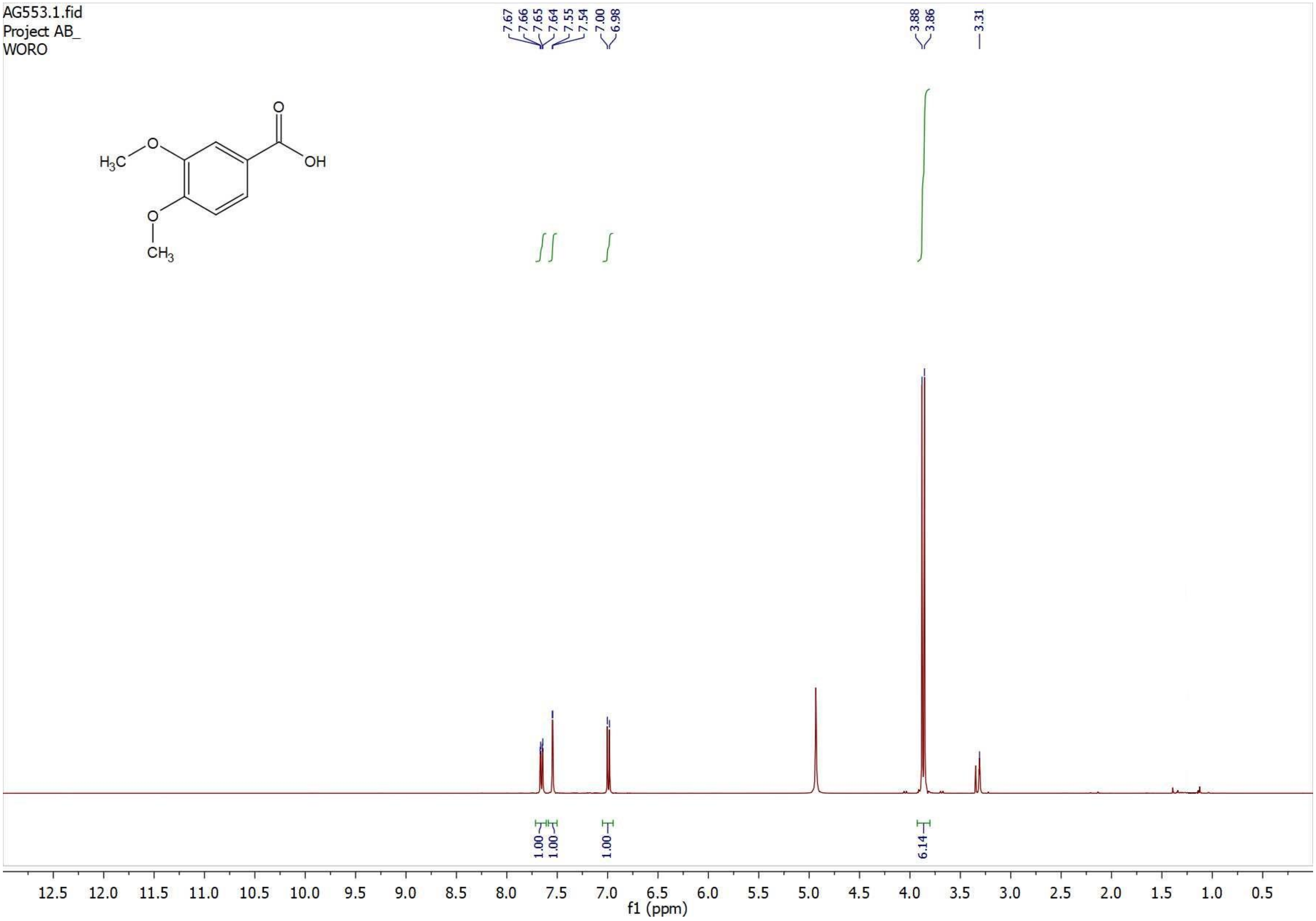

Compound p16

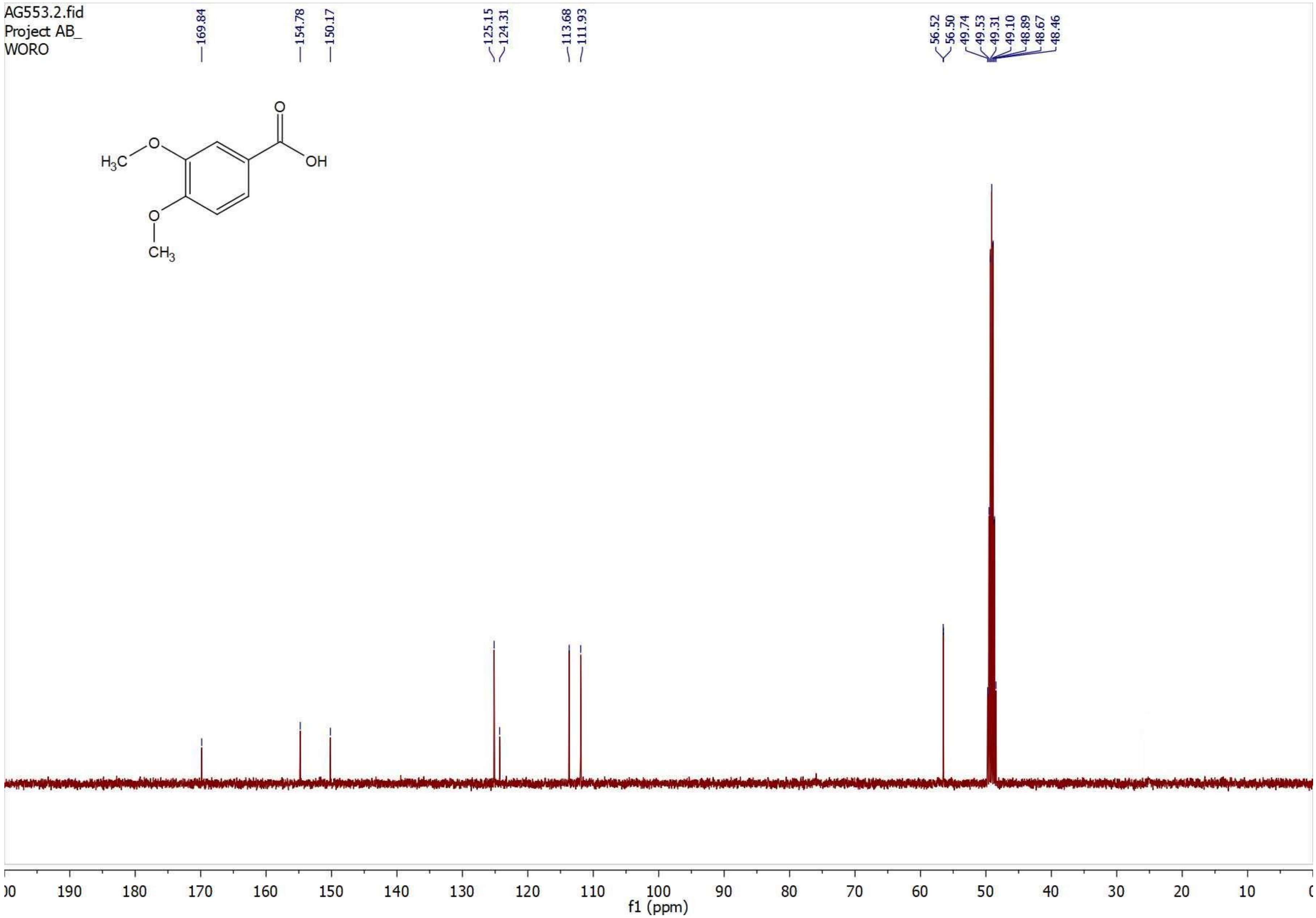

Compound p17

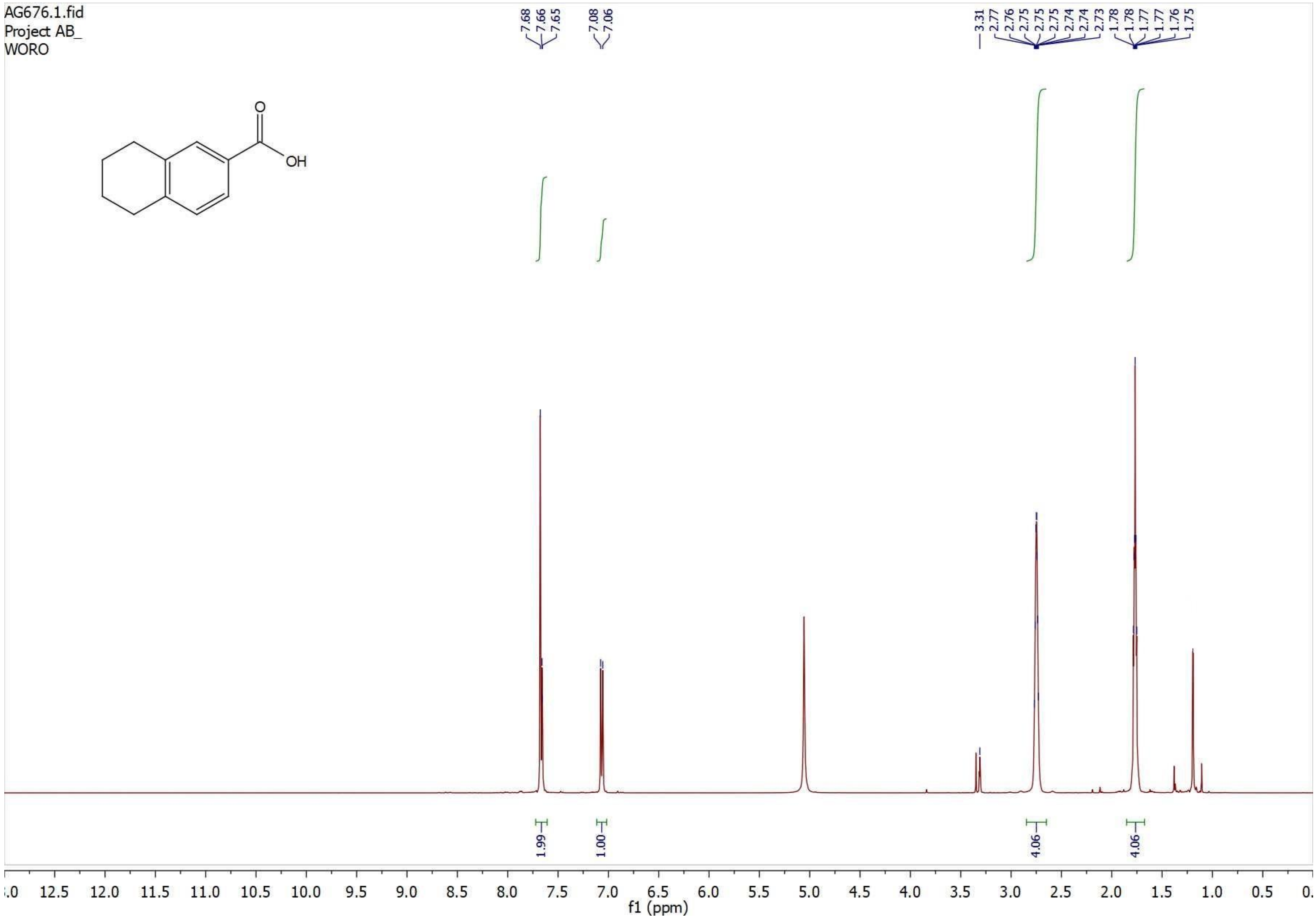

Compound p17

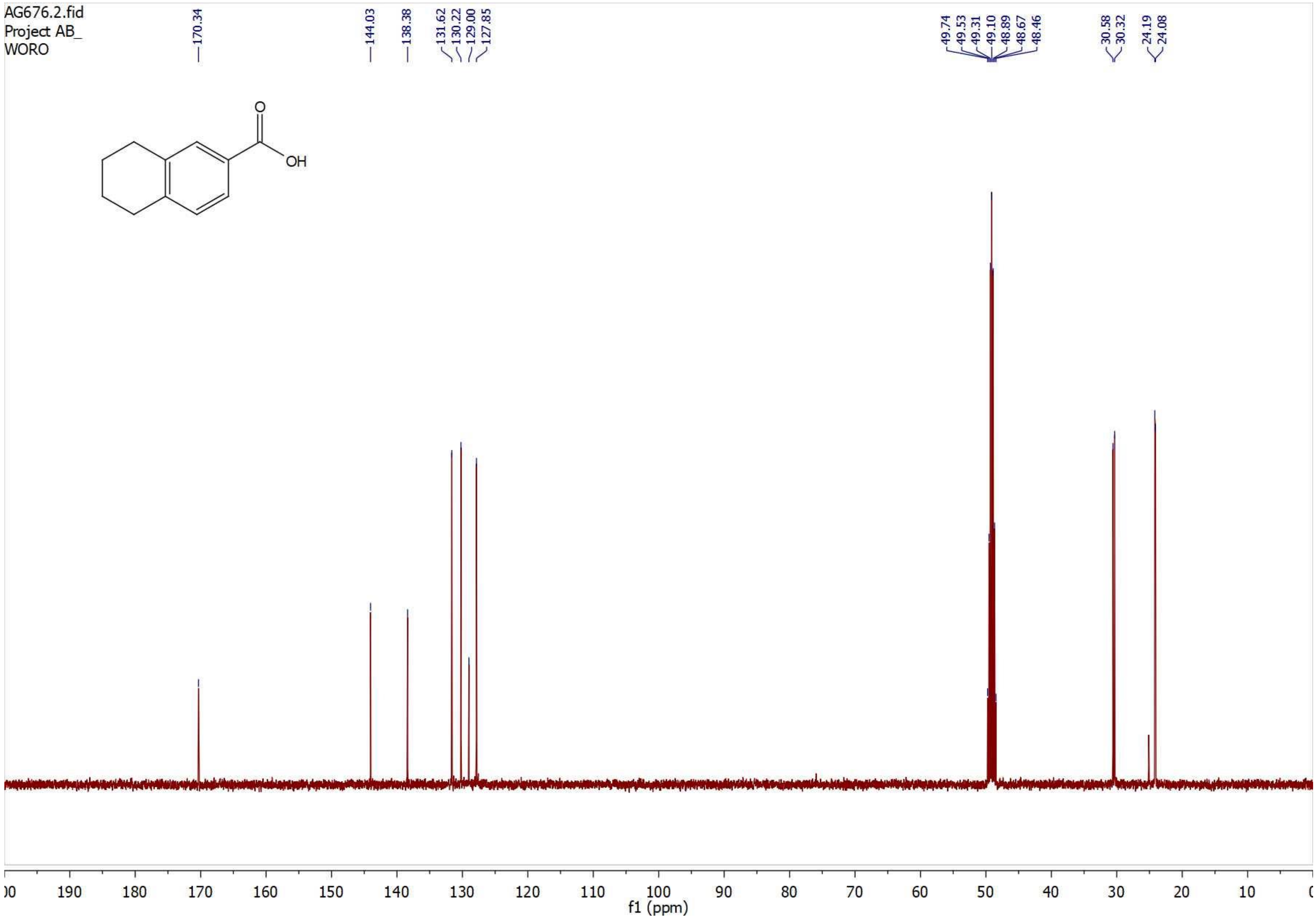

Compound p18

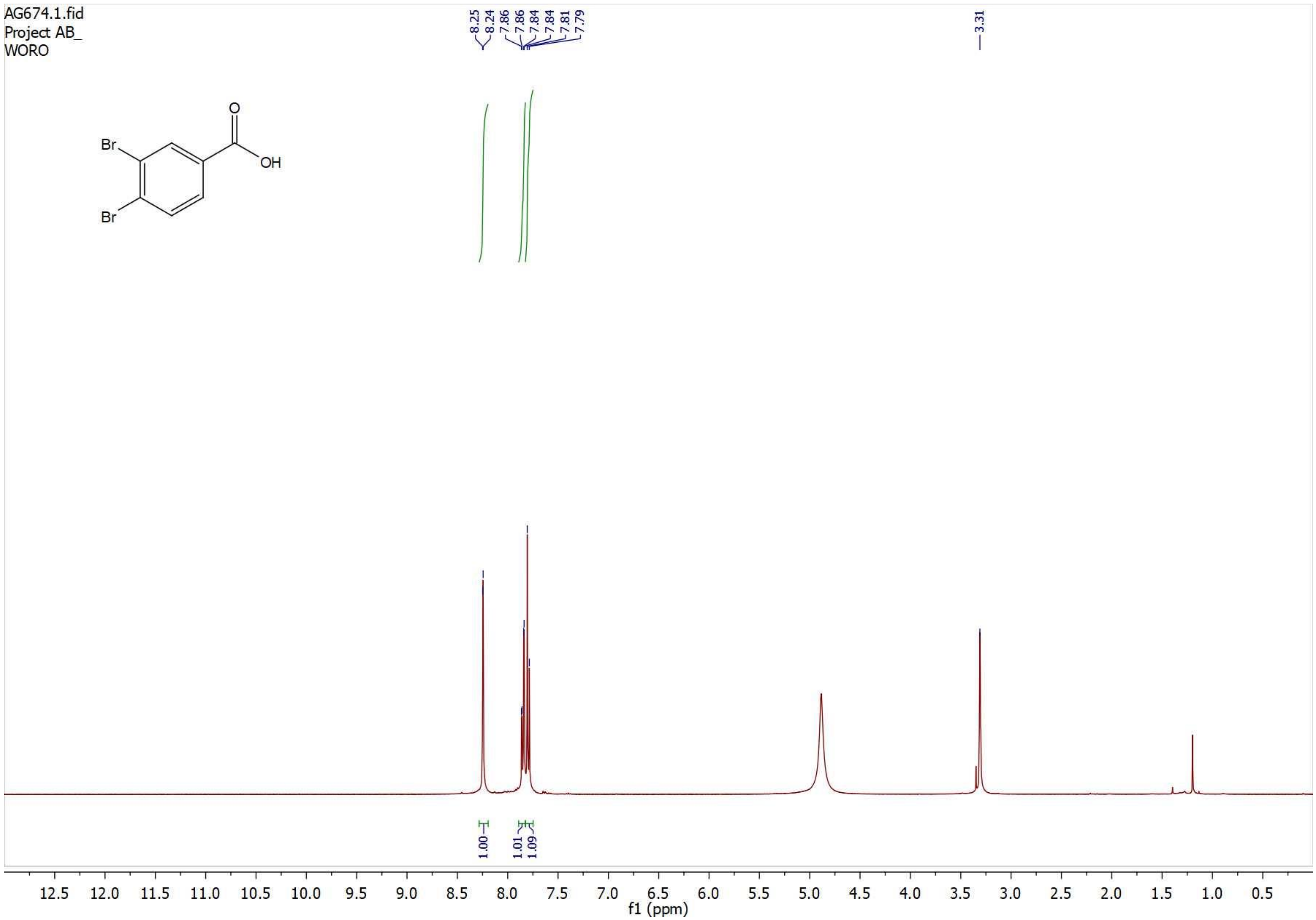

Compound p18

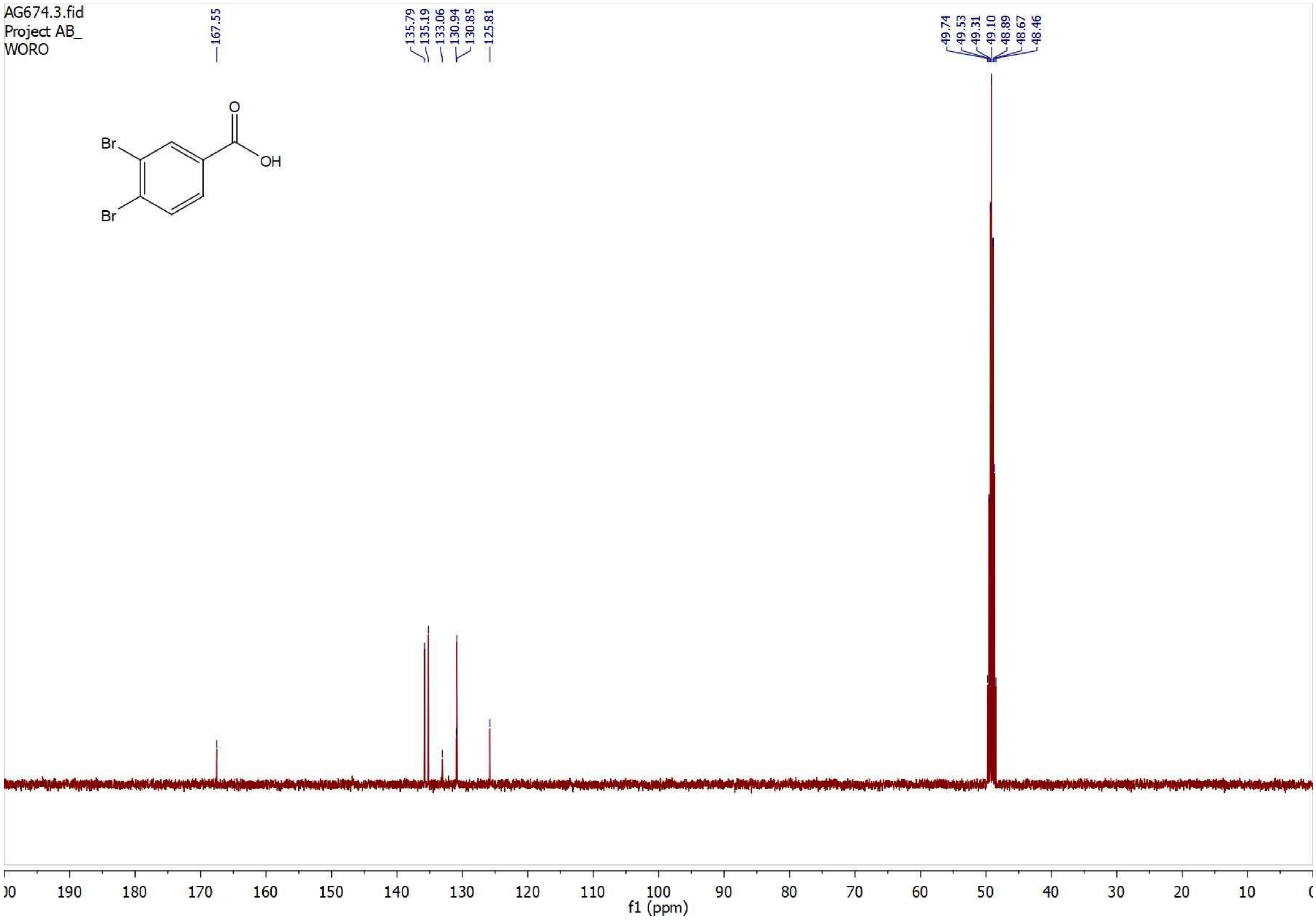

Compound p19

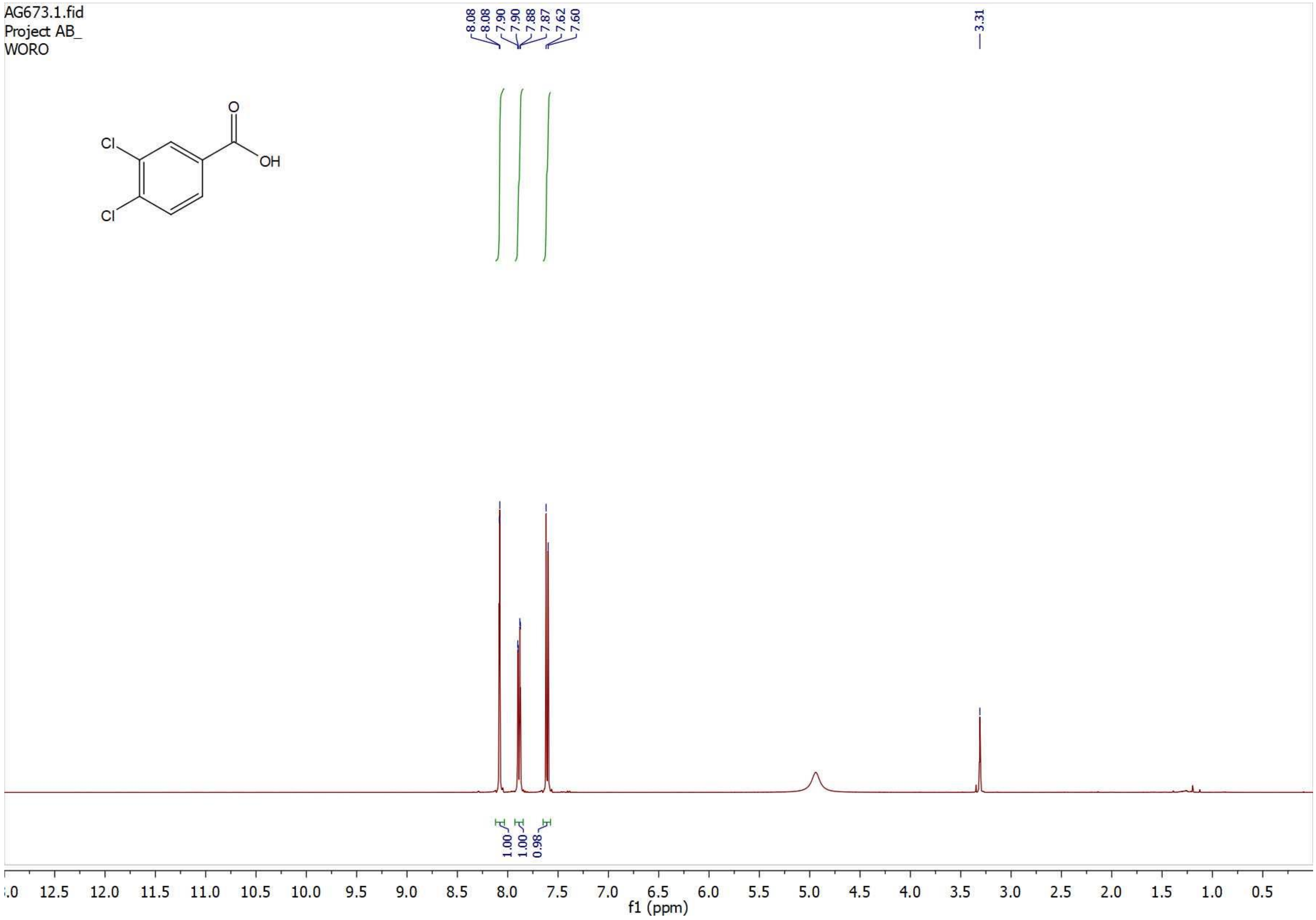

Compound p19

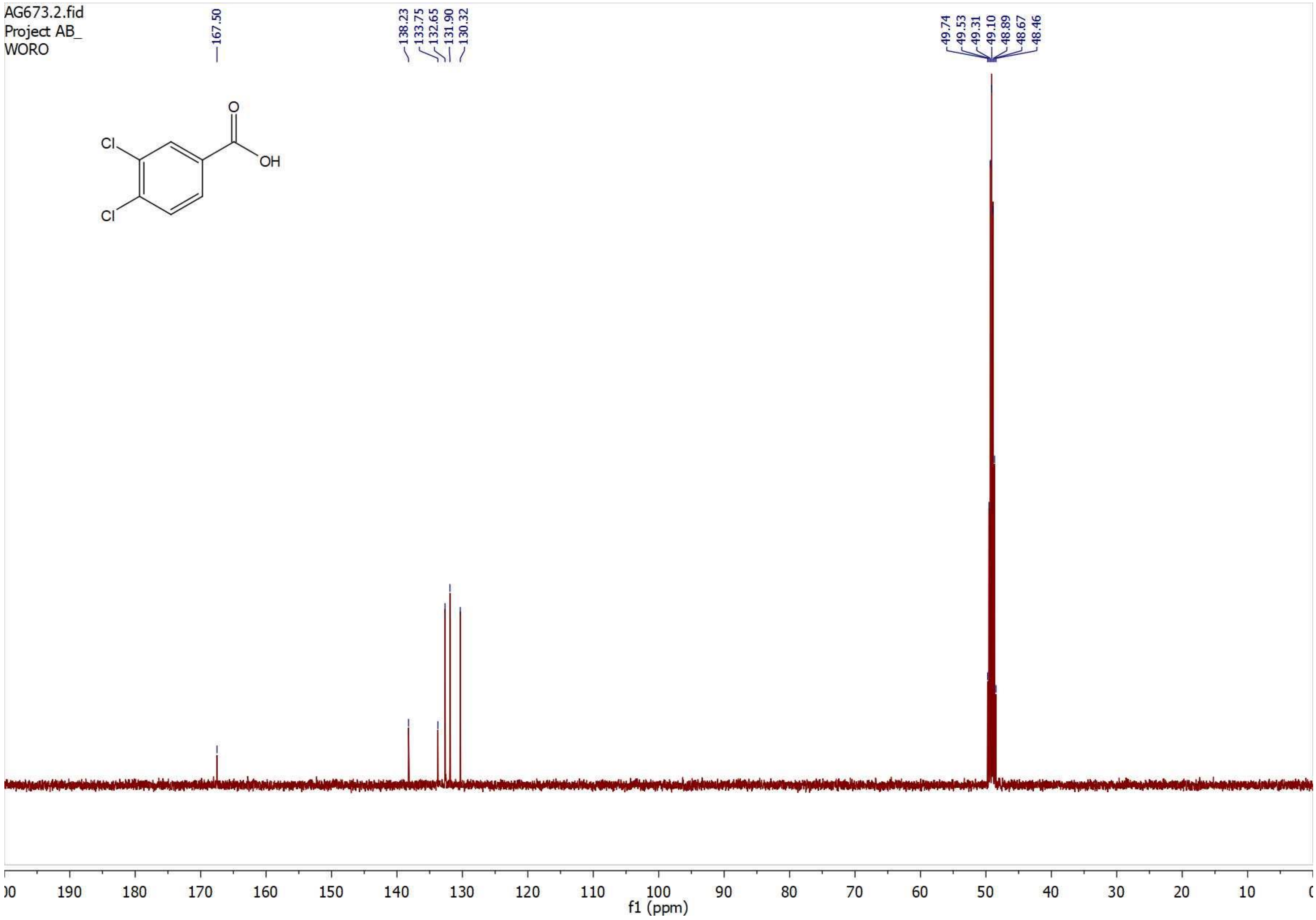

Compound p20

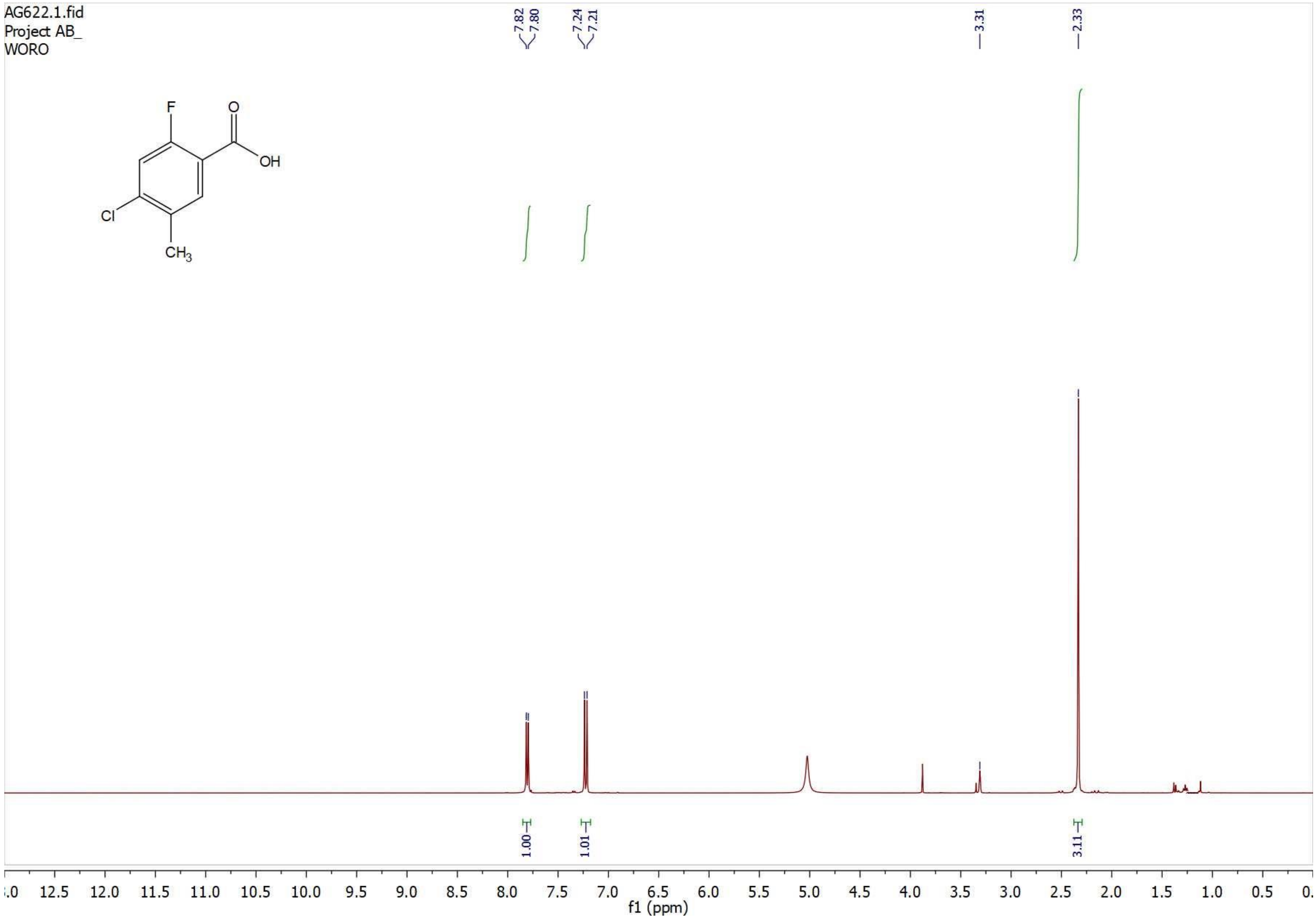

Compound p20

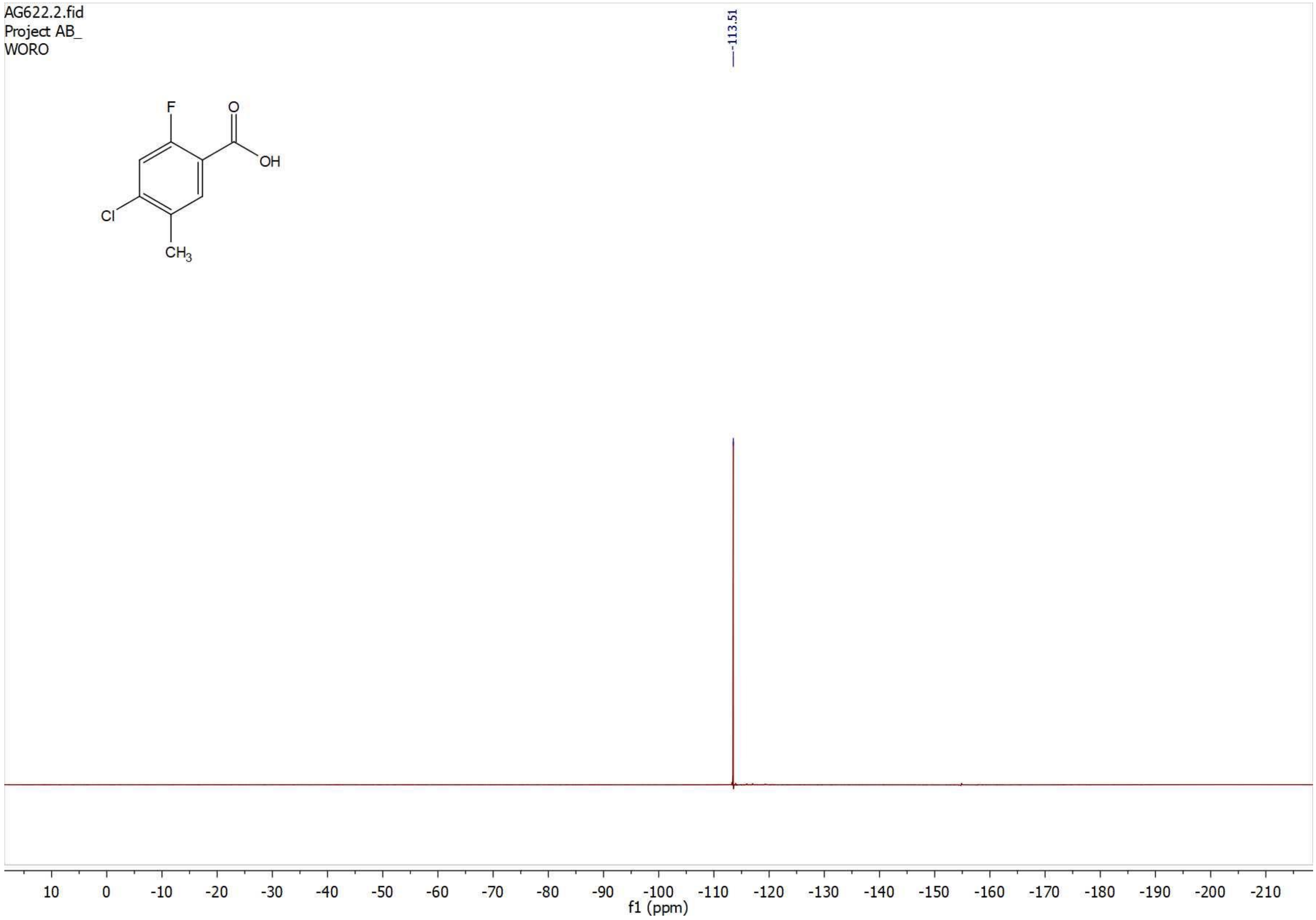

Compound p20

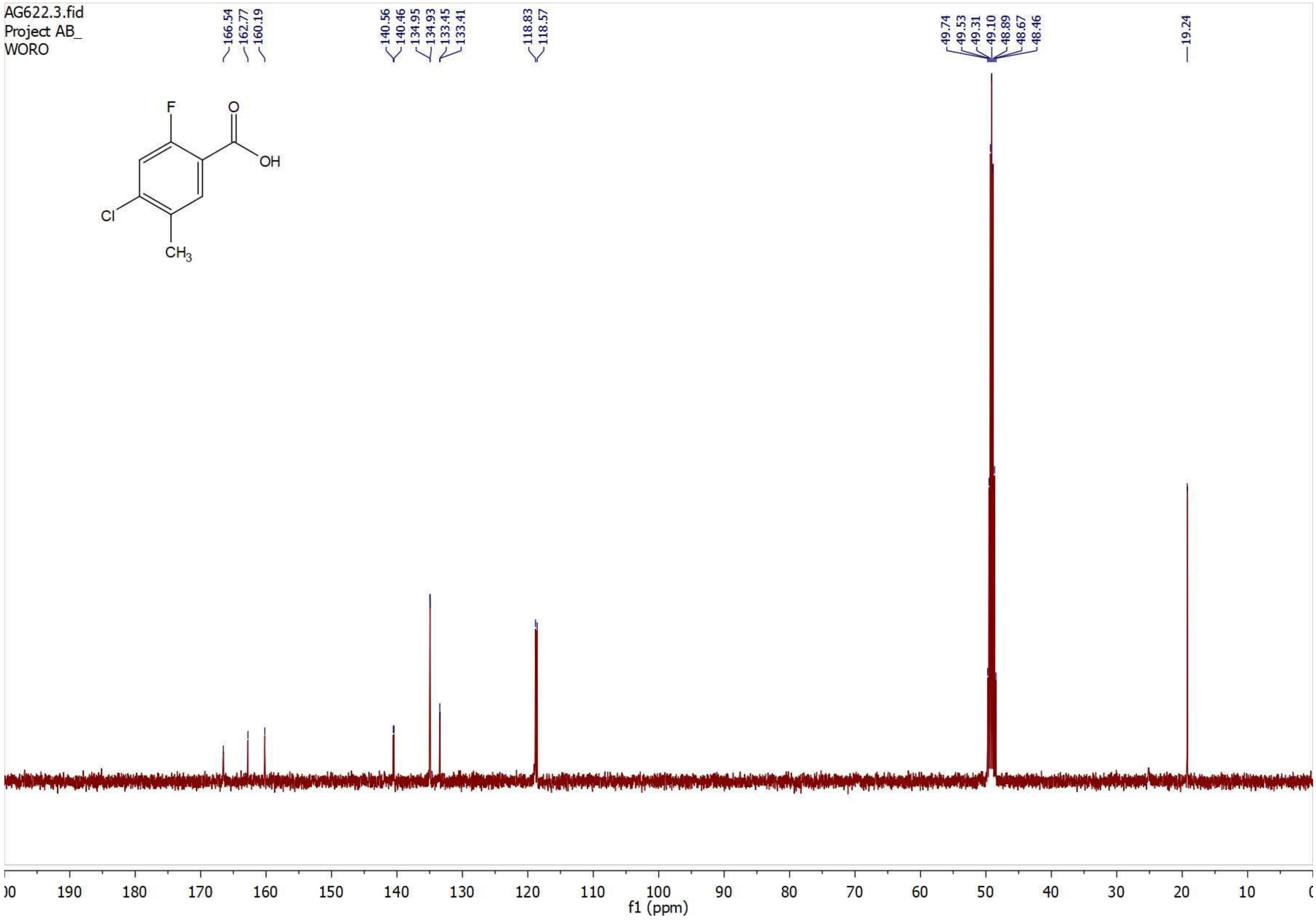

Compound p21

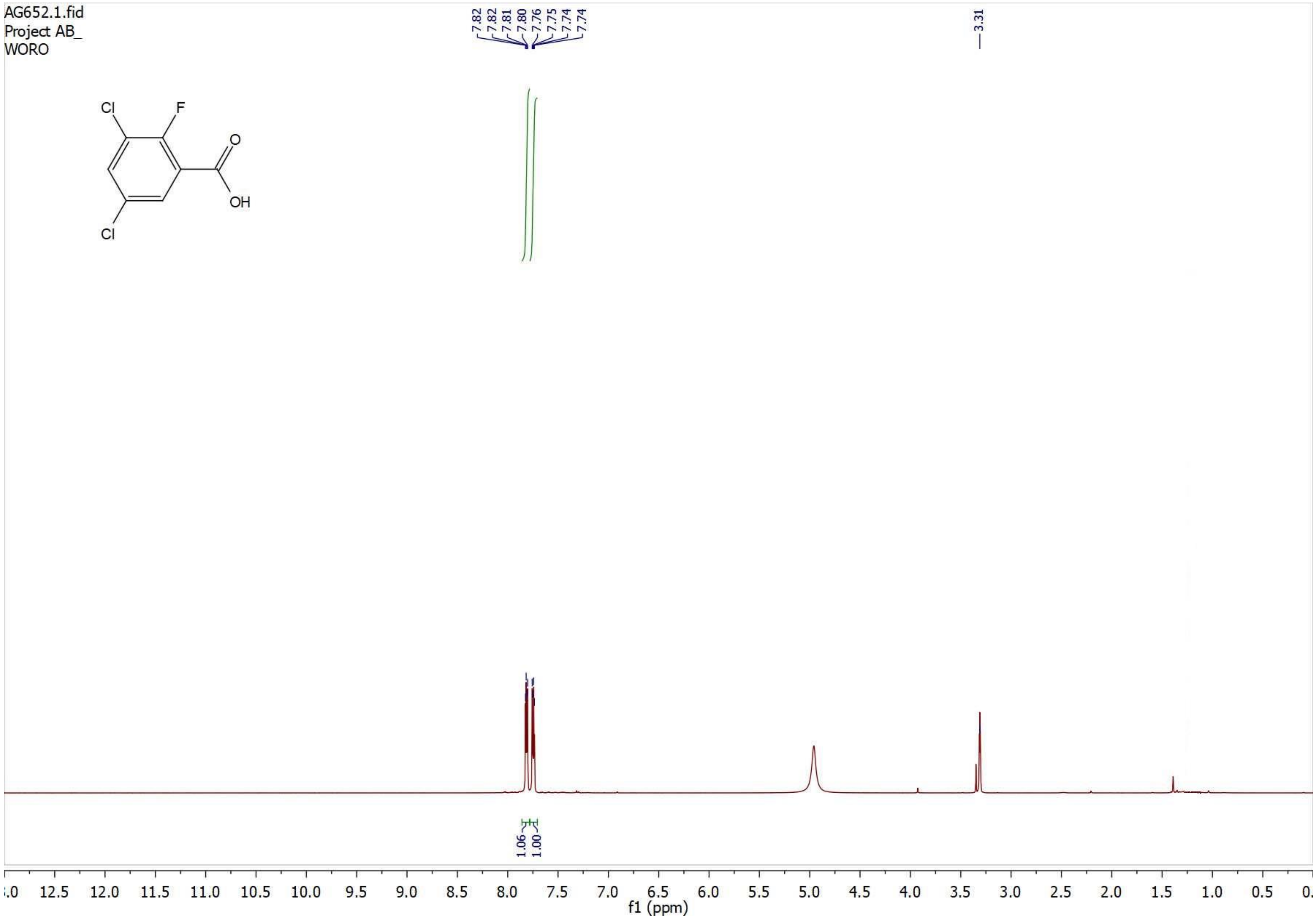

Compound p21

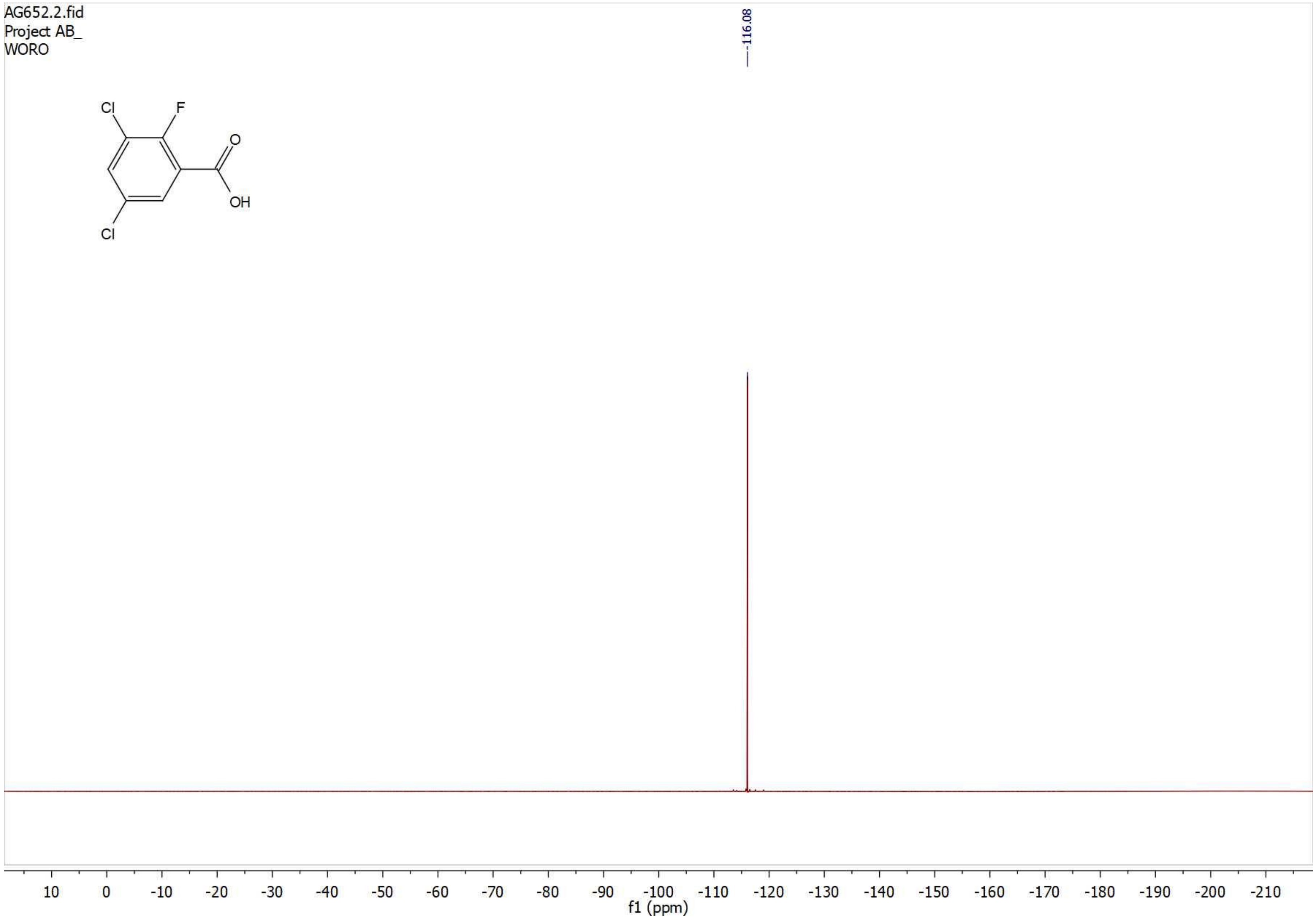

Compound p21

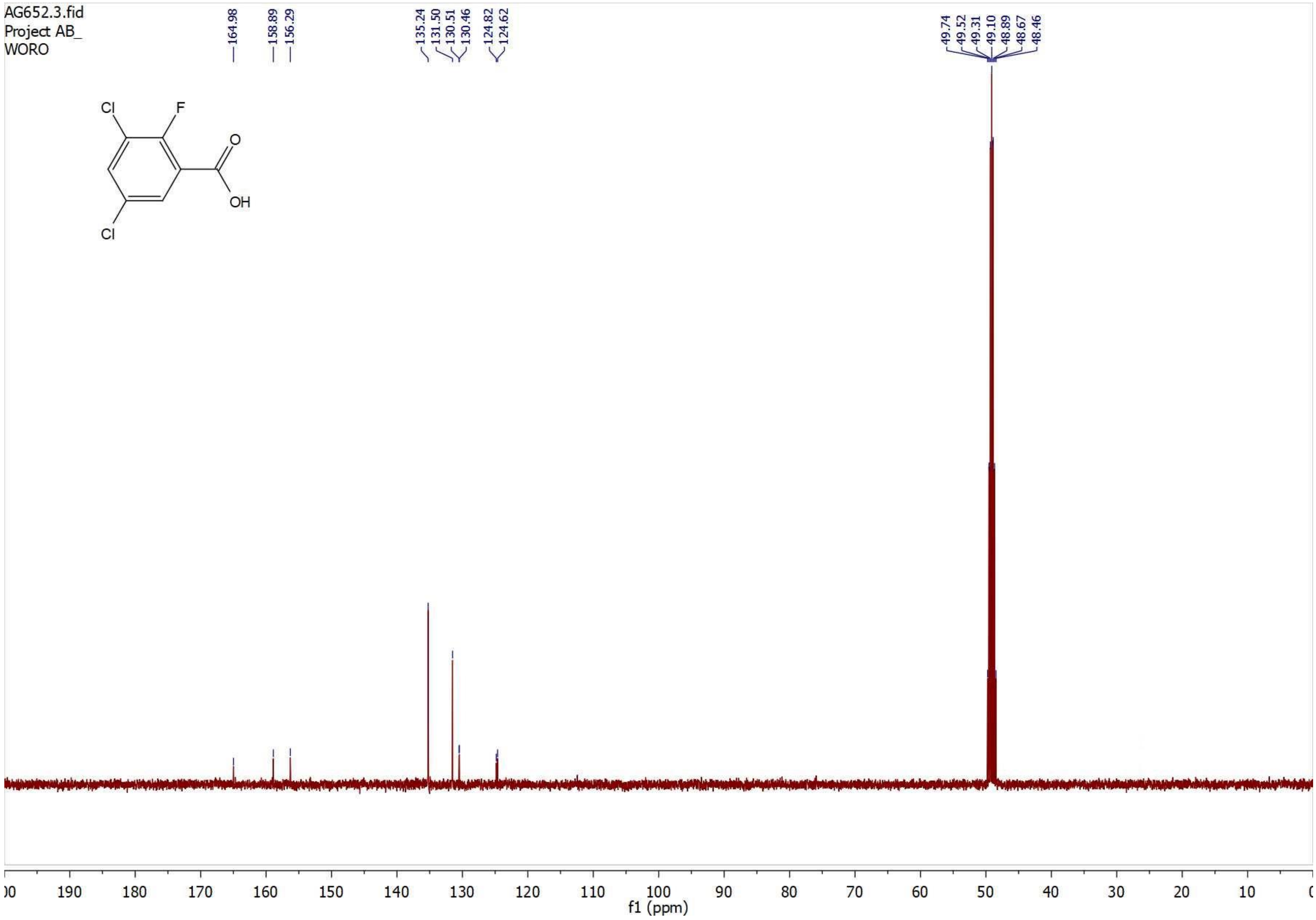

Compound p22

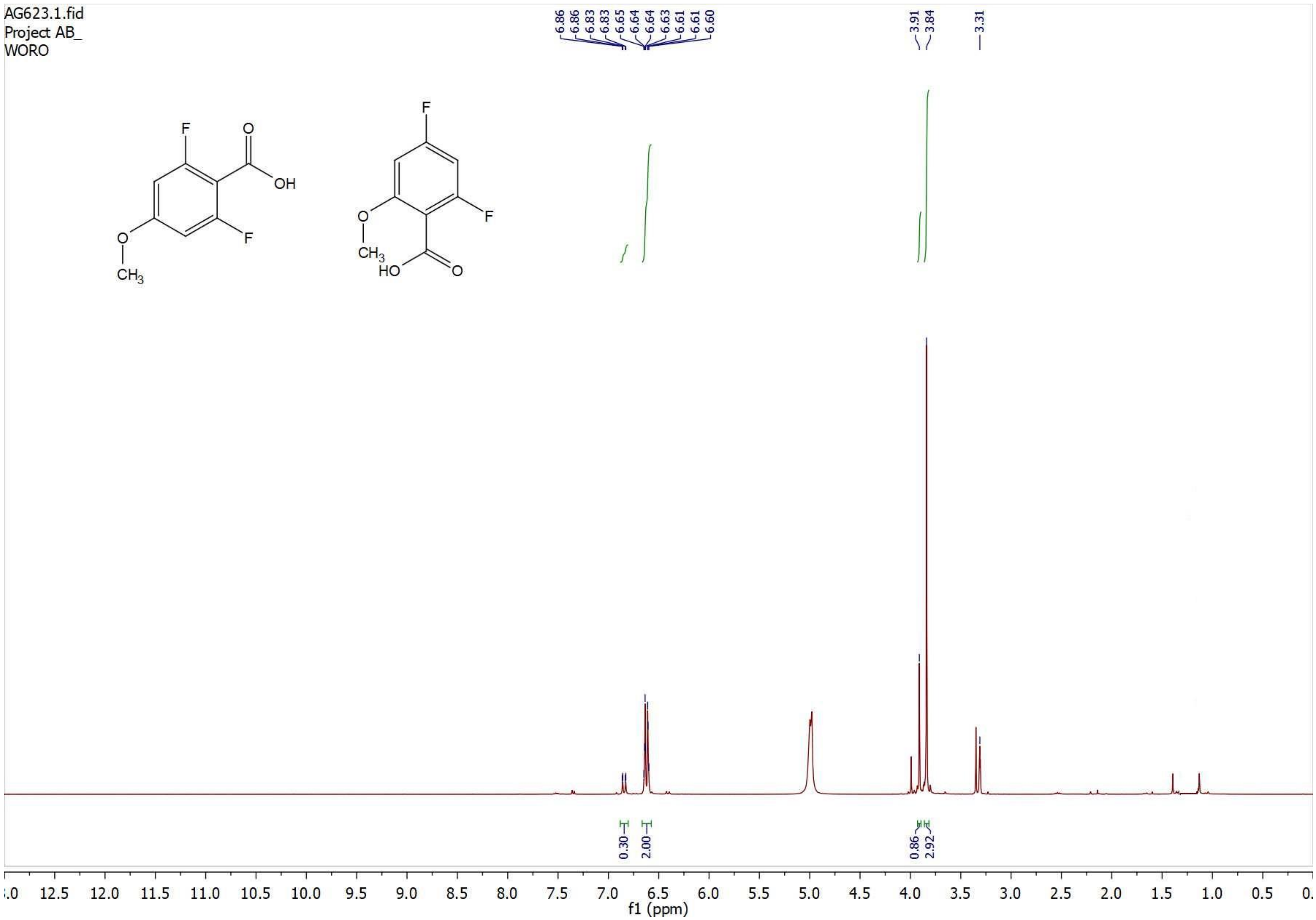

Compound p22

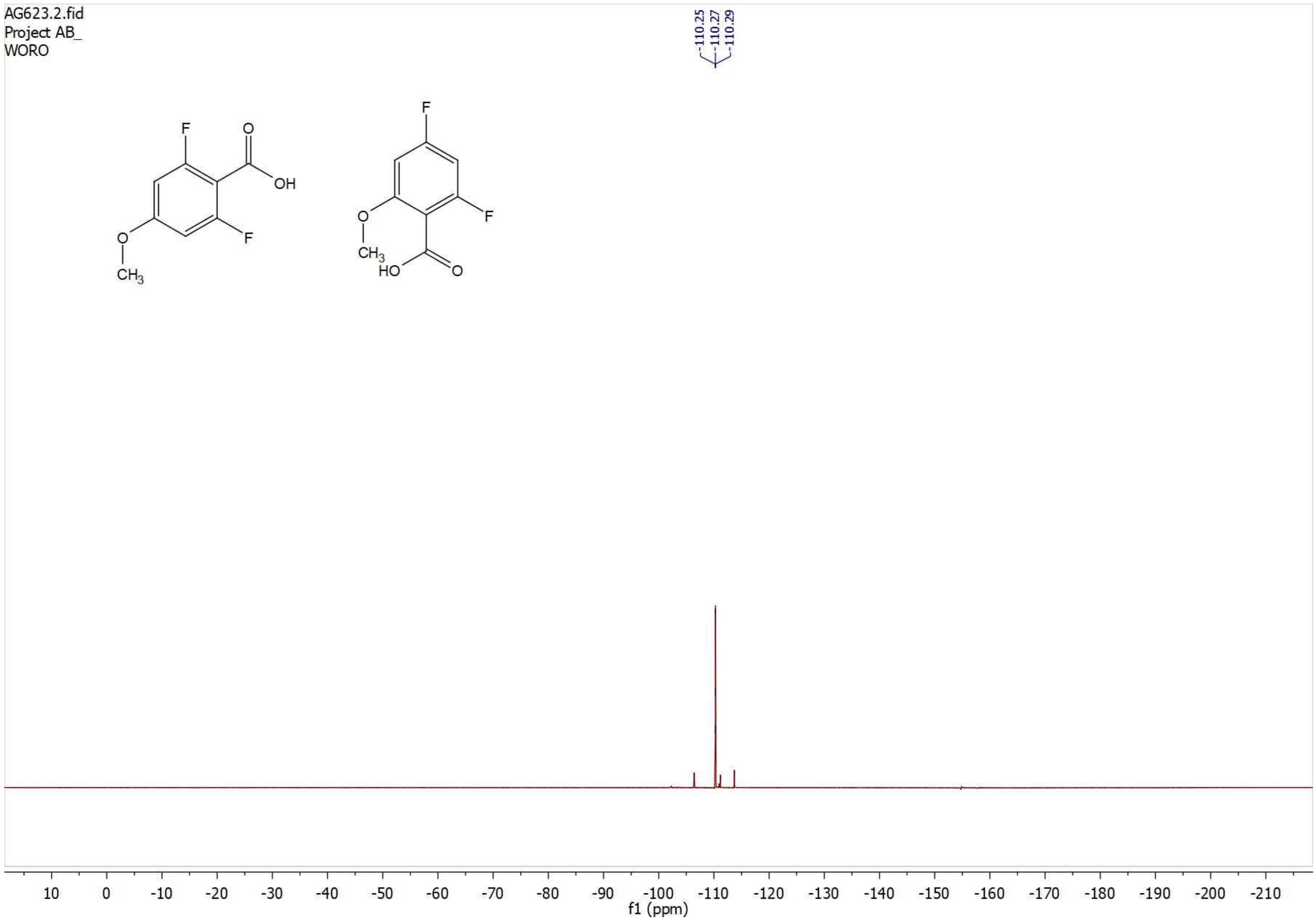

Compound p22

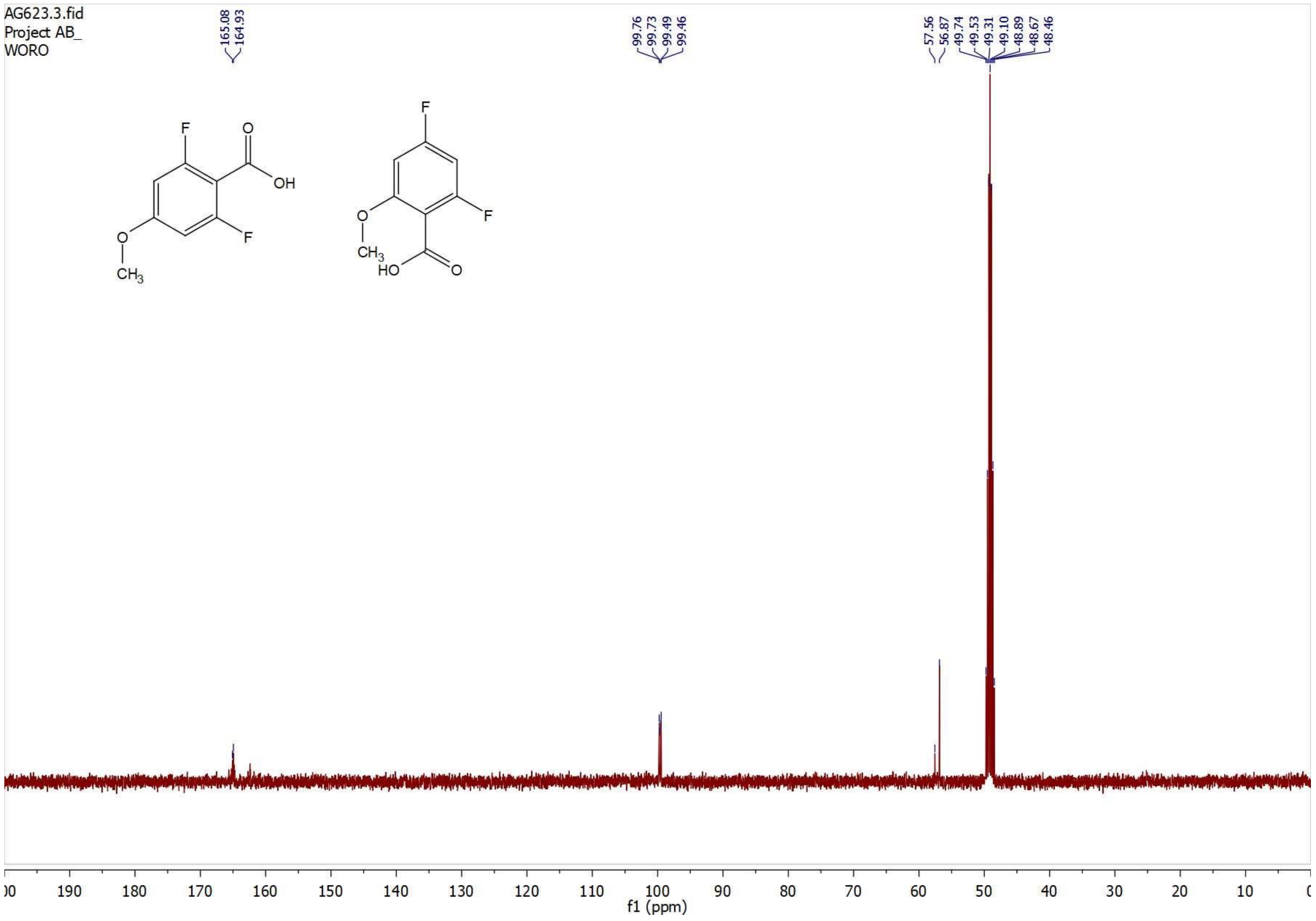

Compound p23

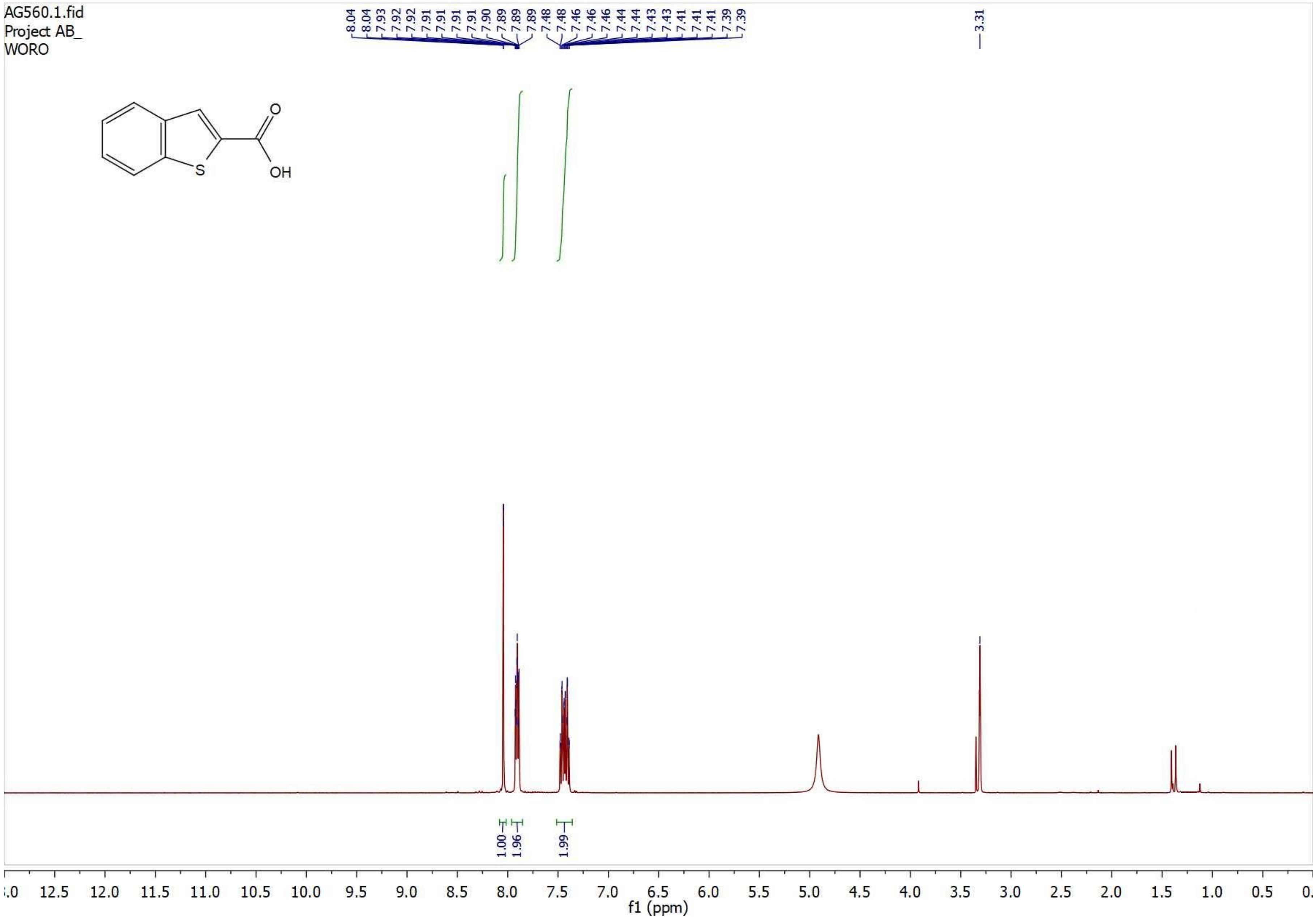

Compound p23

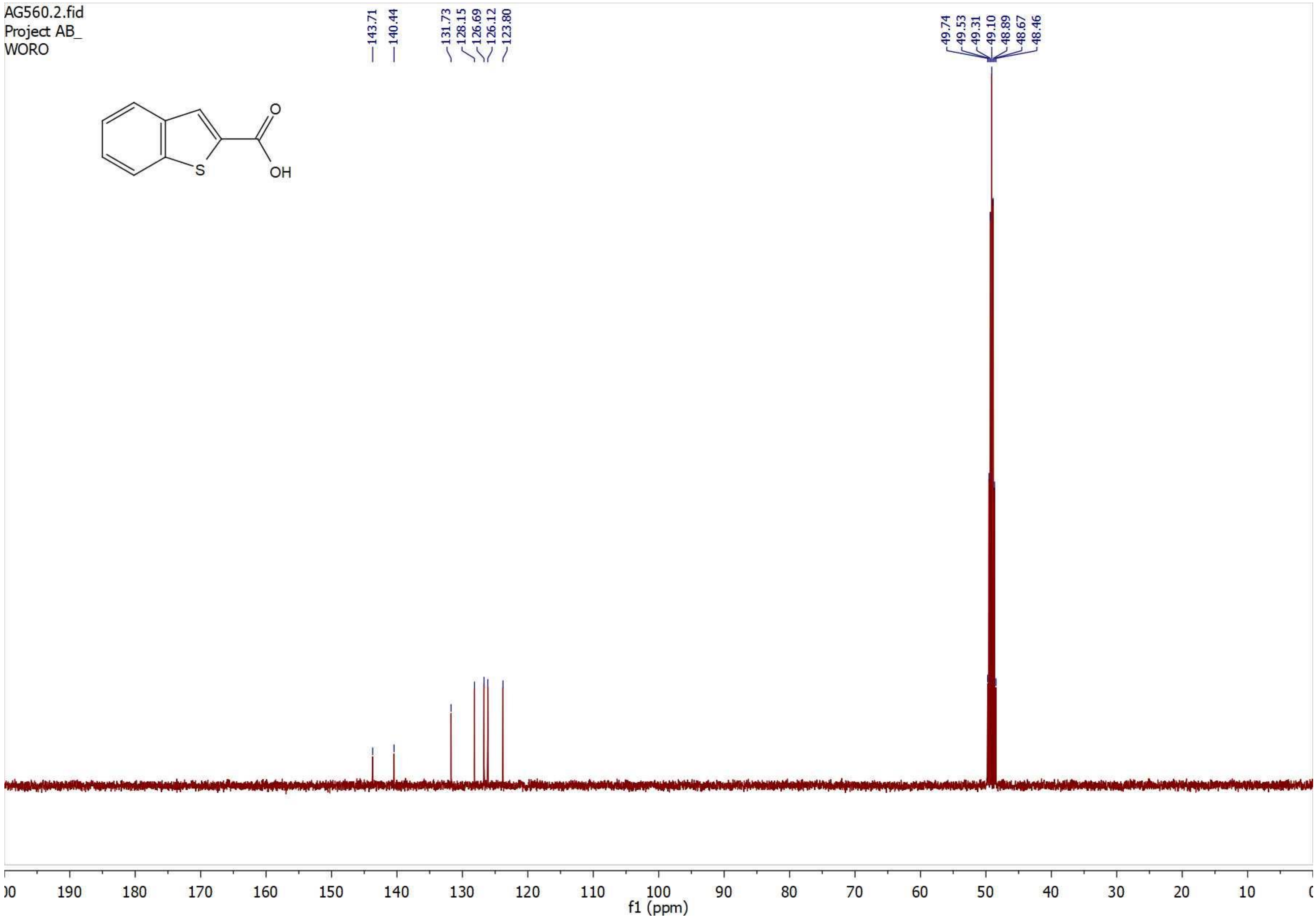

Compound p24

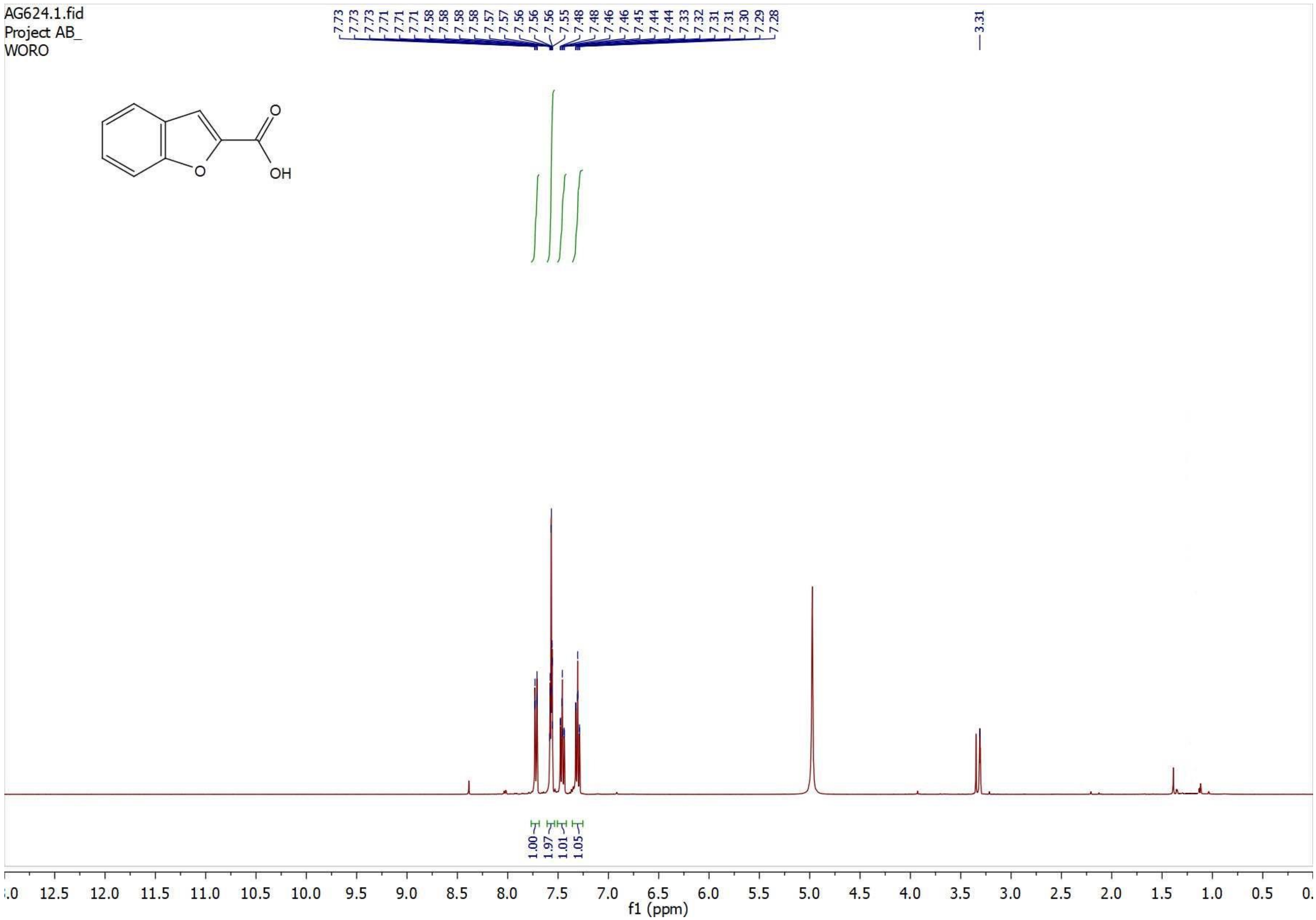

Compound p24

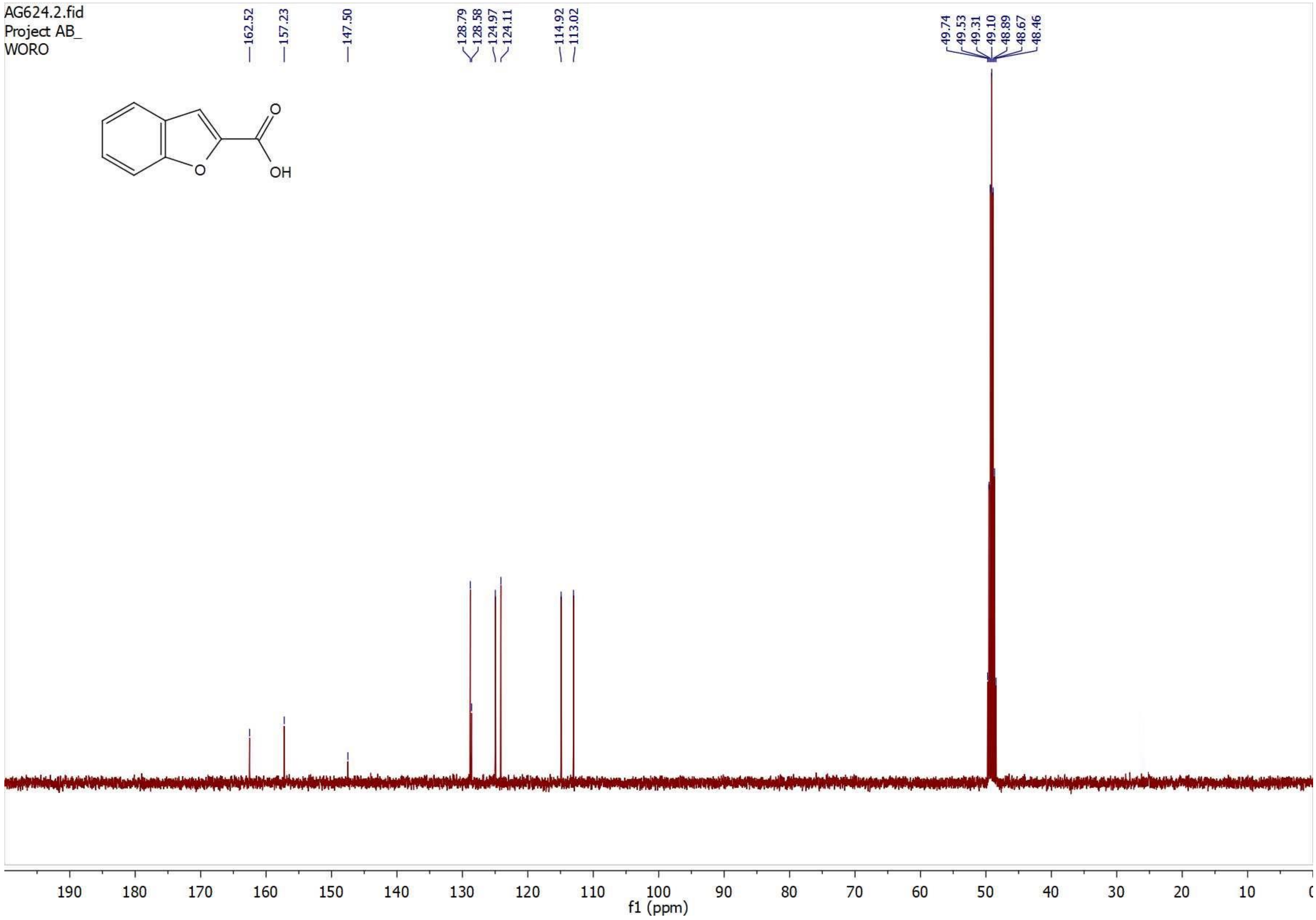

Compound p25

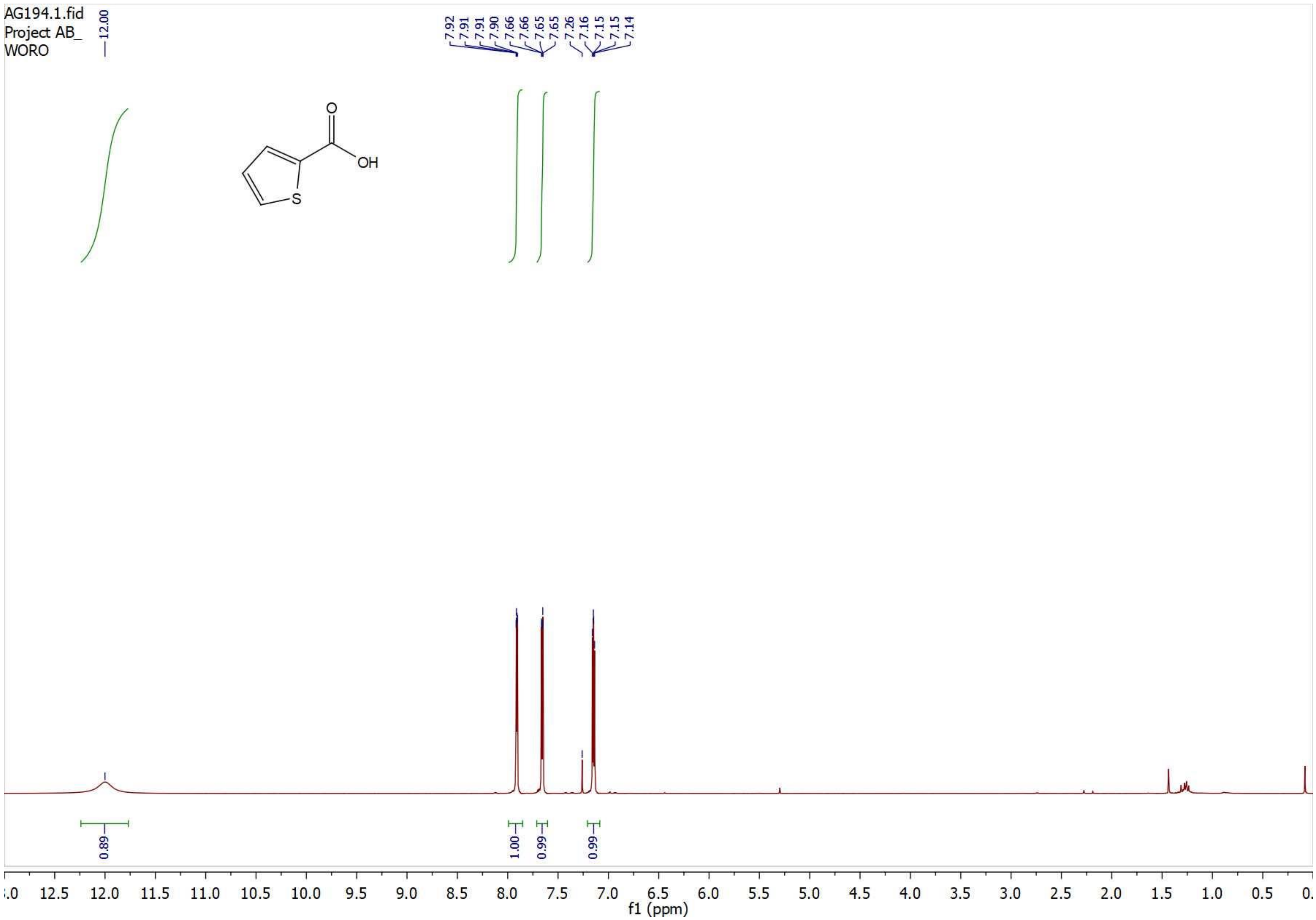

Compound p25

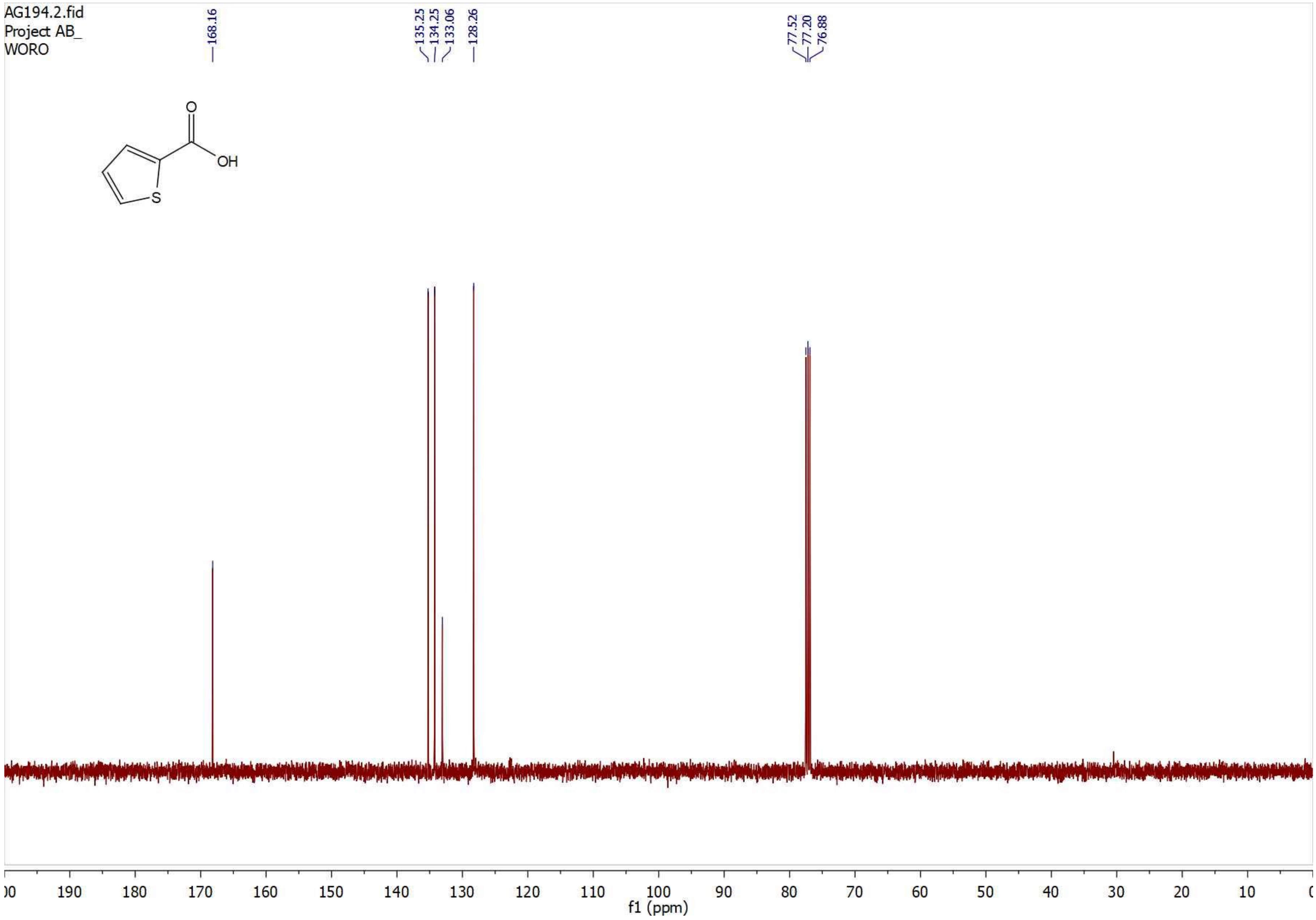

Compound p26

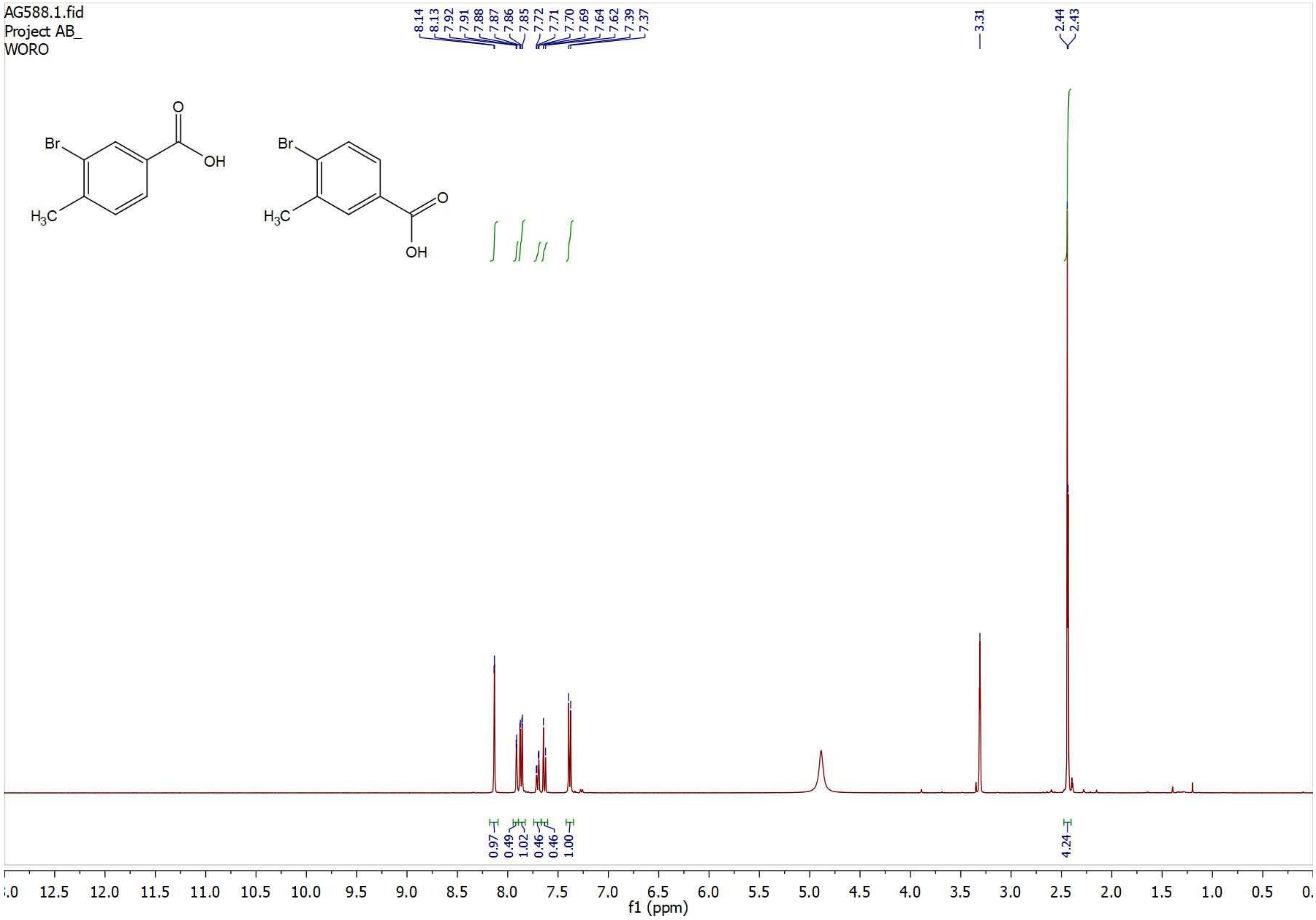

Compound p26

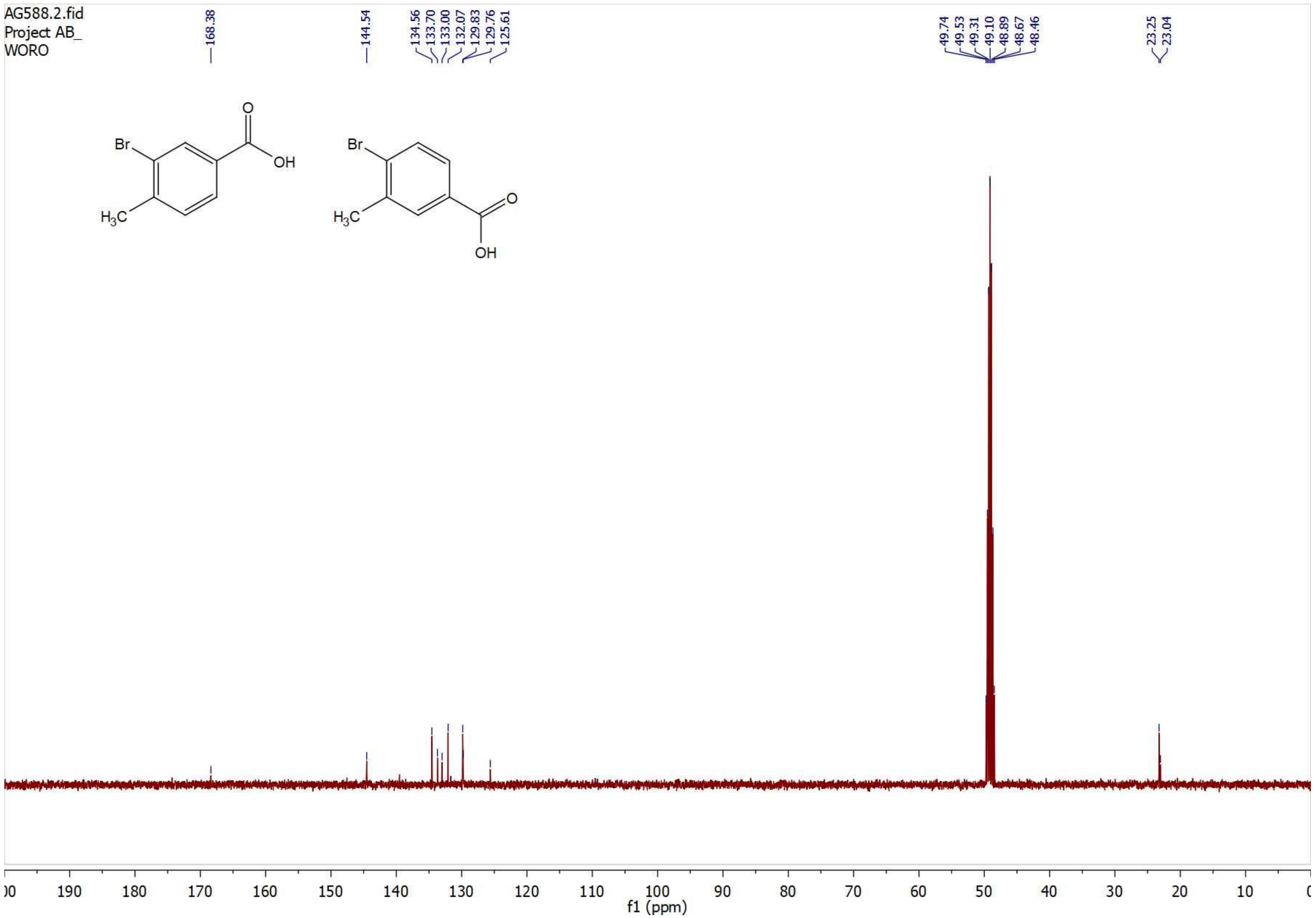

Compound p27

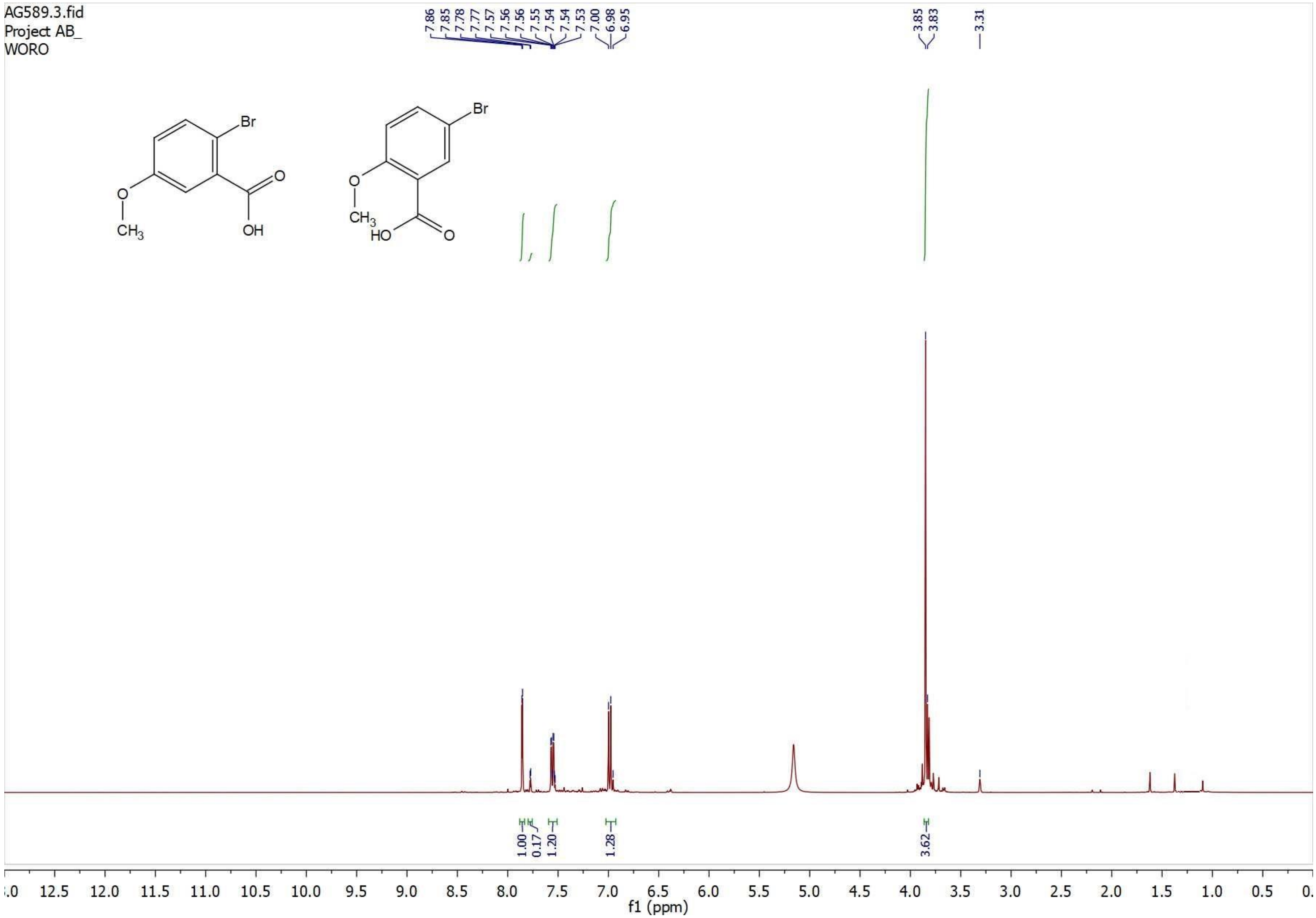

Compound p27

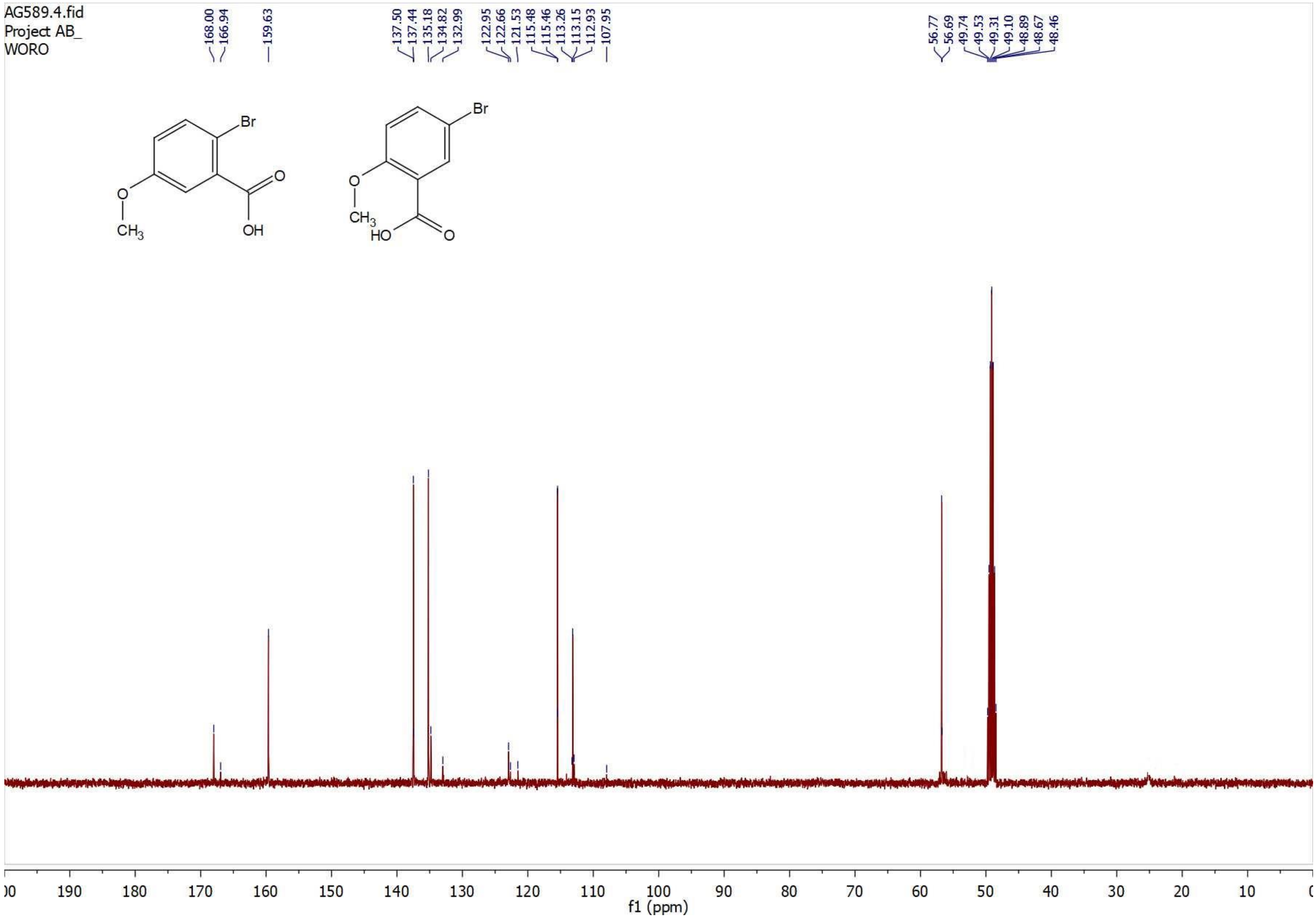

Compound p28

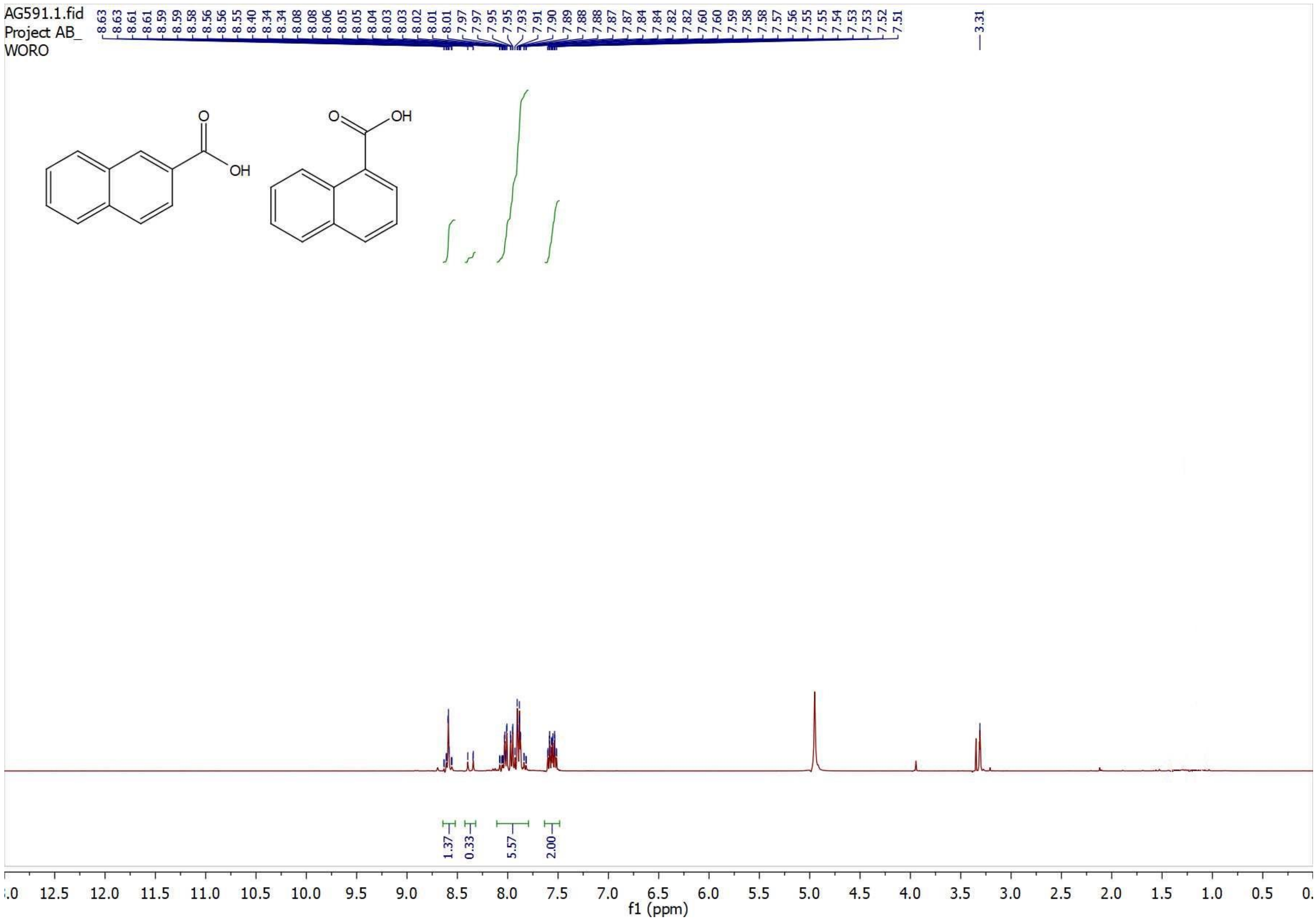

Compound p28

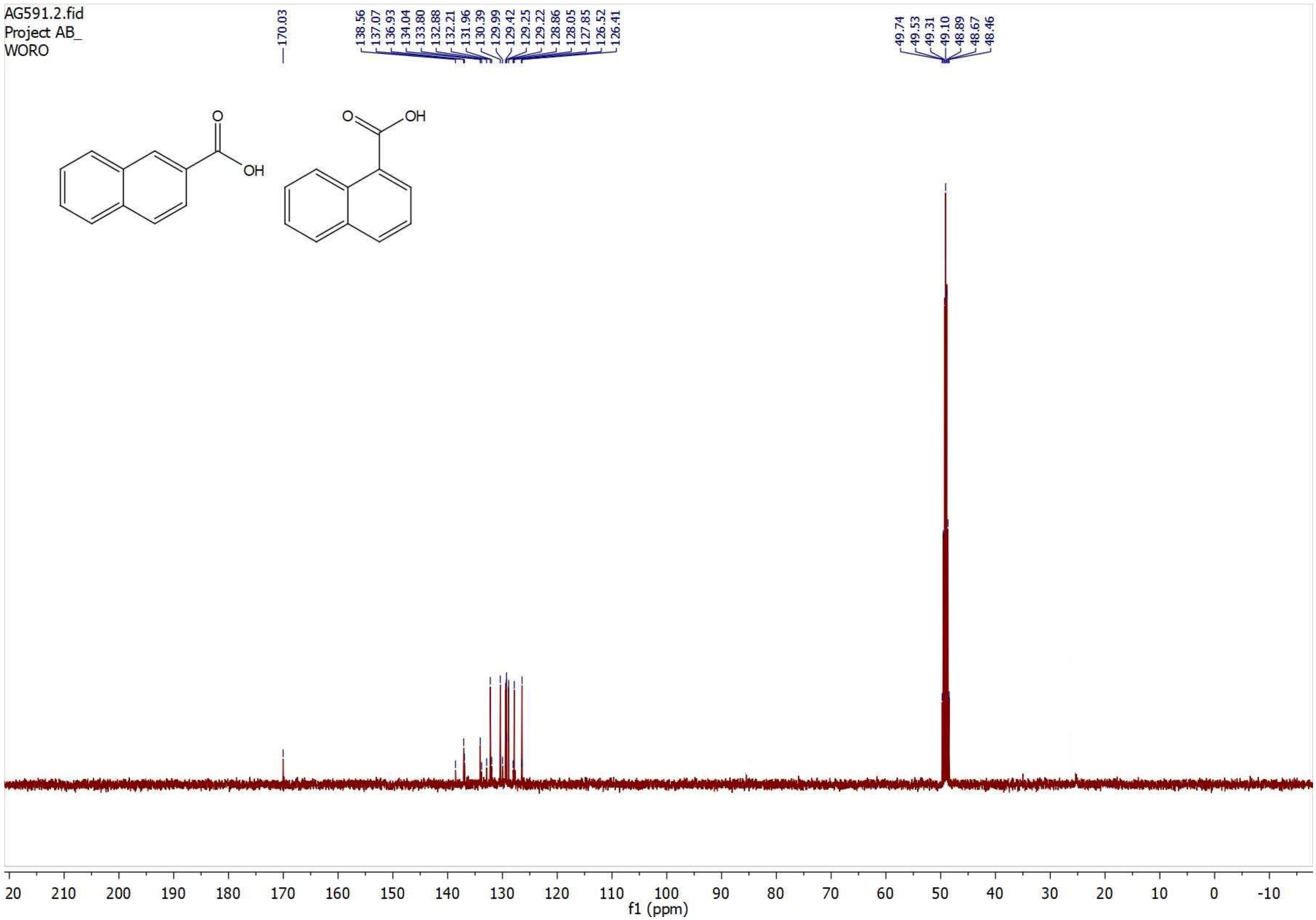

Compound p29

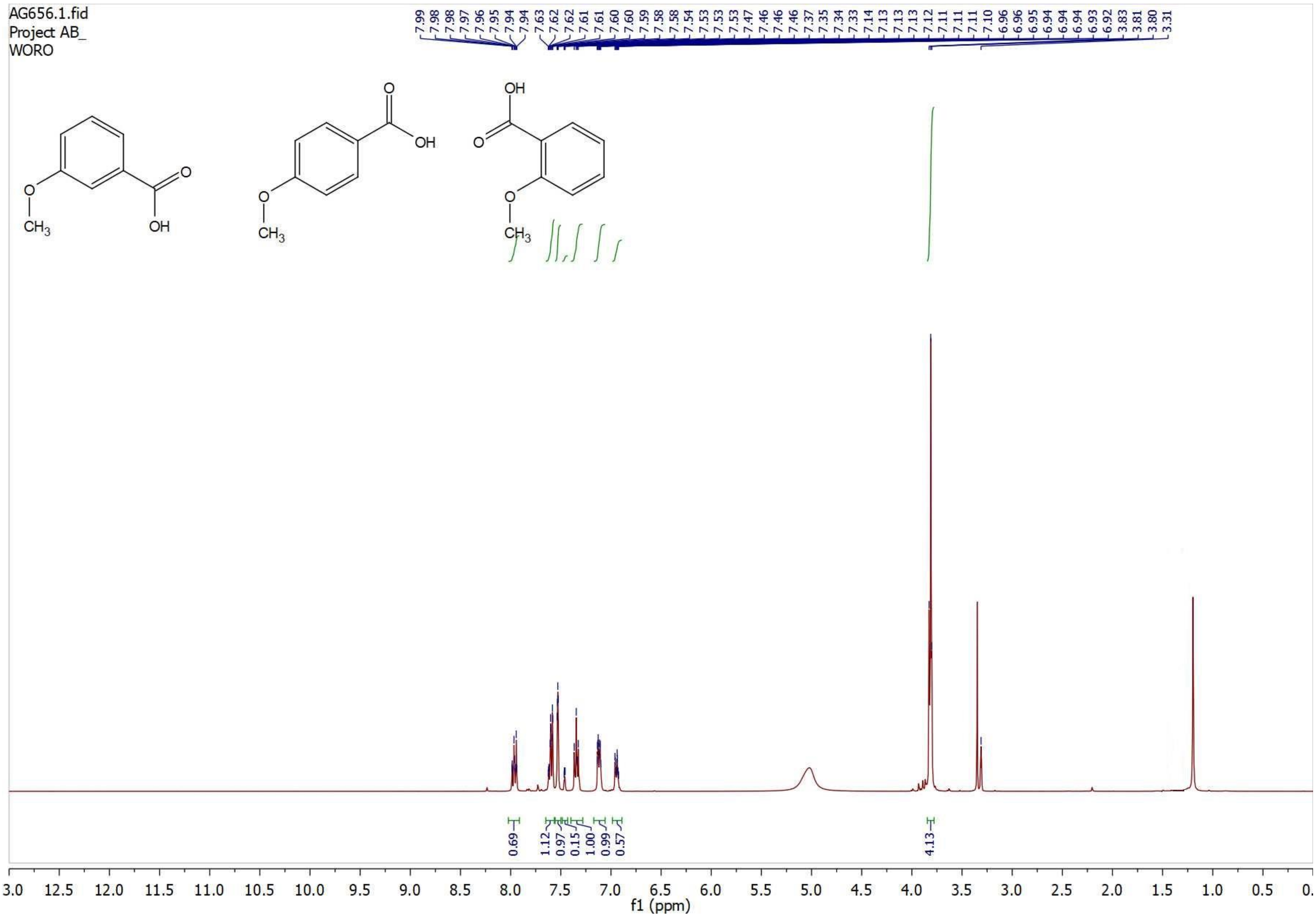

Compound p29

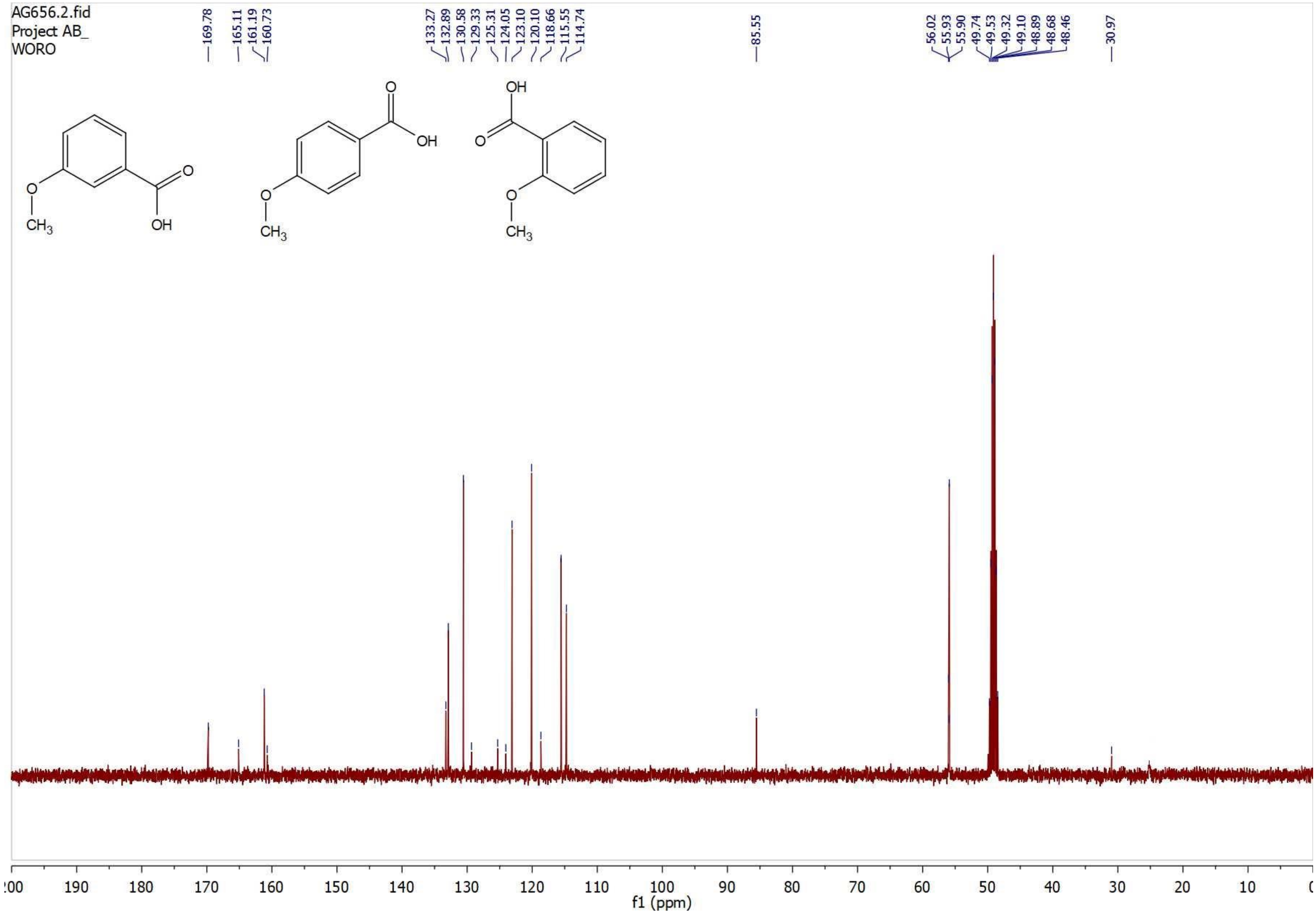

Compound p30

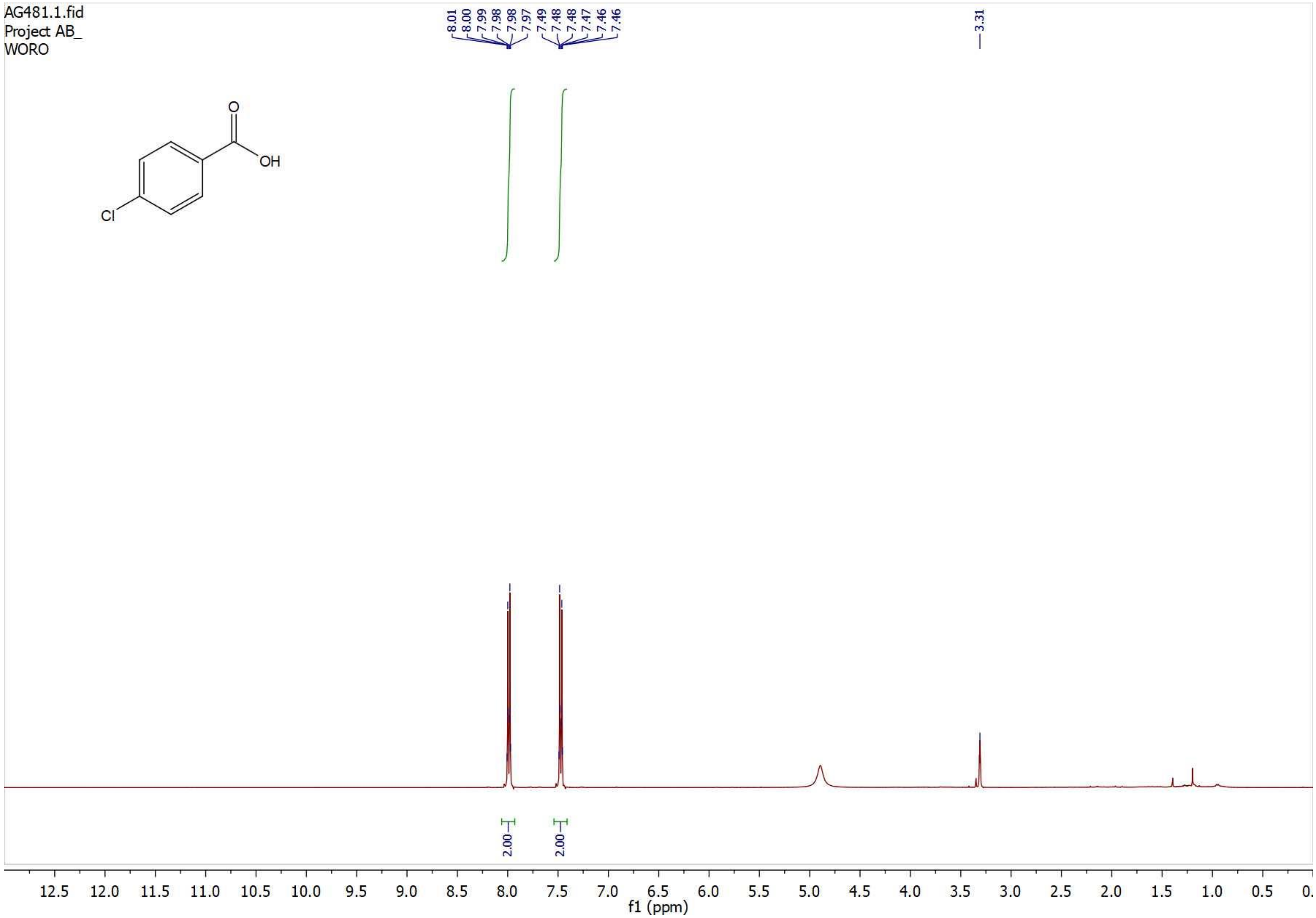

Compound p30

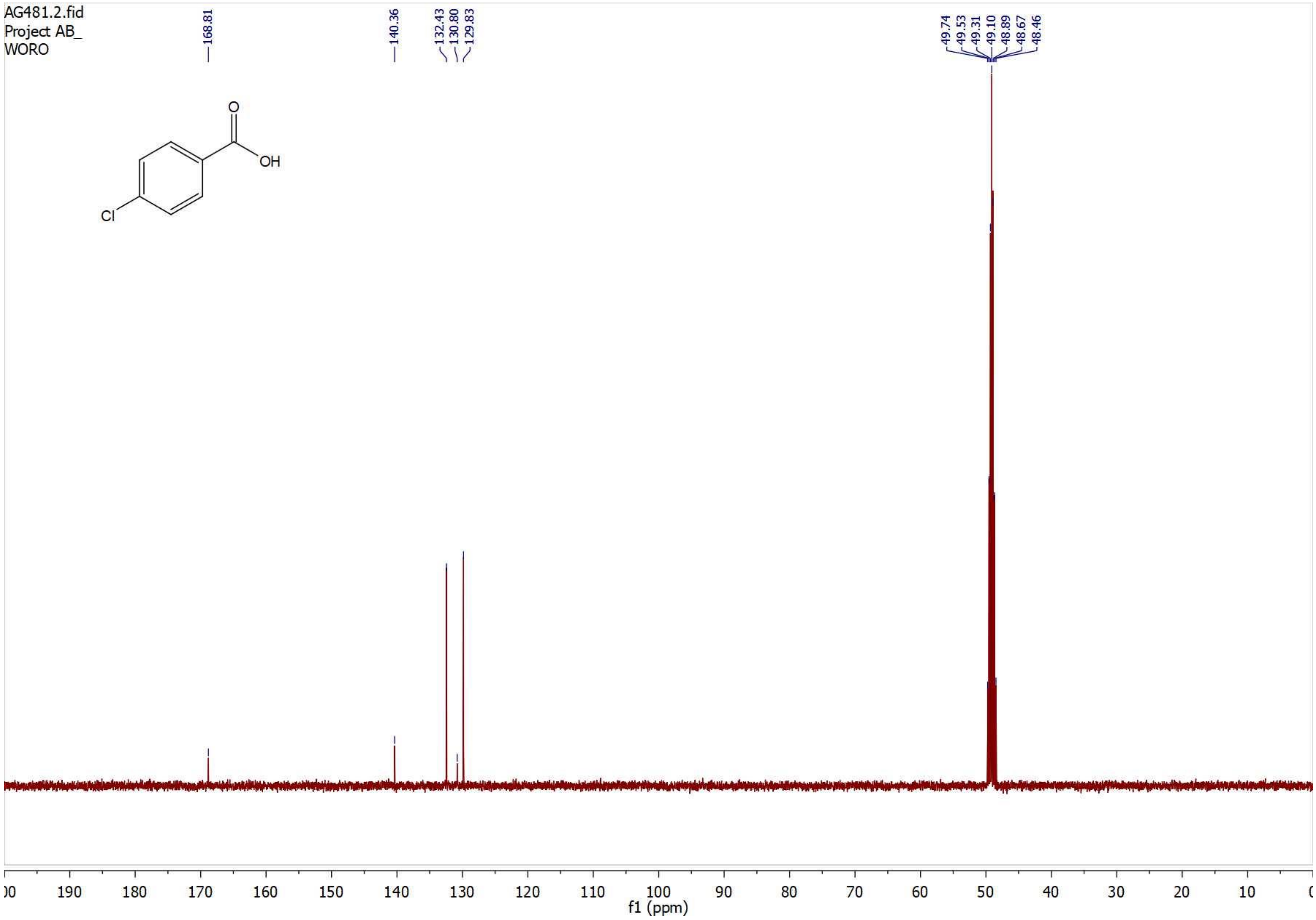

Compound p31

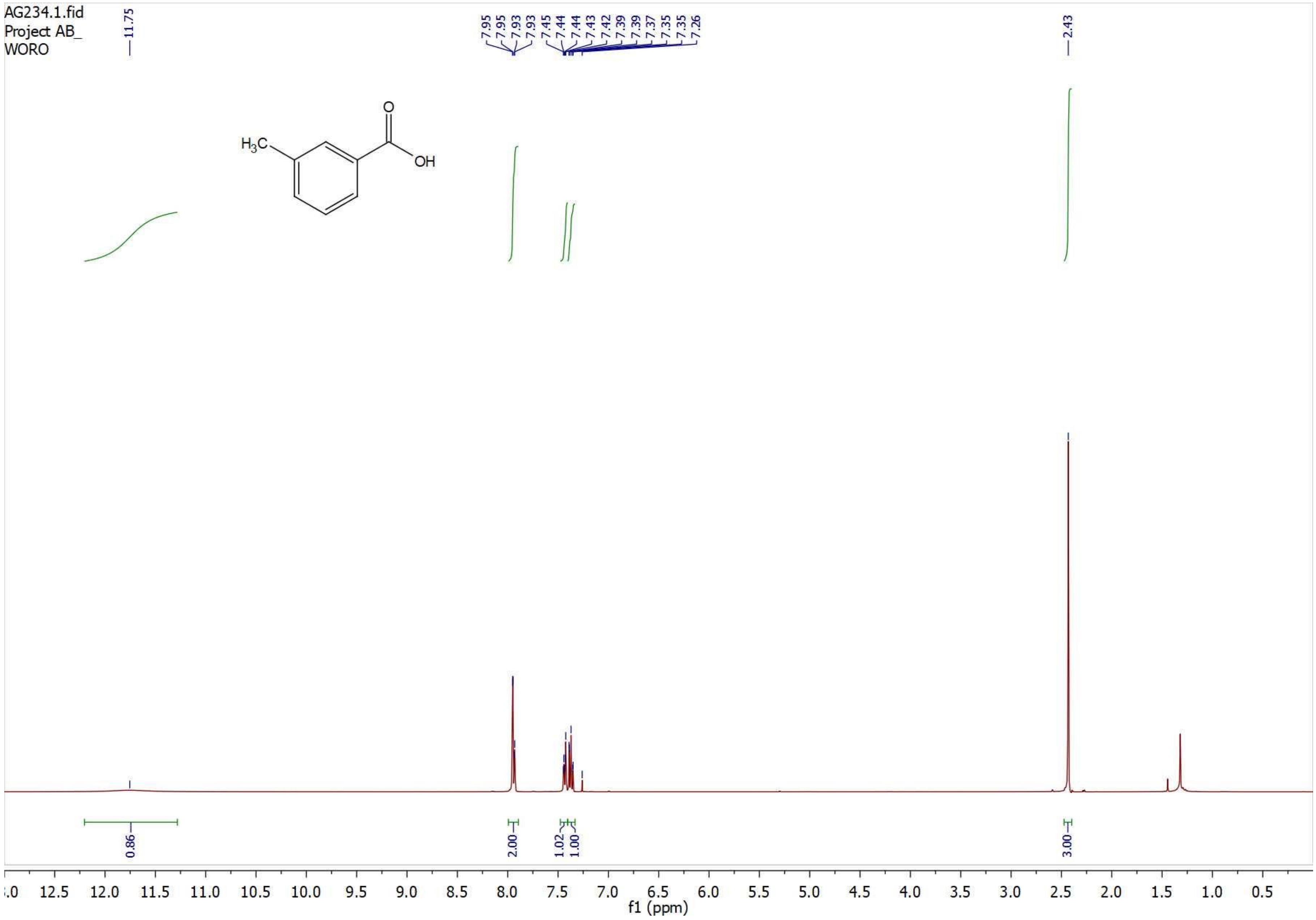

Compound p31

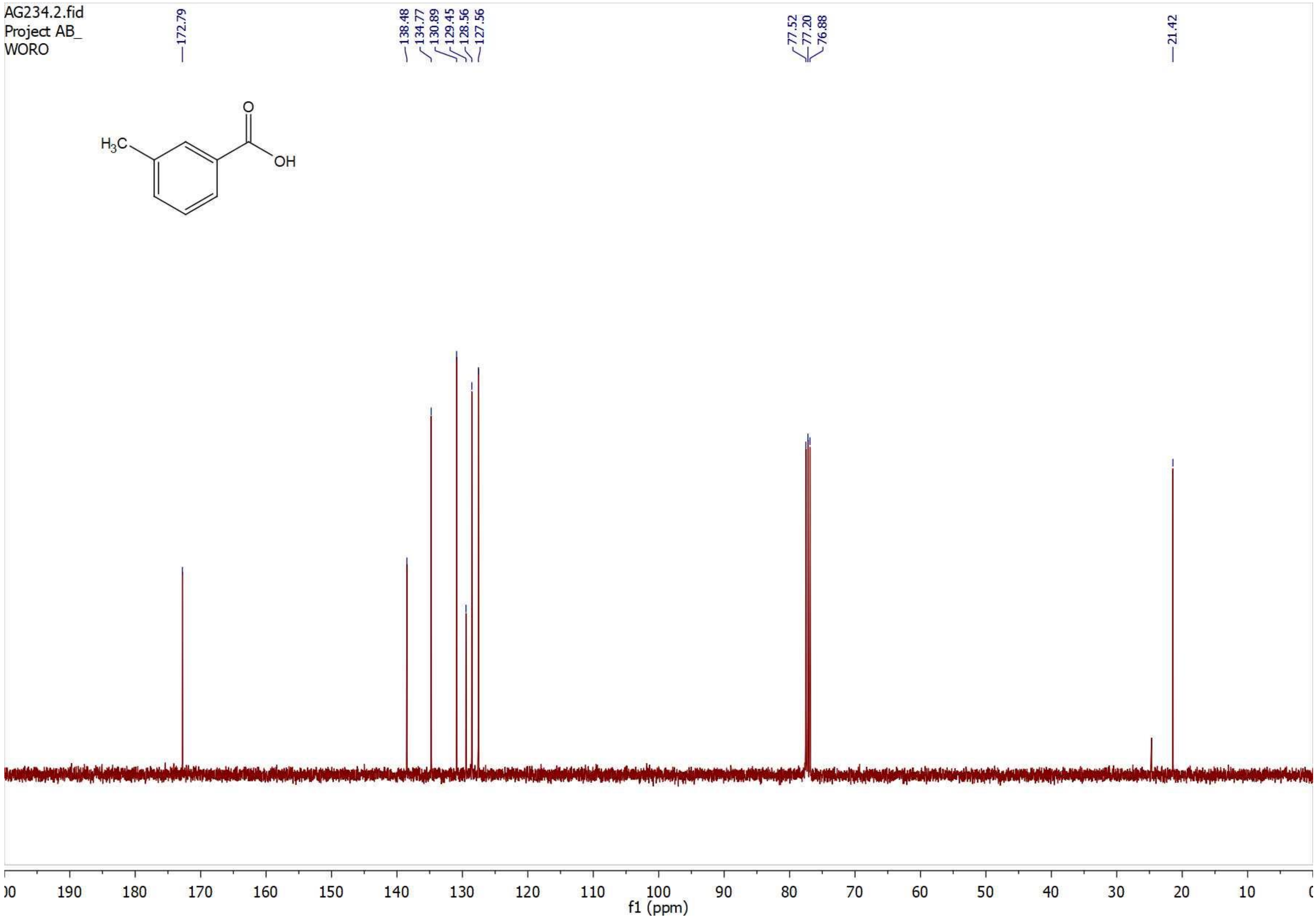

Compound p32

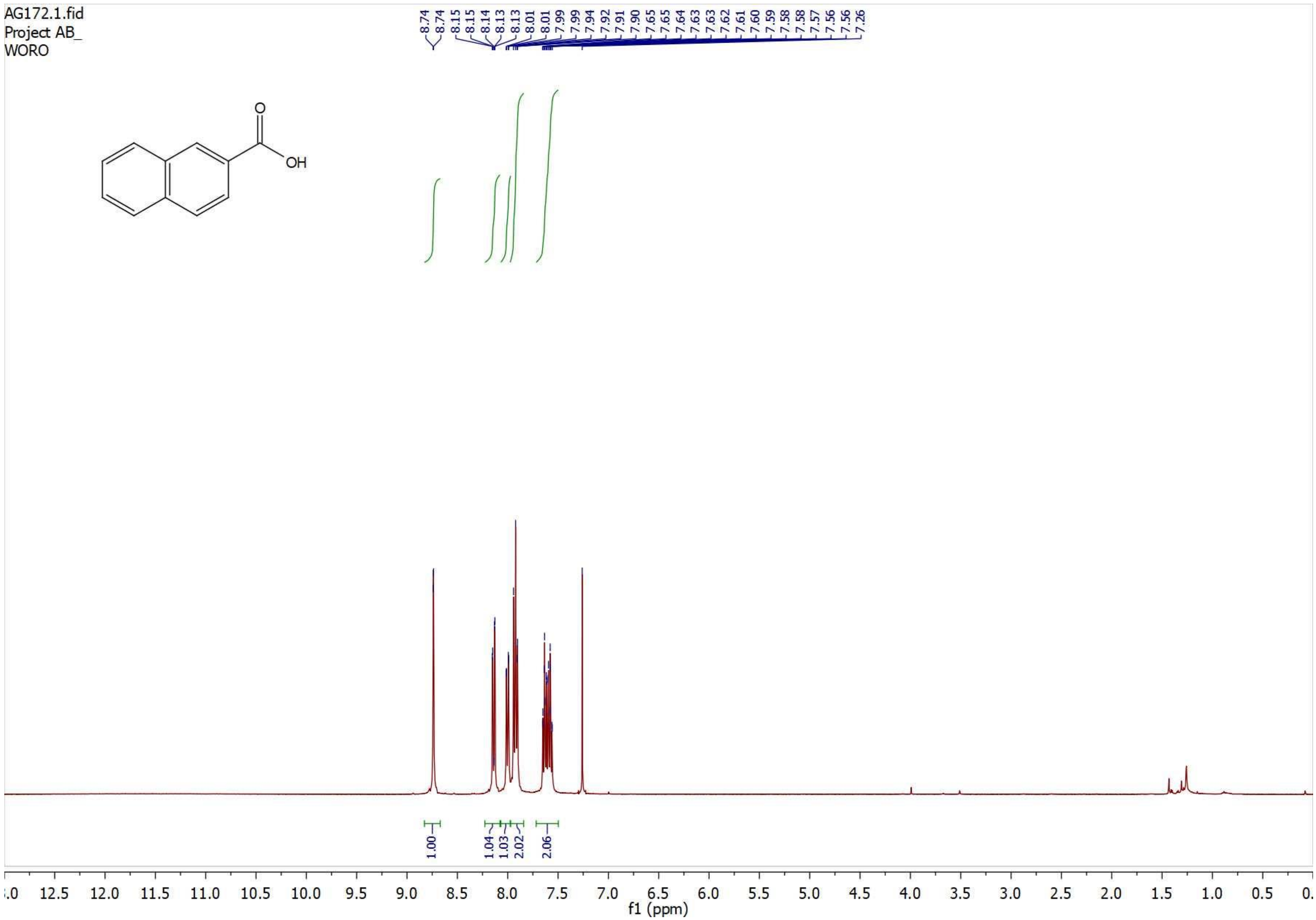

Compound p32

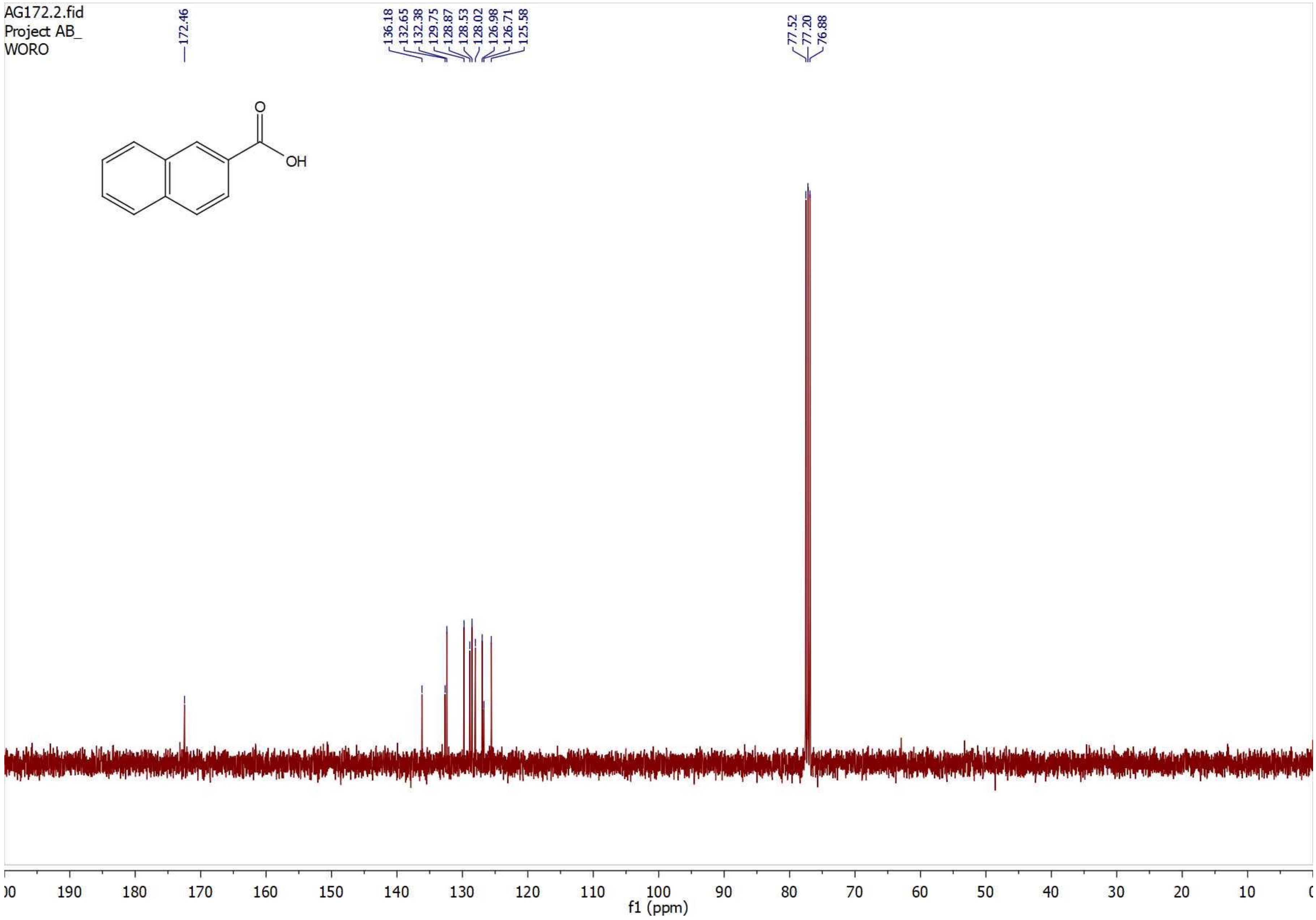

Compound p33

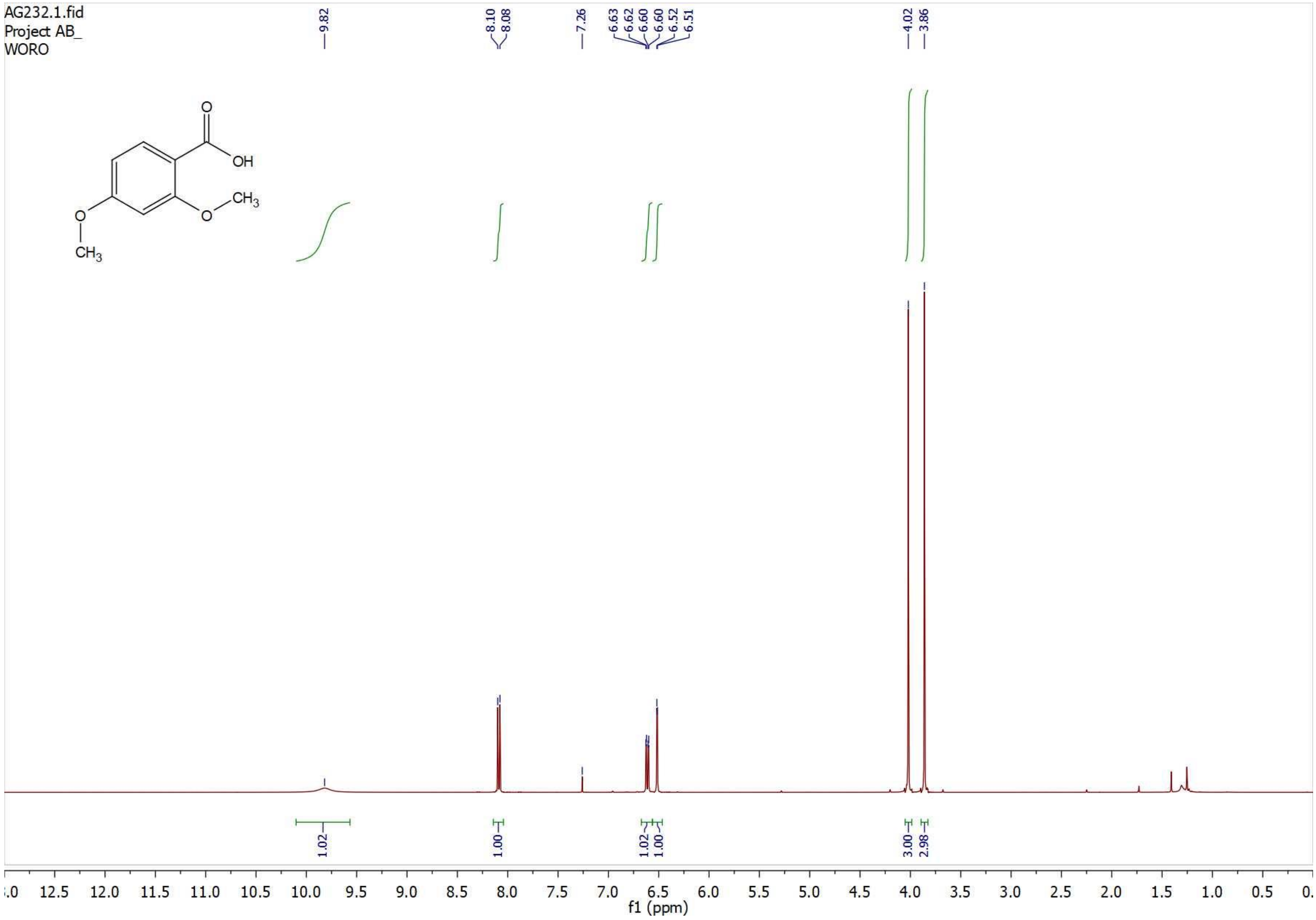

Compound p33

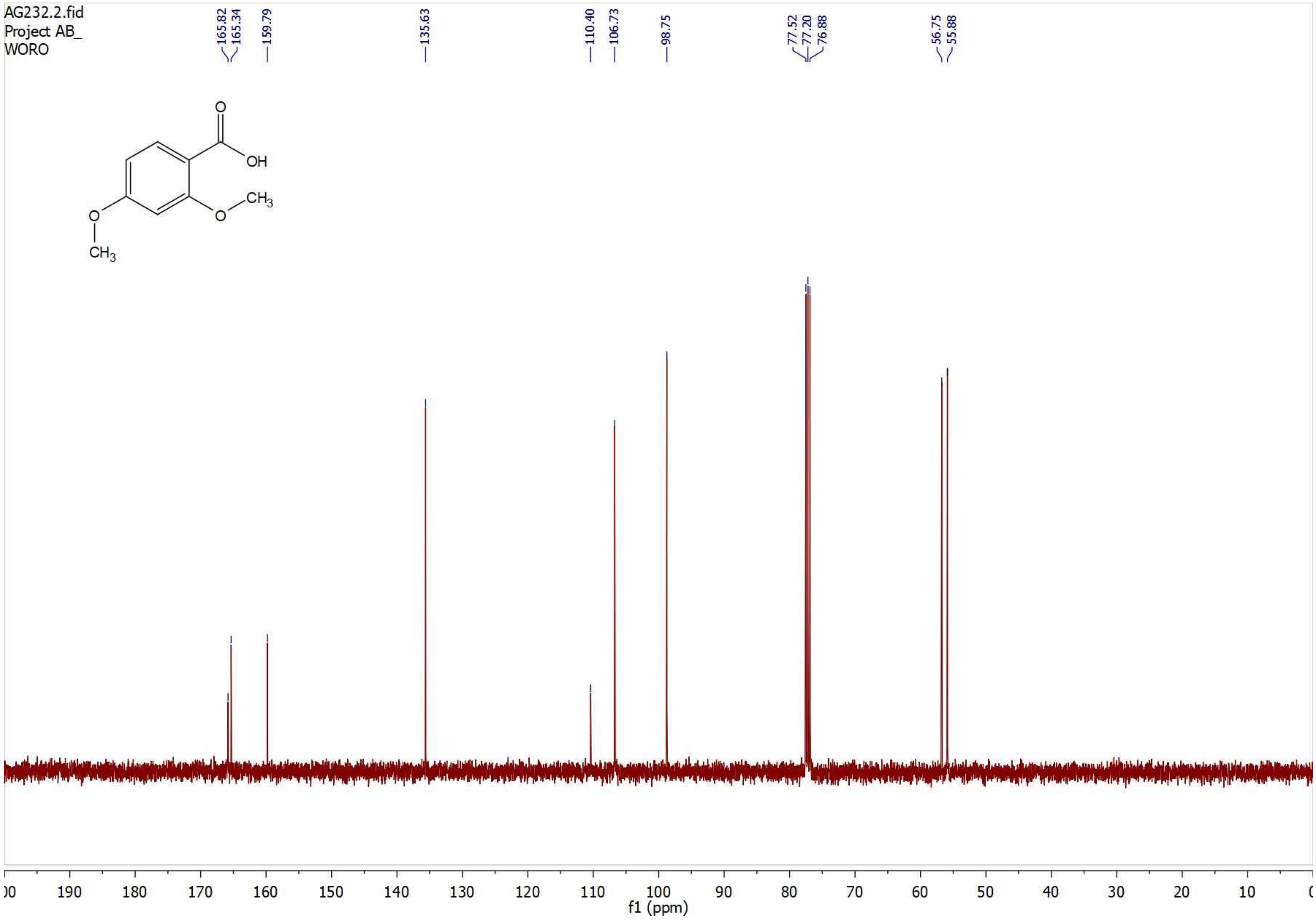

Compound p34

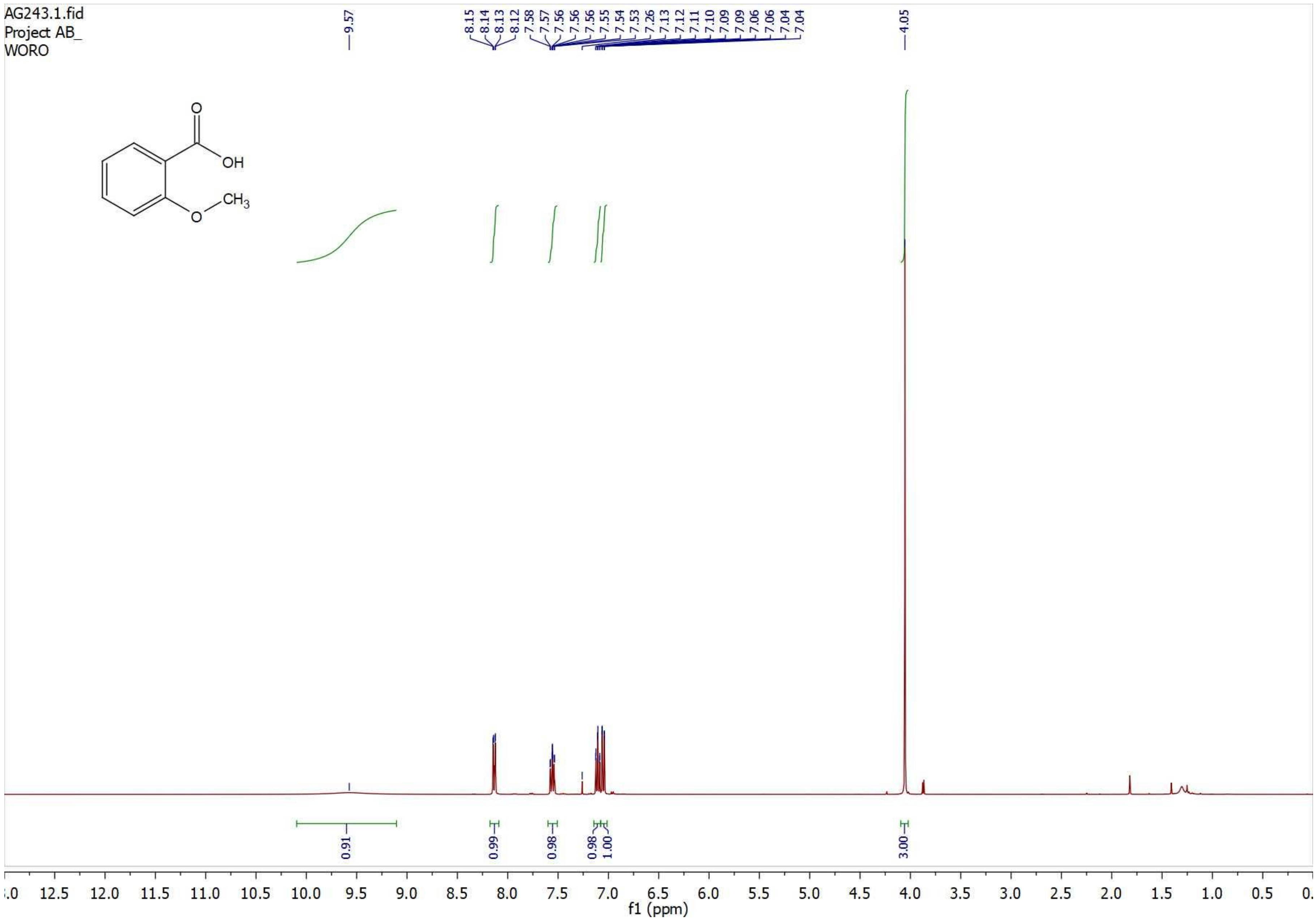

Compound p34

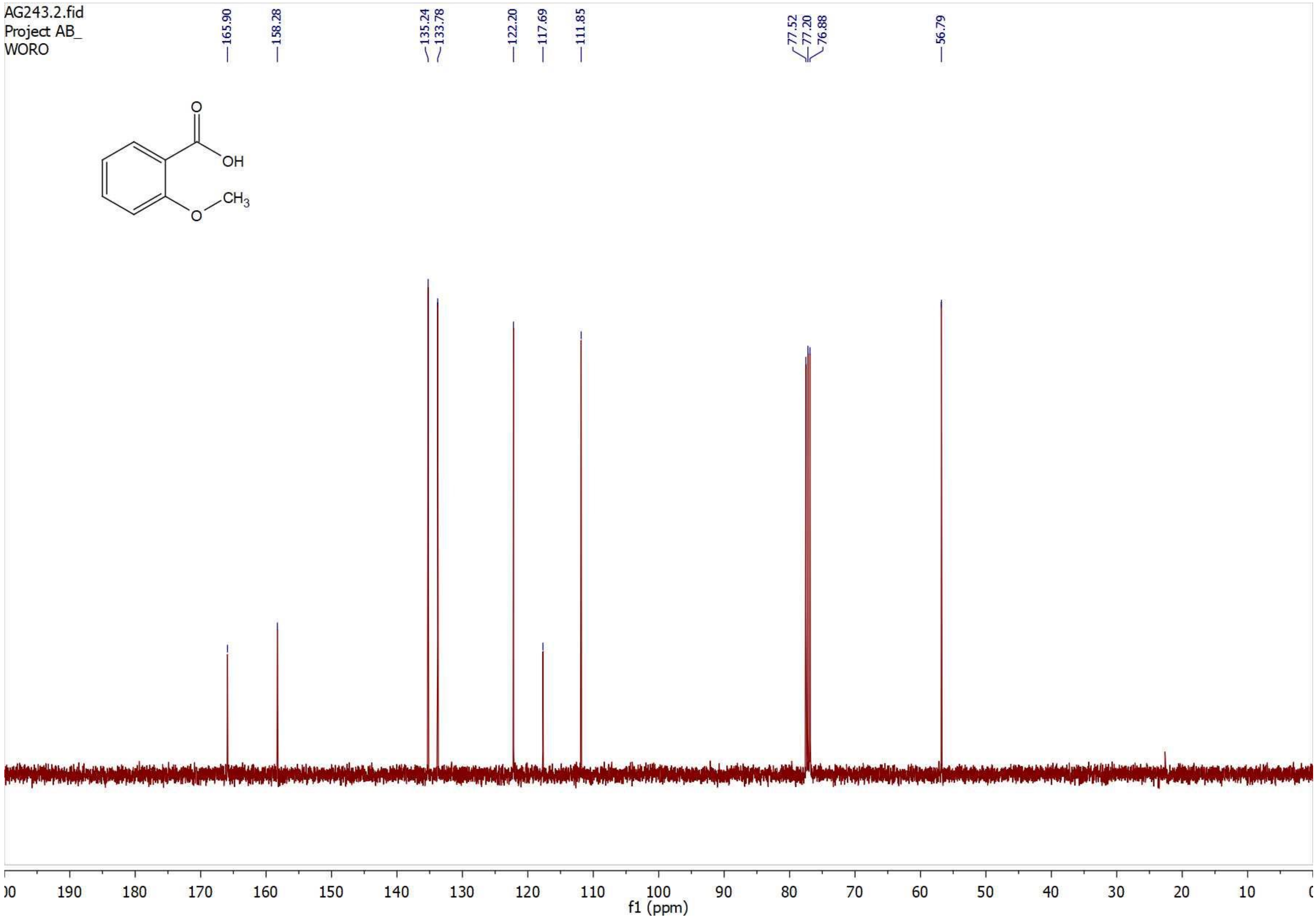

Compound p35

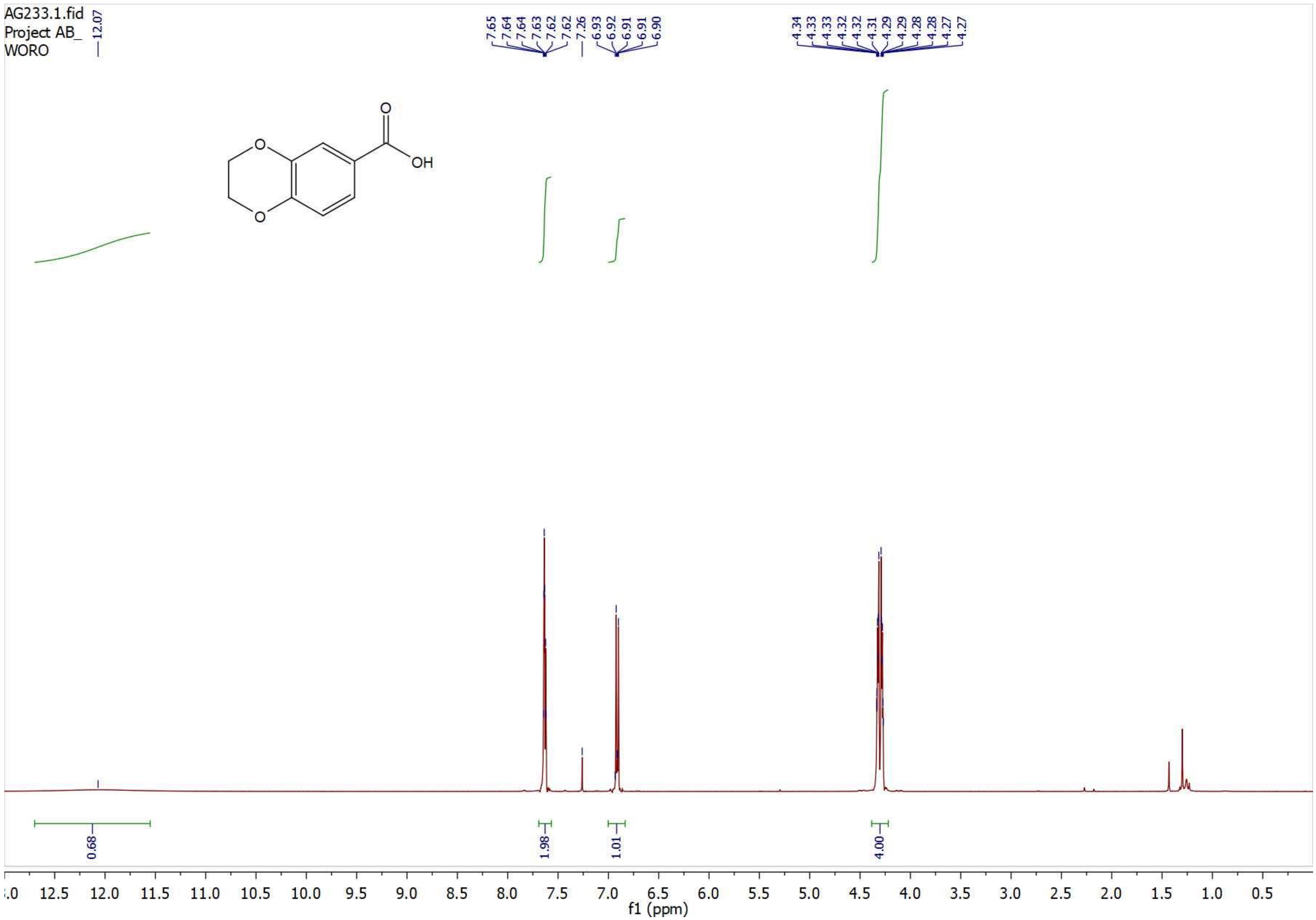

Compound p35

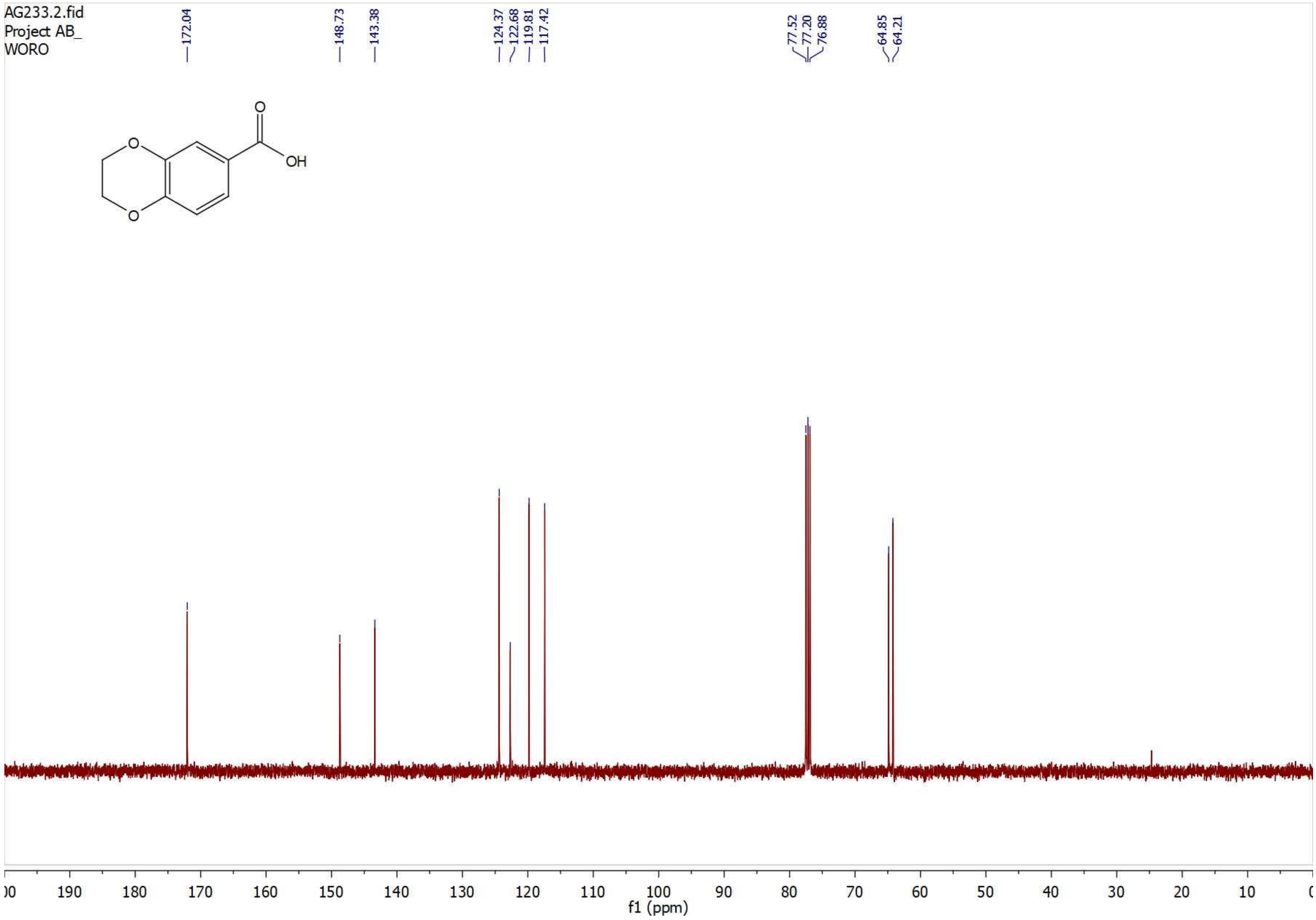

Compound p36

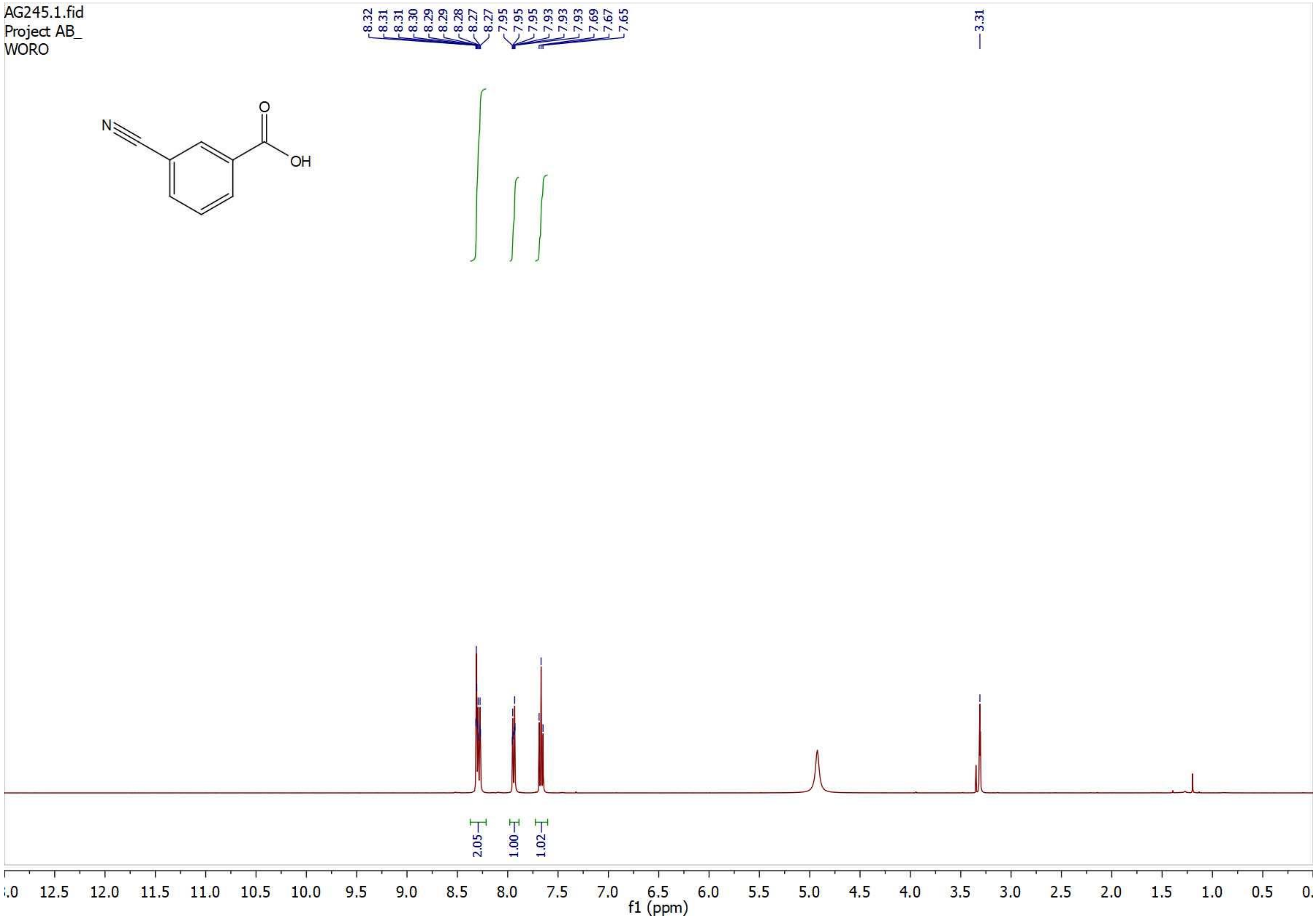

Compound p36

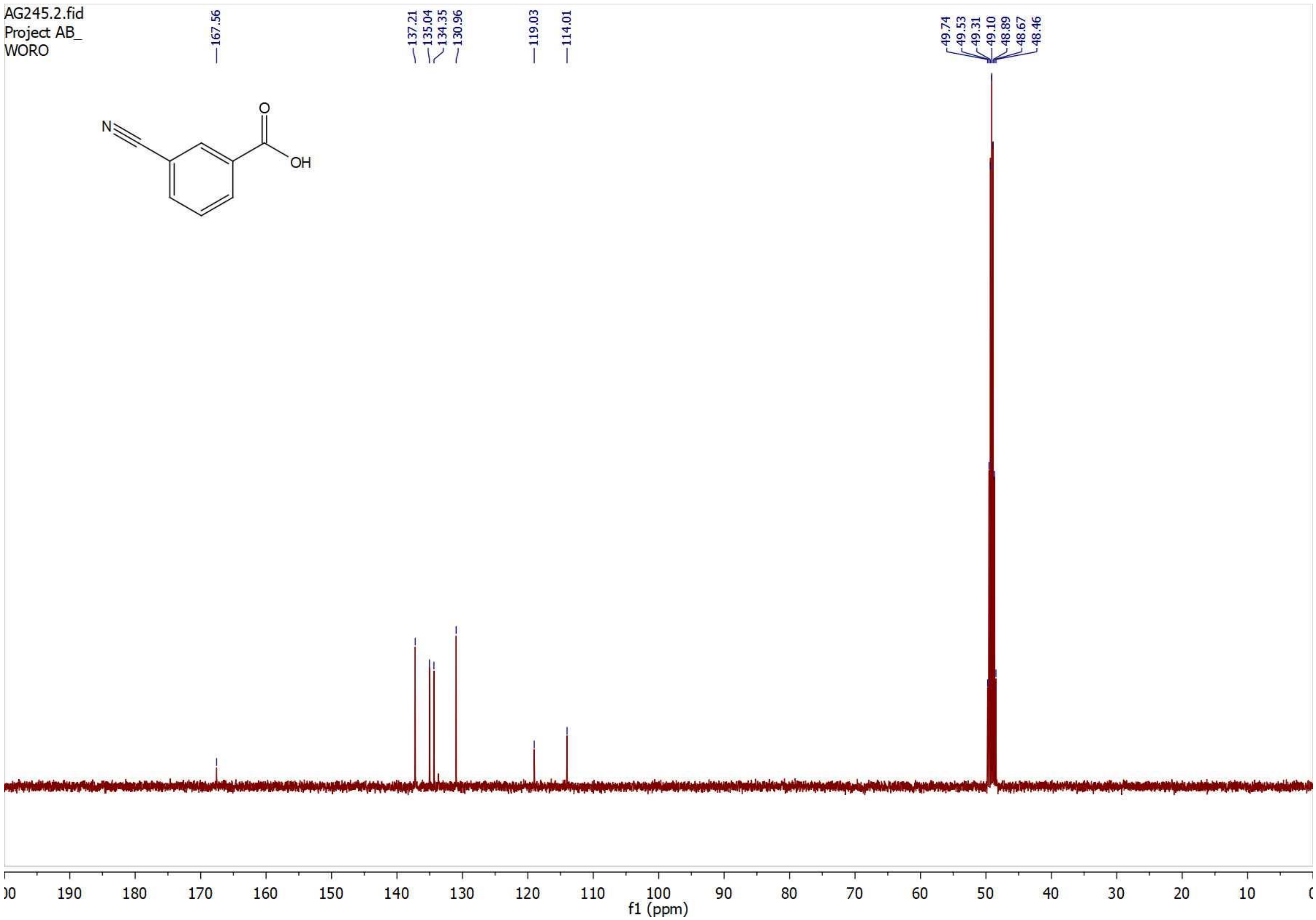

Compound p37

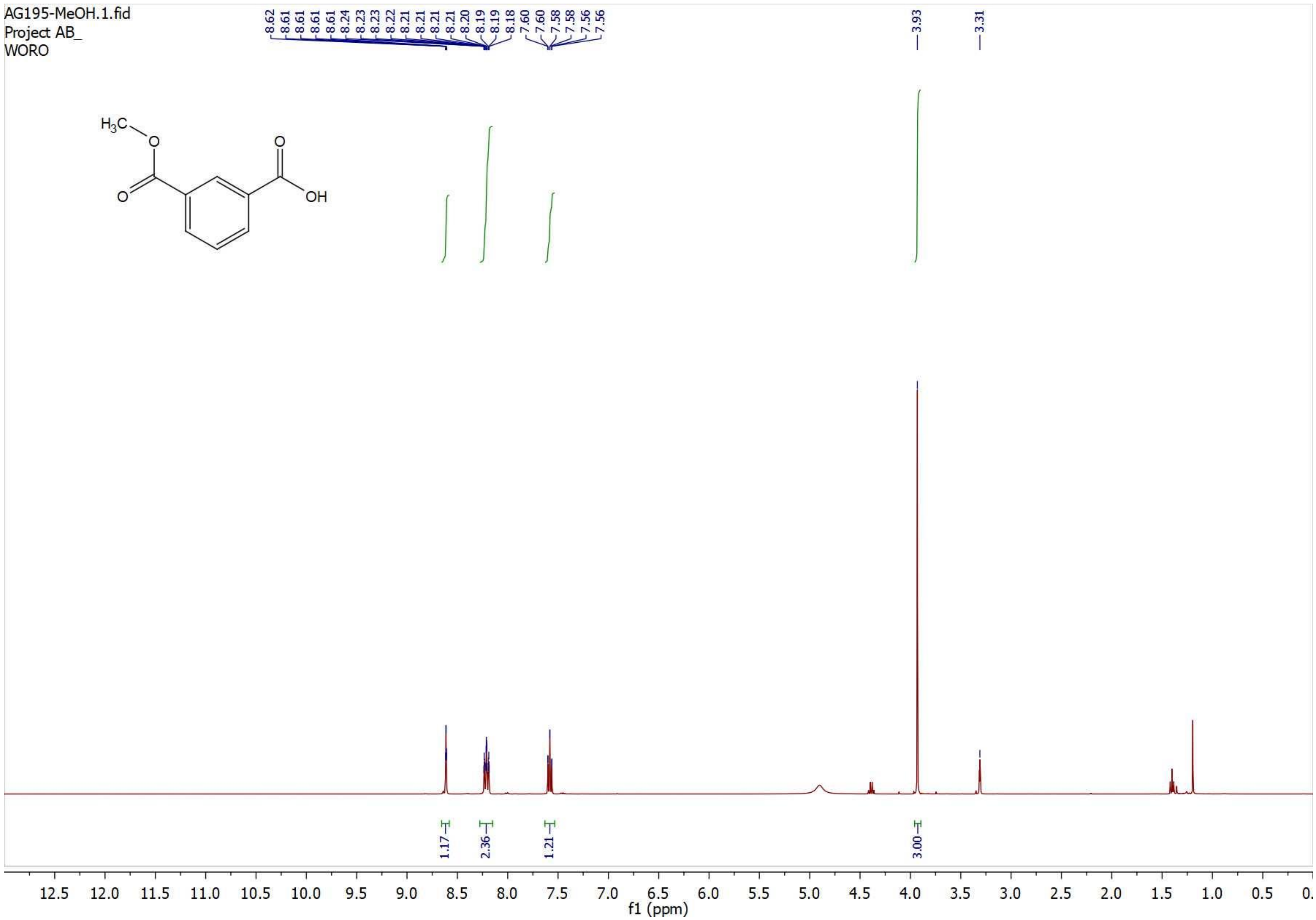

Compound p37

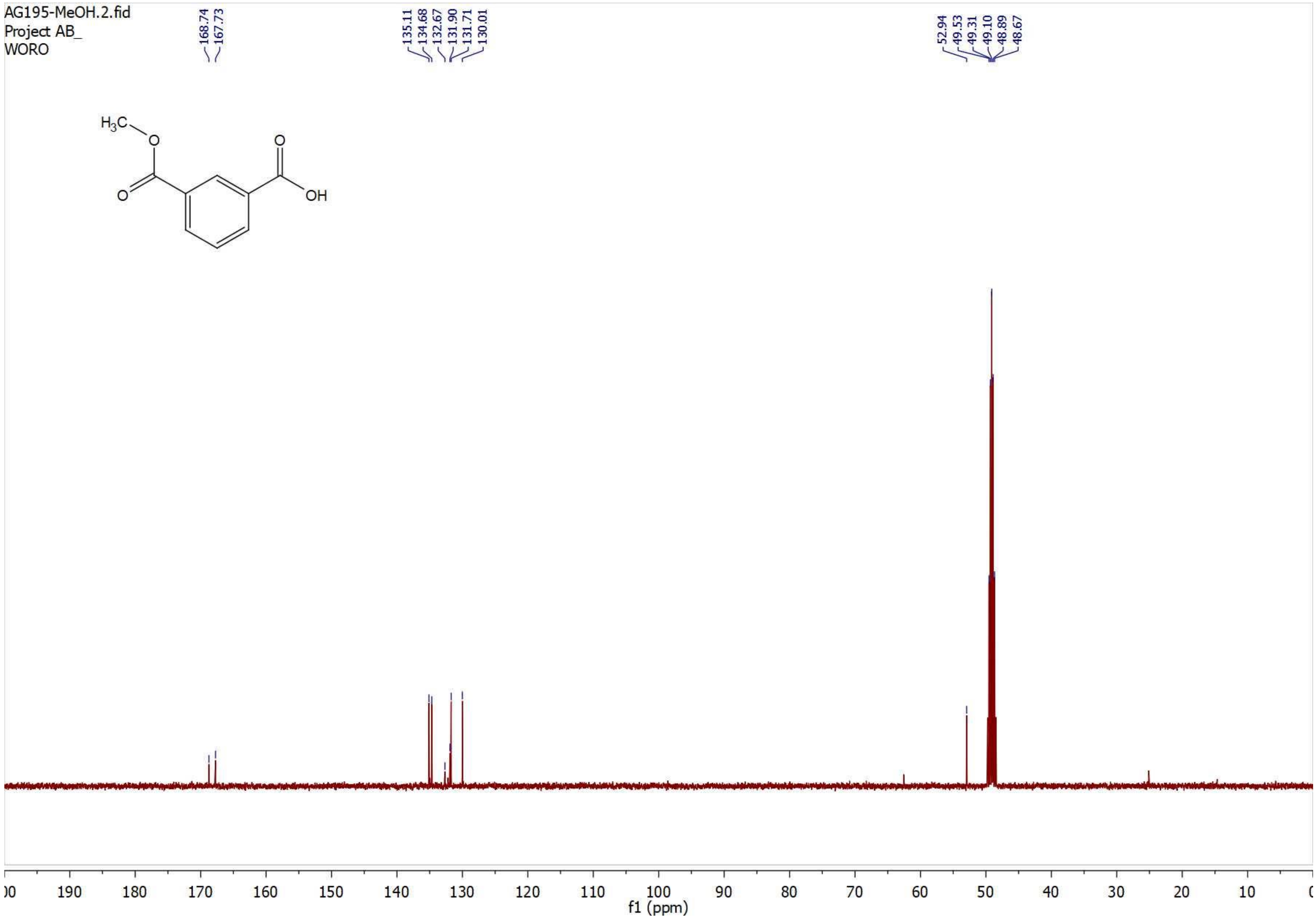

Compound p38

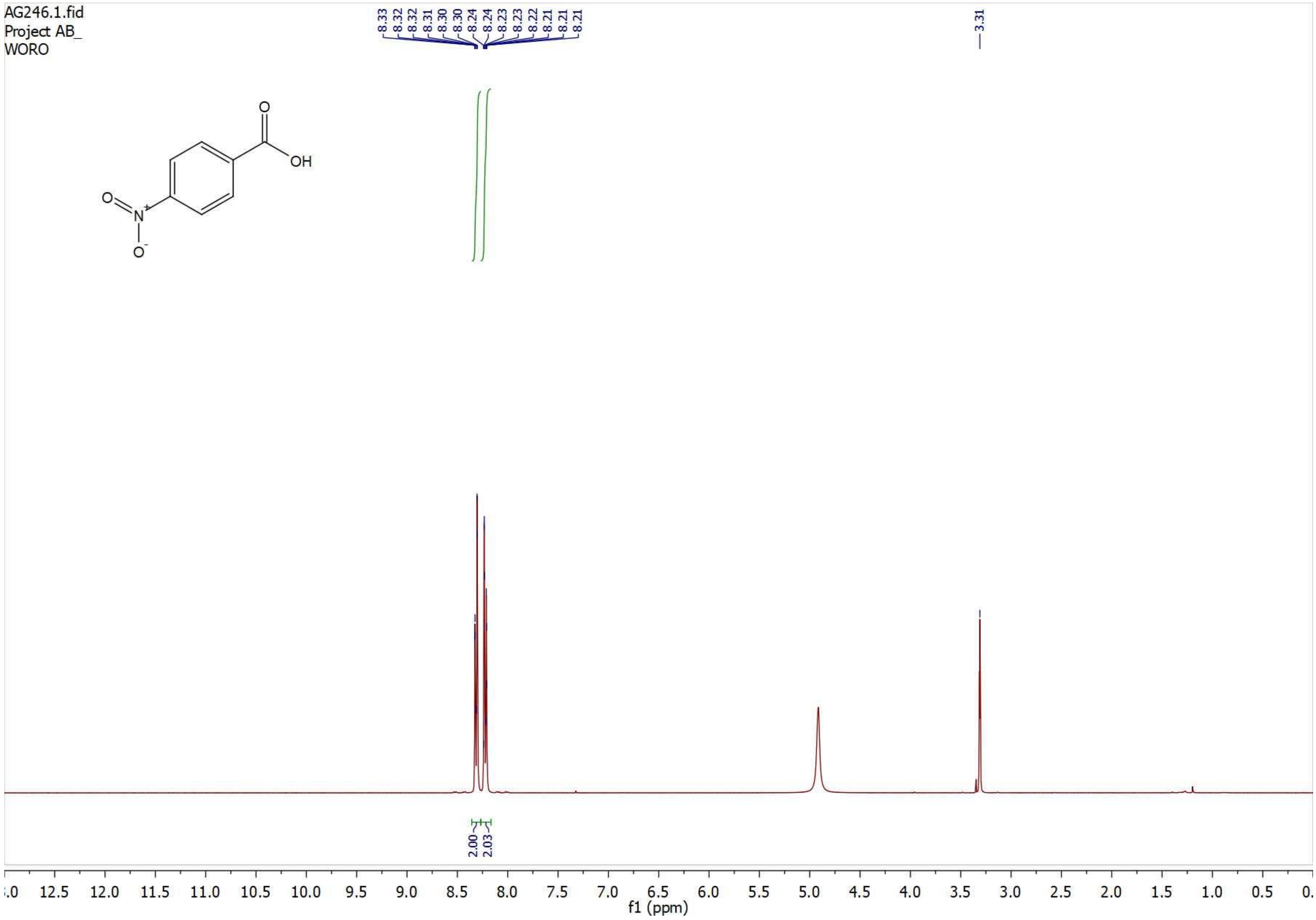

Compound p38

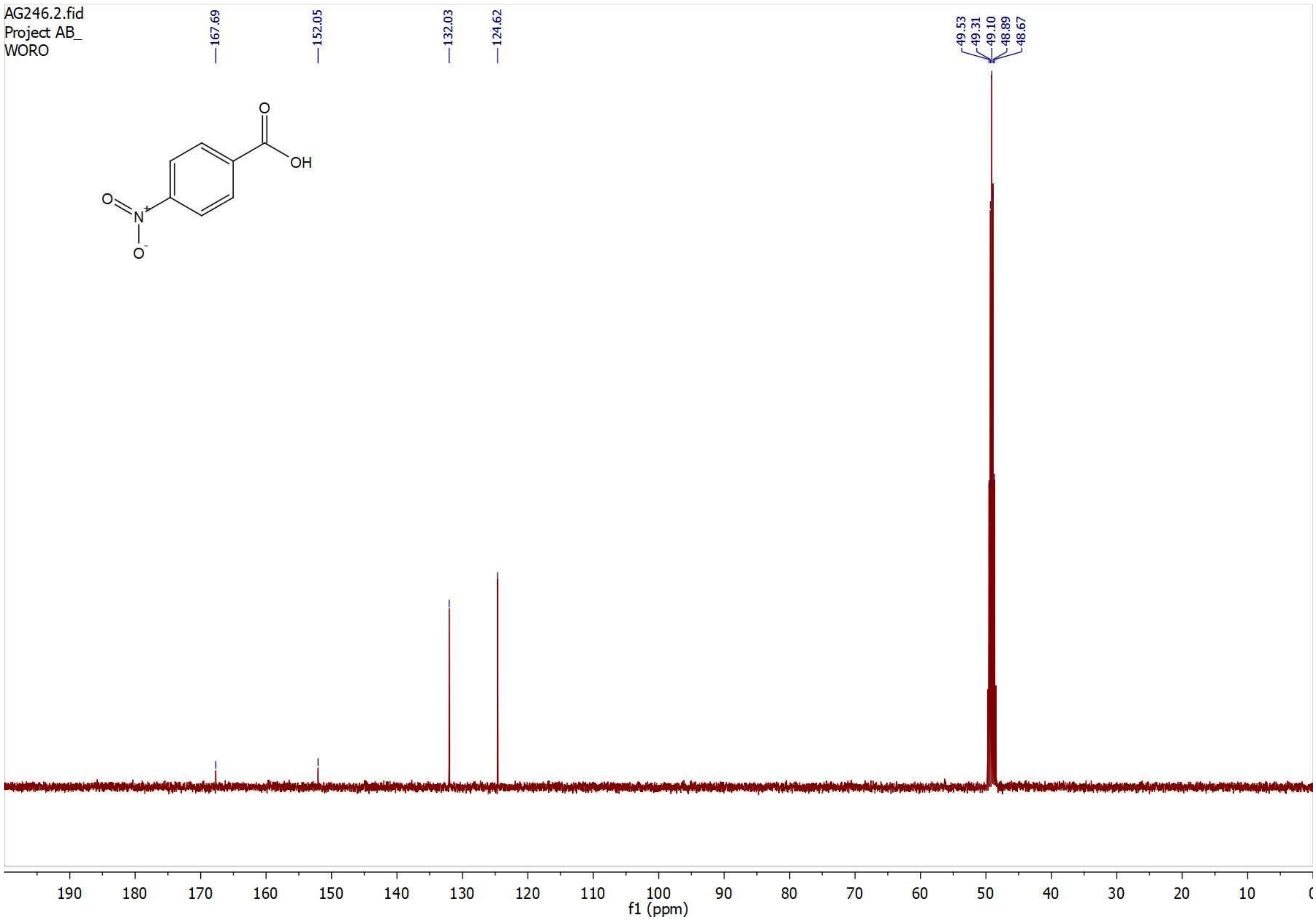

Compound p39

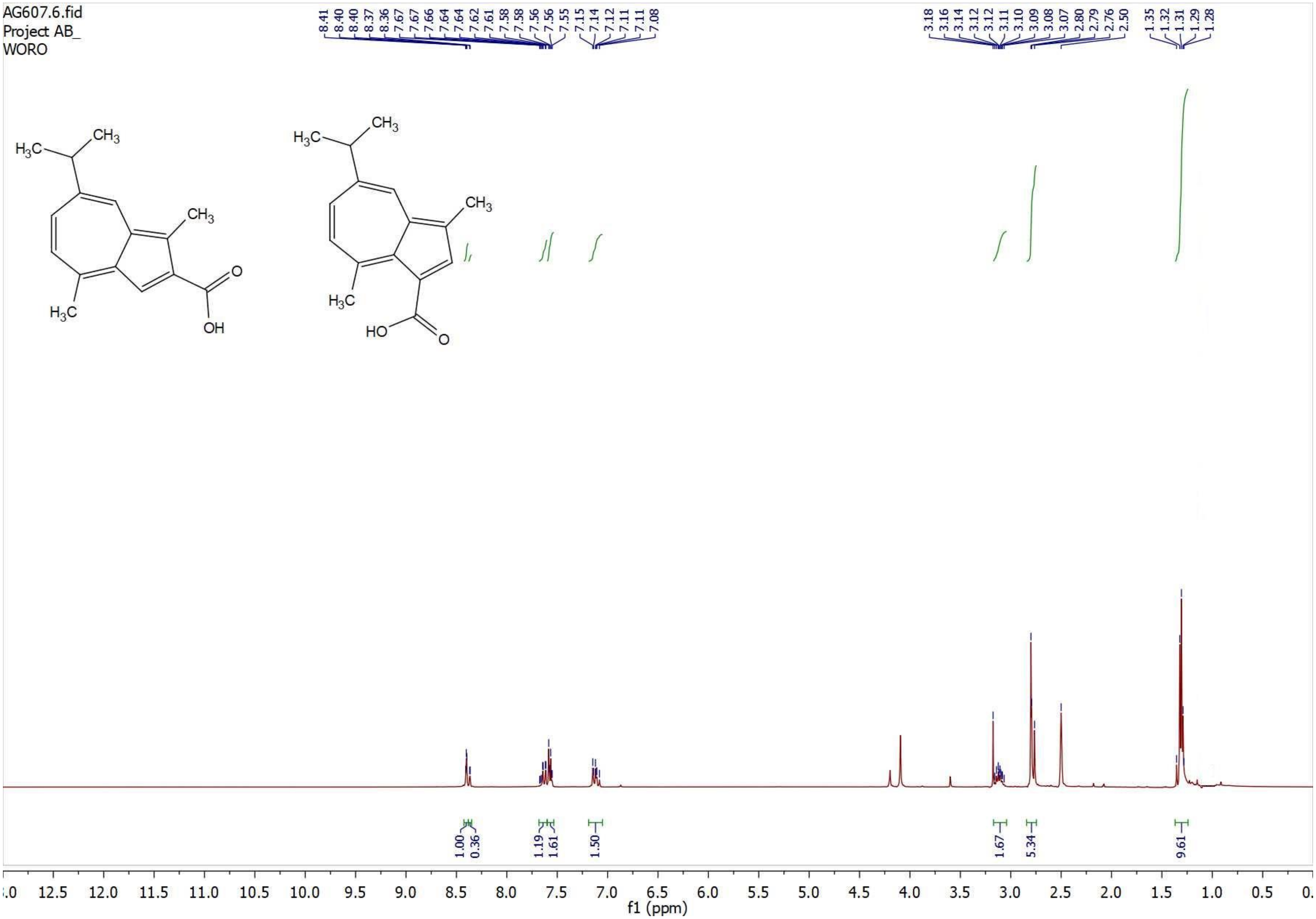

Compound p39

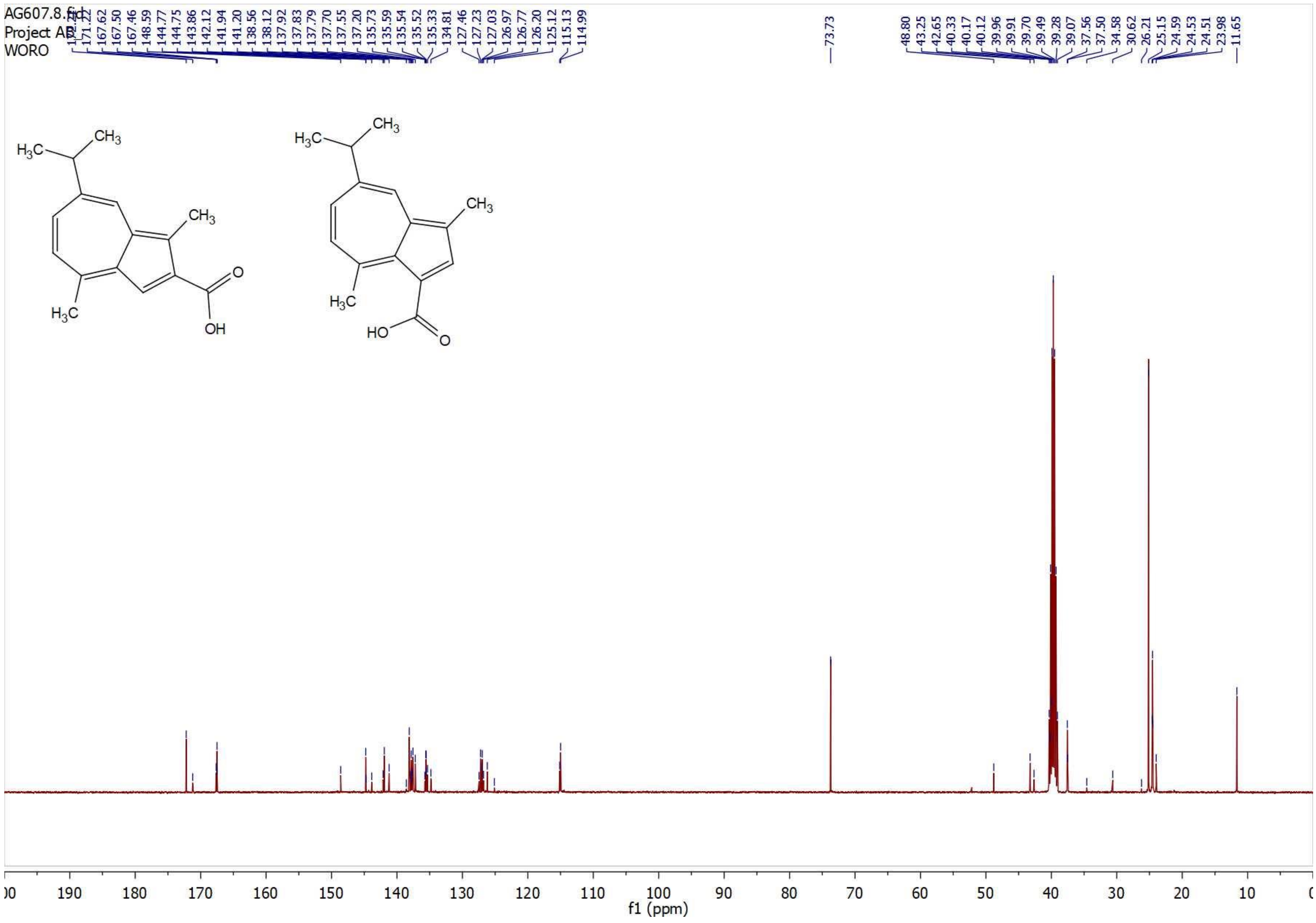

Compound p40

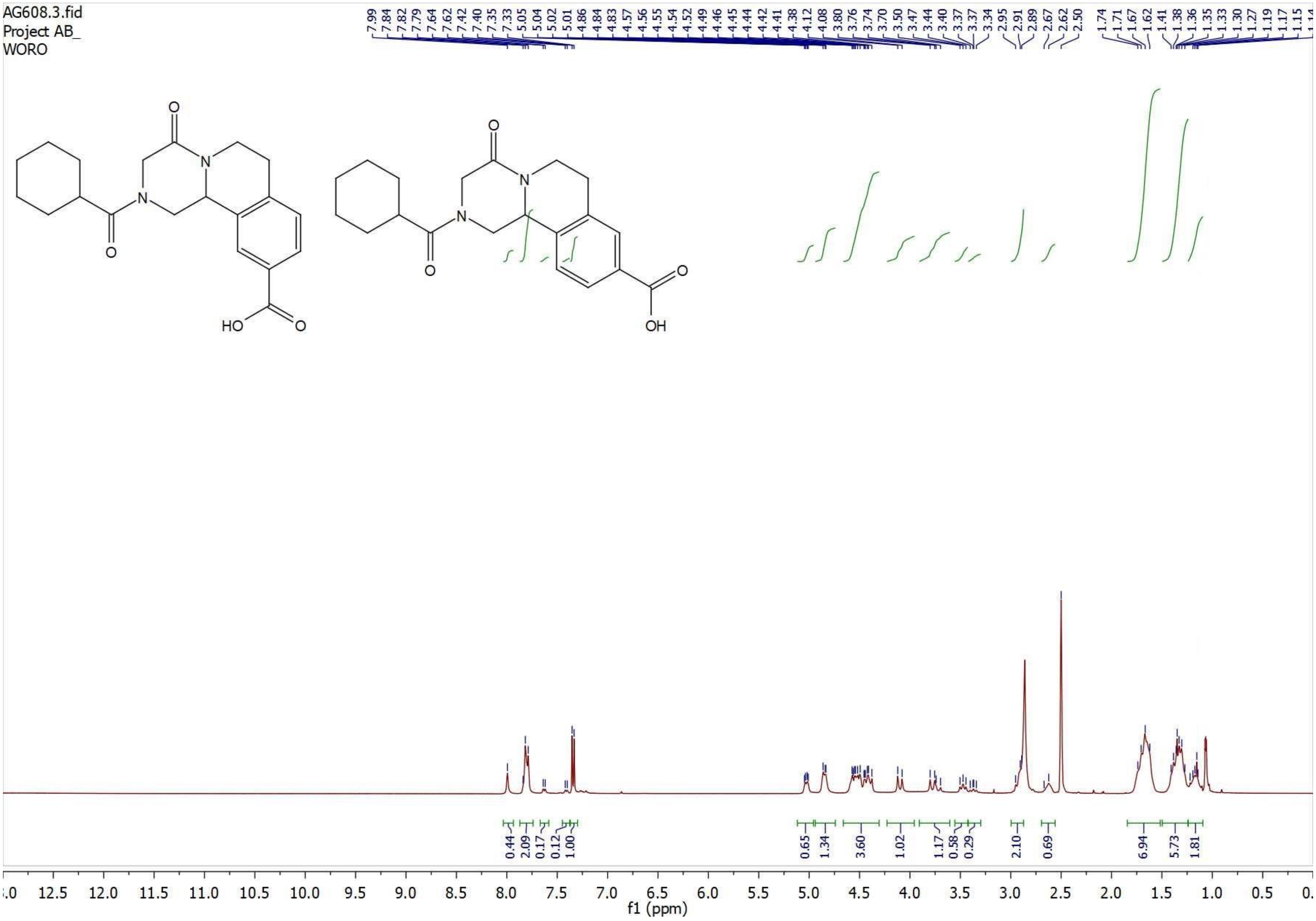

Compound p40

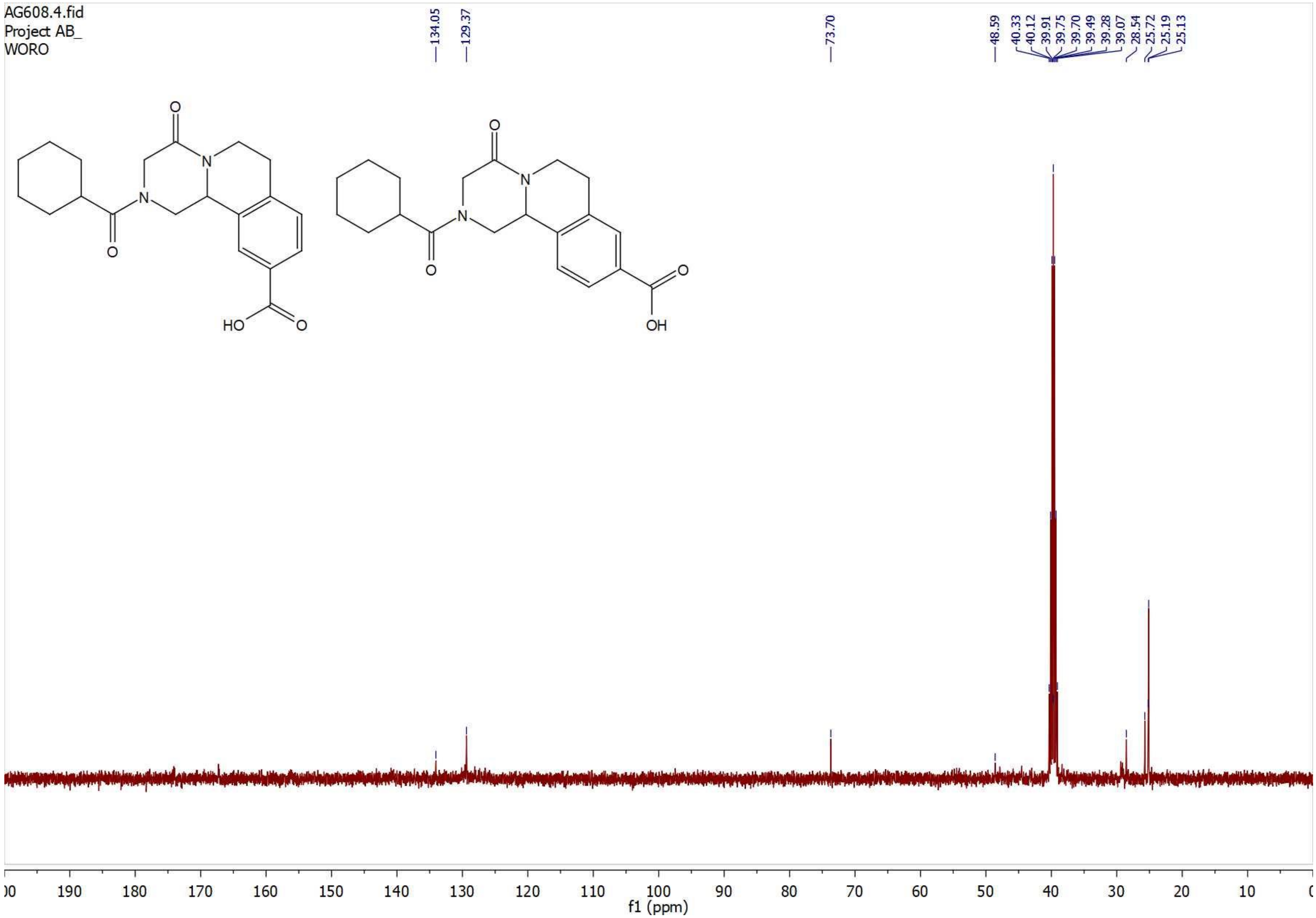

Compound p41

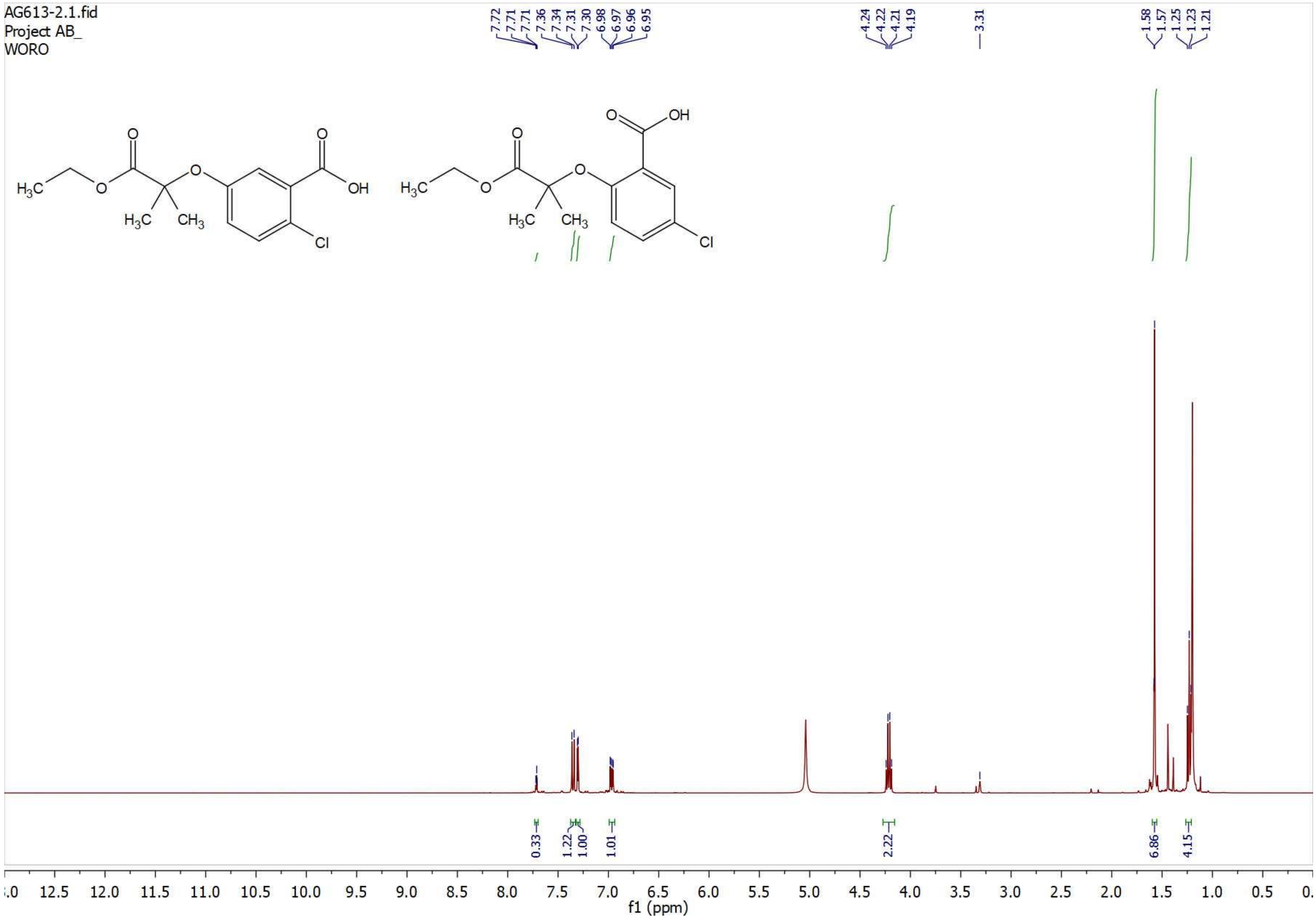

Compound p41

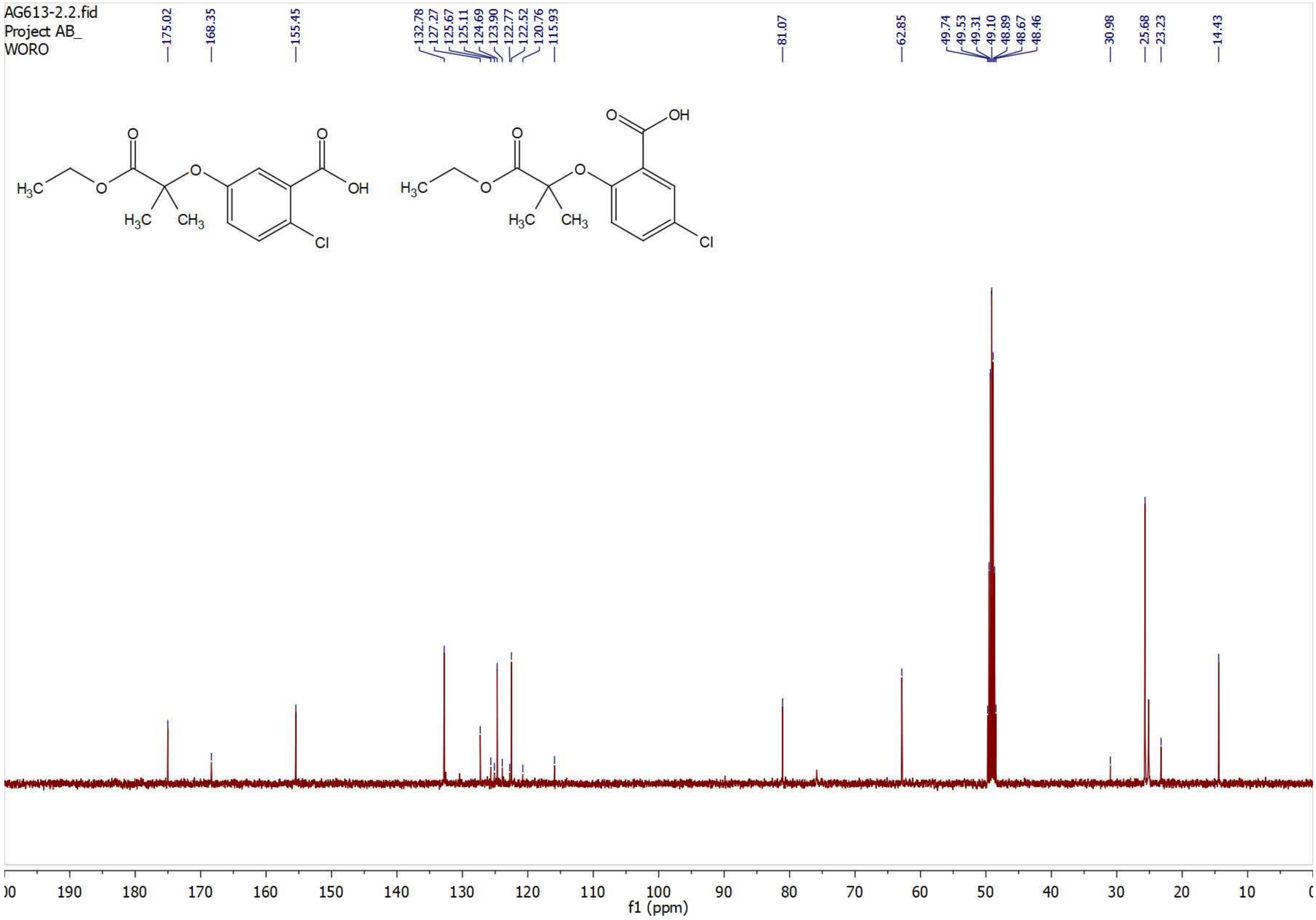

Compound p42

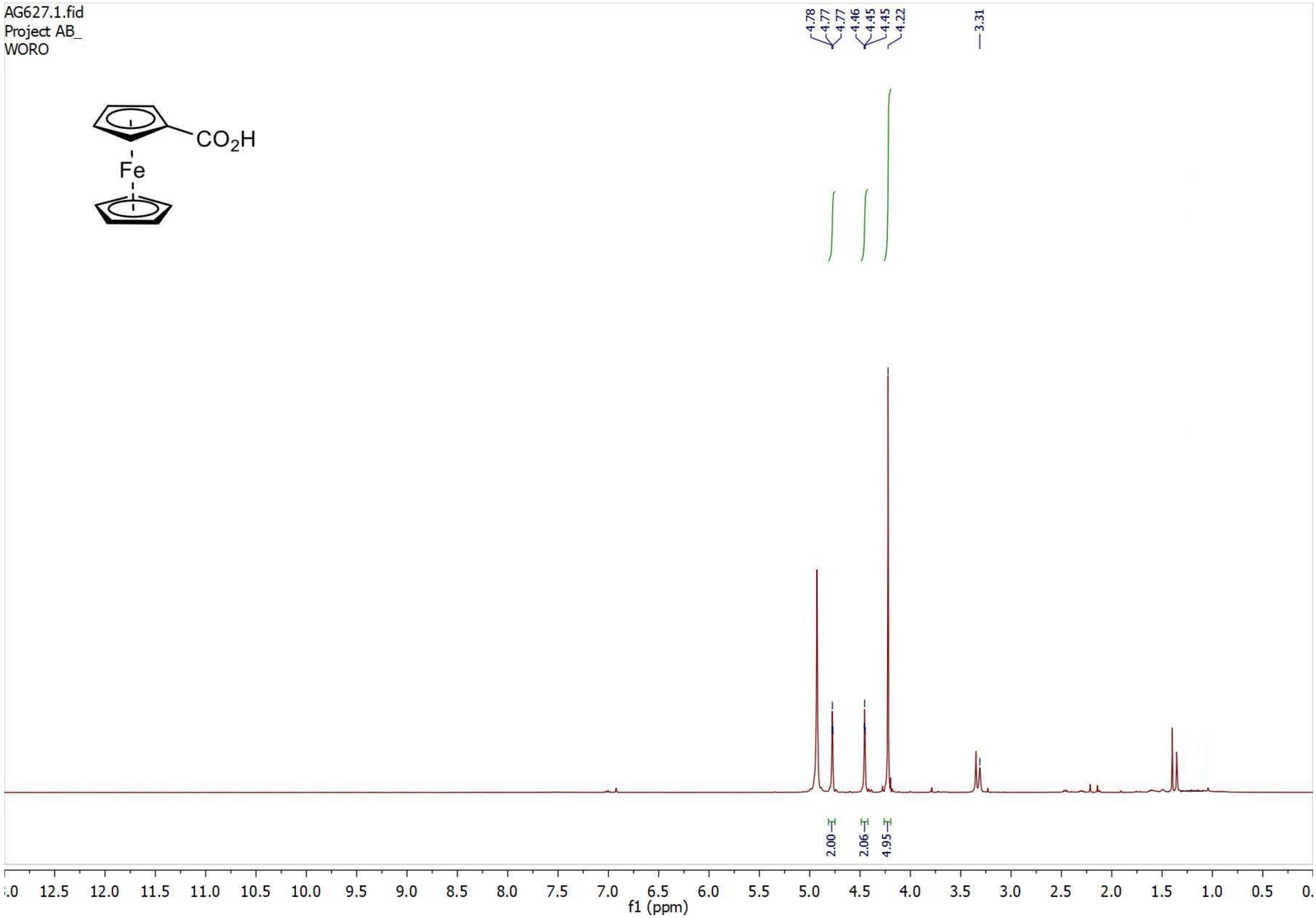

Compound p42

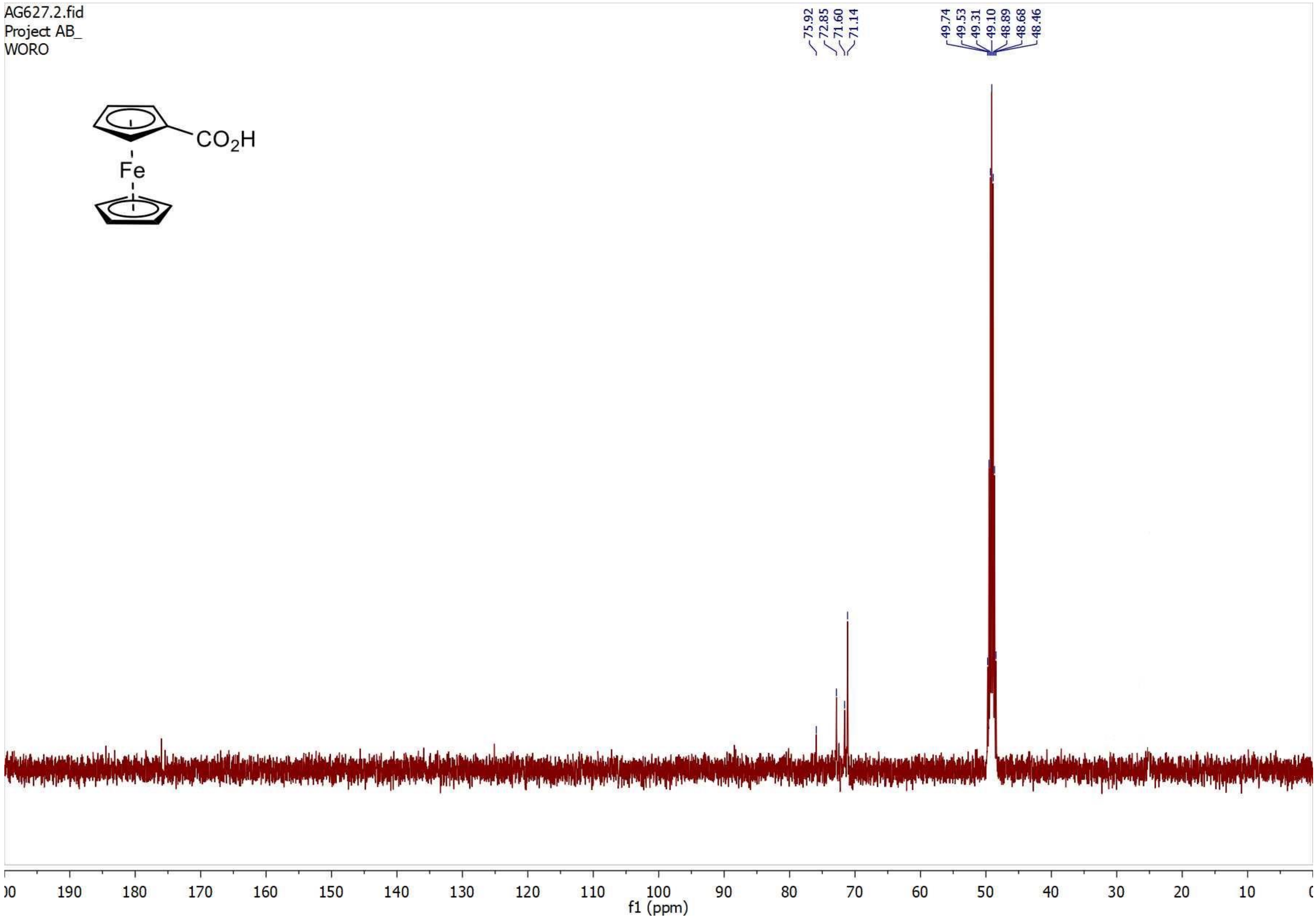

Compound p43

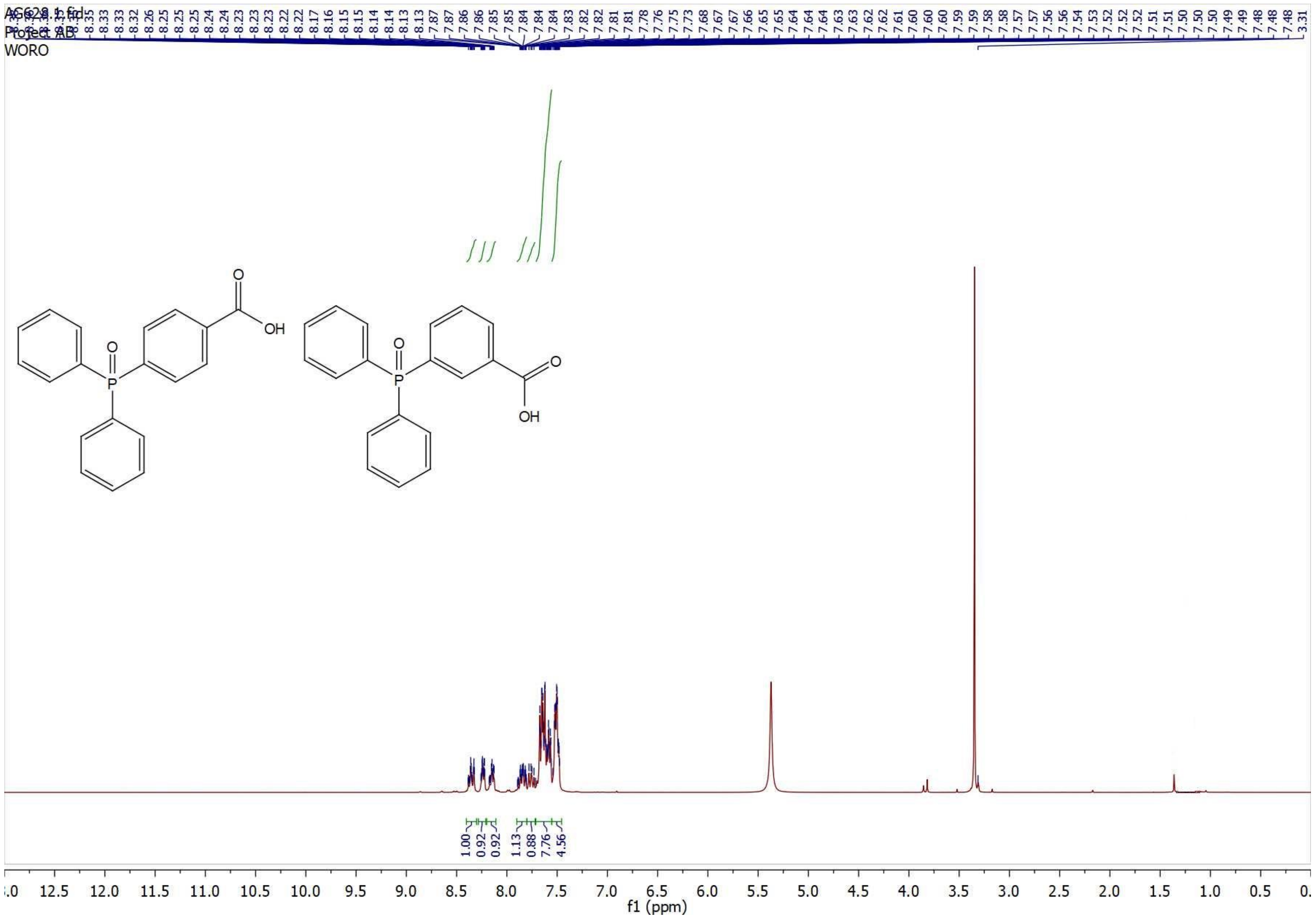

Compound p43

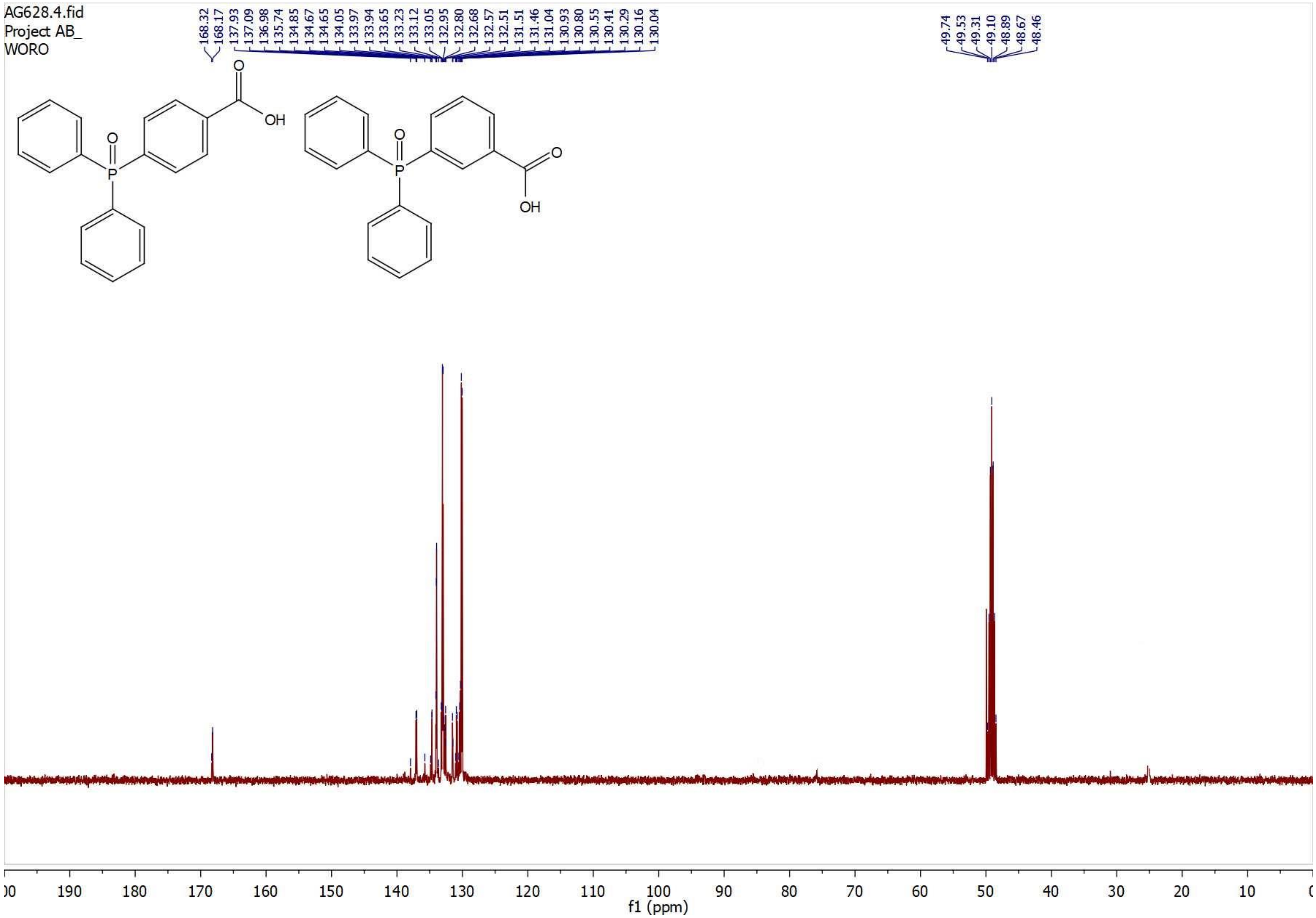

Compound p43

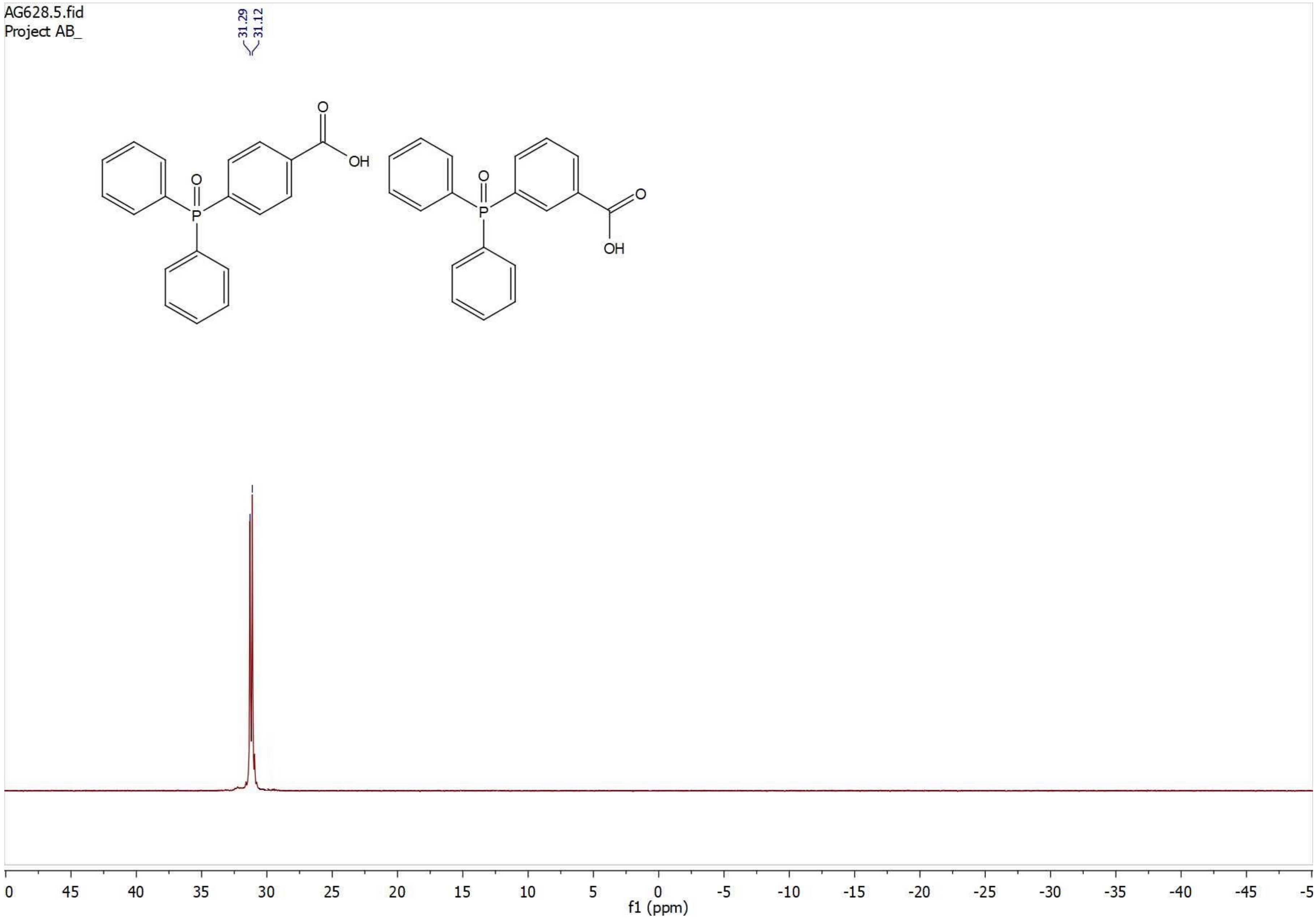

Compound p44

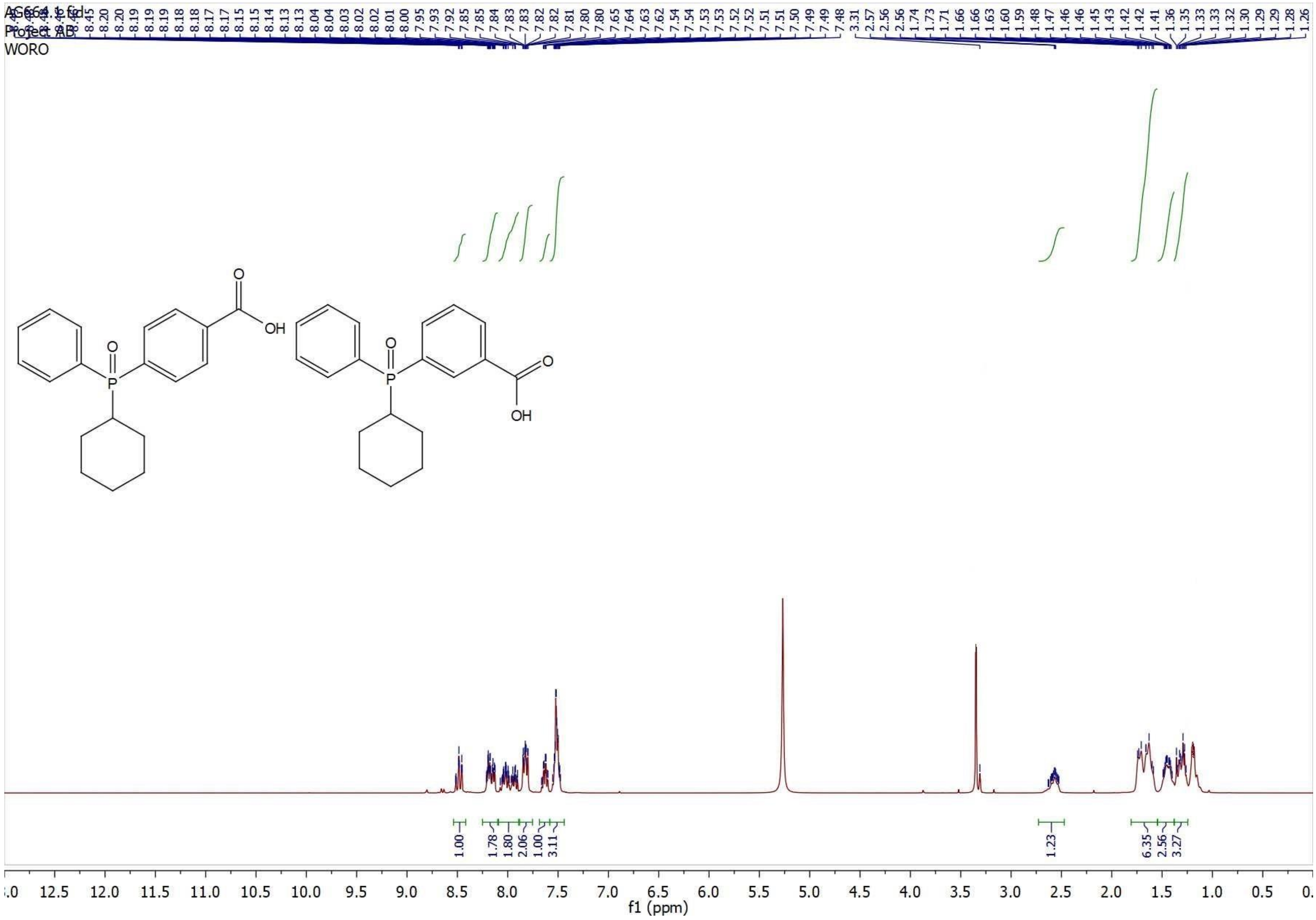

Compound p44

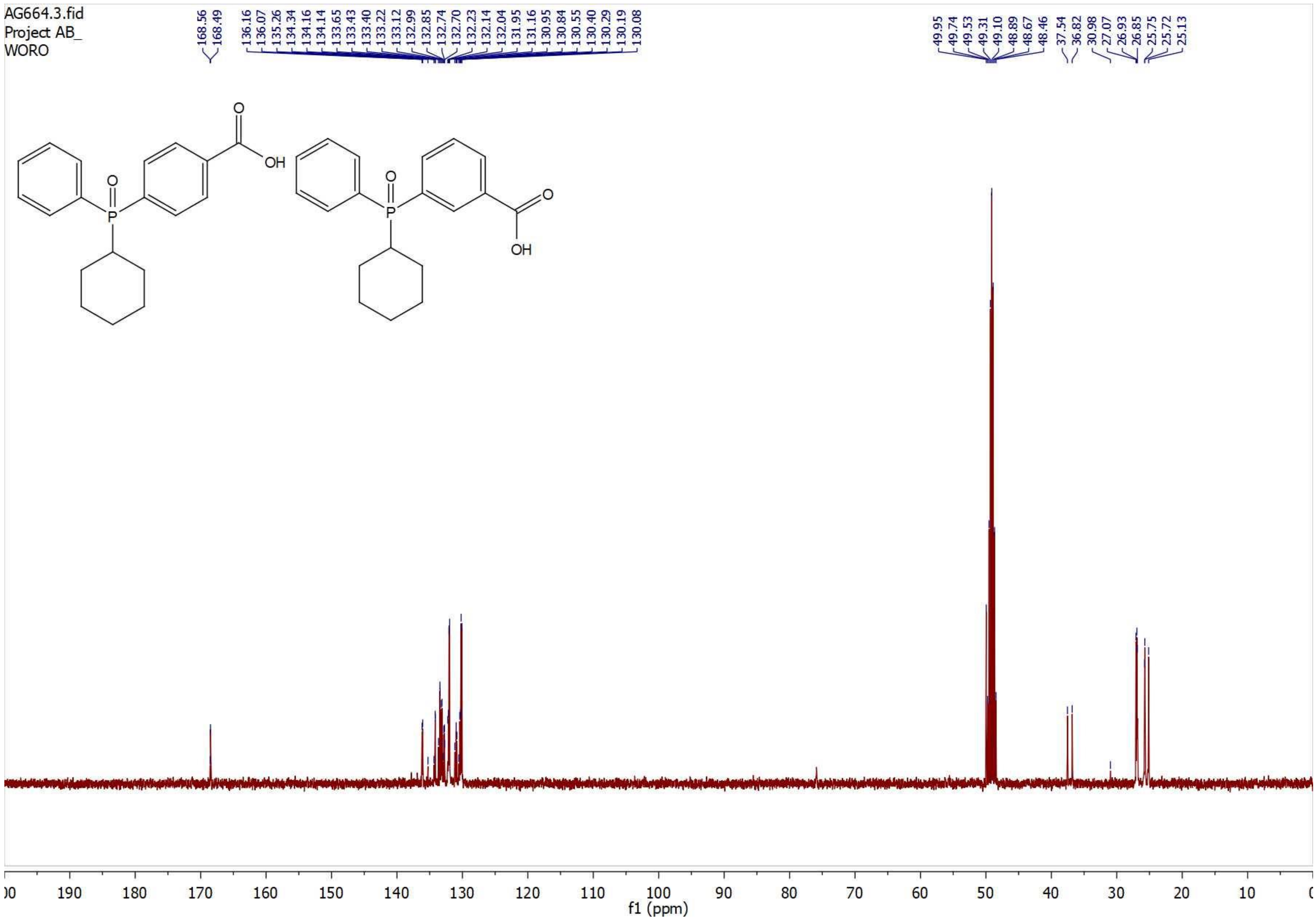

Compound p44

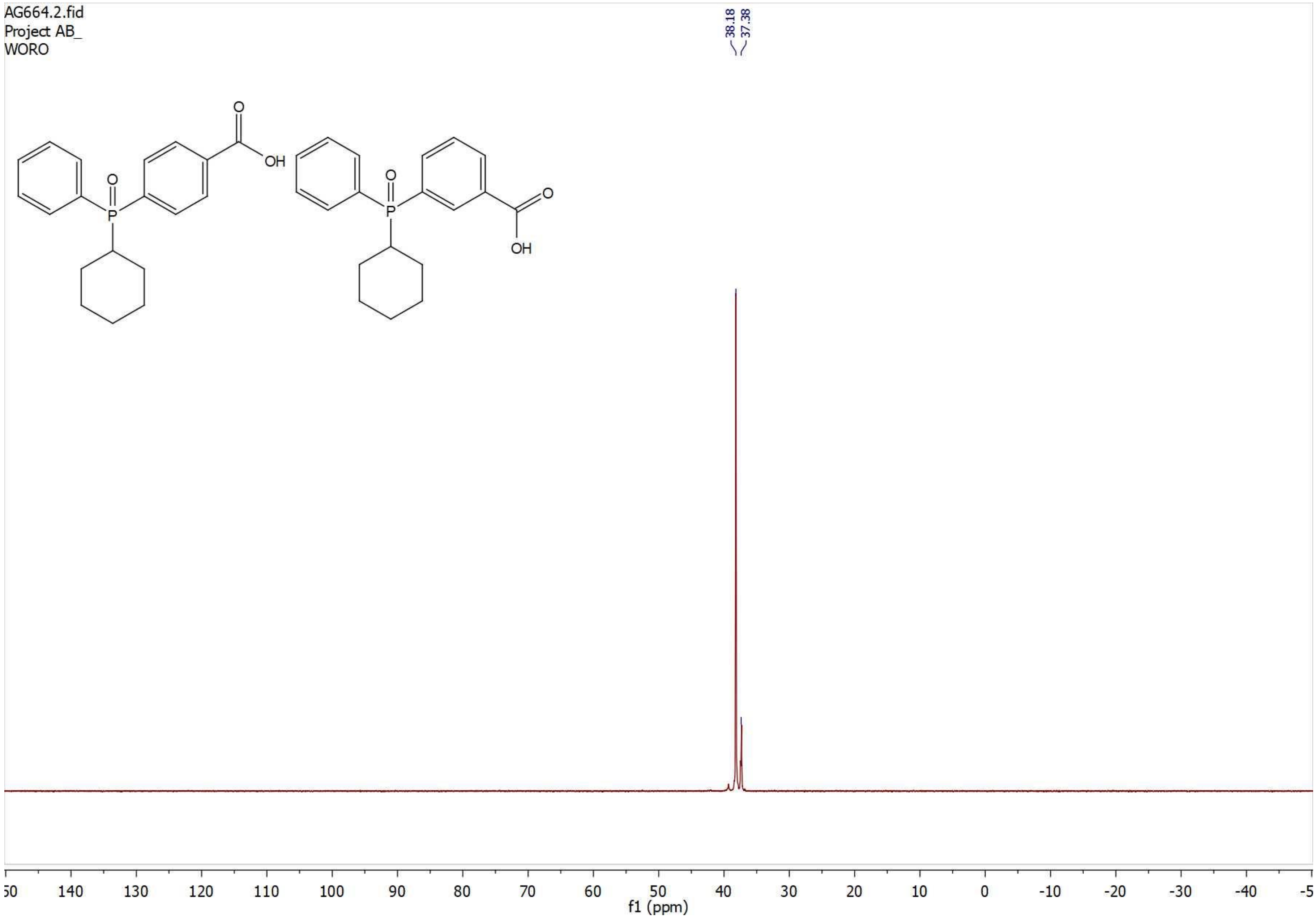

Supplement: Supplementary file 1 — Supplementary [file CHEM-26-6064-s001.pdf]
